# Direct Integration of Functionalized Bridges by One‐Step Superacid‐Catalyzed Reaction to Fabricate Porous Polymers for CO_2_ Capture and Separation

**DOI:** 10.1002/anie.202507863

**Published:** 2025-06-17

**Authors:** Jacopo Perego, Sergio Piva, Charl Xavier Bezuidenhout, Angiolina Comotti, Piero Sozzani, Silvia Bracco

**Affiliations:** ^1^ Department of Materials Science and INSTM Research Unit University of Milano‐Bicocca Via R. Cozzi 55 Milan 20125 Italy

**Keywords:** Carbon dioxide capture, Dynamic breakthrough, Microporous materials, NMR spectroscopy, Pre‐synthetic functionalization

## Abstract

A novel class of ultra‐microporous functionalized porous organic polymers (POPs) was developed starting from glyoxylic acid as a cross‐linker and triflic acid as a catalyst on polyaromatic monomers, generating in situ methine bridges with carboxylic acids. This one‐pot synthetic method generated functionalized POPs with high connectivity per each aromatic group and a high density of aliphatic carboxylic acids decorating the pore walls. Remarkably, the functional groups were transformed into esters, Na‐ and Li‐carboxylates by post‐synthetic modification with high yields, generating polyionic porous polymers. These porous polymers displayed excellent CO_2_ adsorption at 298 K and isosteric heat of adsorption with values as high as 50 kJ mol^−1^ for the Na‐containing POP endowed with numerous ionic charges, as estimated by direct measurements with microcalorimetry coupled to CO_2_ adsorption isotherms. Dynamic breakthrough experiments on self‐supporting monolithic composites demonstrated high selectivity for CO_2_ adsorption over N_2_ up to 500 for diluted streams and 340 under relevant conditions for carbon capture from flue gases (0.15 CO_2_ partial pressure).

## Graphical abstract

The one‐step polymerization of unfunctionalized polyaromatic monomers and glyoxylic acid under superacid‐catalysis conditions promotes the simultaneous generation of highly porous 3D architectures and the controlled decoration of pore surfaces with aliphatic carboxylic acids. These ultra‐microporous frameworks and their ester and polyionic derivatives, based on Li^+^ and Na^+^ carboxylate, demonstrate exceptional affinity for CO_2_ and CO_2_/N_2_ separation capabilities.

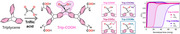

## Introduction

Carbon capture in solid sorbents with low‐energy regeneration processes is an area of extreme importance for the protection of the environment and enables CO_2_ recovery to synthesize added‐value products.^[^ ^1^, ^2^, ^3^ ^]^ Besides the renowned families of porous materials formed by coordination bonds, hydrogen bonding, and covalent bonds,^[^ ^4^, ^5^, ^6^, ^7^, ^8^, ^9^ ^]^ a promising class of materials for gas capture, selective separation, and storage consists of porous organic polymers (POPs).^[^ ^10^, ^11^, ^12^, ^13^, ^14^ ^]^ The most effective reactions for achieving high connectivity in POPs involve either direct carbon−carbon bond formation by Yamamoto, Sonogashira, and Suzuki coupling or molecular bridge insertion among rigid aromatic groups (Friedel–Crafts reactions).^[^ ^15^, ^16^, ^17^ ^]^ POPs hold great potential for implementation in industrial processes due to the high surface area, high chemical and thermal stability of the framework even under harsh conditions, and inexpensive preparation from aromatic monomers in high yields.^[^ ^18^, ^19^, ^20^, ^21^, ^22^, ^23^, ^24^, ^25^, ^26^, ^27^, ^28^, ^29^, ^30^ ^]^

The presence of functional groups in pore walls offers the advantage of creating customized porous materials, which can foster innovative applications. Two distinct strategies can be applied for functionalization, namely pre‐ and post‐functionalization.^[^ ^31^, ^32^, ^33^ ^]^ The use of pre‐functionalized monomers enables the generation of materials with a controlled and homogeneous distribution of functional groups, although it is limited by the synthetic efforts for monomer preparation and severe reaction conditions for the framework formation.^[^ ^34^, ^35^, ^36^, ^37^, ^38^, ^39^ ^]^ The post‐functionalization of a formerly synthesized porous material, which implies a two‐step process, provides a versatile strategy but results in less‐controlled reactions and may lead to a gradient distribution of functional groups in the framework.^[^ ^40^, ^41^, ^42^ ^]^

**Figure 1:**
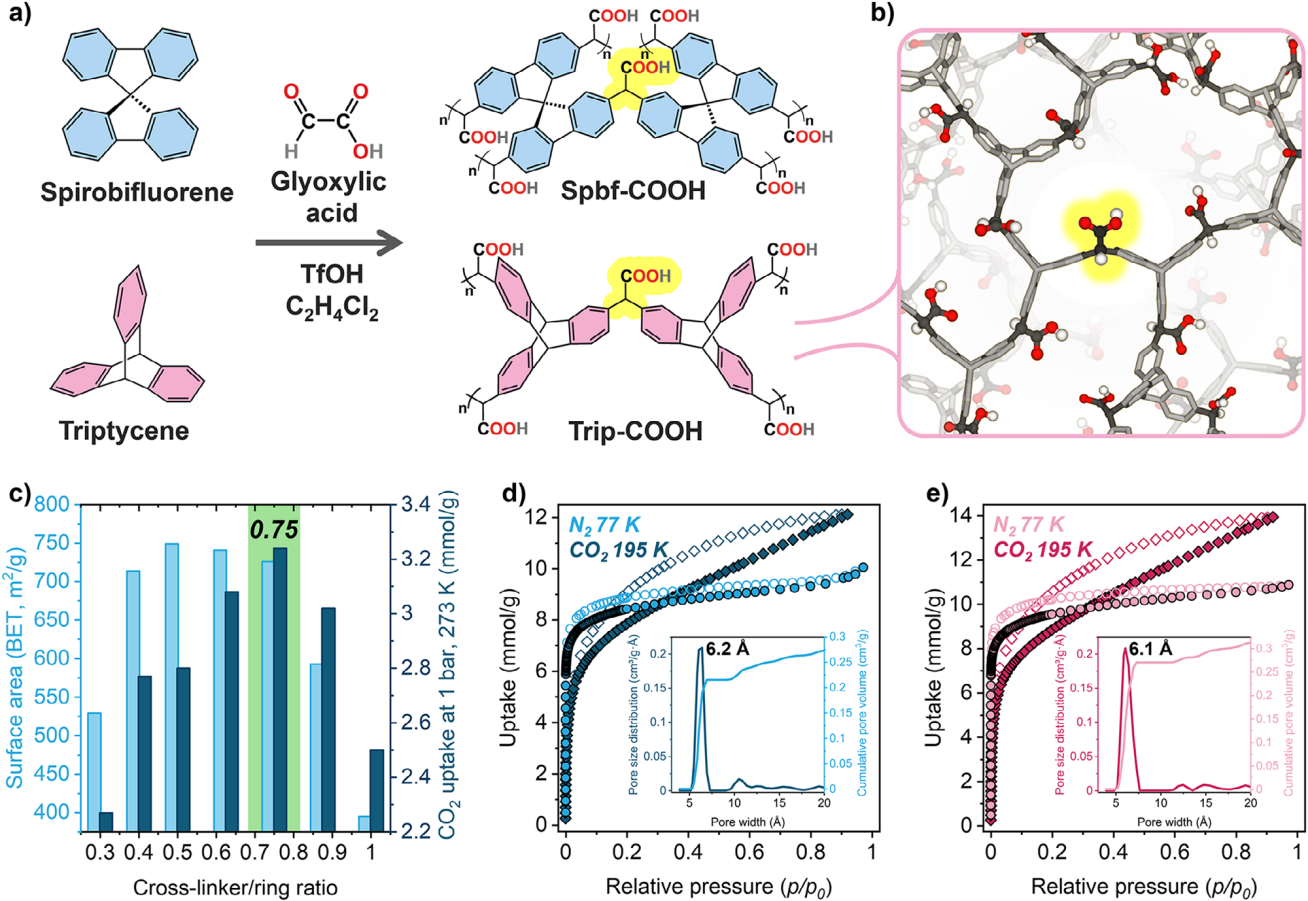
a) Chemical structure of triptycene and spirobifluorene monomers, polymerization reaction, and formation of **POP‐COOHs**. b) Schematic representation of the 3D structure of **Trip‐COOH**. c) BET surface areas and CO_2_ uptake at 1 bar and 273 K of **Spbf‐COOH** versus cross‐linker to phenyl ring ratio. N_2_ adsorption isotherms collected at 77 K up to 1 bar and CO_2_ adsorption isotherms collected at 195 K of d) **Spbf‐COOH** and e) **Trip‐COOH**. Inset: pore size distributions and cumulative pore volumes calculated from N_2_ adsorption isotherm between 0 and 20 Å according to the HS‐2D‐NLDFT theory and carbon slit pore model.

Herein, we report an innovative one‐step approach to connect non‐pre‐functionalized aromatic monomers by inserting functional groups simultaneously with the framework formation. Inspired by the reaction of phenyls with glyoxylic acid under superacid conditions as an effective route to form diarylacetic acid derivatives,^[^ ^43^ ^]^ we fabricated POPs decorated with a high density of accessible aliphatic carboxylic acids and derivatives starting from unfunctionalized aromatic monomers (Figure 1). The use of glyoxylic acid as a cross‐linking agent with a superacidic trifluoromethanesulfonic acid (TfOH) promoted the direct formation of covalent bridging units between the aromatic building blocks, each bearing a carboxylic acid, generating the precisely functionalized porous 3D framework. Furthermore, the carboxylic functionalities were easily transformed to ester and Li‐ and Na‐carboxylate groups in high yield, forming polyionic materials that maintained highly accessible porosity. The connectivity and quantification of functional groups and their derivatives were obtained by a combined approach of calorimetric and spectroscopic methods, including TGA coupled to IR spectroscopy and ss‐NMR. The functionalized POPs displayed high thermal and physicochemical stability and can be prepared on a gram scale using small amounts of organic solvents. The ultra‐microporous nature, combined with the presence of polar functional groups, ensured a high affinity for CO_2_ and excellent CO_2_/N_2_ selectivity values at room temperature for the polyionic frameworks. Self‐standing porous monoliths obtained as composites with polymers exhibited an exceptional CO_2_/N_2_ separation as demonstrated by dynamic breakthrough experiments under operative conditions suited for industrial applications, such as CO_2_ capture from flue gas.

## Results and Discussion

### Synthesis of Carboxyl‐Functionalized POP‐COOHs

Polyaromatic monomers such as triptycene (Trip) and spirobifluorene (Spbf) were selected owing to their topology with aromatic groups radiating from the core of the monomer, favoring the formation of low‐density porous 3D architecture. The monomers possess C_sp3_ carbons connecting three or four aromatic rings, respectively, which prevent a close‐packed architecture and confer rigidity to the framework. The monomers were reacted with glyoxylic acid and TfOH in dichloroethane at 273 K. Then, the reaction proceeded at 298 K for 72 h under stirring, and after washing with H_2_O/EtOH/acetone/CHCl_3_, the compounds were activated at 100 °C overnight to generate porous 3D frameworks (see Synthetic Methods).

A series of porous compounds starting from the spirobifluorene monomer were prepared, varying systematically the reaction time and the molar ratio between the glyoxylic acid and aromatic rings of monomer to fine‐tune the conditions and improve the cross‐linking and the carboxylic acid density without affecting the porosity of the 3D frameworks (Figure 1a,b). The optimized reaction time was established to be 72 h, and the influence of the amount of cross‐linker on the textural properties of the porous solid materials was tested by N_2_ and CO_2_ adsorption isotherms at 77 and 273 K, respectively, and thermogravimetric analysis (Figures 1c–e, S2–S14, and Table S2). N_2_ adsorption isotherms highlighted a higher surface area in the 0.5–0.75 ratio range of cross‐linker over number of rings (C/R), whilst CO_2_ adsorption uptake increased and achieved a maximum in the sample obtained with 0.75 C/R ratio owing to the increase of the carboxylic acid density decorating the pore walls (Figure 1c). This result agreed with the thermogravimetric analysis, which demonstrated the increasing weight loss above 300 °C along the series due to the promotion of decarboxylation (Figures S16 and S17). Further increase of cross‐linker density did not improve porosity, possibly due to the insertion of dangling groups. Thus, the most efficient synthetic conditions with 0.75 C/R ratio were applied to produce the porous polymers starting from spirobifluorene (Spbf) and triptycene (Trip) monomers, denominated **Spbf‐COOH** and **Trip‐COOH**, respectively. The N_2_ adsorption isotherms collected at 77 K displayed Langmuir behavior, typical of highly microporous compounds with the Langmuir and BET surface areas as high as 813 and 749 m^2^ g^−1^ for **Spbf‐COOH** and 890 and 822 m^2^ g^−1^ for **Trip‐COOH**, respectively (Figure 1d,e). Indeed, the pore size distributions calculated from N_2_ adsorption isotherms at 77 K, based on HS‐2D‐NLDFT theory, revealed the ultra‐microporous nature of the frameworks, with sharp and monodisperse distributions centered at ∼6 Å. Pore distribution analysis using a CO_2_ probe at 273 K confirmed the presence of pores with diameters below 7 Å and highlighted narrower cavities with diameters between 3.5 and 5 Å (Figure S20). The CO_2_ adsorption isotherms collected at 1 bar and 195 K indicated a maximum uptake of 12.1 and 13.9 mmol g^−1^ for **Spbf‐COOH** and **Trip‐COOH**, respectively, and exhibited hysteresis in the desorption curves, which closed only at low pressures due to the swelling of the frameworks at high loadings.^[^ ^44^ ^]^ The robustness of the synthetic procedure was demonstrated by preparing **Trip‐COOH** three times on a gram scale and verifying the reproducibility of the textural properties of the porous polymers (Figure S21 and Table S4).

**Figure 2:**
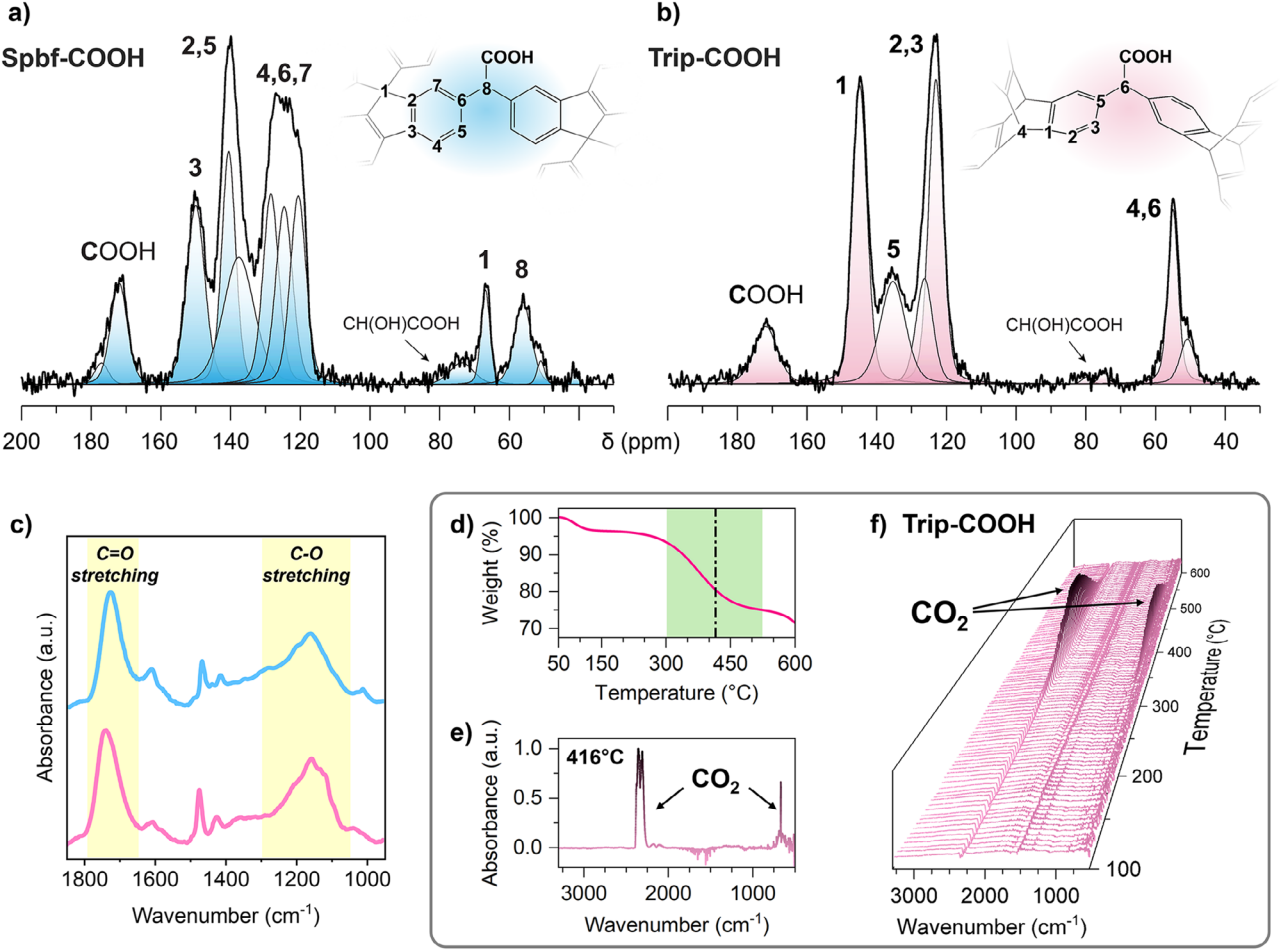
^13^C MAS NMR spectra of a) **Spbf‐COOH** and b) **Trip‐COOH** collected under quantitative conditions. c) FT‐IR spectra of **Spbf‐COOH** (light blue) and **Trip‐COOH** (pink) between 950 and 1850 cm^−1^. The signals associated with the C─O and C═O stretching of carboxylic acid groups are highlighted in yellow. d) TGA analysis between 50 and 600 °C under an inert atmosphere (N_2_ flow, 50 mL min^−1^). The green region highlights the temperature range of the decarboxylation reaction. e) FTIR spectrum coupled to TGA of **Trip‐COOH** measured at 416 °C, displaying the evolution of CO_2_ due to the degradation of carboxylic acid groups. f) 3D plot of the FTIR spectra of the evolved CO_2_ gas at different temperatures.

The composition and connectivity of the frameworks were revealed by ^13^C solid‐state NMR analysis and infrared spectroscopy. ^13^C MAS NMR spectra allowed to identify the presence of diphenylacetic moieties (Ph_2_CH‐COOH) and the quantification of the cross‐linking degree (Figures 2a,b, S22, and S23; Tables S5 and S6). In particular, the ─COOH, C5 and C6 signals of **Trip‐COOH** resonating at *δ* = 171.7, 136.0, and 55.4 ppm, respectively, were diagnostic of the bridging moieties. The area of aromatic carbons versus COOH signals corresponded to 12:1, demonstrating that each aromatic ring was substituted by one acetic bridging group. Similarly, in **Spbf‐COOH**, the diphenylacetic bridges involved the overall structure, and a quantitative substitution of the aromatic rings with acetic moieties corresponding to a 2:1 ratio was observed, as testified by chemical shifts at *δ* = 171.6 and 56.0 ppm of COOH and CH, respectively. In addition, we observed a minor amount of hydroxyacetic pendants with a 0.08 ratio per each aromatic group. ^1^H MAS NMR of both compounds collected at 600 MHz and 30 kHz spinning speed confirmed the above quantification (Figure S25). Infrared spectroscopy showed a strong and broad C═O stretching band at 1722 and 1730 cm^−1^ for **Spbf‐COOH** and **Trip‐COOH**, respectively, proving the presence of carboxylic groups (Figure 2c). A wide band resonating at about 1170 cm^−1^ was attributed to the C─O stretching.^[^ ^45^ ^]^

Thermal analyses of both samples revealed a weight loss from 300 to 500 °C, whose degradation product was identified to be CO_2_ by infrared spectroscopy coupled with thermal analysis (Figure 2d–f). The mass loss is associated with the decarboxylation reactions and accounts for 25 and 23.7 wt% of COOH groups for **Spbf‐COOH** and **Trip‐COOH**, respectively (Figures S27 and S28). These values correlated well with the content of functional groups established by ^13^C solid‐state NMR spectroscopy (22.7 and 21.9 wt% for **Spbf‐COOH** and **Trip‐COOH**, respectively), confirming the high density of functionalities installed in the porous frameworks. Elemental analysis (C, H, N, and O) was consistent with the composition derived from solid‐state NMR analysis, supporting the quantification of the carboxylic acids decorating the pore walls (Table S9).

### Pore Surface Engineering

**Figure 3:**
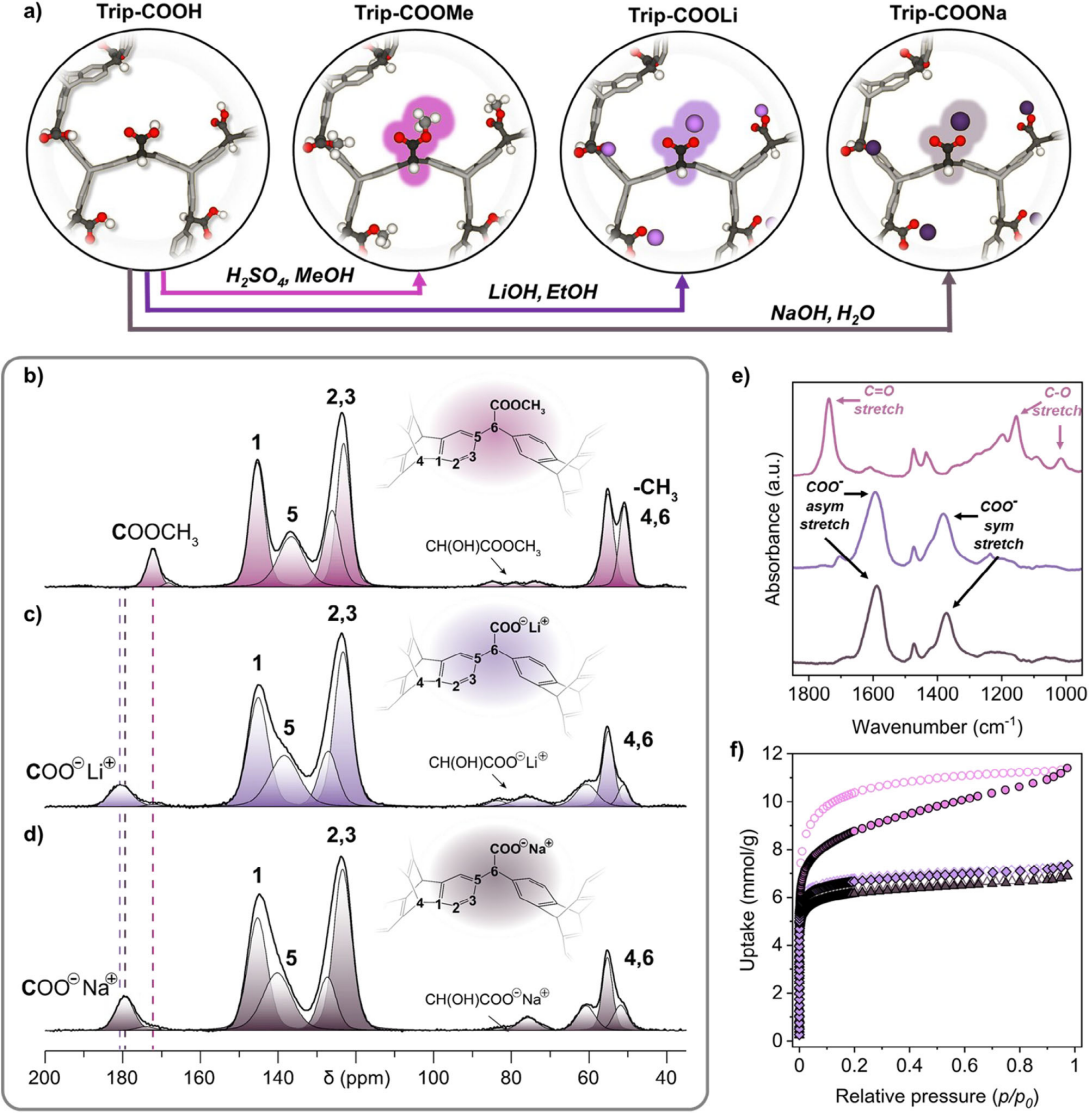
a) Post‐synthetic conversion of the carboxylic acid groups to ester and lithium/sodium carboxylate. ^13^C MAS NMR spectra of b) **Trip‐COOMe**, c) **Trip‐COOLi**, and d) **Trip‐COONa**. e) Infrared spectra and f) N_2_ adsorption isotherms at 77 K of **Trip‐COOMe** (pink), **Trip‐COOLi** (violet), and **Trip‐COONa** (grey).

The accessibility of the carboxylic acids decorating the pore walls and their reactivity enabled the promotion of in situ chemical transformations. The ester derivative of **Trip‐COOH** was obtained by diffusion into the pores of methanol and sulfuric acid and subsequent Fisher esterification in the confined environment (**Trip‐COOMe**), while treatments with LiOH in ethanol and NaOH in water generated polyionic porous polymers with high yield, denoted as **Trip‐COOLi** and **Trip‐COONa** (Figure 3a, see Synthetic Methods).^[^ ^46^, ^47^ ^]^

Solid‐state NMR provides an invaluable tool for investigating the chemical nature of the functional groups and quantifying their bulk conversion. ^13^C MAS spectrum of **Trip‐COOMe** showed a significant increase of the signal at *δ* = 51.0 ppm owing to the formation of methyl ester, indicating that 80.6% of carboxylic acids were converted to ester (Figure 3b). This value was confirmed by the quantification in ^1^H MAS NMR spectrum, displaying a resonance at *δ* = 3.2 ppm (Figure S36). Thermogravimetric analysis revealed a weight loss of 28.4 wt% due to the degradation of the methyl ester groups, corresponding to a 76% conversion yield (Figure S37), in agreement with the degree of methylation estimated by ss‐NMR.

The transformation of carboxylic acids into carboxylate groups in **Trip‐COOLi** and **Trip‐COONa** was demonstrated by the large downfield shift from *δ* = 172.5 to 180.2 ppm and 179.2 ppm, respectively, and resulted in quantitative conversion (90% of COO^−^ groups) (Figures 3c,d, S42, and S43; Tables S12 and S13), as observed in the fully relaxed ^13^C MAS NMR spectra. After thermal treatment under oxidative conditions, the residue analysis indicated a conversion of about 94% for **Trip‐COOLi** and 90% for **Trip‐COONa** (Figures S45 and S46), corroborating the quantification by ss‐NMR. The analysis of the absorption bands in the infrared spectra further corroborated the formation of the carboxylate derivatives. Regarding **Trip‐COOMe**, infrared spectroscopy showed strong absorption bands due to the C═O stretching at 1740 cm^−1^ and the C─O vibrations at 1157 and 1196 cm^−1^, and pointed out a new band at 1050 cm^−1^, characteristic of acetates of primary alcohols (Figure 3e).^[^ ^45^ ^]^ Moreover, vibrational bands at 2950 and 1440 cm^−1^ were associated with asymmetric CH_3_ stretching and symmetric CH_3_ deformation, respectively, supporting the effective esterification reaction (Figures 3e and S47). The generation of the lithium and sodium carboxylate moieties was revealed by the IR spectra, which show a strong asymmetric COO stretching vibration centered at 1597 and 1583 cm^−1^, and a symmetric COO stretching vibration at 1384 and 1371 cm^−1^, concerning **TripCOOLi** and **TripCOONa**, respectively (Figure 3e). The open porosity of the frameworks was proven by N_2_ adsorption isotherms at 77 K and CO_2_ adsorption isotherms at 195 K (Figures 3f, S48, S50, and S51). Langmuir and BET surface areas were estimated to be 795 and 733 m^2^ g^−1^ for **Trip‐COOMe**, 626 and 585 m^2^ g^−1^ for **Trip‐COOLi**, and 575 and 540 m^2^ g^−1^ for **Trip‐COONa**, demonstrating that the frameworks retain their porosity (Table S14). These successful results clearly demonstrate the easy accessibility of carboxylic acids in the framework and their effective chemical transformation, resulting in the fabrication of porous ionic polymers containing negative ionic charges in the porous skeleton counterbalanced by Li^+1^ and Na^+1^ charges. **Trip‐COONa** reverts to **Trip‐COOH** after acid treatment (HCl 0.1 M), as demonstrated by ^13^C MAS NMR spectrum and CO_2_ adsorption isotherm at 273 K (Figures S55–S57).

### CO_2_ Sorption and Separation

**Figure 4:**
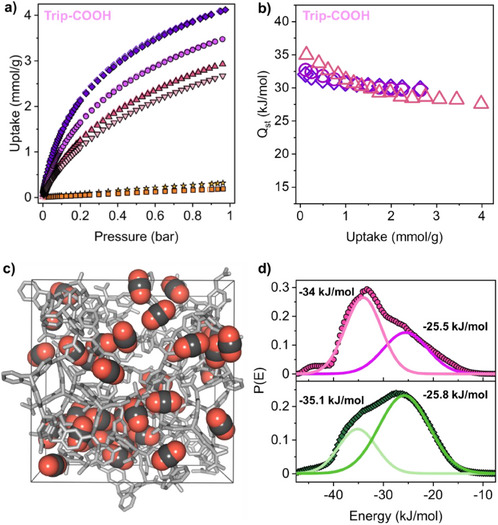
a) CO_2_ adsorption isotherms of **Trip‐COOH** collected at 273 K (diamonds, dark violet), 283 K (circles, purple), 293 K (up‐pointing triangles, pink), and 298 K (down‐pointing triangles, light pink). N_2_ adsorption isotherms collected at 273 K (stars, yellow) and 298 K (squares, orange). Filled and empty symbols represent sorption and desorption branches, respectively. b) Isosteric heat of adsorption (*Q*_st_) by direct measurement with microcalorimetry at 293 K (triangles, pink), calculated from the adsorption isotherms using the Van't Hoff equation (diamonds, dark violet) and the virial method (circles, purple). c) Snapshot of the CO_2_ molecules inside the amorphous cell of **Trip‐COOH** at a fixed pressure sorption simulation of 1 bar and 298 K. The CO_2_ molecules are displayed in CPK. d) Energy distribution of CO_2_ inside the simulated amorphous cell. The energy distribution was calculated at 298 K and pressures of 20 mbar (top) and 1 bar (bottom), respectively.

The ultra‐microporous nature and the high density of functional groups decorating the pore walls of **POP‐COOH** materials could be exploited for CO_2_ capture and separation. CO_2_ adsorption isotherms at distinct temperatures (273, 283, 293, and 298 K) were collected on spirobifluorene‐ and trypticene‐based materials. CO_2_ uptake at 273 K and 1 bar was as high as 4.1 and 3.2 mmol g^−1^ for **Trip‐COOH** and **Spbf‐COOH**, respectively (Figures 4a and S10). CO_2_ adsorption isotherms coupled to microcalorimetry allowed for the direct measurement of the isosteric heat of adsorption at 293 K, which corresponds to 35.0 kJ mol^−1^ at low coverage for **Trip‐COOH** (Figures 4b and S63) due to the favorable interactions between CO_2_ molecules and the carboxylic groups exposed toward the pores. The values are in excellent agreement with those calculated from variable temperature single‐component sorption data and applying the virial analysis and the Van't Hoff equation (Figure 4b). Computational analysis supported experimental observations. A model of the amorphous cell was built by connecting triptycene moieties with methine bridges bearing carboxylic acid units whose calculated density corresponds to the experimental one obtained from helium pycnometer and N_2_ sorption measurements (*ρ*_exp_ = 0.93 g cm^−3^ vs. *ρ*_calc_ = 0.97 g cm^−3^), then the amorphous cell was annealed and optimized (see SI: methods). Using Grand Canonical Monte Carlo methods, the calculated CO_2_ adsorption isotherm at 298 K displayed an uptake of 2.66 mmol g^−1^ at 1 bar, which matched the experimental value (2.67 mmol g^−1^ at 0.96 bar, Figure S65). Preferential adsorption sites for the CO_2_ molecules showed multiple favorable interactions between the host framework and the guest molecules, specifically between the carboxylic acid groups or aromatic rings and CO_2_ molecules. The energy distribution profile P(E) for CO_2_ molecules at 20 mbar showed a prevailing peak at Δ*H* = −34 kJ mol^−1^ associated with the preferential sorption sites (Figure 4d), in excellent agreement with the isosteric heat of adsorption at low coverage, as measured directly by microcalorimetry. On increasing CO_2_ loading, the CO_2_⋯CO_2_ interactions prevail, resulting in a major peak at −25.8 kJ mol^−1^.

CO_2_ can be easily released at 298 K under vacuum for 3 h, and the compounds displayed high cyclability and reproducibility of CO_2_ uptake with no fatigue after several cycles (Figure S66). Additionally, after prolonged exposure (10 days) to water vapors (RH ≈ 85%), acidic vapor (humid HCl), and soaking in water at 25 and 90 °C and in HCl solution (0.1 and 1 M), **Trip‐COOH** retained at least 97% CO_2_ sorption capacity at 1 bar and 273 K, demonstrating exceptional chemical and humidity stability (Figure S67).

**Figure 5:**
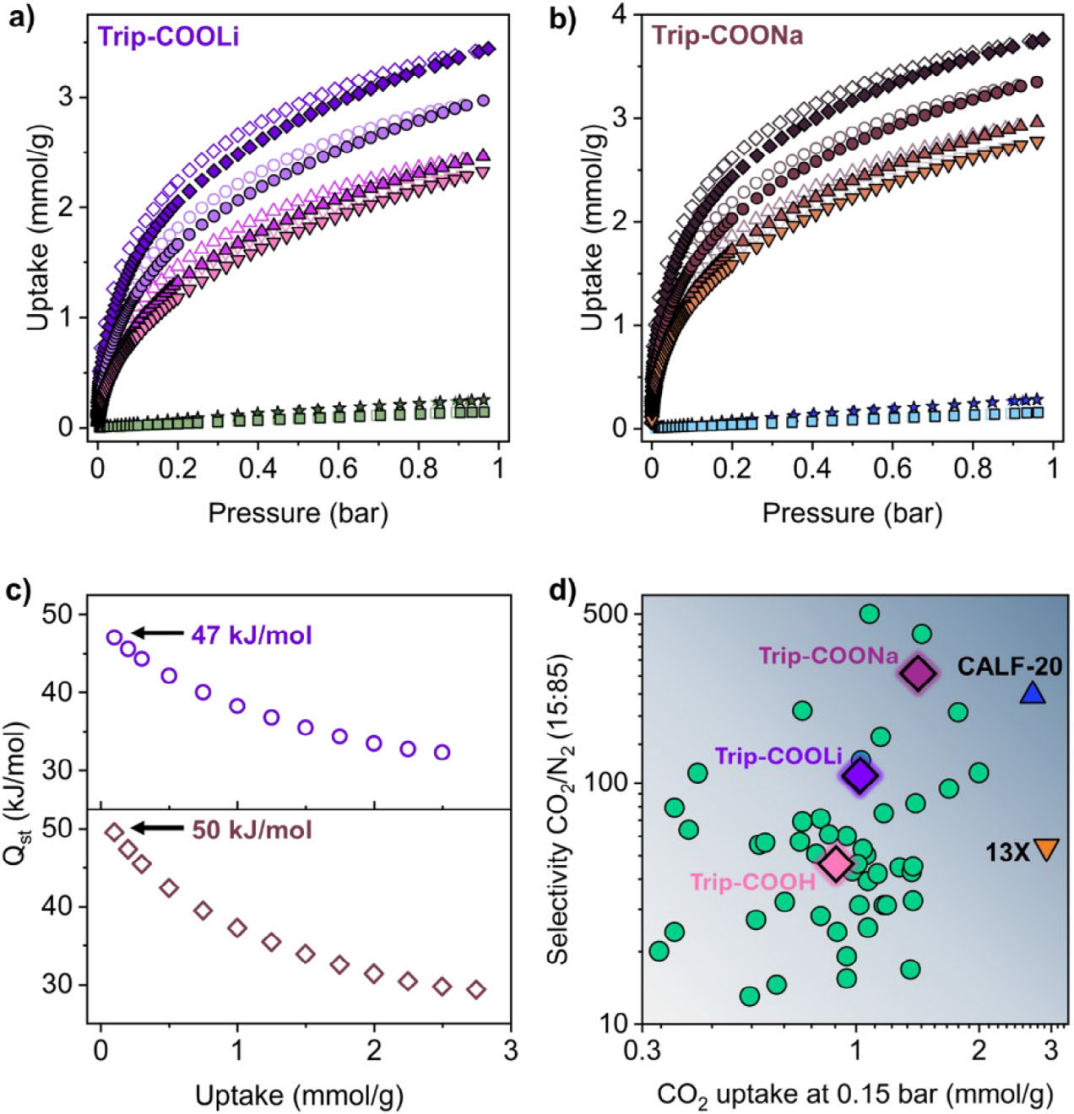
a) CO_2_ adsorption isotherms of **Trip‐COOLi** collected at 273 K (diamonds, dark violet), 283 K (circles, violet), 293 K (up‐pointing triangles, dark pink), and 298 K (down‐pointing triangles, pink). N_2_ adsorption isotherms collected at 273 K (stars, dark green) and 298 K (squares, light green). Filled and empty symbols represent sorption and desorption branches, respectively. b) CO_2_ adsorption isotherms of **Trip‐COONa** collected at 273 K (diamonds, dark brown), 283 K (circles, brown), 293 K (up‐pointing triangles, light brown), and 298 K (down‐pointing triangles, sand). N_2_ adsorption isotherms collected at 273 K (stars, dark blue) and 298 K (squares, light blue). Filled and empty symbols represent sorption and desorption branches, respectively. c) CO_2_ isosteric heats of adsorption (*Q*_st_) calculated from the isotherms collected at different temperatures of **Trip‐COOLi** (top) and **Trip‐COONa** (bottom) according to the virial method. d) CO_2_/N_2_ (15:85, total pressure = 1 bar) selectivity calculated at 298 K plotted against the CO_2_ sorption capacity measured at 0.15 bar from single‐component CO_2_ adsorption isotherm performed at 298 K for the best‐performing porous organic polymers. The diamonds display the performances of **Trip‐COOH** (pink), **Trip‐COOLi** (violet), and **Trip‐COONa** (dark purple), respectively.

Regarding the nanoporous polyionic frameworks, **Trip‐COOLi** and **Trip‐COONa** generated primary adsorption sites that effectively interacted with quadrupolar CO_2_ molecules, resulting in steeper adsorption profiles at low pressure than the parent framework. Indeed, at 0.15 bar and 298 K, the CO_2_ uptakes of **Trip‐COOLi** and **Trip‐COONa** reach values of 1.02 mmol g^−1^ (4.5 wt%) and 1.4 mmol g^−1^ (6.2 wt%) (Figure 5a,b), respectively, with increased uptakes of 13% and 56% compared to the parent **Trip‐COOH**. The porous matrix of **Trip‐COONa** can be easily regenerated with a temperature swing of 100 °C under a vacuum, and after several CO_2_ adsorption/desorption cycles, no fatigue in CO_2_ uptake is observed (Figure S68). **Trip‐COONa** material is unaltered after water vapors (RH ≈ 85%), soaking in water (at 25 and 90 °C) and basic solution (NaOH, 1 M) (Figure S69). The isosteric heat of adsorption, calculated according to the Van't Hoff equation, was as high as 47 kJ mol^−1^ for **Trip‐COOLi** and 49 kJ mol^−1^ for **Trip‐COONa** (Figure 5c), showing an increase of adsorbate–adsorbent affinity by coulombic interactions. Such high values are comparable or superior to the most performing POPs functionalized with polar groups.^[^ ^48^, ^49^, ^50^, ^51^ ^]^ Despite the efficient CO_2_ uptake, both polyionic frameworks adsorbed a negligible amount of N_2_ (less than ∼0.16 mmol g^−1^ at 1 bar and 298 K for both **Trip‐COOLi** and **Trip‐COONa**). Indeed, ideal adsorbed solution theory (IAST), evaluated using the single‐component isotherms, showed an exceptional CO_2_/N_2_ selectivity at 298 K that reaches values as high as 520 at low coverage (0.01 CO_2_ partial pressure, p_CO2_) and 284 at 0.15 p_CO2_ for **Trip‐COONa** (Table S15), promising applications for CO_2_ separation from flue gas. Under typical industrial operative conditions of 0.15 p_CO2_, the selectivity of **Trip‐COONa** outperforms most of the published POPs and is competitive with the top‐performing PPN‐SO_3_Li and *pym*‐CTF‐500 (Figure 5d and Table S15).^[^ ^48^, ^52^ ^]^ Additionally, the selectivity of **Trip‐COONa** overcomes that of well‐known CALF‐20 and zeolite 13X.^[^ ^53^, ^54^, ^55^ ^]^

### Dynamic Breakthrough Separation of CO_2_/N_2_ Mixtures

**Figure 6:**
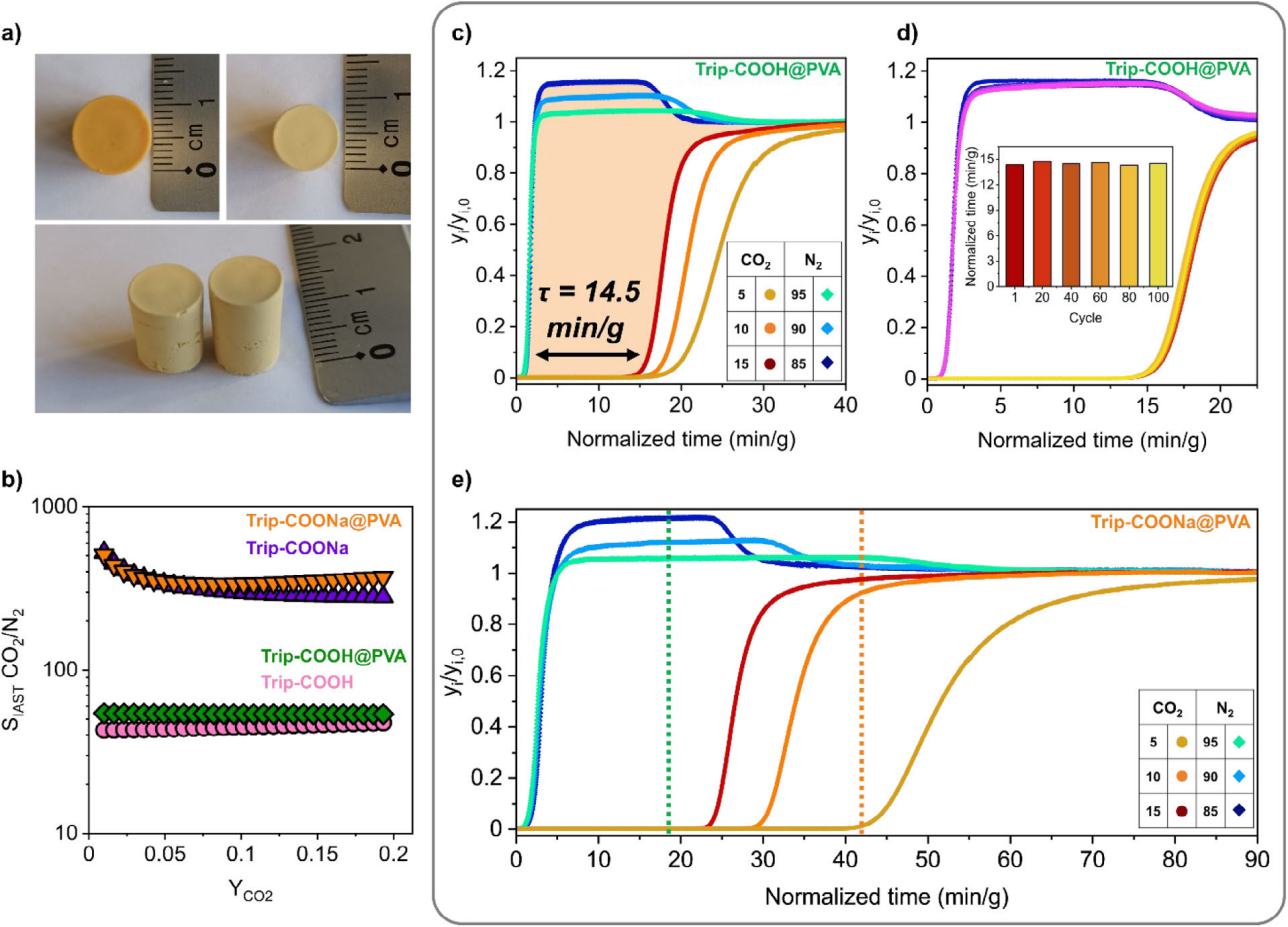
a) **Trip‐COOH@PVA** monoliths before (top, left) and after the activation process (top, right and bottom). b) CO_2_/N_2_ selectivity of **Trip‐COOH@PVA** (green diamonds) and **Trip‐COONa@PVA** (orange, down‐pointing triangles) between 0.01 and 0.2 CO_2_ partial pressure range estimated by IAST theory at 298 K and total pressure of 1 bar. The selectivity values for **Trip‐COOH** (pink circles) and **Trip‐COONa** (violet, up‐pointing triangles) were reported as a comparison. c) Column breakthrough curves for **Trip‐COOH@PVA** for CO_2_/N_2_ separation at different compositions (CO_2_ partial pressures of 0.05, 0.1, and 0.15) collected at 298 K and ambient pressure with a total flow of 6 sccm. d) Breakthrough cycles number 1, 20, 40, 60, 80, and 100 of **Trip‐COOH@PVA** for CO_2_/N_2_ separation (15:85 mixture) collected at 298 K. The material was regenerated after each cycle under He flow (6 sccm) at 298 K. The inset displays the breakthrough time for the isolation of N_2_ with a purity of 99%, highlighting the cyclability of **Trip‐COOH@PVA** over 100 cycles. e) Column breakthrough curves for **Trip‐COONa@PVA** for CO_2_/N_2_ separation at different compositions (CO_2_ partial pressures of 0.05, 0.1, and 0.15) collected at 298 K and ambient pressure with a total flow of 6 sccm. The orange dotted line highlights the longer CO_2_ breakthrough time for **Trip‐COONa@PVA** than that for **Trip‐COOH@PVA** (green dotted line).

The high uptake and favorable interactions of the frameworks with CO_2_ molecules, as well as the high stability under harsh conditions of functionalized **POP‐COOHs**, suggest their potential application in carbon capture and separation from exhaust gases. Breakthrough experiments were performed on the pelletized samples to prevent the disadvantages of powdery materials. Polyvinyl alcohol (PVA) is a valid matrix for the formation of porous composites with **POP‐COOHs** because of the favorable interactions of functional groups with hydroxylic groups of the polymer. Self‐supporting porous monoliths were produced by dispersing POP powders in an aqueous solution containing PVA, resulting in a PVA fraction of 10 wt% (see SI: Synthetic methods). The solvent was slowly replaced with acetone to promote the generation of porous POP‐PVA composite materials.^[^ ^56^ ^]^ The monoliths were extracted from the mold and activated at 100 °C under high vacuum (Figure 6a). The formation of the POP pellet, named **Trip‐COOH@PVA**, was demonstrated by ^13^C MAS NMR: the signals in the 65–75 ppm region corresponded to the CHOH groups forming one, two, and three intramolecular hydrogen bonds depending on the stereochemistry and the number of hydrogen bonds of the polymeric chain, while the signal at *δ* = 47.2 ppm is diagnostic of the CH_2_ units (Figure S70). Notably, the porous composite displayed high microporosity (surface area of 662 and 610 m^2^ g^−1^ calculated according to Langmuir and BET models, respectively) and retained high CO_2_ adsorption capacity at 298 K (0.83 mmol g^−1^, 3.7 wt% at 0.15 bar and 2.6 mmol g^−1^, 11.4 wt% at 1 bar) (Figures S76 and S77). The composite material with Na‐carboxylates, named **Trip‐COONa@PVA**, exhibited a higher CO_2_ uptake of 1.05 mmol g^−1^ (4.6 wt%) at 0.15 bar with respect to **Trip‐COOH@PVA**, according to the stronger interactions of the ionic sites with the quadrupolar CO_2_ molecules (Figure S78). Remarkably, the selective CO_2_ uptake versus N_2_ was retained entirely in **POP@PVA** composites with good CO_2_/N_2_ selectivity at 298 K up to 53 at 0.15 CO_2_ partial pressure for **TRIP‐COOH@PVA** and excellent values as high as 500 at low coverage (at 0.01 p_CO2_) and 340 at 0.15 partial pressure for **Trip‐COONa@PVA** (Figure 6b), demonstrating the positive role played by the presence of charges on the open pore walls of the functionalized framework.

**Trip‐COOH@PVA** was tested in breakthrough studies by diffusing CO_2_/N_2_ mixtures with a total flow rate of 6 sccm at 298 K under atmospheric pressure. When a binary gas‐mixture stream with a CO_2_ relative pressure of 0.15 diffused through the column, CO_2_ was completely removed from the mixture, while N_2_ with high purity (>99.0%) was collected for 14.5 min g^−1^, supporting the selective uptake suggested by single‐component isotherms (Figure 6c). Additionally, **Trip‐COOH@PVA** effectively separates CO_2_/N_2_ mixtures with different compositions (5%, 10%, 15%, and 20% of CO_2_ over N_2_) under continuous flow (Figures 6c and S81), showing longer breakthrough time up to 18 min g^−1^ for more CO_2_ dilute flows (N_2_ purity > 99%).^[^ ^55^ ^]^ Notably, the CO_2_ adsorbed in the porous composite can be easily recovered by flowing an inert gas along the column with no thermal treatment, minimizing the energy penalty for CO_2_ recovery (Figures S82 and S83). These mild activation conditions allowed the effective regeneration of the sorbent bed, as demonstrated by the high cyclability of the sample for 100 cycles (Figure 6d). It is worth noting that the exceptional CO_2_/N_2_ selectivity and increased CO_2_ uptake at low pressure of **Trip‐COONa@PVA** ensured the complete removal of CO_2_ with longer breakthrough times, especially at low CO_2_ partial pressure. Indeed, CO_2_ was effectively removed from a 5:95 CO_2_/N_2_ stream for 42 min g^−1^, an improvement of ∼2.3 times compared to **Trip‐COOH@PVA** (Figure 6e). Effective CO_2_ separation was also achieved for mixtures containing 0.1 and 0.15 CO_2_ partial pressure, which enabled the recovery of high‐purity nitrogen (>99.0%) for 29 and 23.5 min, respectively (Figure 6e). The excellent separation properties of **Trip‐COONa@PVA** can be entirely recovered by applying a thermal treatment of 120 °C for 3 h under He flow, resulting in high cyclability (Figure S84). Overall, these results clearly demonstrate that the polyionic nature and coulombic interactions of the highly functionalized microporous frameworks play a key role in the selective CO_2_ capture from industrial flue gases.

## Conclusion

We propose a novel one‐pot strategy for the concomitant fabrication of ultra‐microporous 3D frameworks and the installation of functional groups on the pore walls. The new synthetic method includes the simultaneous cross‐linking of aromatic groups and controlled pore surface decoration with carboxylic acids, leading to the realization of densely functionalized porous organic polymers. Additionally, the rigidity of the monomers with lateral aromatic arms promotes the generation of open pore skeletons. The generality of the reaction paves the way for using an extensive library of monomers employed in Friedel–Crafts alkylation reactions. Additionally, the fabrication of functionalized POPs at ambient temperature with high yield and the scalable synthetic conditions with minimal use of solvents make this process attractive for sustainable industrial applications. Moreover, the carboxylic acid groups inserted in the pore walls of the high‐surface area porous polymers can be easily accessed, as proven by the post‐synthetic modification reactions that can effectively generate the ester and polyanionic frameworks in quantitative yields. Triptycene‐based functional frameworks display high CO_2_ uptake at room temperature and a favorable isosteric heat of adsorption up to 50 kJ mol^−1^. PVA allowed the preparation of self‐supporting monoliths with **POP‐COOH** and **POP‐COONa** content as high as 90%, which retain the accessible microporosity and CO_2_ capture capacity of the porous frameworks. Continuous flow CO_2_/N_2_ separation was tested with breakthrough measurements highlighting the highly selective CO_2_ uptake with 99% purity, promising applications for CO_2_ capture from exhaust gases after combustion. The high density of functional groups could be exploited for several further applications, such as the selective capture of hazardous basic gases, e.g., NH_3_ and basic vapors, solid‐state cationic conductors (proton exchange polymers), and nanoporous materials for chiral separation. Preliminary results indicated that these functionalized materials can be used to create films and membranes.

## Conflict of Interests

The authors declare no conflict of interest.

## Supporting information



Supporting Information

## Data Availability

The data that support the findings of this study are available from the corresponding author upon reasonable request.
