# Supplementary material for: Direct Integration of Functionalized Bridges by One‐Step Superacid‐Catalyzed Reaction to Fabricate Porous Polymers for CO2 Capture and Separation
Source: Angew Chem Int Ed Engl. 2025 Jun 17;64(32):e202507863. doi: 10.1002/anie.202507863 (PMC12322657; doi:10.1002/anie.202507863)
Supplement: Supplementary file 1 — Supporting Information [file ANIE-64-e202507863-s001.docx]

**Direct Integration of Functionalized Bridges by One-step Superacid-Catalyzed Reaction to Fabricate Porous Polymers for CO_2_ Capture and Separation**

Jacopo Perego,*^[a]^ Sergio Piva,^[a]^ Charl Xavier Bezuidenhout,^[a]^ Angiolina Comotti,^[a]^ Piero Sozzani,^[a]^ Silvia Bracco*^[a]^

^[a ]^Dr. J. Perego, Dr. S. Piva, Dr. C. X. Bezuidenhout, Prof. A. Comotti, Prof. P. Sozzani, Prof. S. Bracco

Department of Materials Science and INSTM research unit

University of Milano-Bicocca

Via R. Cozzi 55, 20125, Milan, Italy

E-mail: jacopo.perego@unimib.it, silvia.bracco@unimib.it

Table of Contents:

- Synthetic Methods
- Experimental Details
- Optimization of the reaction conditions - **Spbf-COOH-x**

Gas adsorption measurements

Infrared spectroscopy

Thermal analysis

Powder X-ray diffraction

- Characterization of **Spbf-COOH** and **Trip-COOH**

Textural properties

^13^C and ^1^H solid-state NMR spectroscopy

Infrared spectroscopy

Thermal analysis

FT-IR coupled TGA analysis

Elemental Analysis

Powder X-ray diffraction

- Characterization of the post-functionalized samples **Trip-COOLi**, **Trip-COONa** and **Trip-COOMe**

^13^C and ^1^H solid-state NMR spectroscopy - **Trip-COOMe**

Thermal analysis - **Trip-COOMe**

FT-IR coupled TGA analysis – **Trip-COOMe**

^13^C and ^1^H solid-state NMR spectroscopy - **Trip-COONa** and **Trip-COOLi**

Thermal analysis - **Trip-COONa** and **Trip-COOLi**

Infrared spectroscopy

Gas adsorption measurements

Powder X-ray diffraction

- Adsorption-coupled calorimetry - **Trip-COOH**
- Computational analysis
- Reversibility, cyclability and stability of **Trip-COOH** and **Trip-COONa**
- Comparison with literature data
- Polymer-based composites: **Trip-COOH@PVA** and **Trip-COONa@PVA**

Infrared spectroscopy

^13^C and ^1^H solid-state NMR spectroscopy

Thermal analysis

FT-IR coupled TGA analysis

Powder X-ray diffraction

Gas adsorption measurements

- Continuous flow gas mixture separation (breakthrough experiments)
- POP-COOH@PVA membrane
- Supplementary References
- **Synthetic Methods**

All reagents were bought from commercial sources and used without any purification. Trypticene (98%), 9,9’-spirobifluorene (97%), glyoxylic acid monohydrate (98%), 1,2-dichloroethane (ACS reagent, ≥ 99%), acetone (ACS reagent, ≥ 99.5%), ethanol (≥ 99.5%), chloroform (≥ 99.8%), methanol (≥ 99.8%), sodium hydroxide (NaOH), lithium hydroxide (LiOH) and sulfuric acid (96%) were purchased from Sigma-Aldrich. Trifluoromethanesulfonic acid (Triflic acid, TfOH, ≥ 99%) was purchased from Fluorochem.

- Preparation of **Spbf-COOH-x** with different cross-linker to aromatic ring ratios.

In a Schlenk tube, 9,9’-Spirobifluorene was dissolved in 1,2-dichloroethane (DCE) at RT (see **table S1** for the details). Then, finely ground glyoxylic acid monohydrate (GA) was dispersed in the solution, and the mixture was cooled to 0°C using an ice/water bath under an inert atmosphere (nitrogen gas). A dropping funnel was mounted and filled with trifluoromethanesulfonic acid (TfOH, see **Table S1**). The triflic acid catalyst was added dropwise at 0°C under a nitrogen atmosphere. The mixture became dark blue, and a solid material started to precipitate. Once the addition was complete, the mixture was allowed to warm up to 25°C and was stirred for 72 hours. Then, the mixture was poured into water (~100 mL) and stirred for 3 hours. The light brown solid was filtered on a PTFE membrane (pore width < 0.2 µm) and washed with water (30 mL), acetone (30 mL), ethanol (30 mL), chloroform (30 mL) and acetone (30 mL). The solid was dried on the filter, recovered in a glass vial and activated under a high vacuum (p ≤ 3 µbar) at 100°C for 16 hours.

**Table S1**. Amounts of monomer and reagents used in preparing samples **spbf-COOH-x** with different cross-linker to aromatic ring ratio.

| **Sample** | **9,9′-Spirobifluorene**  **mg (mmol)** | **Glyoxylic acid monohydrate**  **mg (mmol)** | **Trifluoromethane-sulfonic acid**  **mL (mmol)** | **1,2-Dichloroethane**  **mL** |
| --- | --- | --- | --- | --- |
| **Spbf-COOH-1** | 400 (1.264) | 140 (1.521) | 0.9 (10.19) | 11 |
| **Spbf-COOH-2** | 400 (1.264) | 186 (2.022) | 1.25 (14.16) | 11 |
| **Spbf-COOH-3** | 400 (1.264) | 232 (2.520) | 1.50 (16.99) | 11 |
| **Spbf-COOH-4** | 400 (1.264) | 291 (3.161) | 1.95 (22.09) | 11 |
| **Spbf-COOH** | 400 (1.264) | 350 (3.793) | 2.3 (26.05) | 11 |
| **Spbf-COOH-5** | 400 (1.264) | 407 (4.424) | 2.7 (30.58) | 11 |
| **Spbf-COOH-6** | 400 (1.264) | 464 (5.040) | 3.1 (35.11) | 11 |

- Gram-scale preparation of **trip-COOH**.

In a PTFE-lined 100 mL reactor, triptycene (2 g, 7.86 mmol) was dissolved in 1,2-dichloroethane (DCE, 55 mL). Then, finely ground glyoxylic acid monohydrate (GA, 1.63 g, 17.70 mmol, 0.75 molecules *per* ring) was added to the solution. The mixture was stirred and cooled to 0°C with an ice/water bath, and trifluoromethanesulfonic acid (TfOH, 10 mL, 17 g, 113 mmol) was added dropwise under a nitrogen atmosphere. The mixture became dark red, and a solid material started to precipitate. Once the addition was complete, the mixture was allowed to warm up to 25°C and was stirred for 72 hours. Then, the mixture was poured into water (~200 mL) and stirred for 3 hours. The light brown solid was filtered on a PTFE membrane (pore width < 0.2 µm) and washed with water (50 mL), acetone (50 mL), ethanol (50 mL), chloroform (50 mL) and acetone (50 mL). The solid was dried on the filter, recovered in a glass vial and activated under a high vacuum (p ≤ 3 µbar) at 100°C for 16 hours. After drying, the final yield was ~2.91 g.

The gram-scale preparation was repeated three times to determine its reproducibility. N_2_ at 77 K and CO_2_ at 273 K of each sample (denoted **Trip-COOH**, **Trip-COOH_a** and **Trip-COOH_b**, respectively) were measured to test the reproducibility of the synthetic procedure (**figure S21** and **table S4**).

- **Post-modification** of the carboxylic acid functional groups.

Preparation of **trip-COONa**

**Trip-COOH** (900 mg) was suspended in a sodium hydroxide solution (0.5 M, 73.5 mL). Then, two freeze-pump-thaw processes were performed, and the mixture was stirred for 24 hours at RT. The suspension was filtered on a PTFE membrane (pore width < 0.2 µm) and washed with water (20 mL), ethanol (50 mL) and acetone (50 mL). The solid was dried on the filter, recovered in a glass vial and activated under a high vacuum (p ≤ 3 µbar) at 120°C for 24 hours.

Preparation of **trip-COOLi**

**Trip-COOH** (700 mg) was suspended in a lithium hydroxide solution in ethanol. Then, two freeze-pump-thaw processes were performed, and the mixture was stirred for 24 hours at RT. Then, the suspension was filtered on a PTFE membrane (pore width < 0.2 µm) and washed with water (20 mL), ethanol (50 mL) and acetone (50 mL). The solid was dried on the filter, recovered in a glass vial and activated under a high vacuum (p ≤ 3 µbar) at 120°C for 24 hours.

Preparation of **trip-COOMe**

**Trip-COOH** (900 mg) was suspended in 18 mL of methanol, and 5.4 mL of concentrated sulphuric acid (96%) was added. Then, two freeze-pump-thaw processes were performed before heating the mixture at 85°C for 12 hours under stirring. The suspension was filtered on a PTFE membrane (pore width < 0.2 µm) and washed with methanol (50 mL) and acetone (50 mL). The solid was dried on the filter, recovered in a glass vial and activated under a high vacuum (p ≤ 3 µbar) at 100°C for 16 hours.

- Preparation of self-standing polymer composite **trip-COOH@PVA and Trip-COONa@PVA**

**Trip-COOH** powder (270 mg) was packed in a 5 mL glass vial. Then, 1 mL of a PVA solution in deionized water (30 mg/mL) was diffused. Once the powder was wetted entirely, acetone (0.75 mL) was layered on the top of the wet powder to slowly replace the water and induce PVA aerogel formation. Every 24 hours, the supernatant liquid was replaced with 1 mL of acetone. The exchange process was repeated for 5 days. Then, the liquid was removed, and the glass vial was broken to recover a self-standing monolith, which was immersed in acetone (20 mL) for 2 days to exchange the water with acetone completely. The monolith was dried under a high vacuum (p ≤ 3 µbar) for 16 hours at 100°C to remove the solvent.

**Trip-COONa** powder (270 mg) was packed in a 5 mL glass vial. Then, 1.5 mL of a PVA solution in deionized water (20 mg/mL) was diffused. Once the powder was wetted entirely, acetone (0.75 mL) was layered on the top of the wet powder to slowly replace the water and induce PVA aerogel formation. Every 24 hours, the liquid was replaced with 1 mL of acetone. The exchange process was repeated for 5 days. Then, the liquid was removed, and the glass vial was broken to recover a self-standing monolith, which was immersed in acetone (20 mL) for 2 days to exchange the water with acetone completely. The monolith was dried under a high vacuum (p ≤ 3 µbar) for 24 hours at 120°C to remove the solvent.

- **Experimental Details**

**Elemental Analysis**

CHNS analysis was performed using an Elementar vario MICRO cube. The samples were treated overnight at 100°C under a high vacuum (p ≤ 3 µbar) for at least 12 hours before CHNS measurements.

**Gas Sorption and pore size distribution analysis from CO_2_ isotherms at 273 K**

N_2_ and CO_2_ adsorption experiments were performed using gases with a purity of 5.0 and 4.5, respectively. N_2_ at 77 K, 273 K and 298 K, and CO_2_ adsorption at 195 K, 273 K, 283 K, 293 K and 298 K were collected on a Micromeritics ASAP 2020 HD instrument or a Micromeritics 3Flex instrument. The samples were treated overnight at 100°C under a high vacuum (p ≤ 3 µbar) for at least 12 hours before sorption measurements. The temperature was controlled using a Julabo F12-ED refrigerated/heating circulator. The pore size distributions were calculated according to the HS-2D NLDFT theory for CO_2_ adsorption isotherm at 273 K using the carbon slit pore model as implemented in the Microactive software (Micromeritics).

**N_2_ adsorption isotherms at 77 K and surface area/pore size distribution analysis**

N_2_ adsorption isotherms collected at 77 K were analyzed using the Microactive software. The BET equation was applied to a pressure range of 0.015<*p/p_0_*<0.06, according to the Rouquerol plot analysis. The same pressure range was employed to calculate the Langmuir surface area. The pore size distributions were calculated according to the HS-2D NLDFT theory by N_2_ adsorption isotherms at 77 K using the carbon slit pore model as implemented in the Microactive software (Micromeritics).

**Isosteric heat (Qst) of adsorption calculation**

The isosteric heats of adsorption were calculated from the CO_2_ experimental isotherms collected at 273 K, 283 K, 293 K and 298 K. The Virial method and the Van’t Hoff equation were applied. For the Van’t Hoff analysis, the isotherms were fitted using the Langmuir-Freundlich equation, which provided the best fitting of the experimental data. The virial method gave a better fit for the adsorption isotherms. Remarkably, the two different methods provided consistent results.

**Infrared Spectroscopy (FT-IR)**

Fourier-transform infrared spectroscopy was performed using a Nicolet iS20 FTIR spectrometer equipped with an ATR module in air. The spectra were accumulated 128 times with a resolution of 4 cm^-1^. The samples were treated at 100°C under high vacuum (p ≤ 3 µbar) before the measurement to remove the adsorbed molecules.

**Solid-State NMR Spectroscopy**

^13^C and ^1^H solid-state NMR experiments were carried out at 75.5 and 300.1 MHz, respectively, with a Bruker Avance Neo instrument operating at a static field of 7.04 T equipped with a 4 mm double resonance MAS probe. ^13^C {^1^H} ramped-amplitude Cross Polarization (CP)^[1]^ experiments were performed at room temperature at a spinning speed of 12.5 kHz using a recycle delay of 5 s and contact times of 2 and 0.05 ms. The 90° pulse for the proton was 2.5 μs. Single-Pulse Excitation (SPE) experiments were run using a 90° pulse of 3.84 μs and a recycle delay of 60 s. Crystalline polyethylene was taken as an external reference at 32.8 ppm from TMS.

^13^C T_1_ spin-lattice relaxation times were measured at 75.5 MHz by applying the Torchia pulse sequence. ^[2]^ The experiment was collected at 298 K with a spinning speed of 12.5 kHz.

Single-Pulse Excitation (SPE) experiments (without CP) were run using a 90° pulse of 3.84 μs and satisfying the full relaxation conditions of full relaxation of 5 times the ^13^C T_1_ values (recycle delays of 60 s and 120 s).

Quantitative solid-state ^1^H SPE MAS NMR spectra were also performed with a Bruker Avance III 600 MHz instrument operating at 14.1 T, using a recycle delay of 20 s. A MAS Bruker probe head was used with 2.5 mm ZrO_2_ rotors spinning at 30 kHz. The 90° pulse for the proton was 2.9 μs. The ^1^H chemical shift was referenced to adamantane.

Spectra simulations were performed by the DMFIT program.^[3]^

**Thermogravimetric Analysis (TGA)**

Thermogravimetric analyses were performed using a Mettler Toledo Star System 1 equipped with a gas controller GC10. Samples were outgassed overnight at 100 °C under a high vacuum (p ≤ 3 µbar) before the analysis to remove adsorbed species and inserted in a 70 μL alumina pan in air.

TGA analysis performed under an oxidative atmosphere (dry air, flow rate = 50 mL/min) from 30°C to 1000°C highlighted the thermal stability of the samples and allowed the evaluation of the residue after the degradation of the organic component.

TGA analysis performed under an inert atmosphere (N_2_, flow rate = 50 mL/min) from 50°C to 600°C revealed a weight loss between 300°C and 500°C associated with the decarboxylation reaction of the frameworks.

**Coupled TGA-IR Analysis**

Coupled TGA-IR analysis were performed on samples **Spbf-COOH**, **Trip-COOH**, **Trip-COOMe** and **Trip-COOH@PVA** using a temperature ramp of 15°C/min from 50°C to 600°C under N_2_ atmosphere (flow rate = 50 mL/min). The outlet of the TGA was connected using a heated transfer line (T = 250°C) to a TGA-IR Module for Nicolet™ FTIR Spectrometers, which features a 10 cm pathlength nickel-plated aluminum flow cell.

**Adsorption-Coupled Calorimetry**

Sorption-coupled microcalorimetry enabled the direct measurement of the enthalpy variation associated with CO_2_ adsorption.^[4,5]^ The calorimetry data were recorded on a Setaram μDSC7 Evo instrument equipped with a high-pressure sample holder. CO_2_ dosing and adsorption isotherm collections were performed with a Micromeritics ASAP 2050 adsorber coupled to the μDSC module. The set-up allowed simultaneous determination of CO_2_ adsorption isotherms and heat exchanged during the adsorption process at each adsorption step. Sorption-coupled microcalorimetry measurements were performed twice at 293 K, and the two different runs were averaged to reduce experimental errors. Data acquisition and processing were performed as detailed in the literature.^[6,7]^

**He picnometry**

The density of the pore walls (ρ_w_) was measuread by He picnometry (Micromeritics). The apparent density of the materials was evaluated by considering pore-wall density and pore volume (ρ_a_) as determined by N_2_ adsorption isotherm at 77 K by the following equation ρ_a_ = 1 / (V_p_ + 1/ρ_w_).

**Computational details**

An amorphous cell containing 31 triptycene molecules was constructed with cell dimensions of 28.25 x 28.25 x 28.25 Å. This cell size was chosen to reproduce the experimental density of the material calculated from the He pycnometry density measurements (skeletal density) and considering the pore volume determined from the analysis of the N_2_ adsorption isotherm at 77 K (ρ_exp_ = 0.93 cm^3^/g against ρ_calc_ = 0.97 cm^3^/g). The unit cell was optimized in the *P1* space group using molecular mechanics. Molecular mechanics simulations were carried out using the Forcite module within the Biovia Materials Studio software suite. The details of the optimization are reported below. The model was compared against experimental data. The calculated and experimental micropore volumes (V_p,calc_ = 0.30 cm^3^/g against V_micropore,exp_ = 0.30 cm^3^/g) are in perfect agreement.

| *Forcite geometry optimization parameters* | |
| --- | --- |
| Quality | Ultra-fine |
| Algorithm | Quasi-Newton |
| *Energy parameters* | |
| Forcefield | Dreiding |
| Quality | Ultra-fine |
| *Summation method* | |
| Electrostatic | Atom based |
| Van der Waals | Atom based |

The CO_2_ adsorption isotherm simulation at 298 K was performed using the Sorption module within the Biovia Materials Studio software suite, enabling a direct comparison with the experimental observations (Figure S65). The Sorption module calculated the loading of the CO_2_ molecules in the amorphous cell model over a range of total fugacities (0.20 ≤ *f*(Kpa) ≤ 100) at a constant temperature (T = 298 K). The details of the sorption simulation are reported below.

| *Sorption calculation parameters* | |
| --- | --- |
| Method | Configurational bias |
| Equilibration steps | 100000 |
| Production steps | 1000000 |
| Fugacity steps (logarithmic scale) | 40 |
| Exchange | 3 |
| Regrow | 2 |
| Twist | 1 (Amplitude = 5°) |
| Rotate | 1 (Amplitude = 5°) |
| Translate | 1 (Amplitude = 1Å) |
| *Energy parameters* | |
| Forcefield | Dreiding |
| Quality | Fine |
| *Summation method* | |
| Electrostatic | Ewald & Group |
| Van der Waals | Atom based |

Single-point adsorption simulations at fixed pressures were calculated using the Sorption module within the Biovia Materials Studio software suite. Two simulations at fixed pressures of 2 Kpa (20 mbar), and 100 Kpa (1000 mbar), at a temperature of 298 K were carried out. The details of the fixed-pressure sorption simulations are reported below.

| *Sorption calculation parameters* | |
| --- | --- |
| Method | Configurational bias |
| Equilibration steps | 5000000 |
| Production steps | 25000000 |
| Exchange | 3 |
| Regrow | 2 |
| Twist | 1 (Amplitude = 5°) |
| Rotate | 1 (Amplitude = 5°) |
| Translate | 1 (Amplitude = 1Å) |
| *Energy parameters* | |
| Forcefield | Dreiding |
| Quality | Ultra-fine |
| *Summation method* | |
| Electrostatic | Ewald & Group |
| Van der Waals | Atom based |

**Breakthrough Analysis**

*Breakthrough experimental set-up*

Breakthrough experiments were performed using gases with a purity of 5.0 (N_2_ and He) and 4.5 (CO_2_). The breakthrough curves were collected using a Micromeritics SAA (Selective Adsorption Analyzer) coupled with a mass spectrometer (Pfeiffer Vacuum ThermoStar GSD 301 T3 Benchtop Mass Spectrometer) placed at the exit of the breakthrough column. The mass spectra signals have been recorded against time. The opening times of the valve, the temperature of the sample and the environmental chamber and the flow rates were recorded and stored. Typical mass spectrometry and breakthrough data are reported below (Figure S1).^[8,9,10]^

*Dead volume and MS response correction*

The measured breakthrough curves were analyzed and corrected for the system dead volume and the detector (MS) response. The blank curves were collected by filling the breakthrough column with glass beads of ~ 1 mm. The volume of glass beads was the same as the volume occupied by the sample. It was evaluated by measuring the density of the glass using He pycnometry and weighing the correct amount of inert material inside the column. Thus, the dead volume of the blank correction is equal to the empty volume in the presence of the sample. Breakthrough experiments with the dense glass beads were conducted using the same parameters, such as the total flow rate, temperature, CO_2_/N_2_ mixture compositions, as the breakthrough measurements performed with the porous polymers and the measured dead time was subtracted from the raw data. Typical values for the dead time correction were about 30 seconds for a total flow of 6 sccm, while the CO_2_ breakthrough times were about 11 minutes under the same conditions.

The breakthrough data were normalized against the masses of the samples of 635.5 mg for **Trip-COOH@PVA** and 371.4 mg for **Trip-COONa@PVA**.


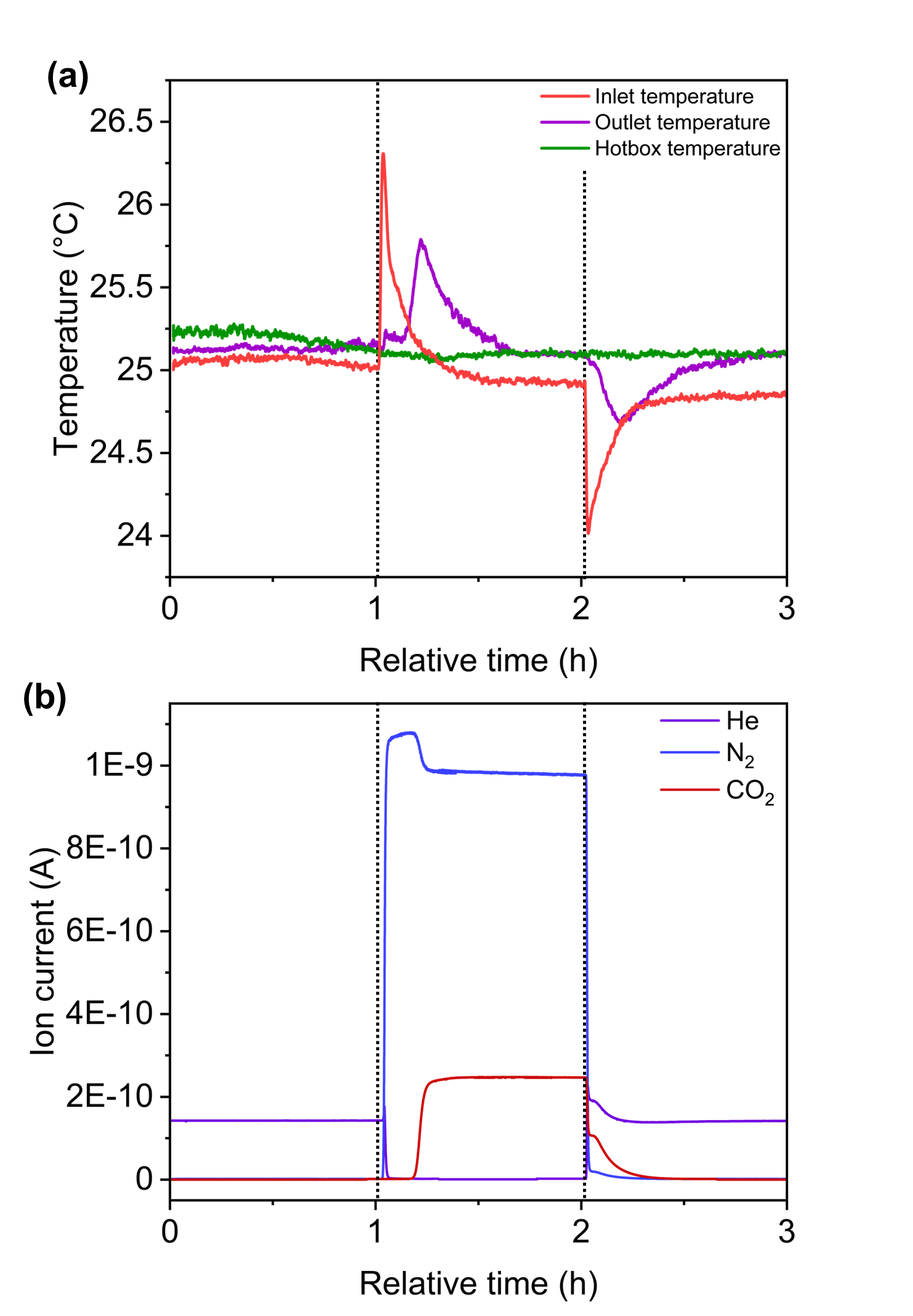


**Figure S1**. Example of the raw data collected from the Micromeritics SSA and the mass spectrometer. (a) Inlet, outlet and heatbox temperatures recorded during a typical breakthrough adsorption/desorption cycle. The dotted vertical lines displayed the time of valve openings: from 0 to 1.02 h Helium (6 sccm) flowed along the column, at 1.02 h the 15:85 CO_2_/N_2_ mixture (6 sccm total) was injected into the system, while stopping the He flow; then, at 2.02 h the He flow (6sccm) was switched on, while halting the CO_2_/N_2_ mixture, to desorb the CO_2_ and regenerate the material. The temperature changes of the porous polymer due to adsorption/desorption processes at the top of the column (inlet temperature) and at the bottom of the column (outlet temperature) were displayed along with the temperature of the hotbox, which contained the breakthrough apparatus. (b) Mass spectrometer signals are registered for He, N_2_, and CO_2_ as a function of time. The dotted vertical lines displayed the time of valve openings.

- **Optimization of the reaction conditions** - **Spbf-COOH-x**

**Gas adsorption measurements**

**Table S2**. Textural properties were measured and calculated from cryogenic N_2_ sorption (77 K) and CO_2_ sorption isotherms collected at 273 K, 283 K, 293 K and 298 K for samples **Spbf-COOH-x**.

| Sample | Langmuir surface area (m^2^/g)^1^ | BET surface area (m^2^/g)^1^ | Pore volume (cm^3^/g)^2^ | Micropore volume (cm^3^/g)^2^ | CO_2_ uptake at 1 bar, 273 K (mmol/g) | CO_2_ uptake at 1 bar, 298 K (mmol/g) | Q_st_ at 0.1 mmol/g (kJ/mol) ^3^ |
| --- | --- | --- | --- | --- | --- | --- | --- |
| Spbf-COOH-1 | 575 | 529 | 0.27 | 0.19 | 2.27 | - | - |
| Spbf-COOH-2 | 776 | 714 | 0.32 | 0.26 | 2.77 | 1.68 | 30.9 |
| Spbf-COOH-3 | 813 | 749 | 0.37 | 0.27 | 2.80 | 1.65 | 31.0 |
| Spbf-COOH-4 | 804 | 741 | 0.31 | 0.28 | 3.08 | 1.79 | 31.1 |
| Spbf-COOH | 785 | 726 | 0.31 | 0.27 | 3.24 | 1.93 | 31.4 |
| Spbf-COOH-5 | 643 | 594 | 0.25 | 0.22 | 3.02 | 1.89 | 31.2 |
| Spbf-COOH-6 | 427 | 395 | 0.15 | 0.14 | 2.50 | 1.52 | 33.0 |

^1^ BET surface areas were calculated in the range 0.015<*p/p_0_*<0.06 according to the Rouquerol analysis. Langmuir surface areas were calculated in the range 0.015<*p/p_0_*<0.06. ^2^ Total and micropore volumes were calculated according to NLDFT theory and HS-2D-NLDFT Carbon, N_2_, 77 K pore model. Micropore volume was evaluated in the pore size range between 0 Å and 20 Å. ^3^ The isosteric heats of adsorption were calculated using the virial method, using the CO_2_ sorption isotherms collected at 273 K, 283 K, 293 K and 298 K.

The N_2_ adsorption isotherm at 77 K and CO_2_ adsorption isotherms at 273 K, 283 K, 293 K and 298 K of the **Spbf-COOH-x** frameworks were reported below.


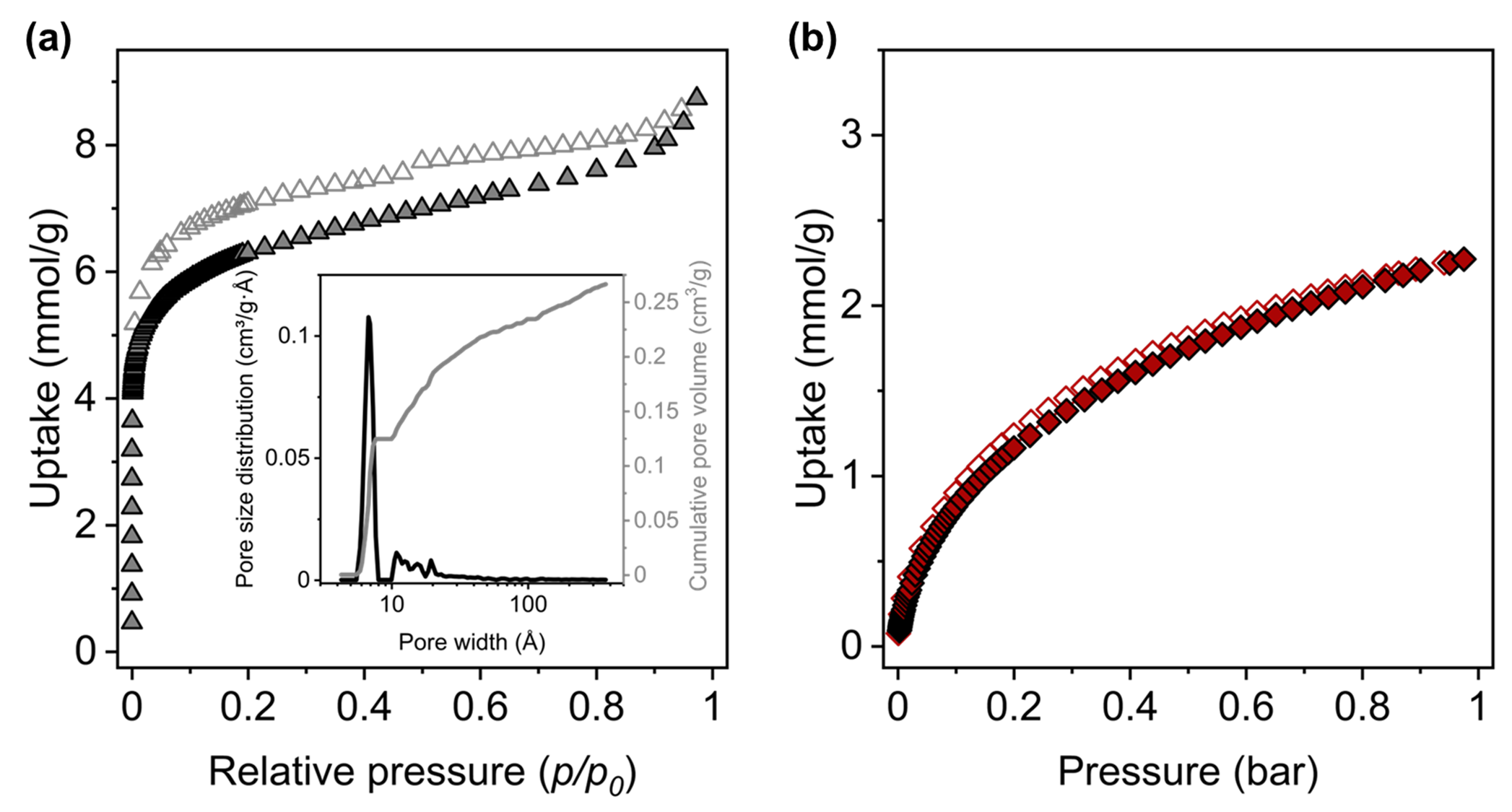


**Figure S2**. (a) N_2_ adsorption isotherm collected at 77 K of **Spbf-COOH-1**. Filled and empty symbols represent sorption and desorption branches, respectively. Inset: differential pore size distribution (black) and cumulative pore size distribution (grey) calculated from N_2_ adsorption isotherm at 77 K according to NLDFT theory and HS-2D-NLDFT, Carbon, N_2_, 77 K pore model. (b) CO_2_ adsorption isotherm collected at 273 K of **Spbf-COOH-1**. Filled and empty symbols represent sorption and desorption branches, respectively.


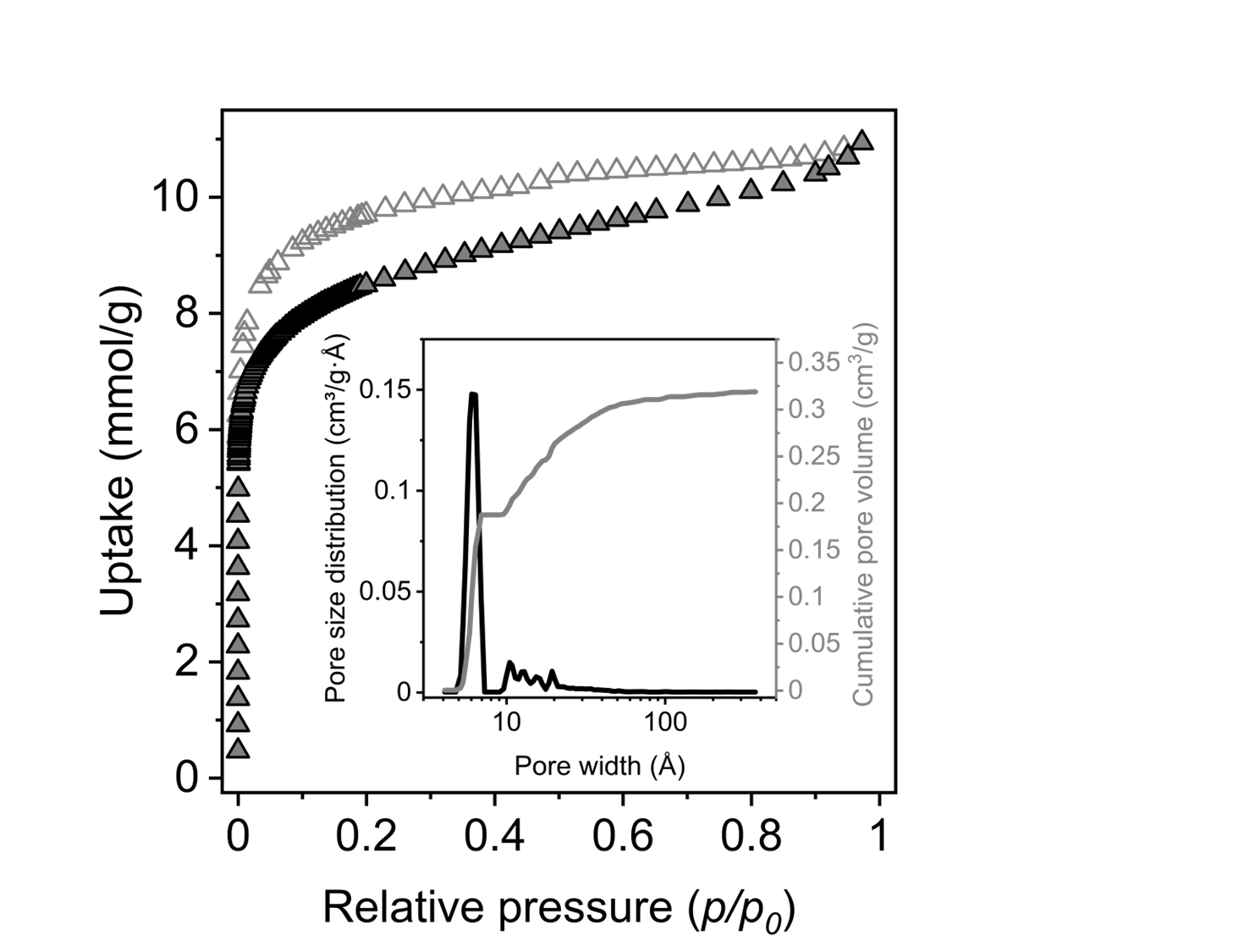


**Figure S3**. N_2_ adsorption isotherm collected at 77 K of **Spbf-COOH-2**. Filled and empty symbols represent sorption and desorption branches, respectively. Inset: differential pore size distribution (black) and cumulative pore size distribution (grey) calculated from N_2_ adsorption isotherm at 77 K according to NLDFT theory and HS-2D-NLDFT, Carbon, N_2_, 77 K pore model.


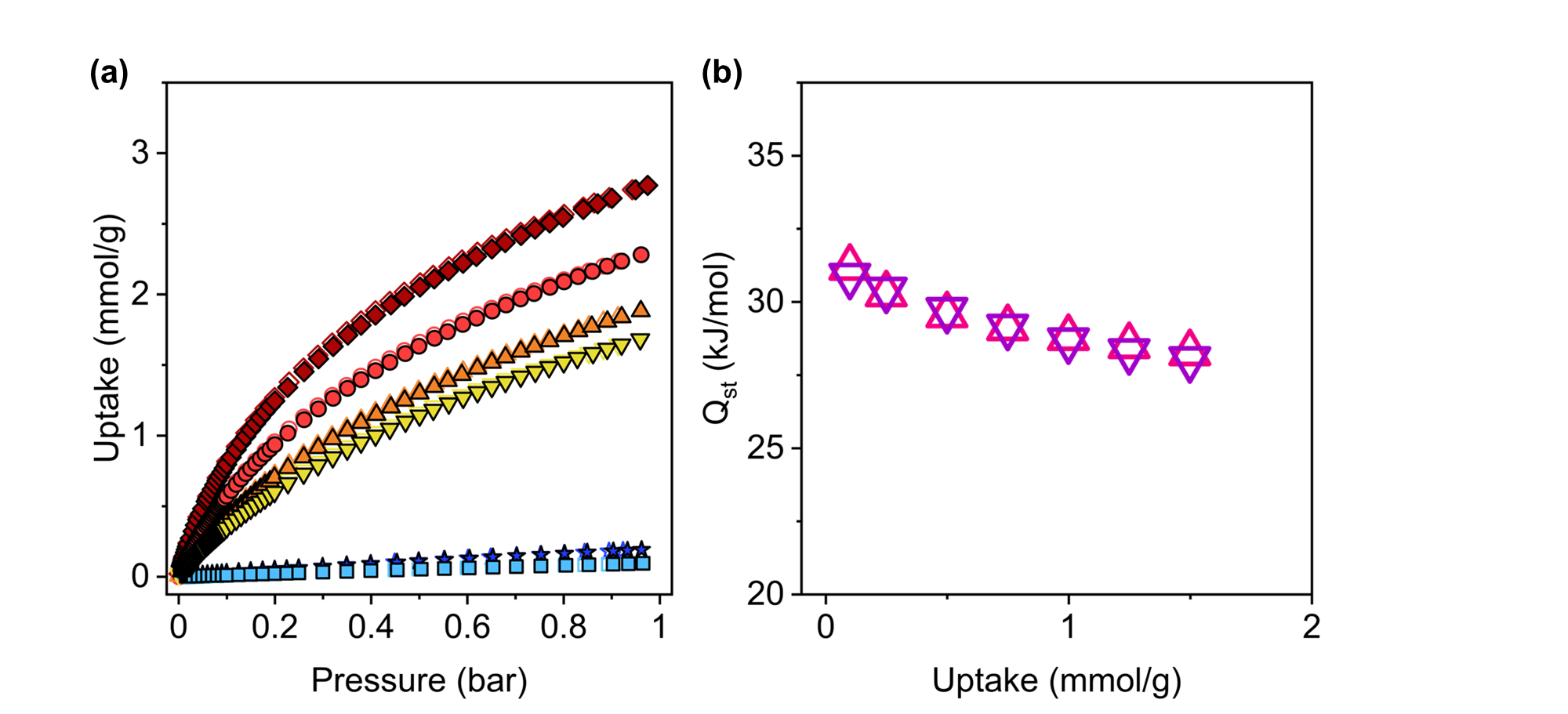


**Figure S4**. (a) CO_2_ adsorption isotherms of **Spbf-COOH-2** collected at 273 K (diamonds, dark red), 283 K (circles, red), 293 K (up-pointing triangles, orange) and 298 K (down-pointing triangles, yellow), and N_2_ adsorption isotherms collected at 273 K (stars, blue) and 298 K (squares, light-blue). Filled and empty symbols represent sorption and desorption branches, respectively. (b) Isosteric heat of adsorption for CO_2_ calculated from the isotherms collected at different temperatures using the virial method (down-pointing triangles, violet) and the Van’t Hoff method after fitting with the Langmuir-Freundlich model (up-pointing triangles, purple).


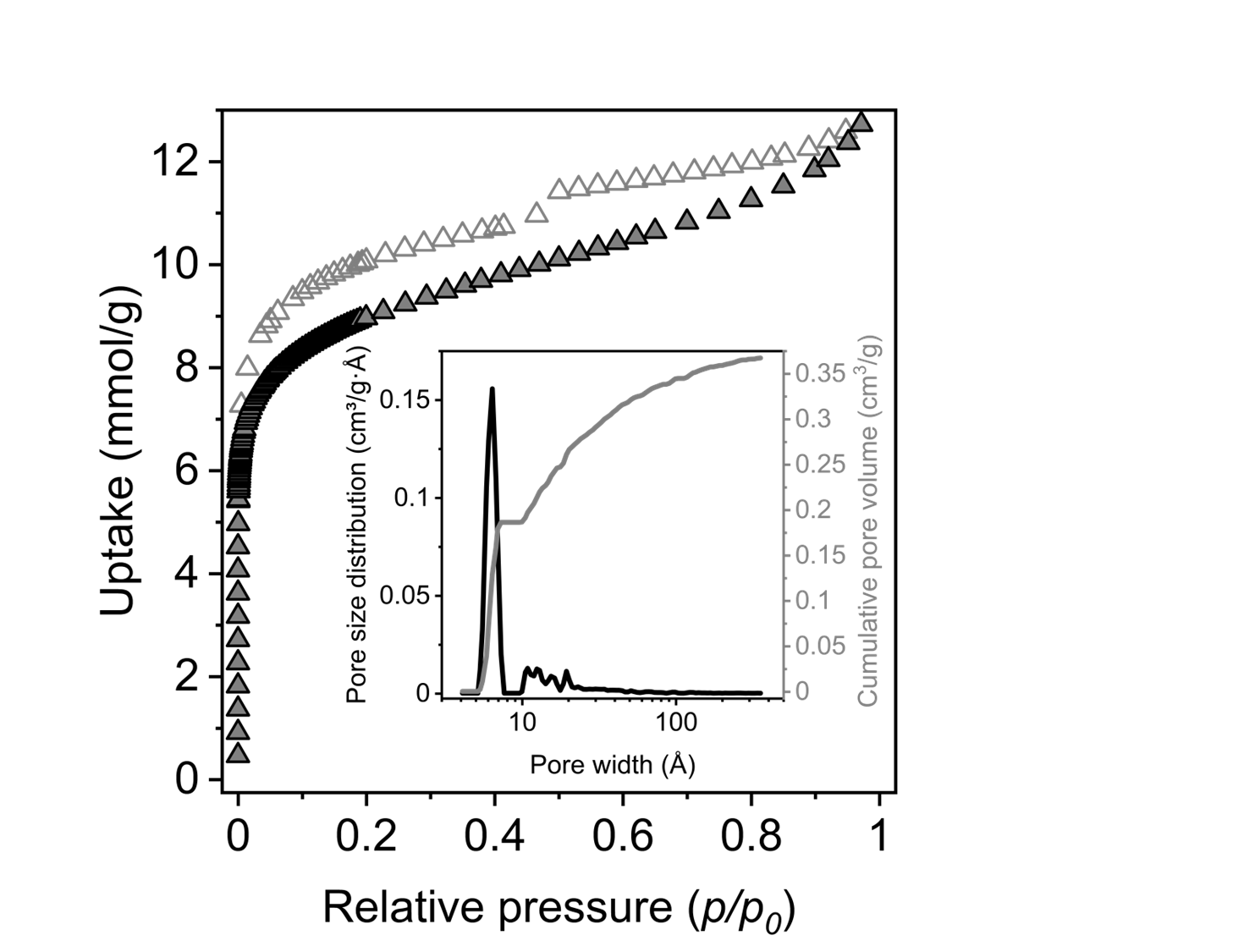


**Figure S5**. N_2_ adsorption isotherm collected at 77 K of **Spbf-COOH-3**. Filled and empty symbols represent sorption and desorption branches, respectively. Inset: differential pore size distribution (black) and cumulative pore size distribution (grey) calculated from N_2_ adsorption isotherm at 77 K according to NLDFT theory and HS-2D-NLDFT, Carbon, N_2_, 77 K pore model.


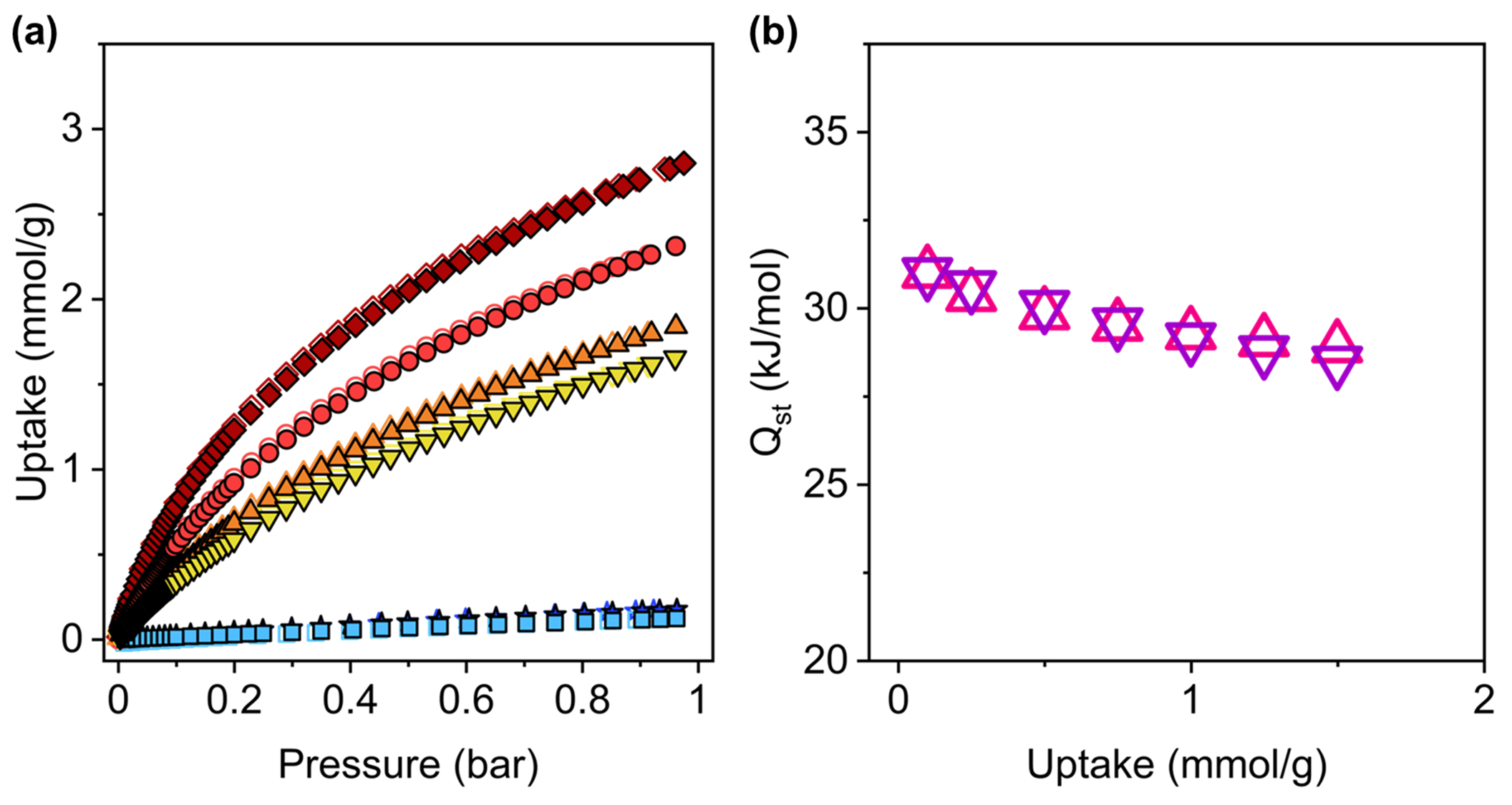


**Figure S6**. (a) CO_2_ adsorption isotherms of **Spbf-COOH-3** collected at 273 K (diamonds, dark red), 283 K (circles, red), 293 K (up-pointing triangles, orange) and 298 K (down-pointing triangles, yellow), and N_2_ adsorption isotherms collected at 273 K (stars, blue) and 298 K (squares, light-blue). Filled and empty symbols represent sorption and desorption branches, respectively. (b) Isosteric heat of adsorption for CO_2_ calculated from the isotherms collected at different temperatures using the virial method (down-pointing triangles, violet) and the Van’t Hoff method after fitting with the Langmuir-Freundlich model (up-pointing triangles, purple).


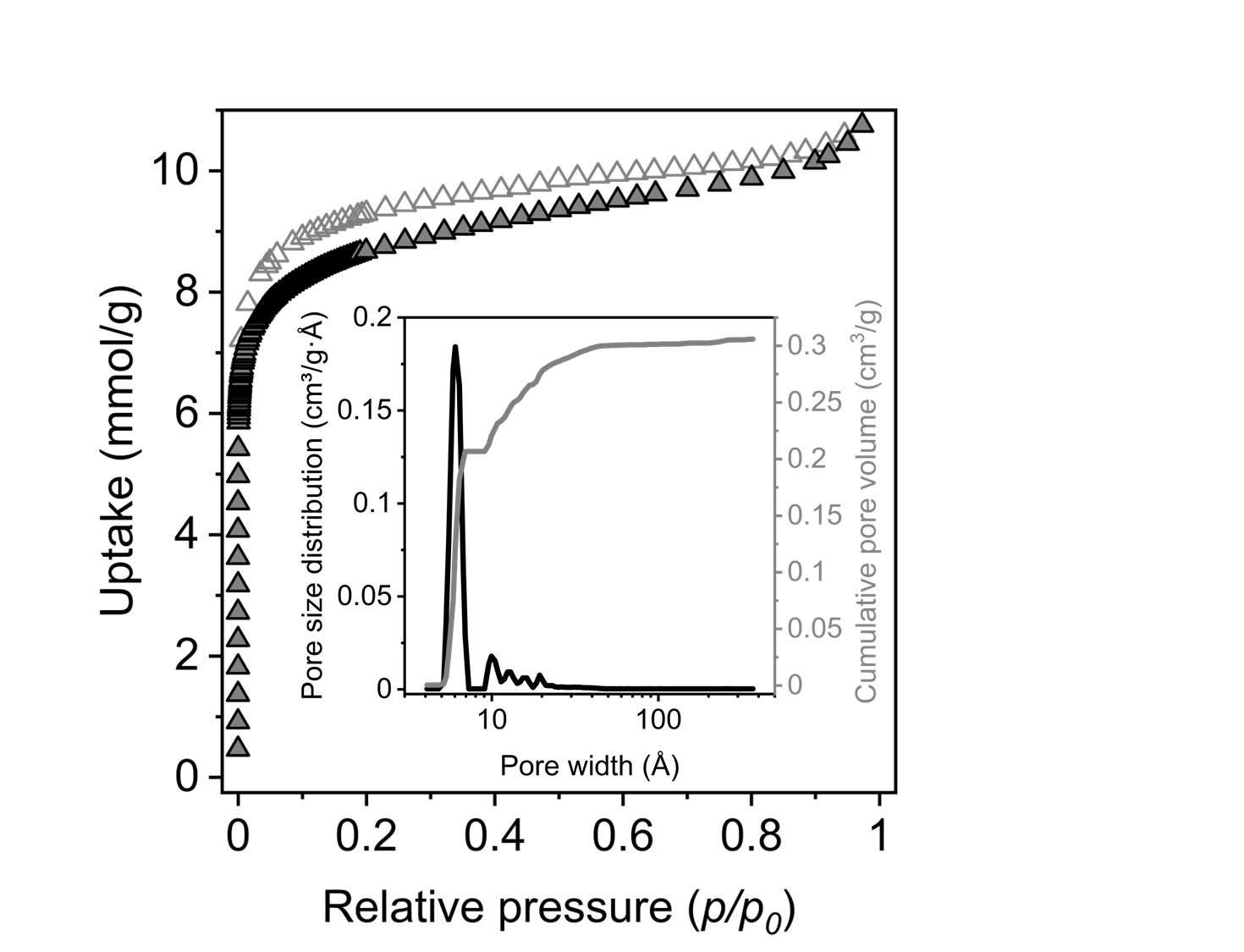


**Figure S7**. N_2_ adsorption isotherm collected at 77 K of **Spbf-COOH-4**. Filled and empty symbols represent sorption and desorption branches, respectively. Inset: differential pore size distribution (black) and cumulative pore size distribution (grey) calculated from N_2_ adsorption isotherm at 77 K according to NLDFT theory and HS-2D-NLDFT, Carbon, N_2_, 77 K pore model.


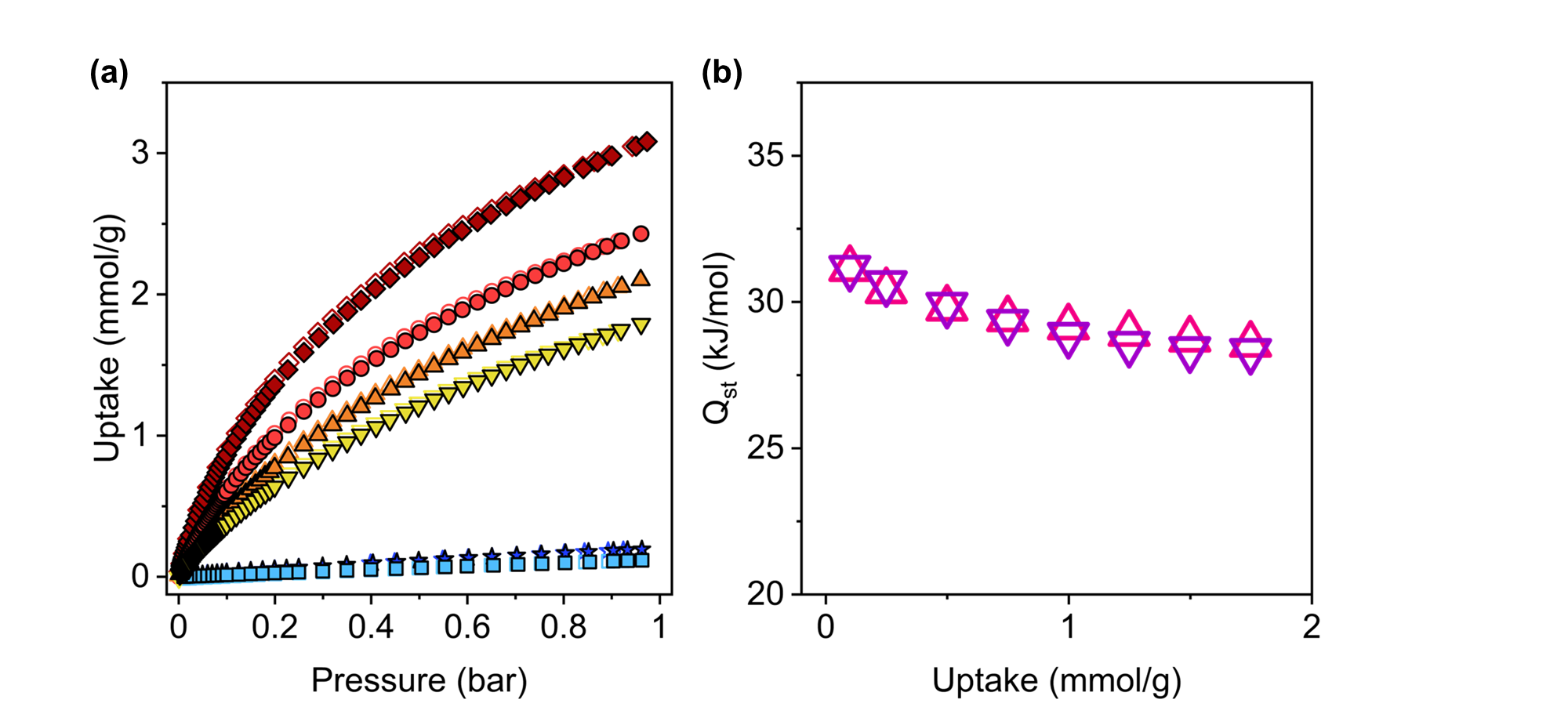


**Figure S8**. (a) CO_2_ adsorption isotherms of **Spbf-COOH-4** collected at 273 K (diamonds, dark red), 283 K (circles, red), 293 K (up-pointing triangles, orange) and 298 K (down-pointing triangles, yellow), and N_2_ adsorption isotherms collected at 273 K (stars, blue) and 298 K (squares, light-blue). Filled and empty symbols represent sorption and desorption branches, respectively. (b) Isosteric heat of adsorption for CO_2_ calculated from the isotherms collected at different temperatures using the virial method (down-pointing triangles, violet) and the Van’t Hoff method after fitting with the Langmuir-Freundlich model (up-pointing triangles, purple).


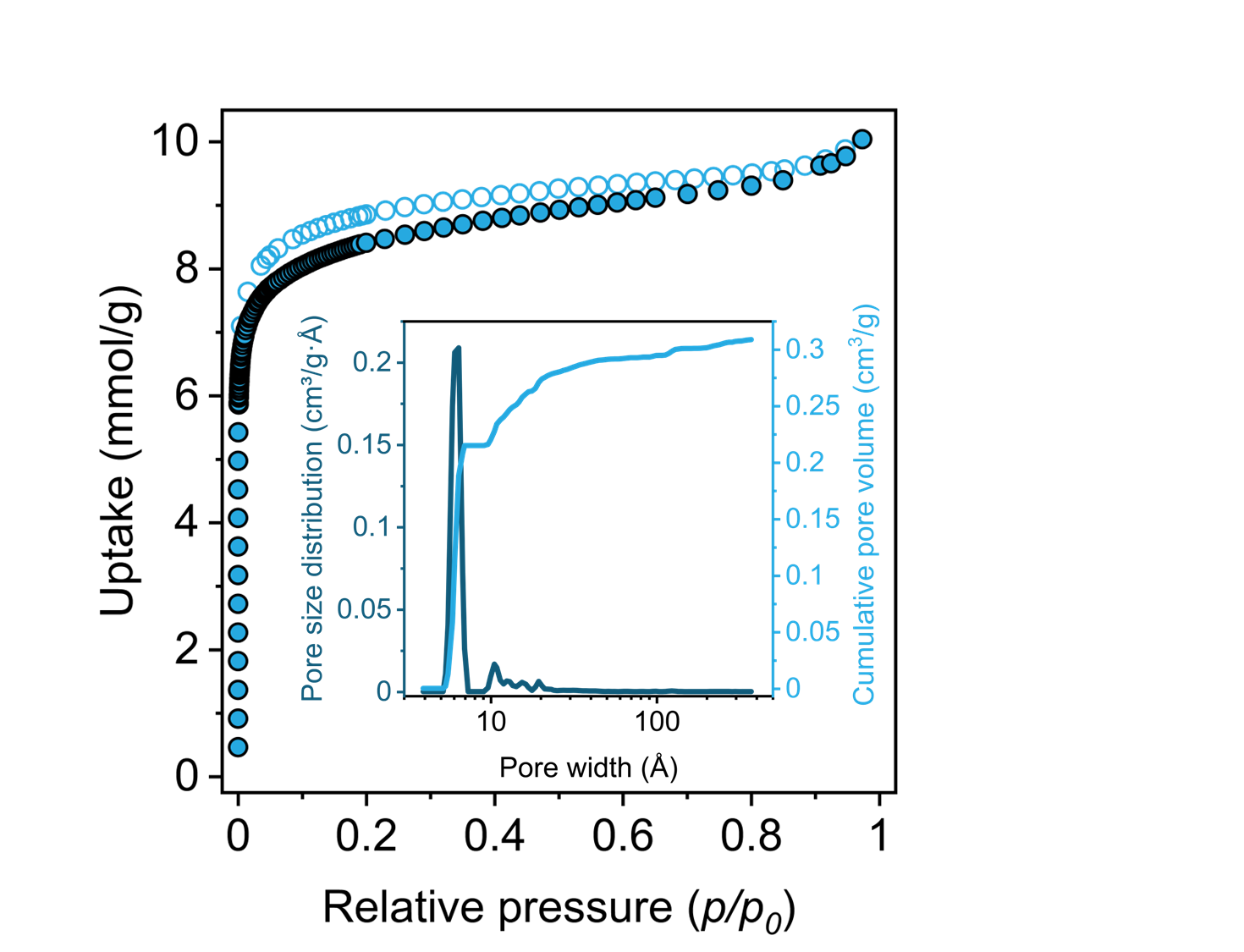


**Figure S9**. N_2_ adsorption isotherm collected at 77 K of **Spbf-COOH**. Filled and empty symbols represent sorption and desorption branches, respectively. Inset: differential pore size distribution (blue) and cumulative pore size distribution (light blue) calculated from N_2_ adsorption isotherm at 77 K according to NLDFT theory and HS-2D-NLDFT, Carbon, N_2_, 77 K pore model.


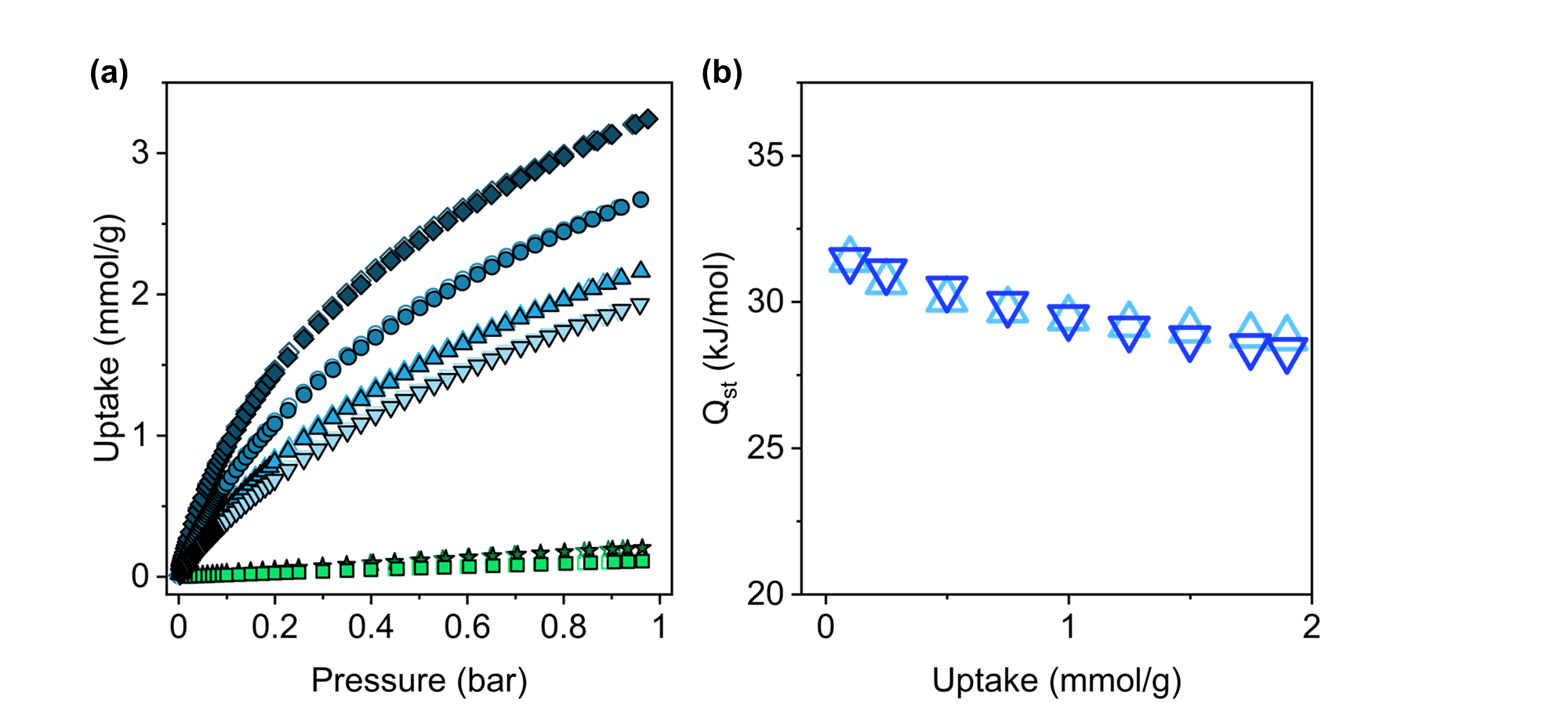


**Figure S10**. (a) CO_2_ adsorption isotherms of sample **Spbf-COOH** collected at 273 K (diamonds, dark blue), 283 K (circles, blue), 293 K (up-pointing triangles, light-blue) and 298 K (down-pointing triangles, lighter blue), and N_2_ adsorption isotherms collected at 273 K (stars, dark green) and 298 K (squares, light green). Filled and empty symbols represent sorption and desorption branches, respectively. (b) Isosteric heat of adsorption for CO_2_ calculated from the isotherms collected at different temperatures using the virial method (down-pointing triangles, blue) and the Van’t Hoff method after fitting with the Langmuir-Freundlich model (up-pointing triangles, light-blue).


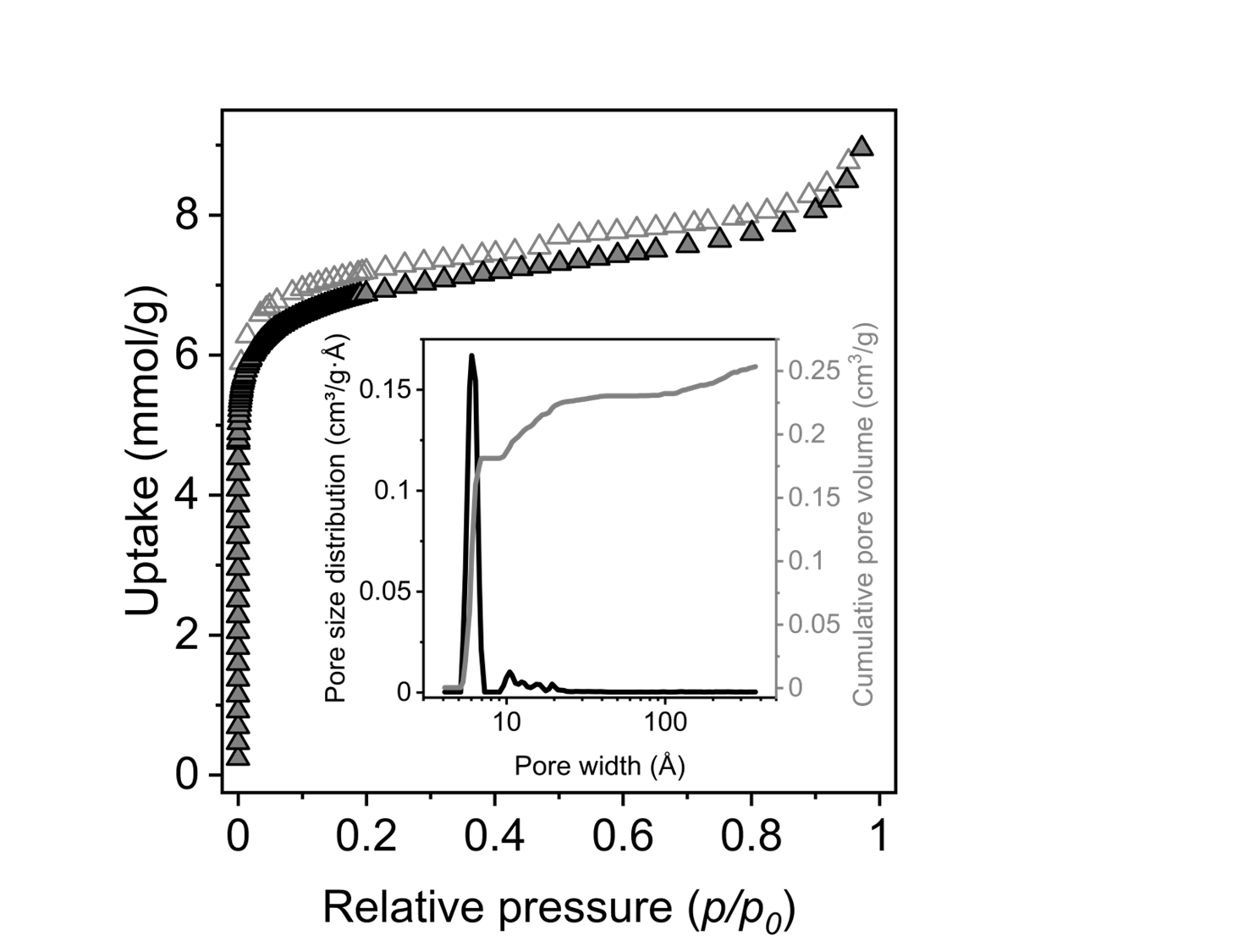


**Figure S11**. N_2_ adsorption isotherm collected at 77 K of **Spbf-COOH-5**. Filled and empty symbols represent sorption and desorption branches, respectively. Inset: differential pore size distribution (black) and cumulative pore size distribution (grey) calculated from N_2_ adsorption isotherm at 77 K according to NLDFT theory and HS-2D-NLDFT, Carbon, N_2_, 77 K pore model.


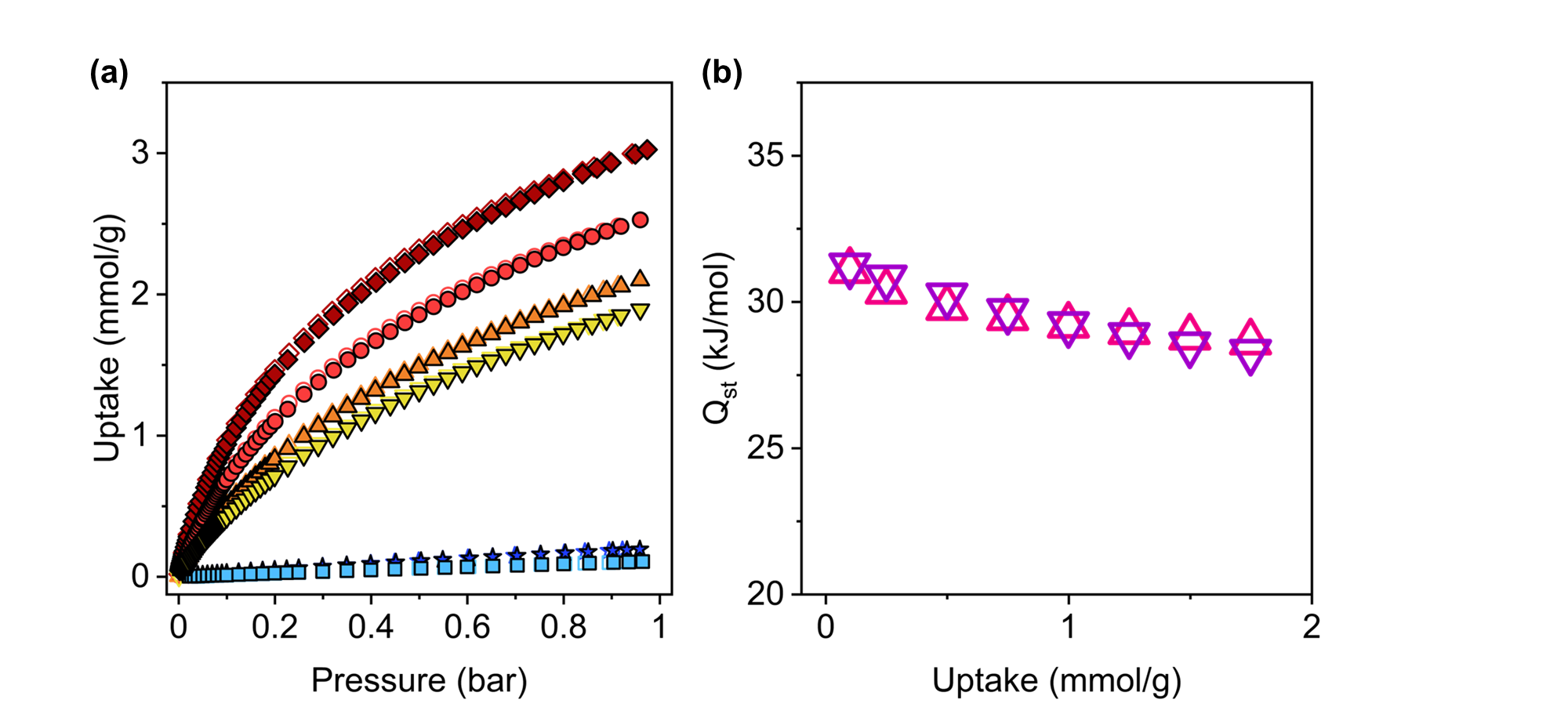


**Figure S12**. (a) CO_2_ adsorption isotherms of **Spbf-COOH-5** collected at 273 K (diamonds, dark red), 283 K (circles, red), 293 K (up-pointing triangles, orange) and 298 K (down-pointing triangles, yellow), and N_2_ adsorption isotherms collected at 273 K (stars, blue) and 298 K (squares, light-blue). Filled and empty symbols represent sorption and desorption branches, respectively. (b) Isosteric heat of adsorption for CO_2_ calculated from the isotherms collected at different temperatures using the virial method (down-pointing triangles, violet) and the Van’t Hoff method after fitting with the Langmuir-Freundlich model (up-pointing triangles, purple).


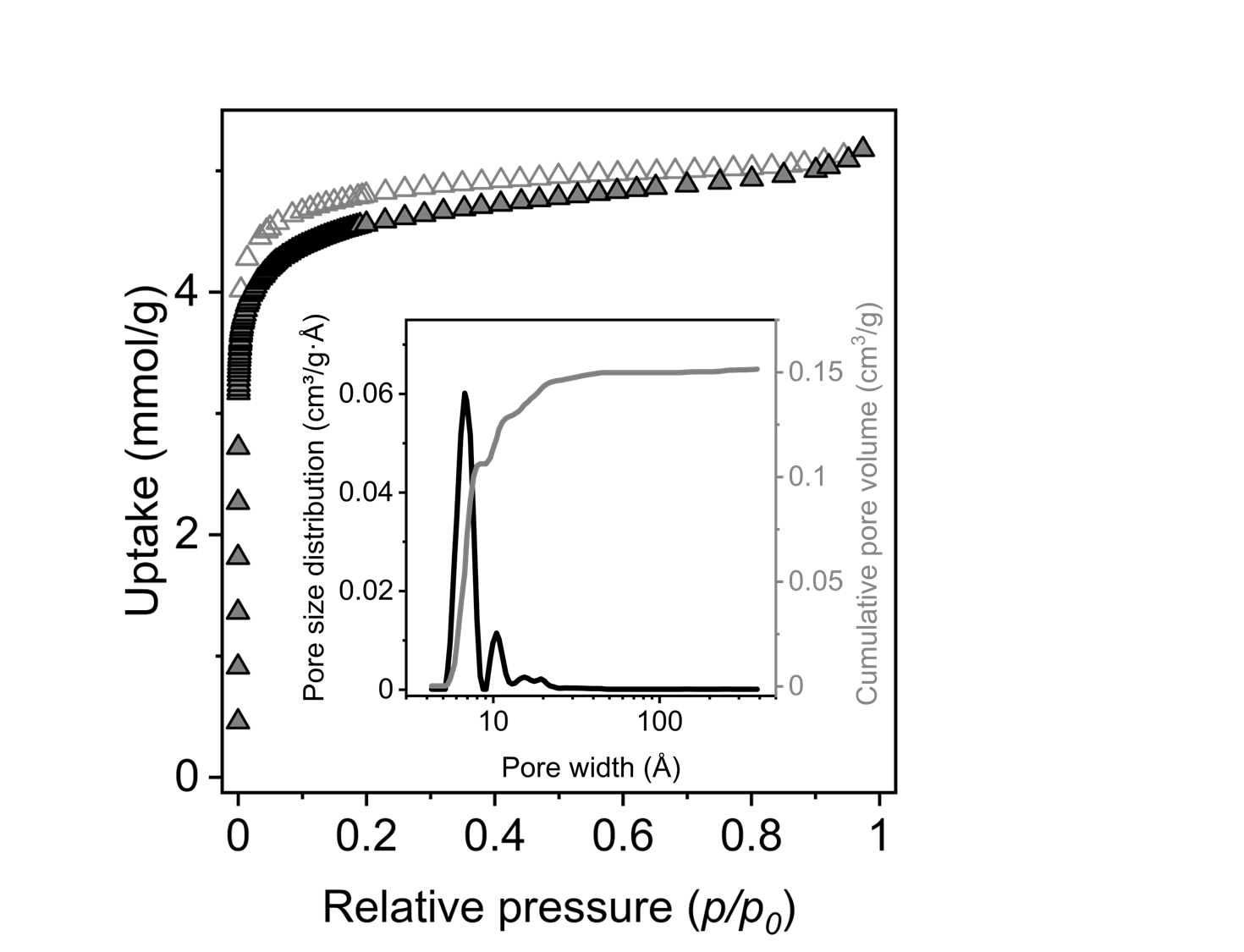


**Figure S13**. N_2_ adsorption isotherm collected at 77 K of sample **Spbf-COOH-6.** Filled and empty symbols represent sorption and desorption branches, respectively. Inset: differential pore size distribution (black) and cumulative pore size distribution (grey) calculated from N_2_ adsorption isotherm at 77 K according to NLDFT theory and HS-2D-NLDFT, Carbon, N_2_, 77 K pore model.


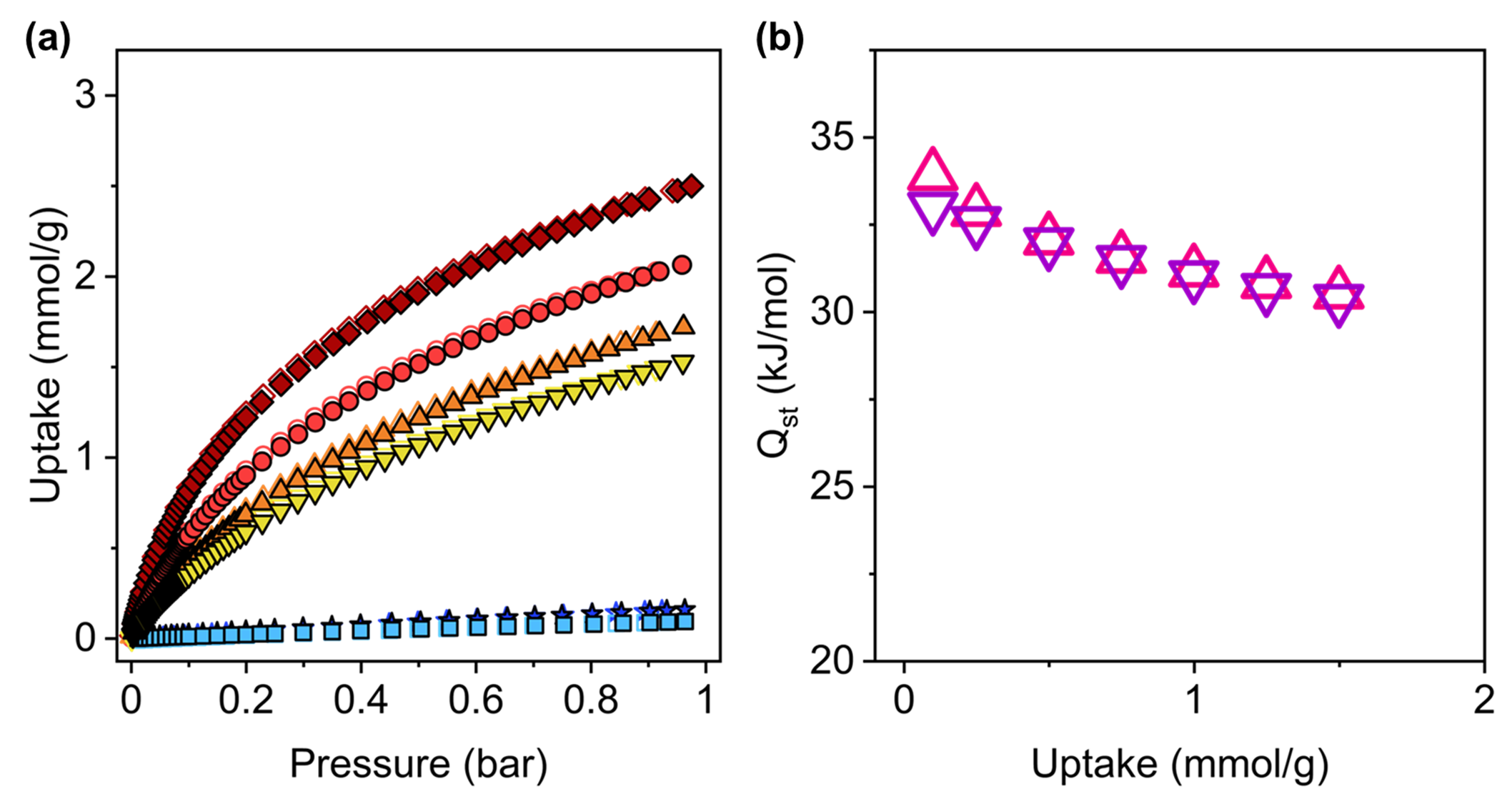


**Figure S14**. (a) CO_2_ adsorption isotherms of **Spbf-COOH-6** collected at 273 K (diamonds, dark red), 283 K (circles, red), 293 K (up-pointing triangles, orange) and 298 K (down-pointing triangles, yellow), and N_2_ adsorption isotherms collected at 273 K (stars, blue) and 298 K (squares, light-blue). Filled and empty symbols represent sorption and desorption branches, respectively. **(b)** Isosteric heat of adsorption for CO_2_ calculated from the isotherms collected at different temperatures using the virial method (down-pointing triangles, violet) and the Van’t Hoff method after fitting with the Langmuir-Freundlich model (up-pointing triangles, purple).

**Infrared spectroscopy**


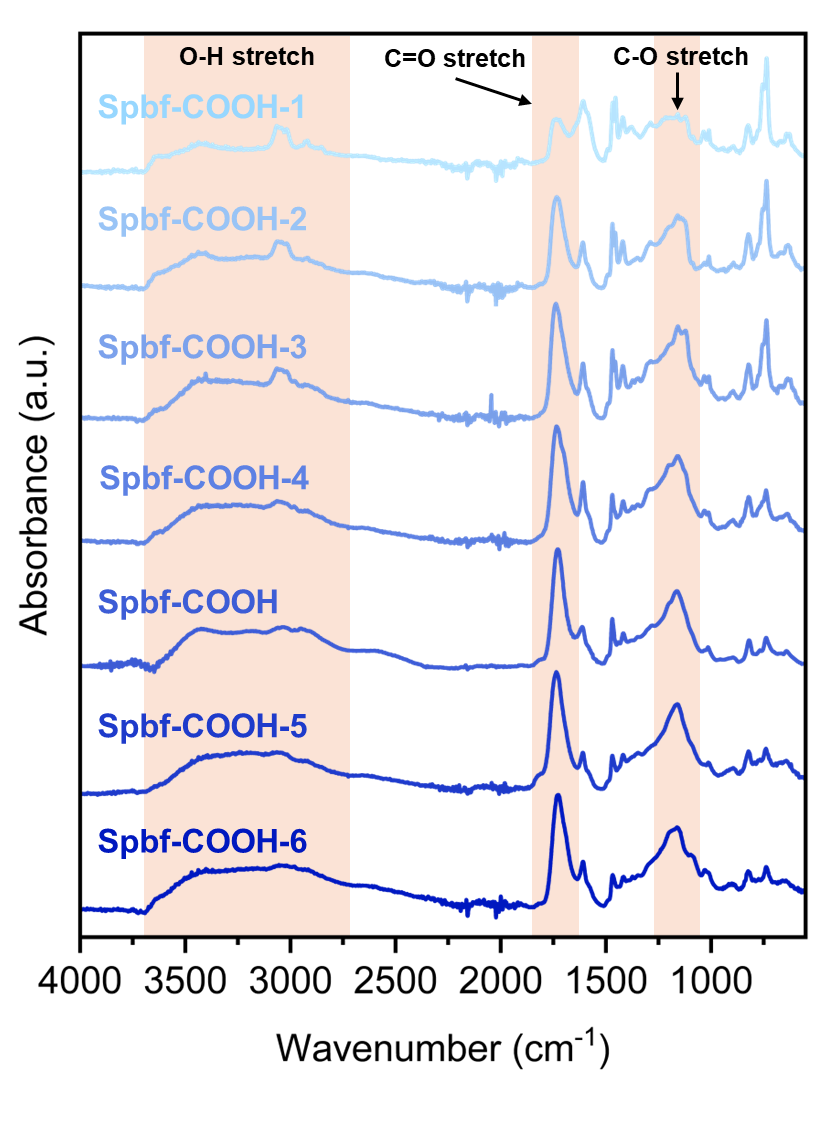


**Figure S15**. FT-IR spectra of **Spbf-COOH-x** collected from 525 cm^-1^ to 4000 cm^-1^. The characteristic vibrational bands associated with the carboxylic acid functional groups are highlighted in the figures.

**Thermal analysis**


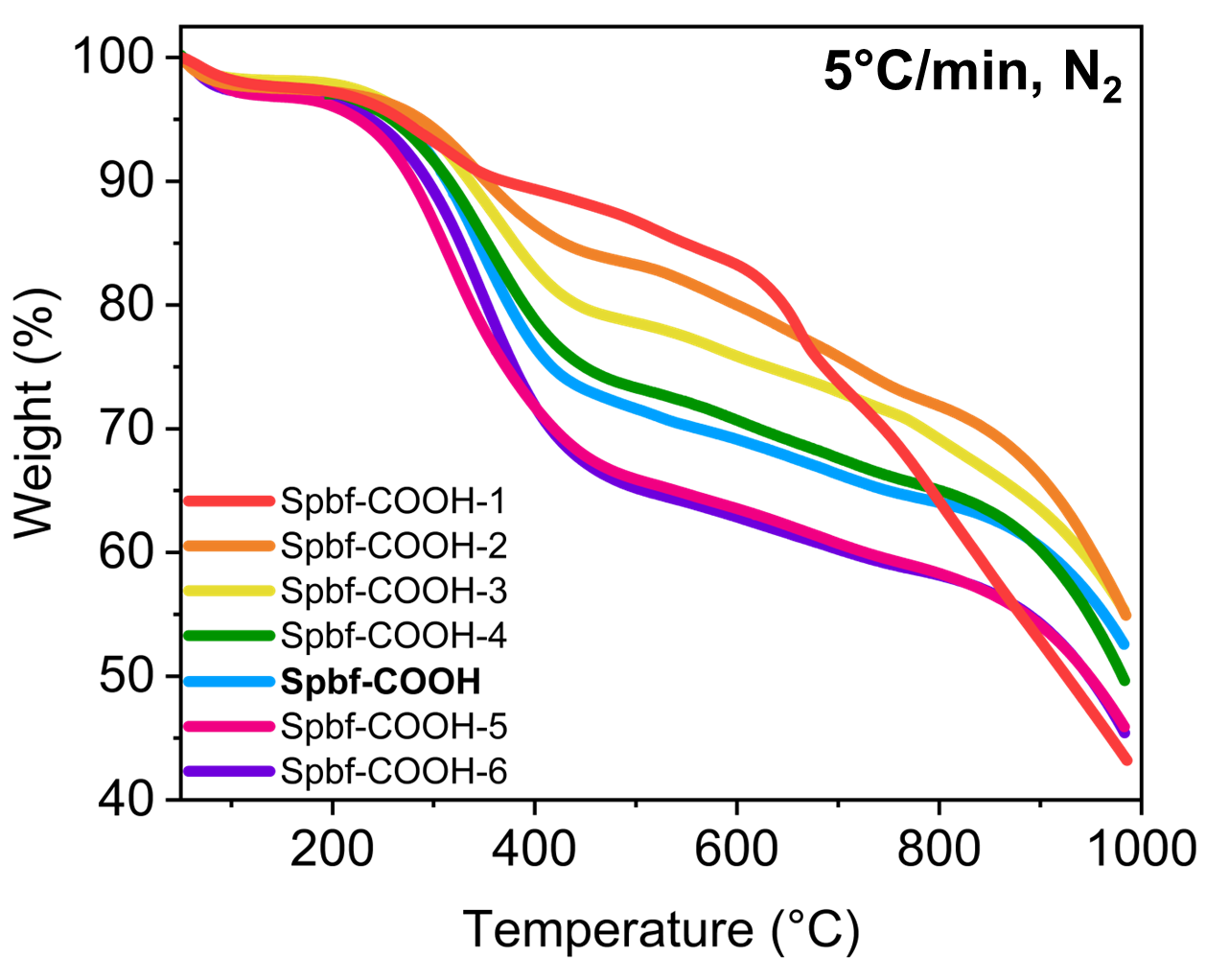


**Figure S16**. TGA analysis of the samples **Spbf-COOH-x** prepared with different cross-linker to monomer ratio collected under inert atmosphere (N_2_ flow, 50 mL/min) with a heating rate of 5°C/min between 50°C and 1000°C.


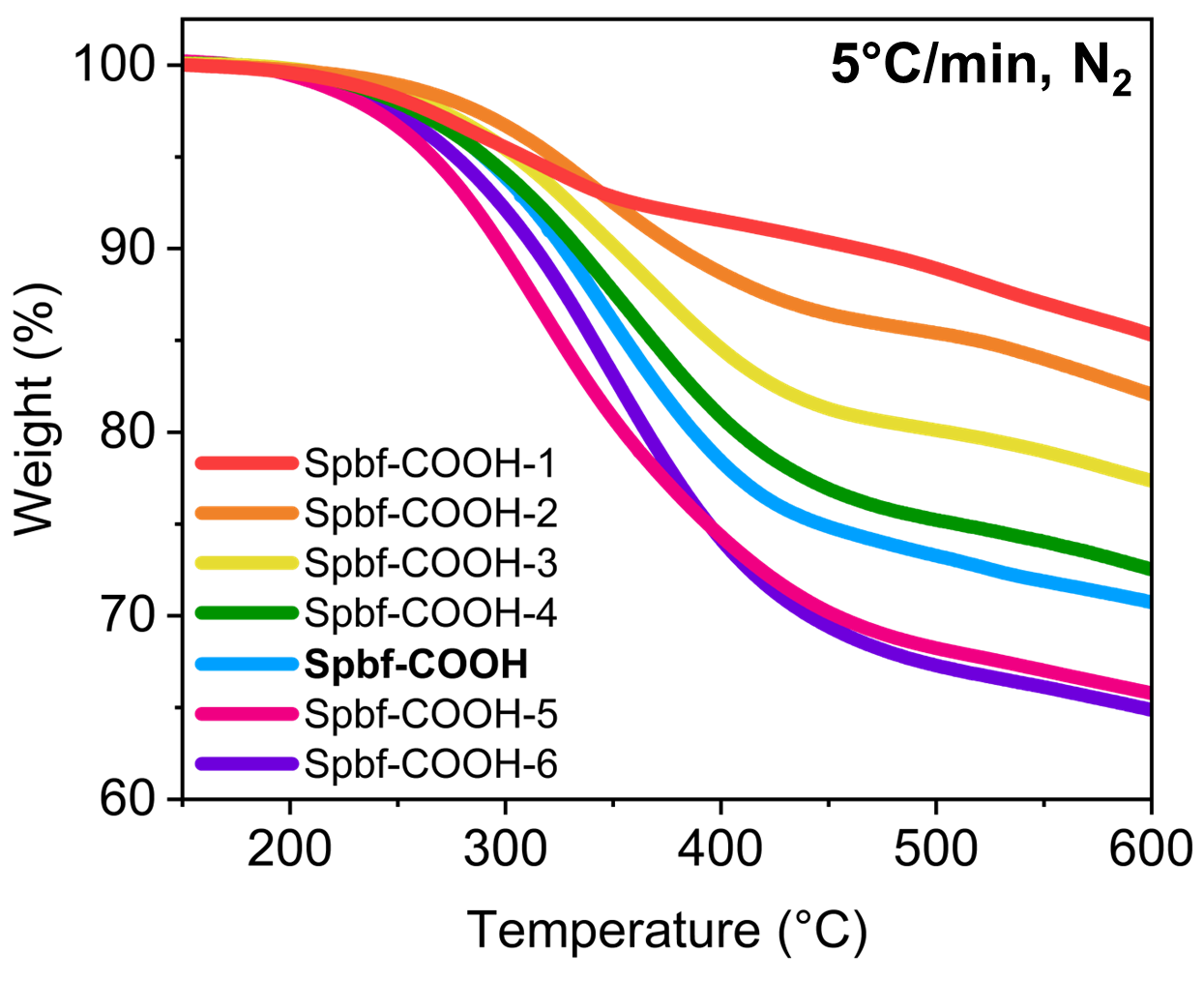


**Figure S17**. TGA analysis of the samples **Spbf-COOH-x** prepared with different cross-linker to monomer ratios collected under an inert atmosphere (N_2_ flow, 50 mL/min) with a heating rate of 5°C/min between 50°C and 600°C. The weight loss between 200°C and 500°C was ascribed to the decarboxylation reaction of the frameworks, as proved for **Spbf-COOH** (coupled TGA-FTIR, **figures S30, S31**). These weight losses increased along with the molar fraction of the cross-linker employed in the synthesis, suggesting the formation of higher densities of cross-linkers and carboxylic acid groups.

**Powder X-ray diffraction**


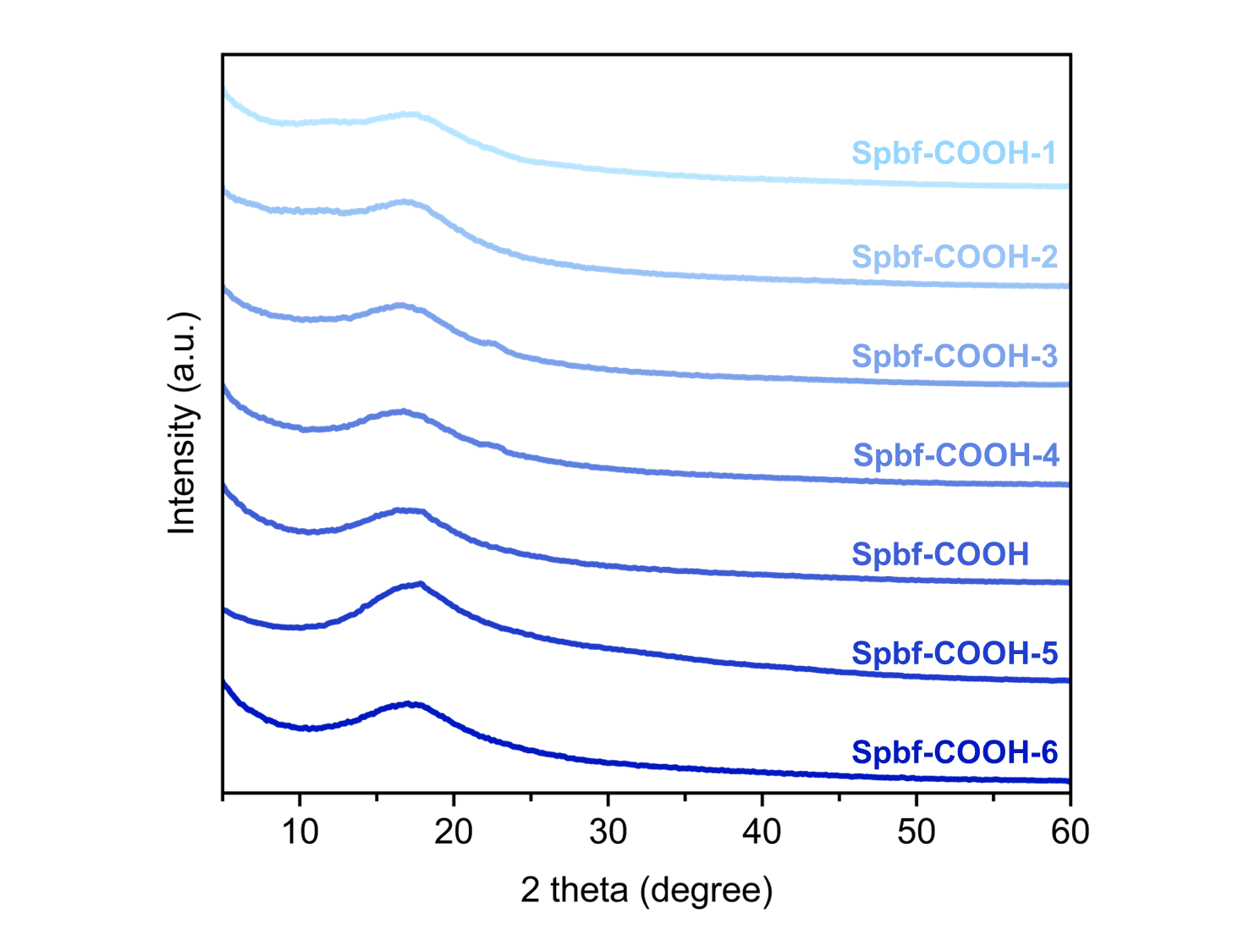


**Figure S18**. Powder X-ray diffraction of samples **Spbf-COOH-x**. From top to bottom (lighter to darker color): **Spbf-COOH-1**, **Spbf-COOH-2**, **Spbf-COOH-3**, **Spbf-COOH-4**, **Spbf-COOH**, **Spbf-COOH-5** and **Spbf-COOH-6**. PXRD patterns displayed a broad peak at ~17.5° 2 theta.

- **Characterization of Spbf-COOH and Trip-COOH**

**Textural properties**

**
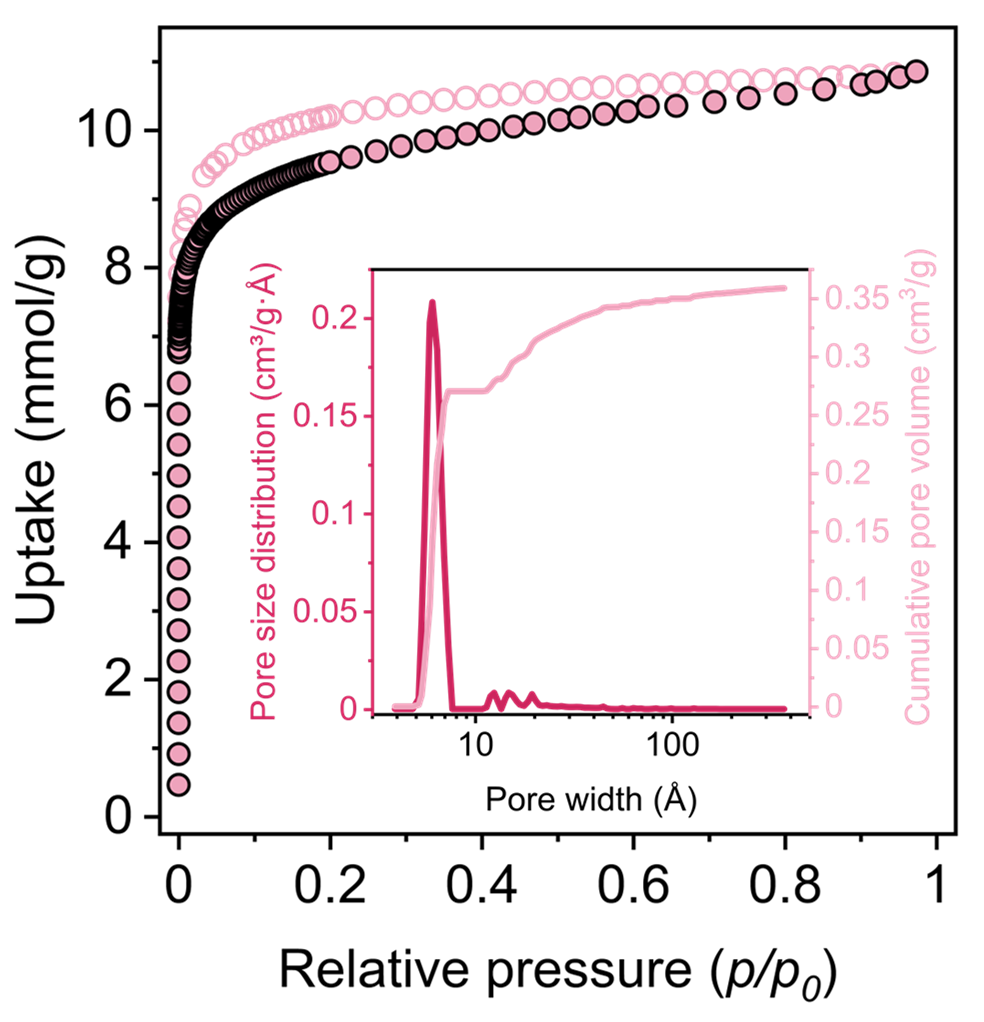
**

**Figure S19**. N_2_ adsorption isotherm collected at 77 K of the sample **Trip-COOH**. Filled and empty symbols represent sorption and desorption branches, respectively. Inset: differential pore size distribution (purple) and cumulative pore size distribution (pink) calculated from N_2_ adsorption isotherm at 77 K according to NLDFT theory and HS-2D-NLDFT, Carbon, N_2_, 77 K pore model.

**Table S3**. Textural properties measured and calculated from cryogenic N_2_ sorption (77 K) and CO_2_ sorption isotherms collected at 273 K, 283 K, 293 K and 298 K for **Trip-COOH**.

| Sample | Langmuir surface area (m^2^/g)^1^ | BET surface area (m^2^/g)^1^ | Pore volume (cm^3^/g)^2^ | Micropore volume (cm^3^/g)^2^ | CO_2_ uptake at 1 bar, 273 K (mmol/g) | CO_2_ uptake at 1 bar, 298 K (mmol/g) | Q_st_ at 0.1 mmol/g (kJ/mol) ^3^ |
| --- | --- | --- | --- | --- | --- | --- | --- |
| Trip-COOH | 890 | 822 | 0.35 | 0.30 | 4.12 | 2.67 | 32.0 |

^1^ BET surface areas were calculated in the range 0.015<*p/p_0_*<0.06 according to the Rouquerol analysis. Langmuir surface areas were calculated in the range 0.015<*p/p_0_*<0.06. ^2^ Total and micropore volumes were calculated according to NLDFT theory and HS-2D-NLDFT Carbon, N_2_, 77 K pore model. Micropore volume was evaluated in the pore size range between 0 Å and 20 Å. ^3^ The isosteric heats of adsorption were calculated using the virial method, using the CO_2_ sorption isotherms collected at 273 K, 283 K, 293 K and 298 K.

**
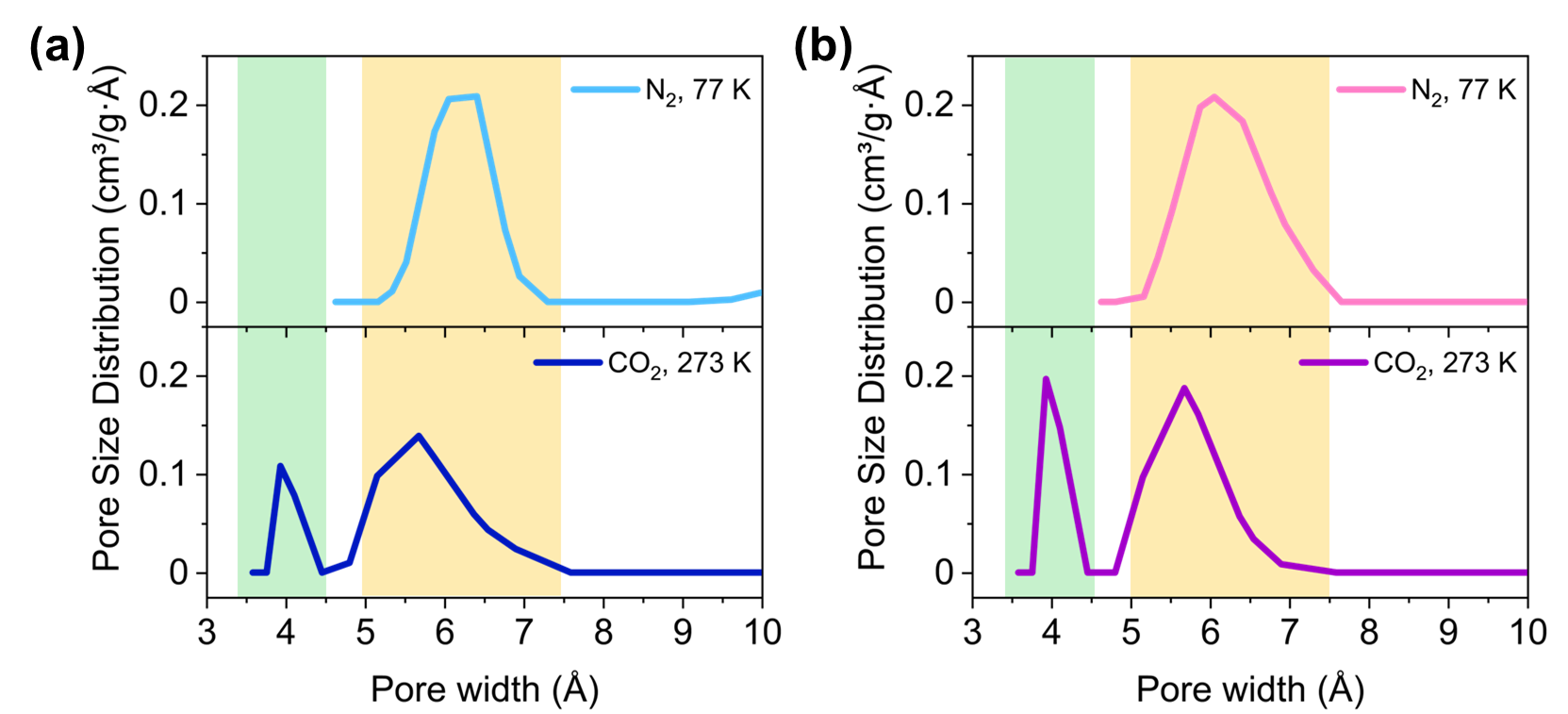
**

**Figure S20.** (a) Top: pore size distribution of **Spbf-COOH** calculated from N_2_ adsorption isotherm at 77 K according to the HS 2D-NLDFT theory and carbon slit pore model for nitrogen adsorption at 77 K (light blue). The PSD displayed a narrow peak in the ultra-microporous region (5 < pore diameter (Å) < 7.5), highlighted by the orange shading. Bottom: pore size distribution of **Spbf-COOH** calculated from CO_2_ adsorption isotherm at 273 K according to the HS 2D-NLDFT theory and carbon slit pore model for CO_2_ adsorption at 273 K (dark blue). The PSD displayed a peak centered at the same value as the one calculated from the N_2_ isotherm at 77 K. Furthermore, it highlights the presence of ultra-micropores with mean sizes between 3.5 Å and 5 Å, which can only be accessed by CO_2_ molecules.(b) Top: pore size distribution of **Trip-COOH** calculated from N_2_ adsorption isotherm at 77 K according to the HS 2D-NLDFT theory and carbon slit pore model for nitrogen adsorption at 77 K (pink). The PSD displayed a narrow peak in the ultra-microporous region (5 < pore diameter (Å) < 7.5), highlighted by the orange shading. Bottom: pore size distribution of **Trip-COOH** calculated from CO_2_ adsorption isotherm at 273 K according to the HS 2D-NLDFT theory and carbon slit pore model for CO_2_ adsorption at 273 K (purple). The PSD displayed a peak centered at the same value as the one calculated from the N_2_ isotherm at 77 K. Furthermore, it highlights the presence of ultra-micropores with mean sizes between 3.5 Å and 5 Å, which can only be accessed by CO_2_ molecules.


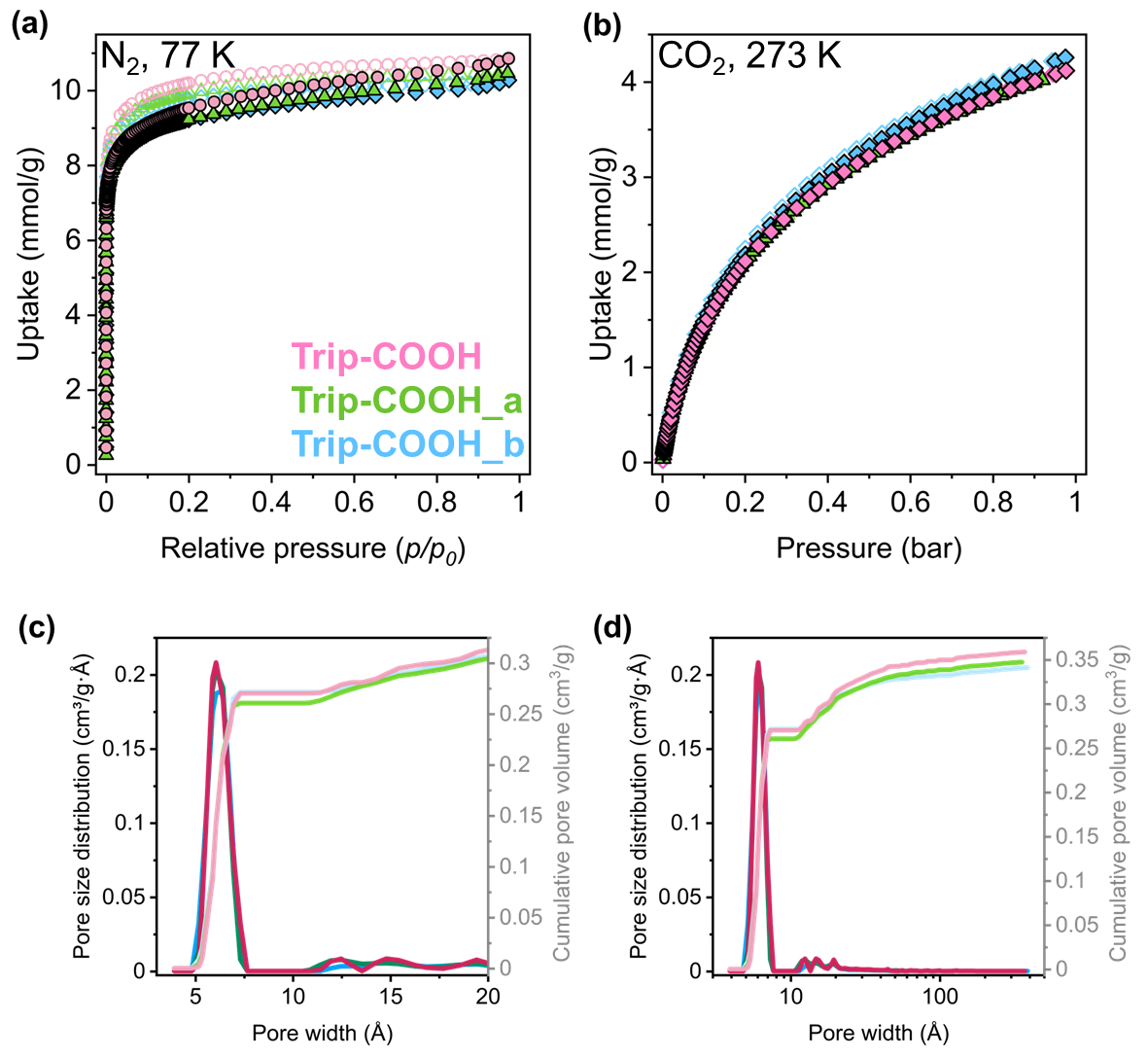


**Figure S21**. **Reproducibility of the textural properties of different batches.** The gram-scale synthesis of **Trip-COOH** was repeated three times to produce **Trip-COOH**, **Trip-COOH_a** and **Trip-COOH_b**. (a) N_2_ adsorption isotherms collected at 77 K for **Trip-COOH** (pink circles), **Trip-COOH_a** (green triangles) and **Trip-COOH_b** (light blue diamonds). (b) CO_2_ adsorption isotherms collected at 273 K for **Trip-COOH** (pink circles), **Trip-COOH_a** (green triangles) and **Trip-COOH_b** (light blue diamonds). Filled and empty symbols represent sorption and desorption branches, respectively. (c) and (d) Differential pore size distribution and cumulative pore size distribution calculated from N_2_ adsorption isotherm at 77 K according to NLDFT theory and HS-2D-NLDFT, Carbon, N_2_, 77 K pore model for sample **Trip-COOH** (pink), **Trip-COOH_a** (green) and **Trip-COOH_b** (light blue).

**Table S4**. Textural properties measured and calculated from cryogenic N_2_ sorption (77 K) and CO_2_ sorption isotherms collected at 273 K for samples **Trip-COOH**, **Trip-COOH_a** and **Trip-COOH_b**.

| Sample | Langmuir surface area (m^2^/g)^1^ | BET surface area (m^2^/g)^1^ | Pore volume (cm^3^/g)^2^ | Micropore volume (cm^3^/g)^2^ | CO_2_ uptake at 1 bar, 273 K (mmol/g) |
| --- | --- | --- | --- | --- | --- |
| Trip-COOH | 890 | 822 | 0.35 | 0.30 | 4.12 |
| Trip-COOH_a | 862 | 798 | 0.35 | 0.30 | 4.03 |
| Trip-COOH_b | 870 | 798 | 0.34 | 0.31 | 4.25 |

^1^ BET surface areas were calculated in the range 0.015<*p/p_0_*<0.06 according to the Rouquerol analysis. Langmuir surface areas were calculated in the range 0.015<*p/p_0_*<0.06. ^2^ Total and micropore volumes were calculated according to NLDFT theory and HS-2D-NLDFT Carbon, N_2_, 77 K pore model. Micropore volume was evaluated in the pore size range between 0 Å and 20 Å.

**^13^C and ^1^H solid-state NMR spectroscopy**


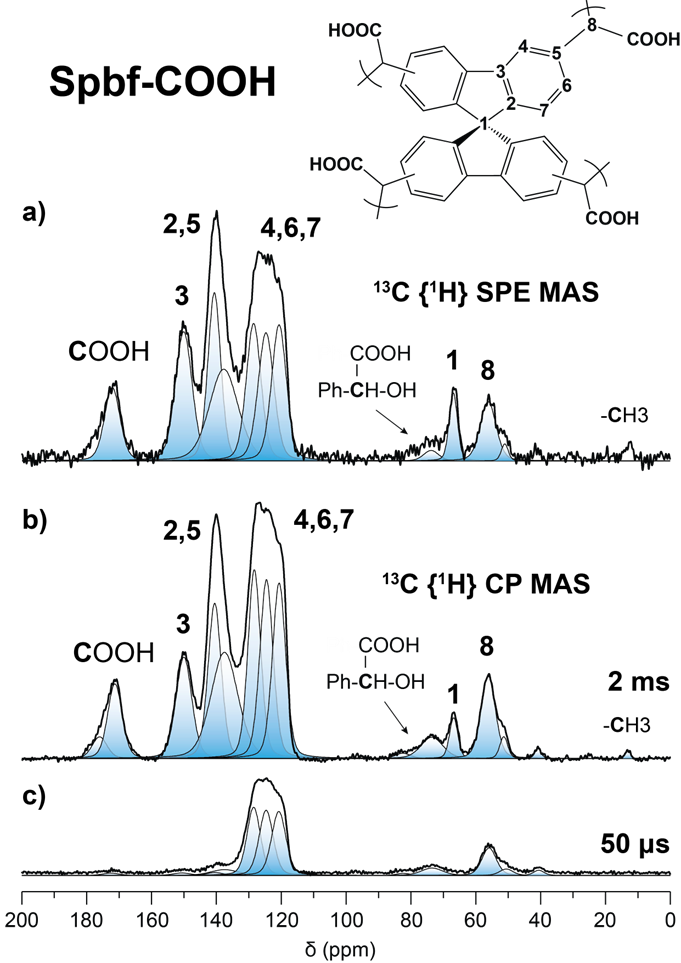


**Figure S22**. ^13^C MAS NMR analysis of **Spbf-COOH** performed at room temperature, 7.04 T, with a spinning speed of 12.5 kHz: a) quantitative ^13^C {^1^H} SPE spectrum collected with a recycle delay of 60 s; ^13^C {^1^H} CP spectra collected with a contact time of 2 ms (b) and 0.05 ms (c).

**Table S5**. ^13^C chemical shifts of **Spbf-COOH** from the simulation of ^13^C {^1^H} SPE MAS spectrum collected at room temperature, 7.04 T, with a spinning speed of 12.5 kHz and a recycle delay of 60 s.

| **Spbf-HCP-COOH** | **Assignment** | **δ (ppm)**  **^13^C {^1^H}**  **SPE MAS** | **Amount (%)** |
| --- | --- | --- | --- |
| 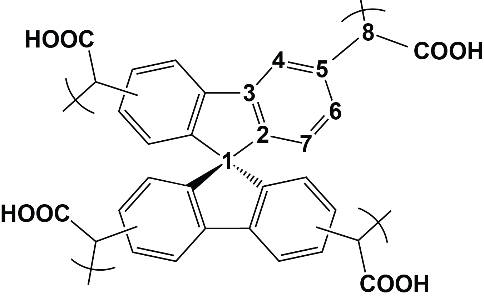 | **C**OOH | 172.0 | 7.1 |
|  | **3** | 150.0 | 81.8 |
|  | **2, 5** | 140.5, 137.6 |  |
|  | **4, 6, 7** | 128.4, 124.6, 120.5 |  |
|  | -**C**H-OH-COOH | 73.6 | 1.0 |
|  | **1** | 66.8 | 3.5 |
|  | **8** | 56.1, 50.9 | 6.6 |

**Spbf-COOH**: The total area of C2, C3, C4, C5, C6 and C7 peaks correspond to 81.8, thus each aromatic carbon atom counts for 13.6. The ratio between aromatic and bridging units is 2:1, thus each CH and COOH is expected to be 6.8. The areas of COOH and C8 signals are 6.8 and 6.4 (average 6.6), indicating each aromatic unit is connected to another unity by an acetic group. The area of CHOH-COOH pendants corresponds to 1, giving rise to a ratio of 0.07 with respect to each aromatic unit. The bridging unit-aromatic ring ratio corresponds to 6.6 / 13.6 = 0.48. per each aromatic unit.


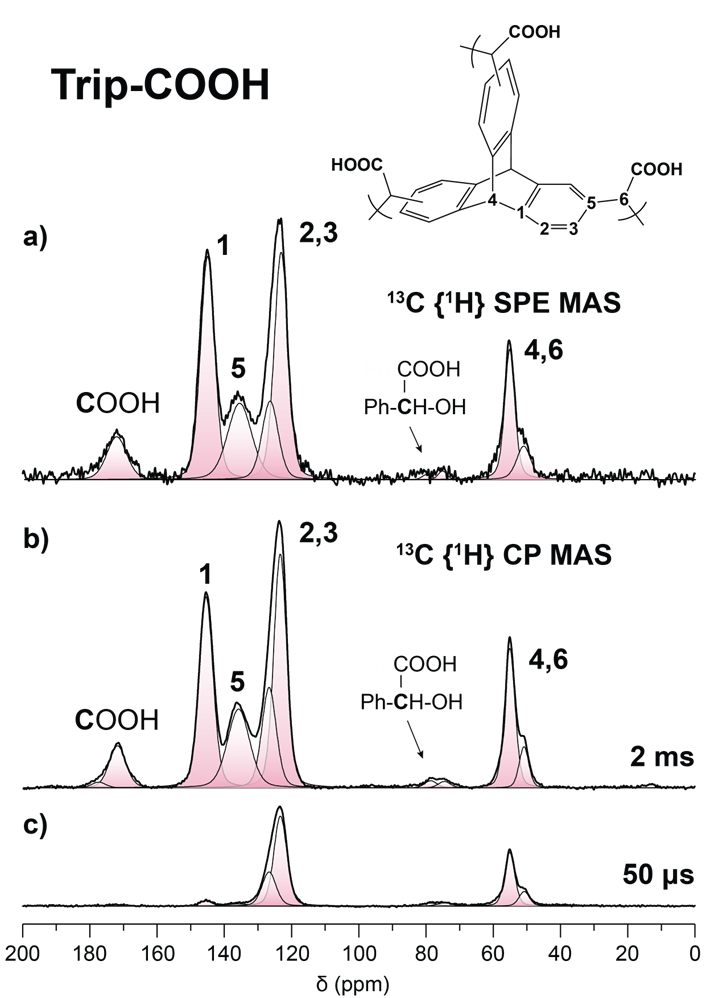


**Figure S23**. ^13^C MAS NMR analysis of **Trip-COOH** performed at room temperature, 7.04 T, with a spinning speed of 12.5 kHz: a) ^13^C {^1^H} SPE spectrum collected with a recycle delay of 60 s; ^13^C {^1^H} CP spectra collected with a contact time of 2 ms (b) and 0.05 ms (c).

**Table S6.** ^13^C chemical shifts of **Trip-COOH** from the simulation of ^13^C {^1^H} SPE MAS spectrum collected at room temperature, 7.04 T, with a spinning speed of 12.5 kHz and a recycle delay of 60 s.

| **Trip-COOH** | **Assignment** | **δ (ppm)**  **^13^C {^1^H}**  **SPE MAS** | **Amount (%)** |
| --- | --- | --- | --- |
| 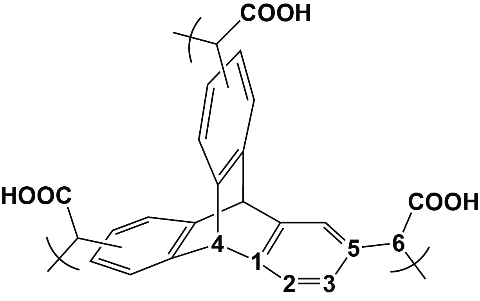 | **C**OOH | 172.5 | 6.7 |
|  | **1** | 145.2 | 76.8 |
|  | **5** | 135.7 |  |
|  | **2, 3** | 126.6, 123.3 |  |
|  | **C**H-OH-COOH | 80.0, 75.3 | 1.0 |
|  | **4, 6** | 55.3, 51.1 | 15.5 |

**Trip-COOH**: The total area of C1, C2, C3, and C5 peaks corresponds to 76.8, thus each aromatic carbon atom accounts for 12.8. The ratio between aromatic and bridging units is 2:1, thus each CH and COOH should be 6.4. The area of the COOH signal is 6.7, indicating each aromatic unit is connected to another unit by an acetic group. The area of CHOH-COOH pendants corresponds to 1, giving rise to a ratio of 0.08 with respect to each aromatic unit. The bridging unit-aromatic ring ratio corresponds to 6.6 / 13.6 = 0.48. per each aromatic unit.

corresponds to 6.7 / 12.8 = 0.52 per each aromatic unit.


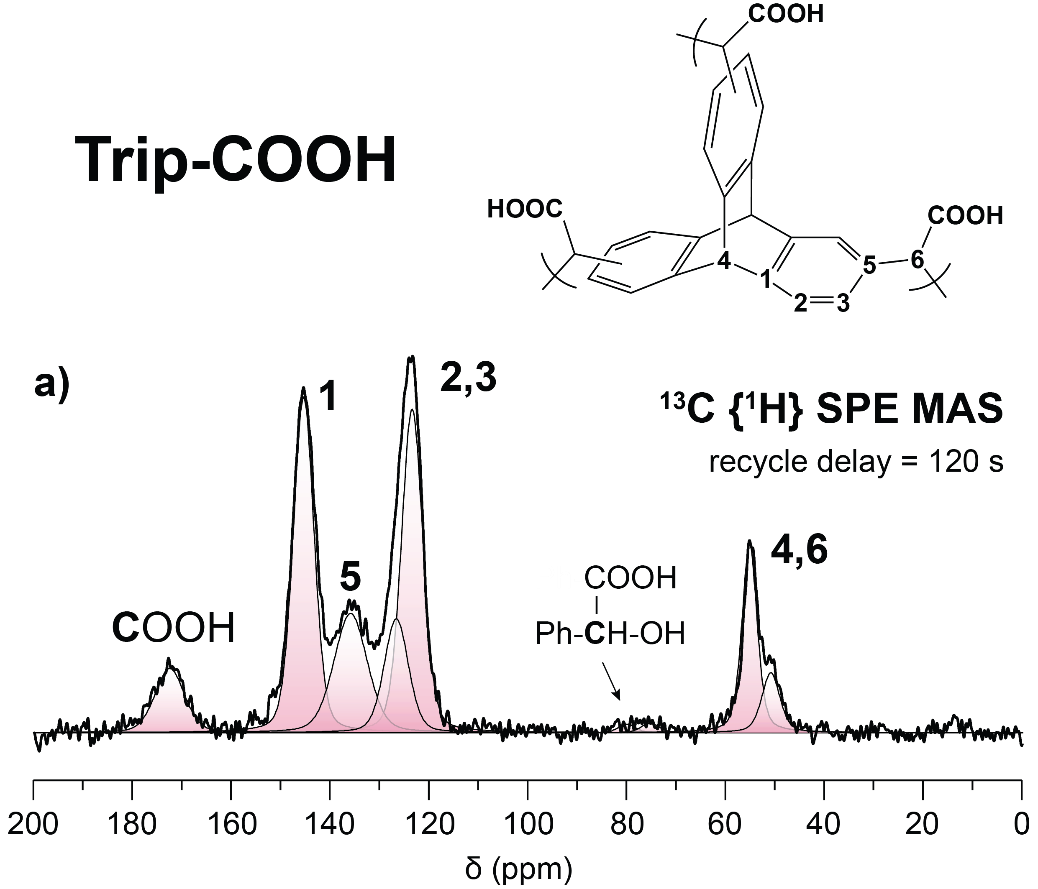


**Figure S24.** ^13^C {^1^H} SPE MAS spectrum of **Trip-COOH** collected at room temperature, 7.04 T, with a spinning speed of 12.5 kHz and a recycle delay of 120 s.

**Table S7.** ^13^C T_1_ TORCHIA NMR relaxation times of **Trip-COOH** collected at 298 K, 7.04 T and 12.5 kHz.

| **Assignment** | **^13^C T_1_ (s) of Trip-COOH** |
| --- | --- |
| **C**OOH | 3.9 |
| **C**Harom | 4.2 |
| **C**H | 4.4 |

^13^C T_1_ spin-lattice relaxation times were measured at 75.5 MHz by applying the TORCHIA pulse sequence.^[2]^ The experiment was collected at 298 K with a spinning speed of 12.5 kHz.

**
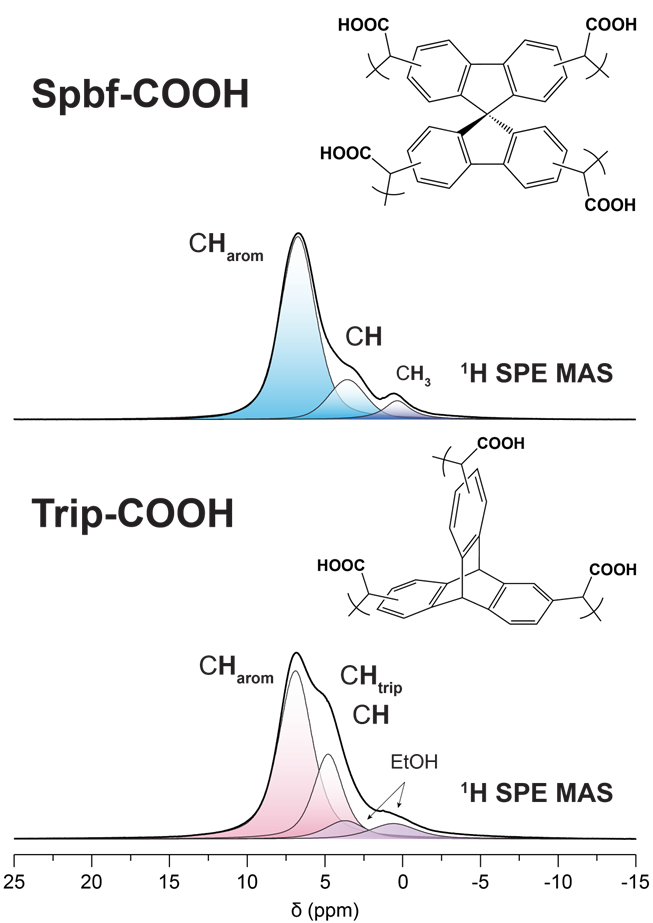
**

**Figure S25**. ^1^H SPE MAS spectra of **Spbf-COOH** (top) and **Trip-COOH** (bottom) collected at room temperature, 14.09 T, with a spinning speed of 30.0 kHz and a recycle delay of 20 s.

**Table S8**. ^1^H chemical shifts of **Spbf-COOH** and **Trip-COOH** from the simulation of quantitative MAS spectra collected at room temperature, 30.0 kHz and 14.09 T.

| **Spbf-COOH** | **Assignment** | **δ (ppm)**  **^1^H SPE MAS** | **Amount (%)** |
| --- | --- | --- | --- |
| 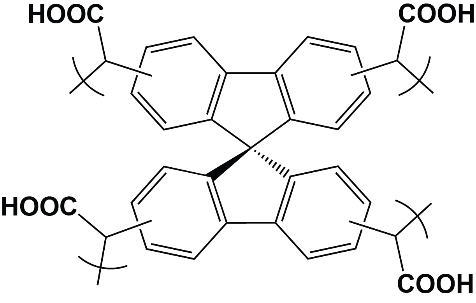 | C**Harom** | 6.7 | 76.1 |
|  | C**H** | 3.7 | 16.7 |
|  | **CH_3_** | 0.3 | 7.2 |
| **Trip-COOH** | **Assignment** | **δ (ppm)**  **^1^H SPE MAS** | **Amount (%)** |
| 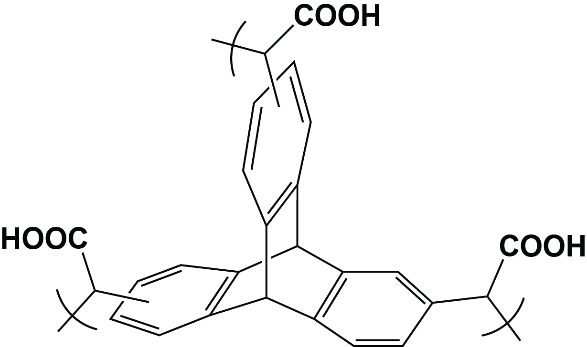 | C**Harom** | 6.9 | 59.1 |
|  | C**H** | 4.8 | 26.0 |
|  | **EtOH** | 3.7, 0.6 | 15.0 |

**Infrared spectroscopy**

**
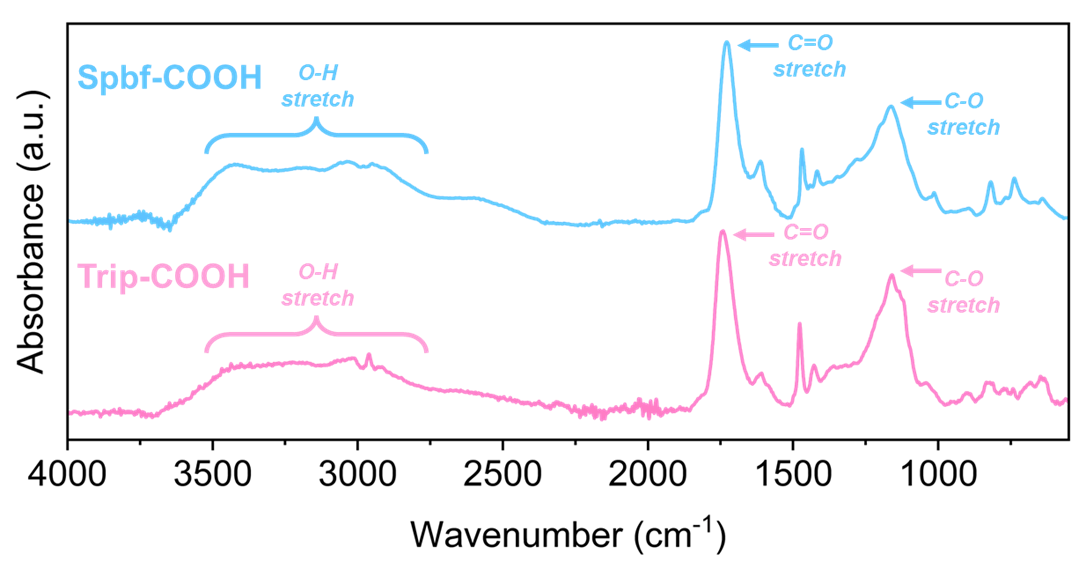
**

**Figure S26**. FT-IR spectra of **Spbf-COOH** (top, light blue) and **Trip-COOH** (bottom, pink) collected from 525 cm^-1^ to 4000 cm^-1^. The characteristic vibrational bands associated with the carboxylic acid functional groups are highlighted in the figure.

**Thermal analysis**

Thermogravimetric analyses were conducted under an inert atmosphere (N_2_ flow, 50 mL/min) and under oxidative conditions (dry air flow, 50 mL/min). Analyses under inert atmospheres allowed the observation of a first weight loss between 230°C and 500°C associated with the frameworks' decarboxylation reaction. TGA analysis under oxidative conditions permitted to quantify the residual weight after complete combustion of the organic substances.

**
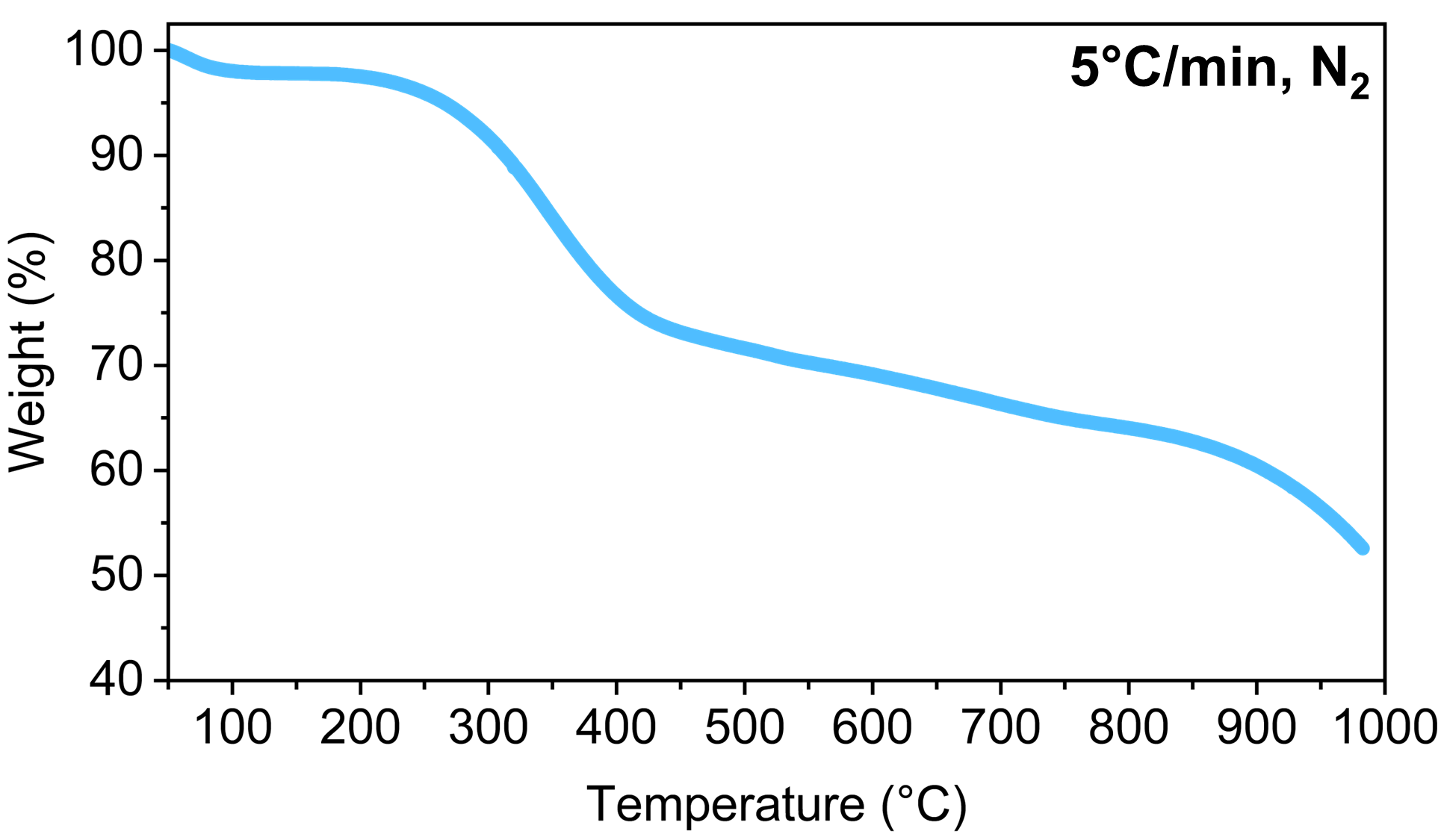
**

**
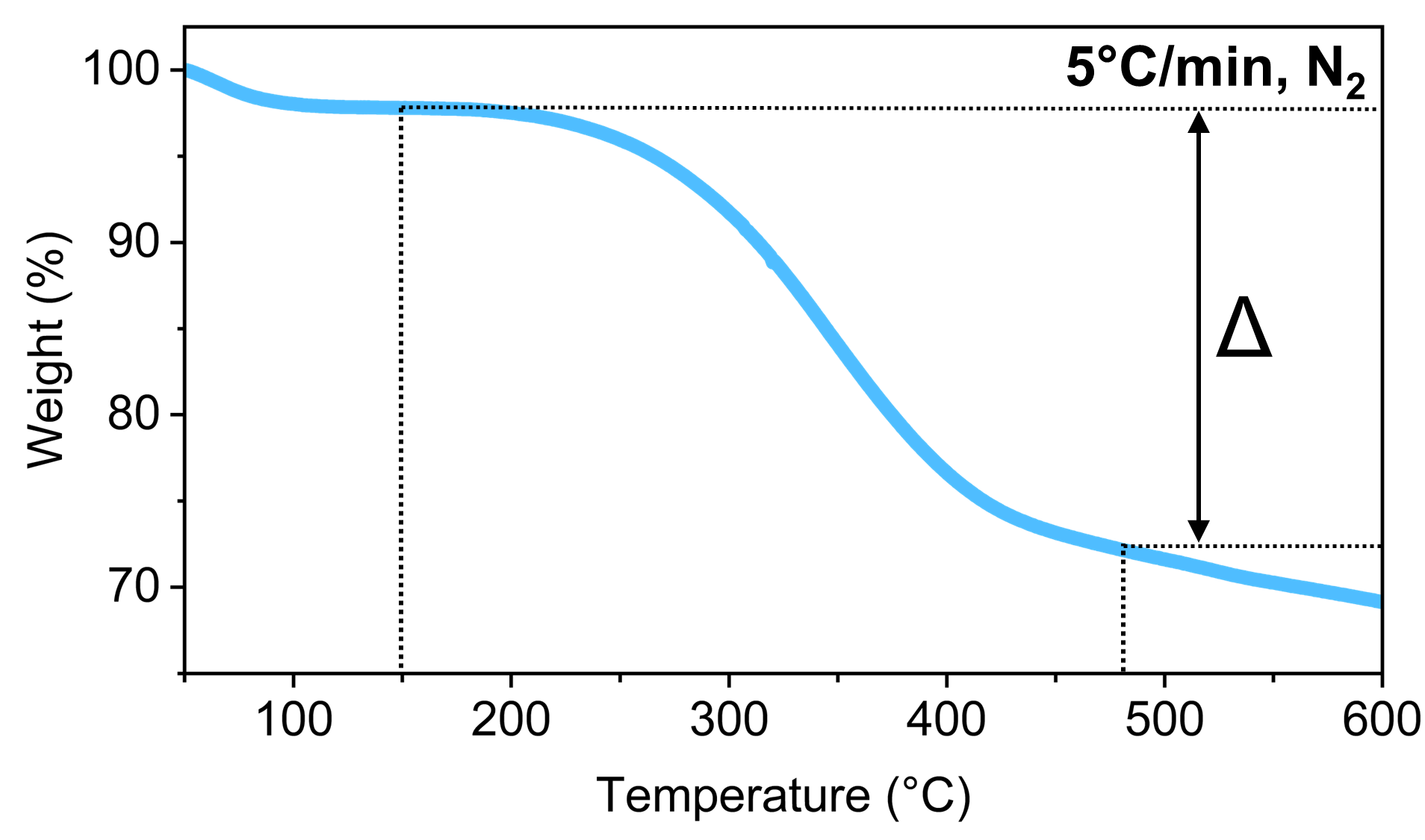
**

**Figure S27**. Top: thermal analysis of **Spbf-COOH** collected from 50°C to 1000°C. The thermogram was collected under N_2_ flow (50 mL/min) with a heating rate of 5°C/min. Bottom: enlargement of the thermogram of **Spbf-COOH** between 50°C and 600°C. The thermogram was collected under N_2_ flow (50 mL/min) with a heating rate of 5°C/min. The normalized weight loss of 25 wt% between 150°C and 480°C was ascribed to the decarboxylation reaction (see FT-IR coupled TGA analysis below).

**
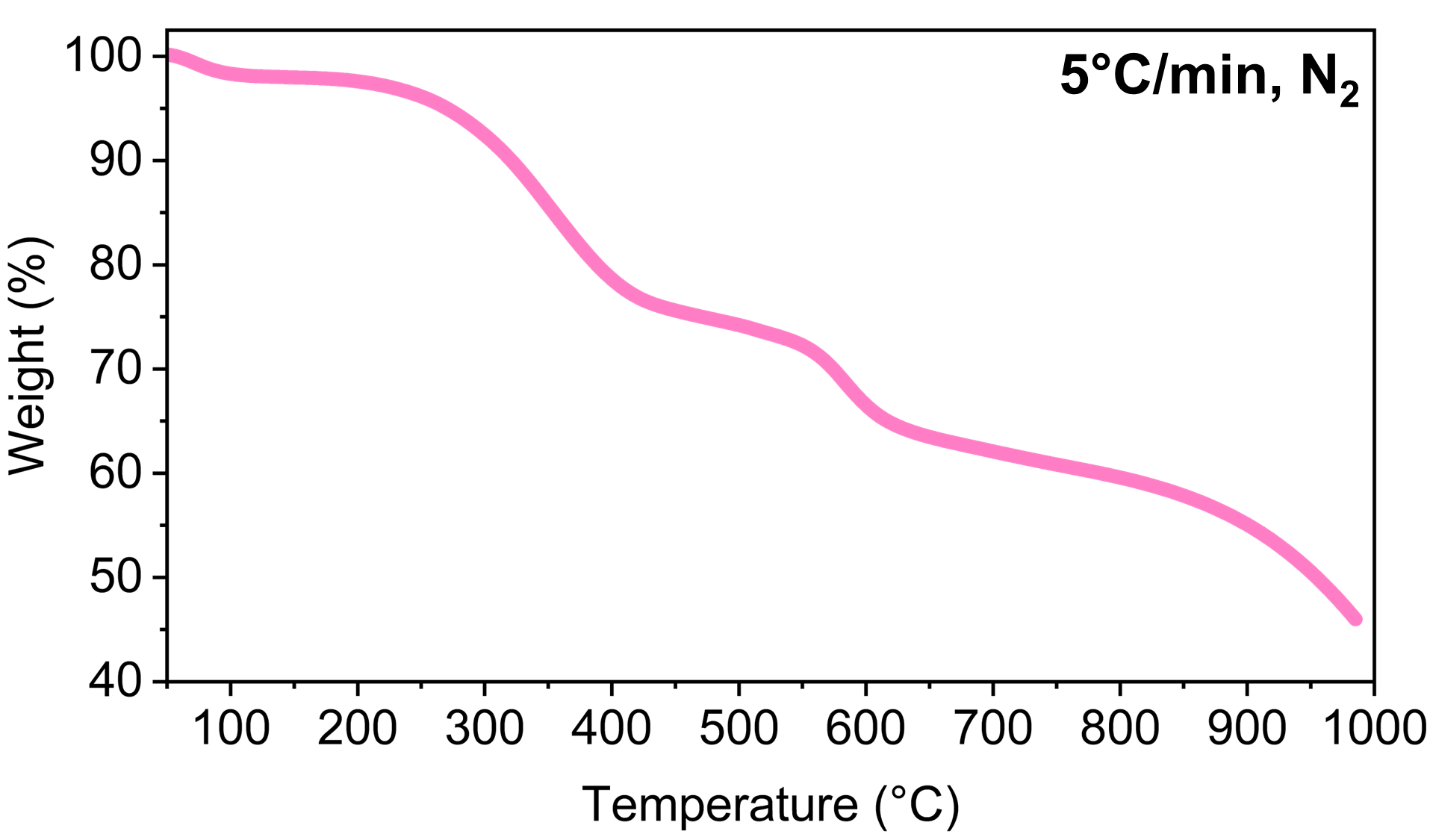
**
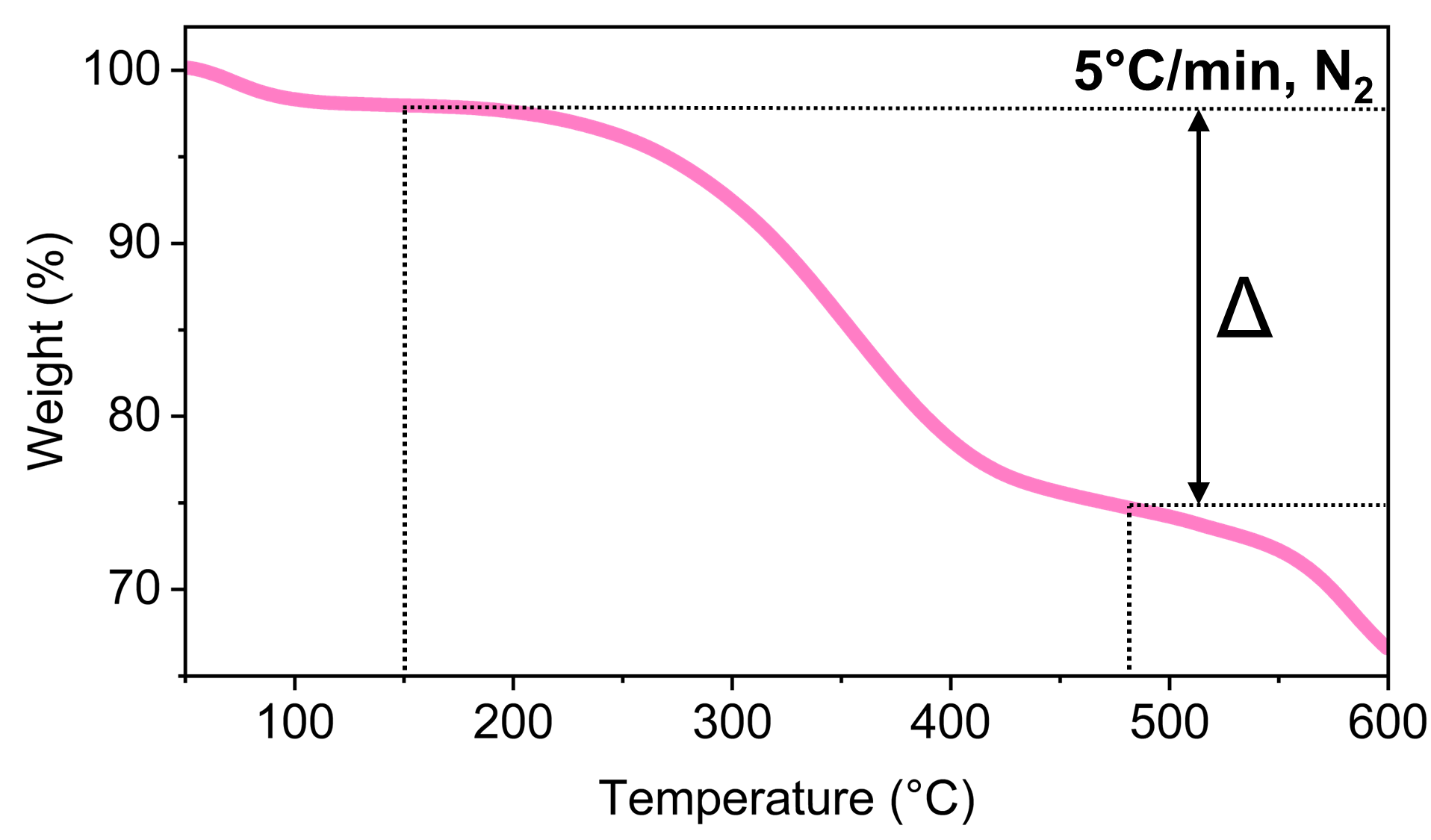


**Figure S28**. Top: thermal analysis of **Trip-COOH** collected from 50°C to 1000°C. The thermogram was collected under N_2_ flow (50 mL/min) with a heating rate of 5°C/min. Bottom: enlargement of the thermogram of **Trip-COOH** between 50°C and 600°C. The thermogram was collected under N_2_ flow (50 mL/min) with a heating rate of 5°C/min. The normalized weight loss of 23.7 wt% between 150°C and 480°C was ascribed to the decarboxylation reaction (see FT-IR coupled TGA analysis below).

**
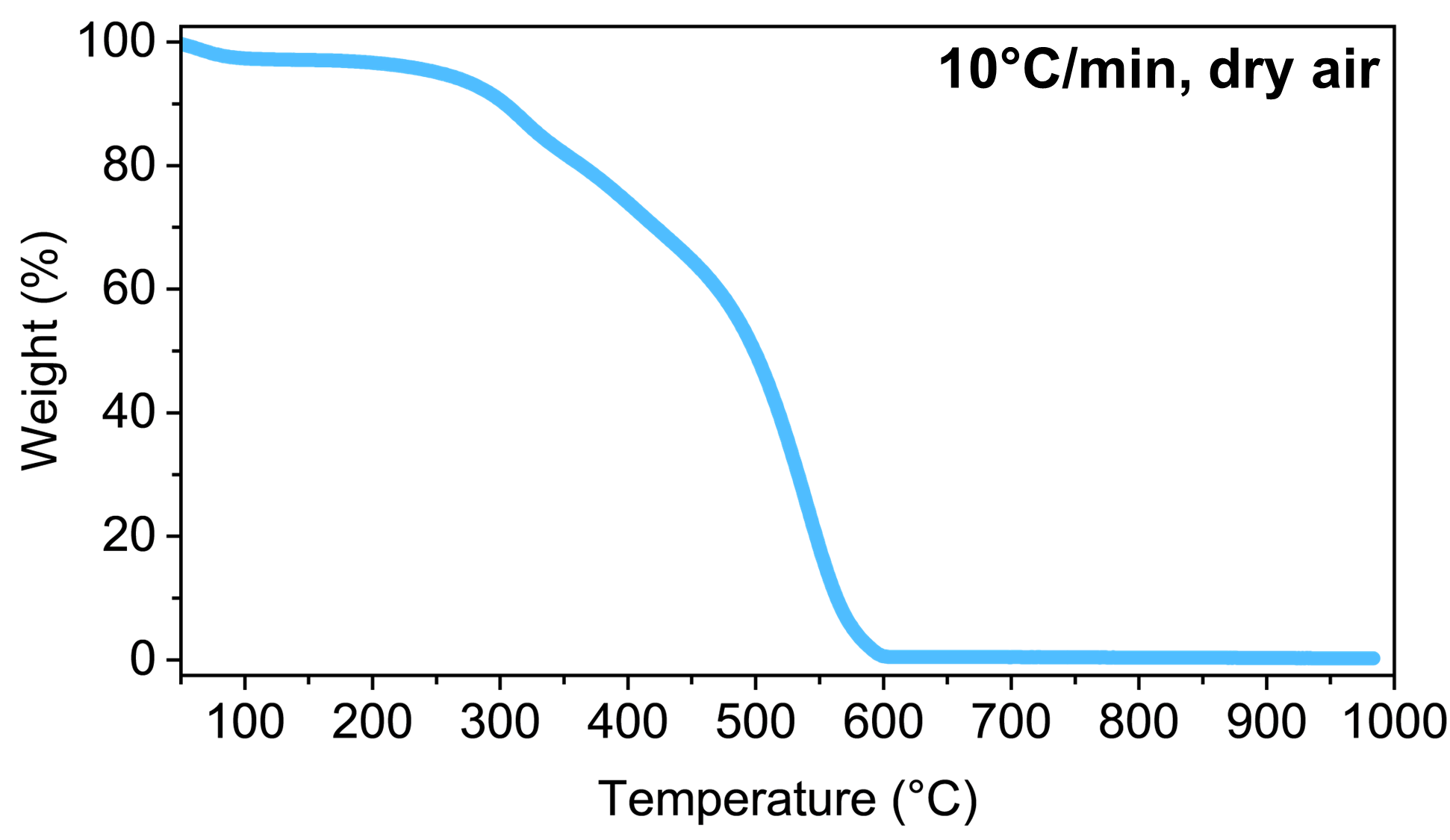
**


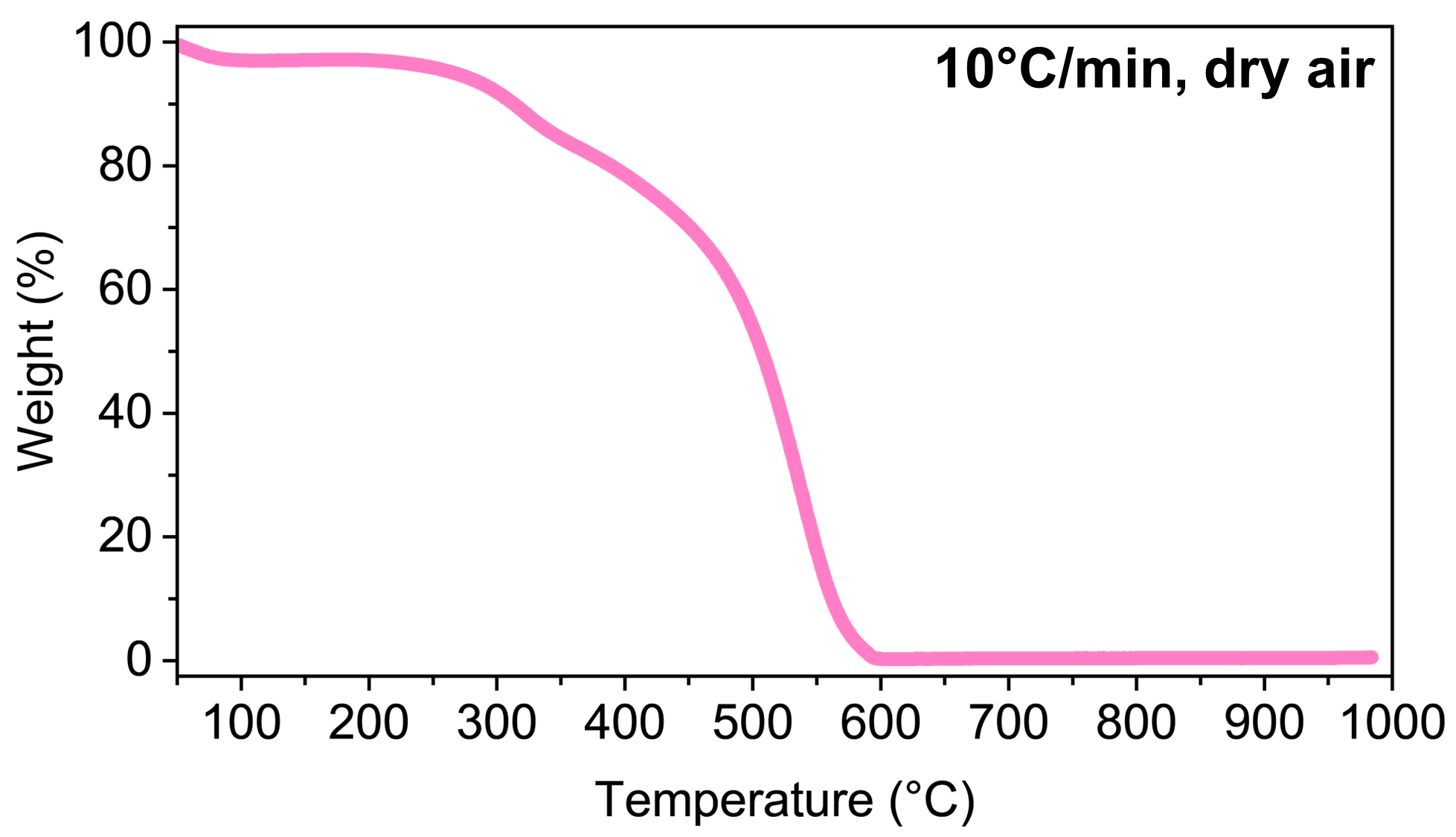


**Figure S29**. Top: thermal analysis of **Spbf-COOH** collected from 50°C to 1000°C. The thermogram was collected under dry air flow (50 mL/min) with a heating rate of 10°C/min. The residue at 700°C is about 0.2 wt%. Bottom: Thermal analysis of **Trip-COOH** collected from 50°C to 1000°C. The thermogram was collected under dry air flow (50 mL/min) with a heating rate of 10°C/min. The residue at 700°C is about 0.3 wt%.

**FT-IR coupled TGA analysis**

The main compounds identified in the evolved gas during the thermal decomposition of all the tested materials were mainly ascribed to CO_2_ release.^[11,12]^

**
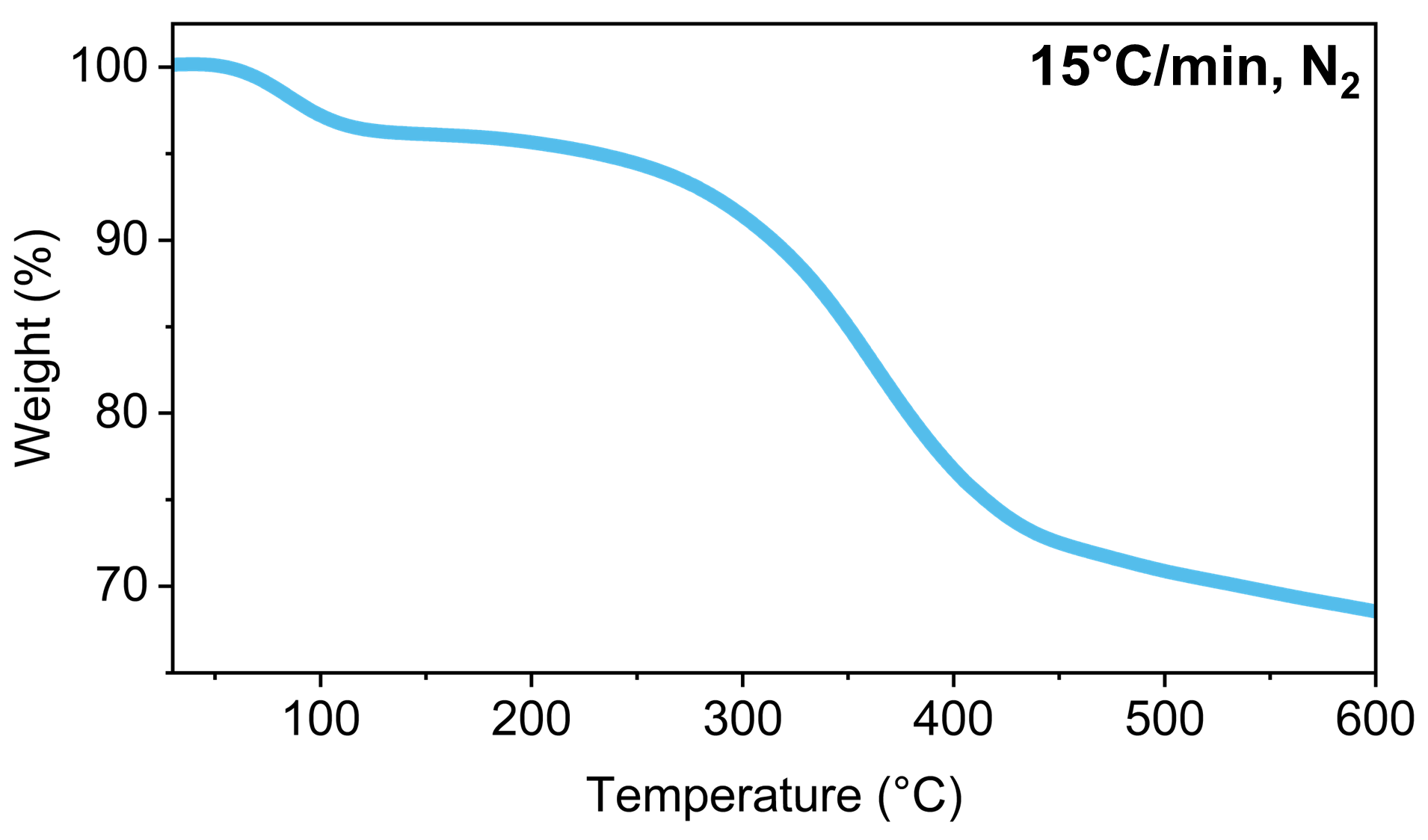
**

**
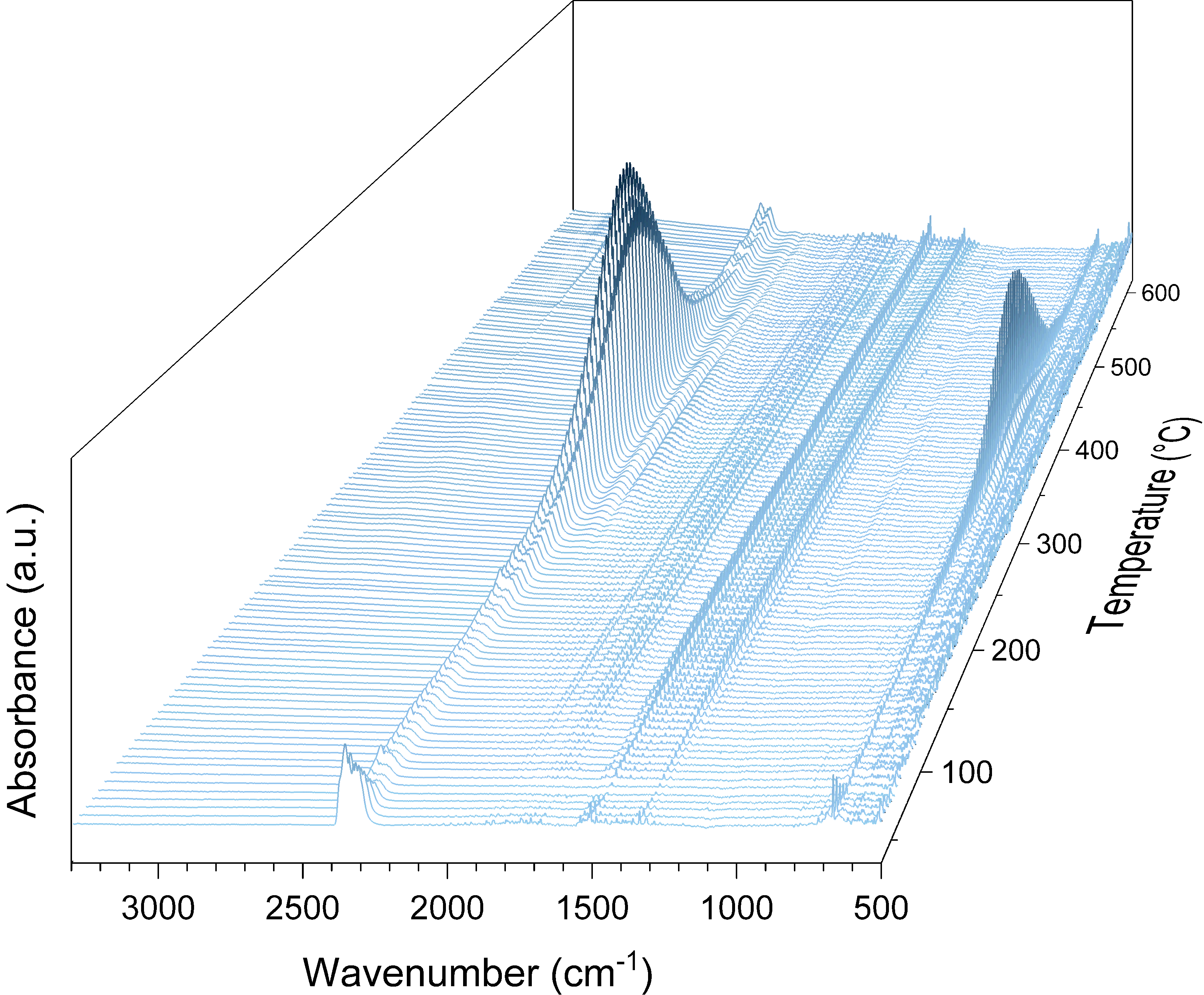
**

**Figure S30**. Top: Thermal analysis of **Spbf-COOH** coupled with FT-IR spectroscopy. The thermogram was collected under N_2_ flow (50 mL/min) with a heating rate of 15°C/min. Bottom: 3D plot of TGA-IR data of **Spbf-COOH**. The plot displays the FT-IR spectra as a function of the sample temperature. The release of CO_2_ molecules in the temperature range between 50°C and 150°C is related to the desorption of guest molecular species from the porous framework. In the temperature range between 300°C and 500°C, strong IR-active bands centered at 668 cm^-1^ and between 2300/2350 cm^-1^ are due to the bending and asymmetric stretching of CO_2_, respectively.

**
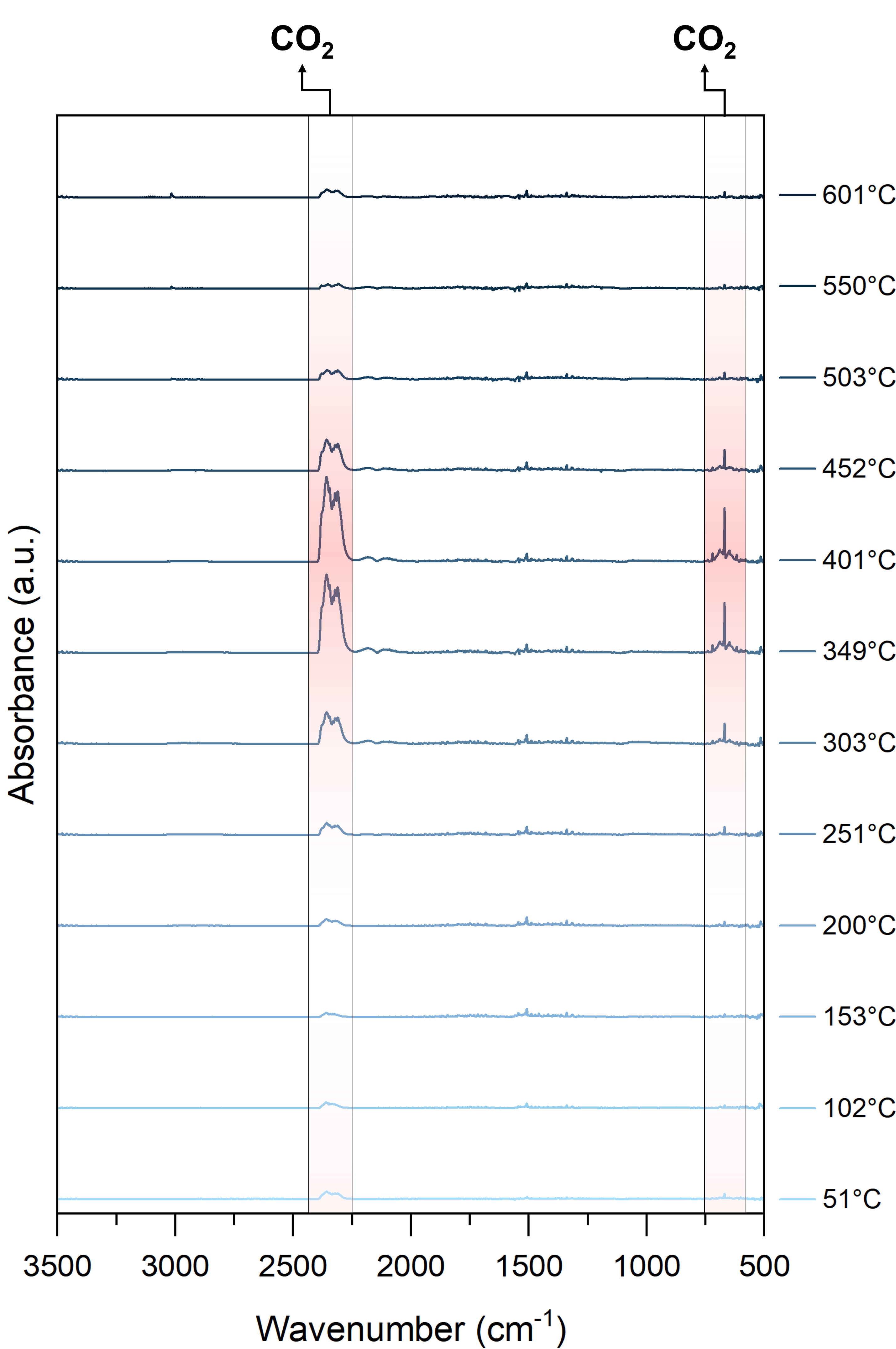
**

**Figure S31**. Selected FT-IR spectra of the evolved gases/vapors collected at different temperatures from **Spbf-COOH**. The colored boxes highlight the CO_2_ vibrational bands.

**
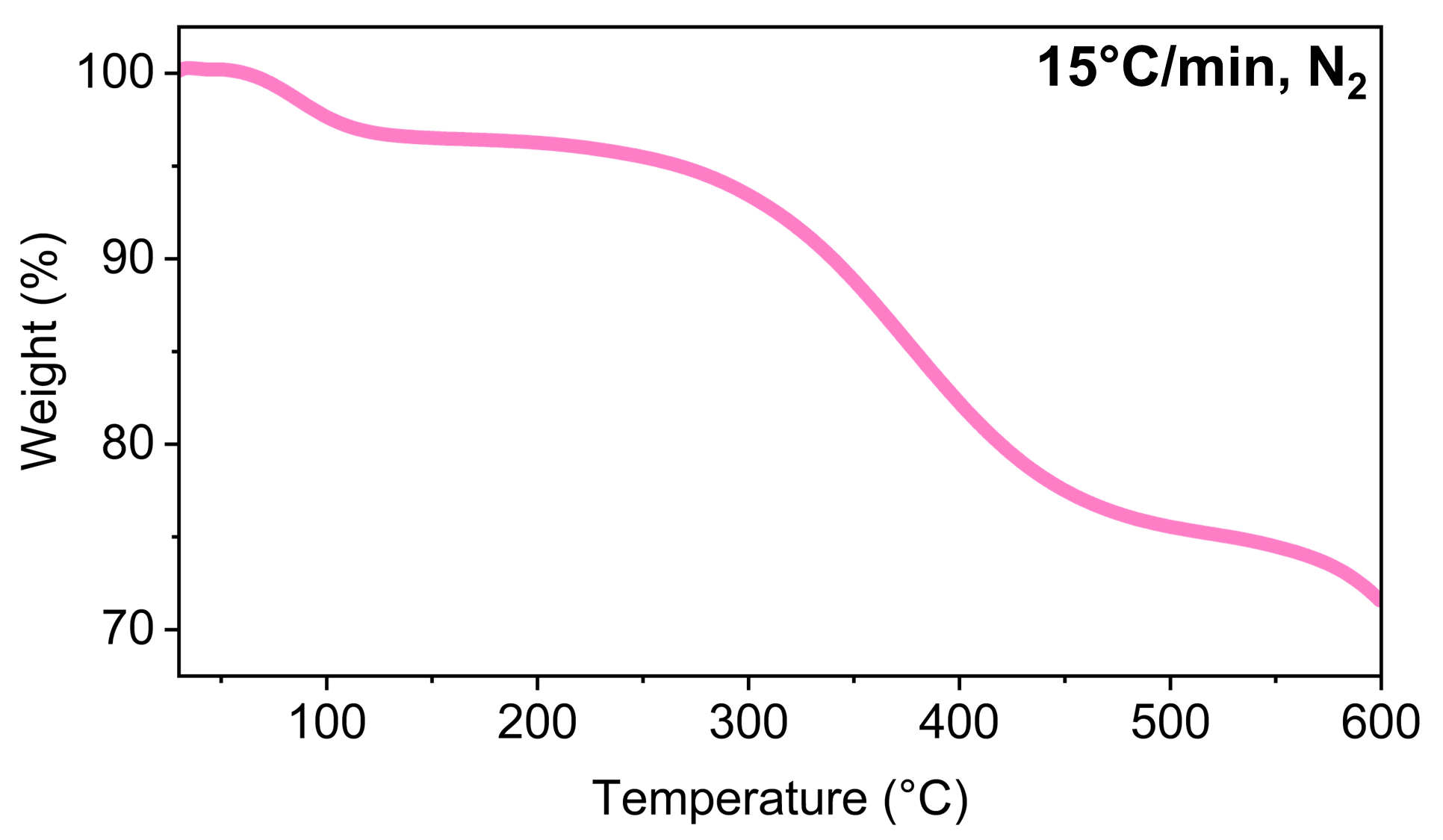
**

**
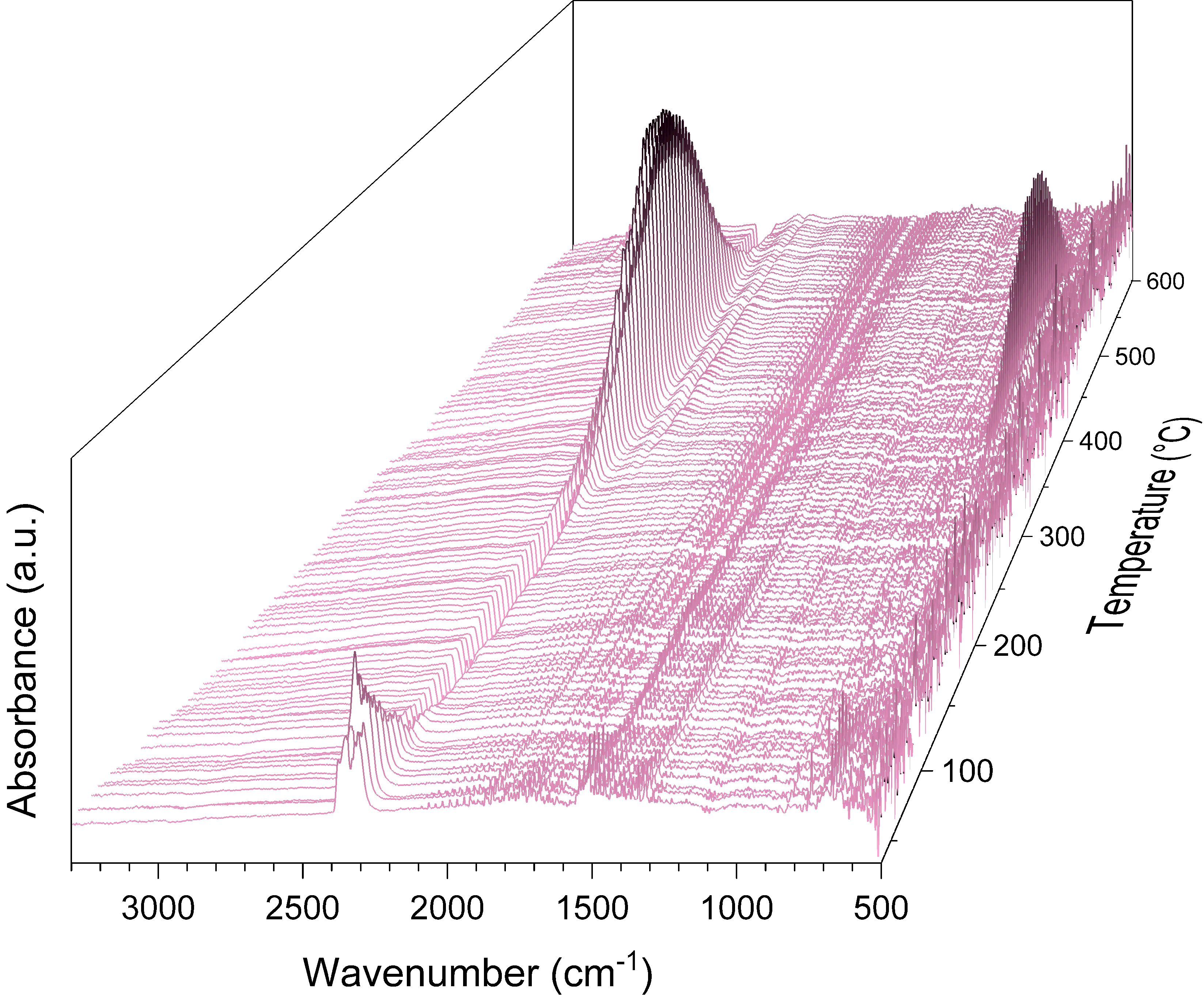
**

**Figure S32**. Top: thermal analysis of **Trip-COOH** coupled with FT-IR spectroscopy. The thermogram was collected under N_2_ flow (50 mL/min) with a heating rate of 15°C/min. Bottom: 3D plot of TGA-IR data of **Trip-COOH**. The plot displays the FT-IR spectra as a function of the sample temperature. The release of CO_2_ molecules in the temperature range between 50°C and 150°C is related to the desorption of guest molecular species from the porous framework. In the temperature range between 300°C and 500°C, strong IR-active bands centered at 668 cm^-1^ and between 2300/2350 cm^-1^ are due to the bending and asymmetric stretching of CO_2_, respectively.

**
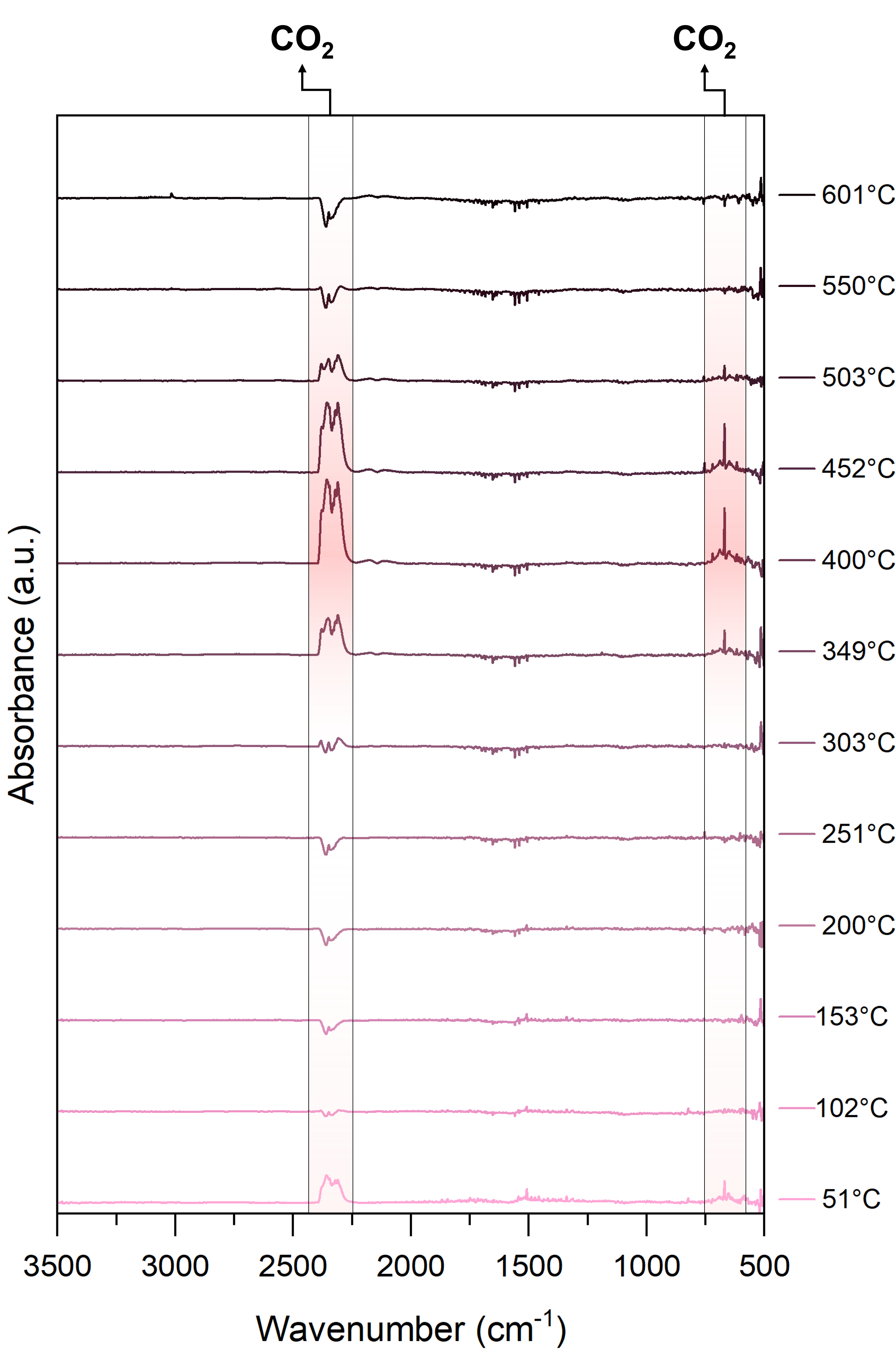
**

**Figure S33**. Selected FT-IR spectra of the evolved gases/vapors collected at different temperatures from **Trip-COOH**. The colored boxes highlight the CO_2_ vibrational bands.

**Elemental Analysis**

**Table S9**. Elemental analysis composition of **Spbf-COOH** and **Trip-COOH.**

| **Sample** | **C(%)** | | **H(%)** | | **N(%)** | | **S(%)** | | **O(%)** | |
| --- | --- | --- | --- | --- | --- | --- | --- | --- | --- | --- |
|  | **Exp.** | **Calc.** | **Exp.** | **Calc.** | **Exp.** | **Calc.** | **Exp.** | **Calc.** | **Exp.** | **Calc.** |
| **Spbf-COOH** | 74.30 | 78.7 | 4.120 | 4.1 | 0.30 | 0 | 0.021 | 0 | 20.95 | 17.2 |
| **Trip-COOH** | 77.56 | 78.9 | 4.487 | 4.4 | 0.34 | 0 | 0.000 | 0 | 18.27 | 16.6 |

* The amount of C, H, N, S and O were calculated based on the composition from solid-state NMR experiments.

**Powder X-ray diffraction**


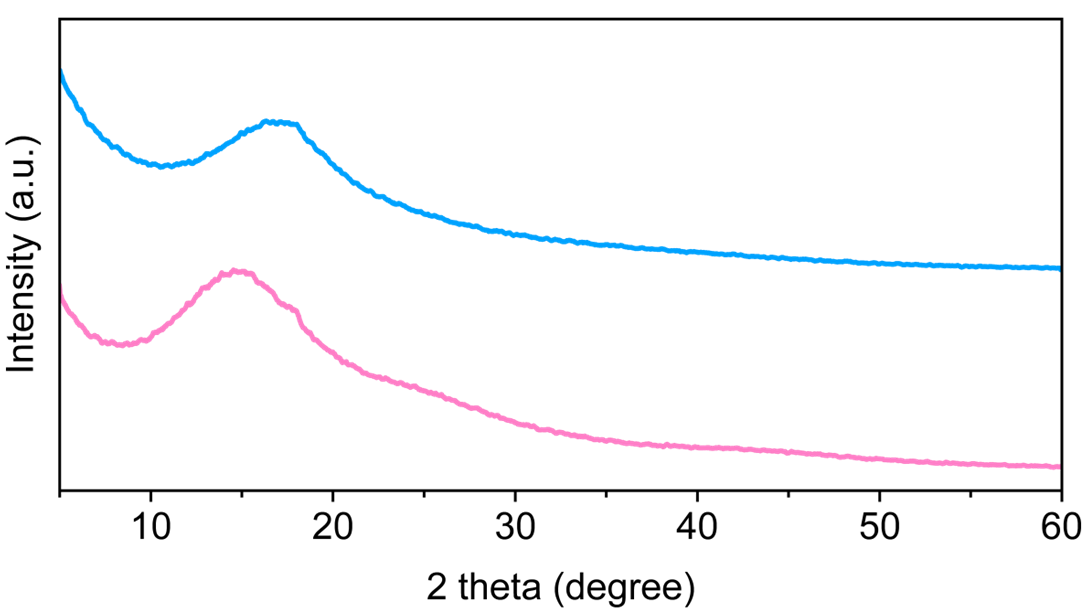


**Figure S34**. Powder X-ray diffraction of **Spbf-COOH** (top, light blue) and **Trip-COOH** (bottom, pink) collected from 5 to 60 2 theta degrees.

- **Characterization of the post-functionalized samples Trip-COOLi**, **Trip-COONa** and **Trip-COOMe**

**^13^C and ^1^H solid-state NMR spectroscopy - Trip-COOMe**

**
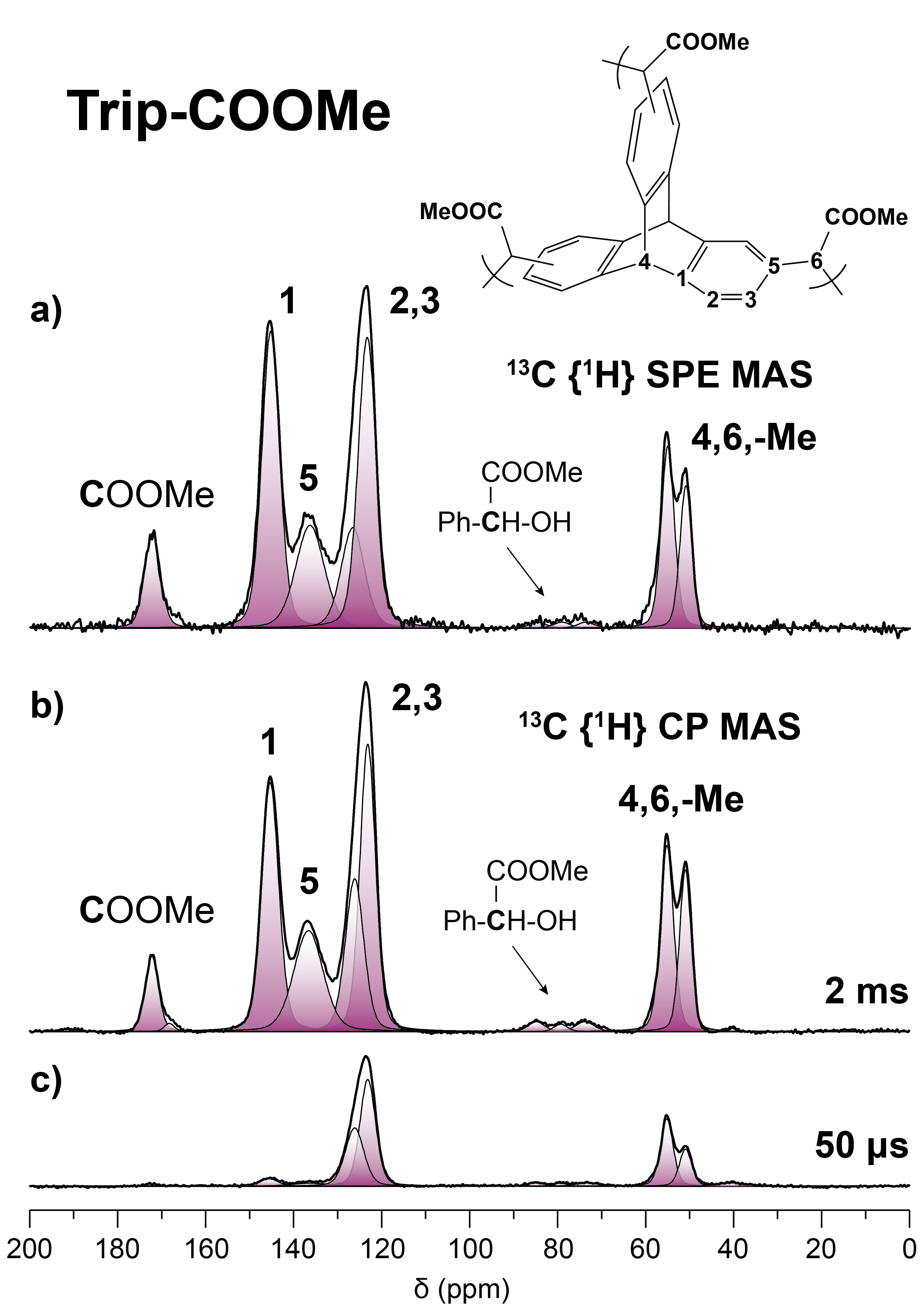
**

**Figure S35**. ^13^C MAS NMR analysis of **Trip-COOMe** performed at room temperature, 7.04 T, with a spinning speed of 12.5 kHz: a) ^13^C {^1^H} SPE spectrum collected with a recycle delay of 60 s; ^13^C {^1^H} CP spectra collected with a contact time of 2 ms (b) and 0.05 ms (c).

**Table S10**. ^13^C chemical shifts of Trip-COOH/Me from the simulation of ^13^C {^1^H} SPE MAS spectrum collected at room temperature, 7.04 T, with a spinning speed of 12.5 kHz and a recycle delay of 60 s.

| **Trip-COOH/Me** | **Assignment** | **δ (ppm)**  **^13^C {^1^H}**  **SPE MAS** | **Amount (%)** |
| --- | --- | --- | --- |
| 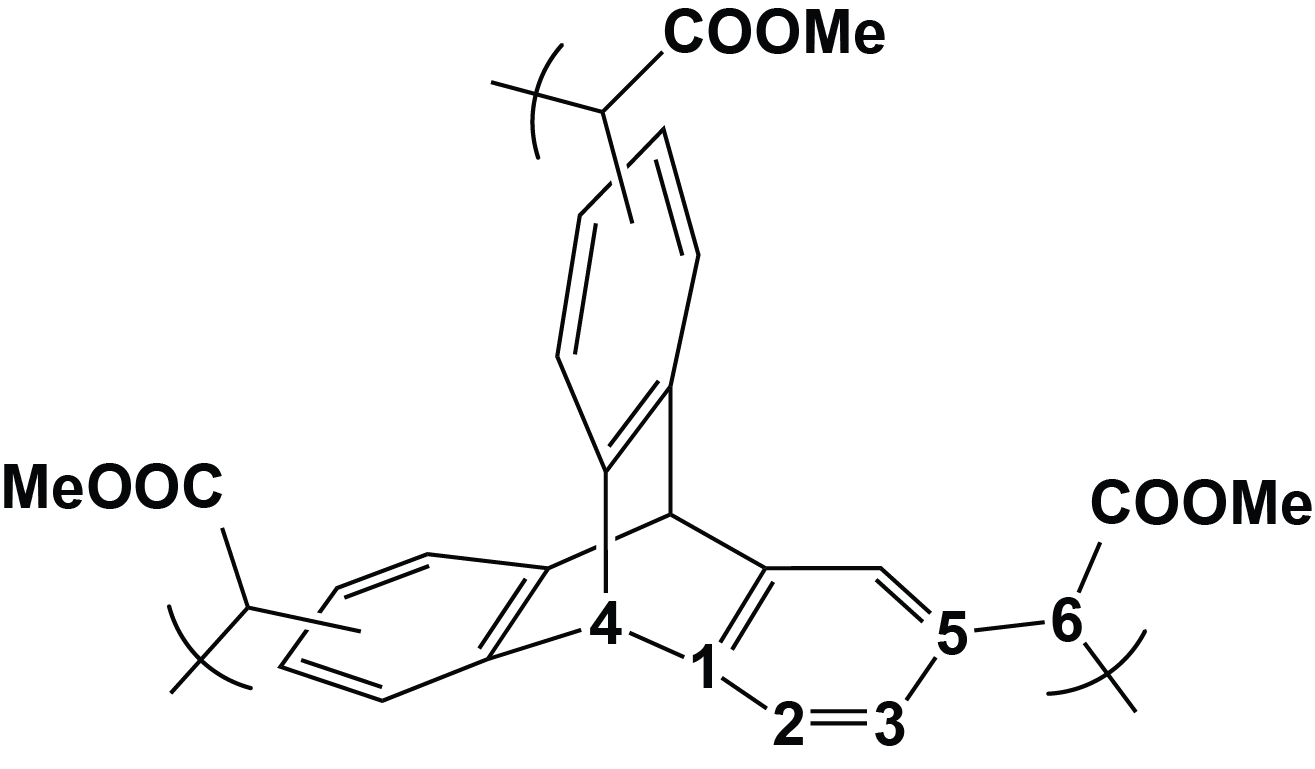 | **C**OOMe | 172.3 | 6.9 |
|  | **1** | 145.3 | 72.9 |
|  | **5** | 136.5 |  |
|  | **2, 3** | 126.8, 123.5 |  |
|  | **C**H-OH-COOMe | 84.8, 79.2, 73.7 | 1.3 |
|  | **4, 6, -Me** | 55.2, 51.0 | 18.9 |

**Trip-COOH/Me:** The total area of the aromatic groups is 72.9 and corresponds to 12.1 per each aromatic carbon. The signal intensity of COOH/COOMe groups at 172.3 ppm is 6.9 while the signal intensity of CH(OH)COOH/Me groups in 73.7-84.8 ppm region corresponds to 1.3. The amount of bridging CH-COOH/Me groups can be evaluated starting from the intensity of each aromatic group (12.1) and subtracting the CH(OH)COOH/Me group intensity of 1.3 and dividing the result by two since (12.1-1.3 = 10.8 / 2 = 5.4). The estimated total COOH/COOMe groups corresponds to 6.7 (5.4 + 1.3) in agreement with the value of 6.9 obtained experimentally from the COOH/Me signal intensity. The signal intensity in the 51-55 ppm region is of 18.9: it corresponds to the signal intensity of the two trypticene carbons CH in the monomeric unit (12.1/3*2=8.1), the CH of CH-COOH/Me bridges (5.4) and the methyl groups of the methylester. The methyl group intensity can be estimated to be 5.35 (18.9 – 8.1 = 5.4) and the methyl fraction over the total COOH/COOMe intensity is 80.6% (5.4 / 6.7 * 100).

**
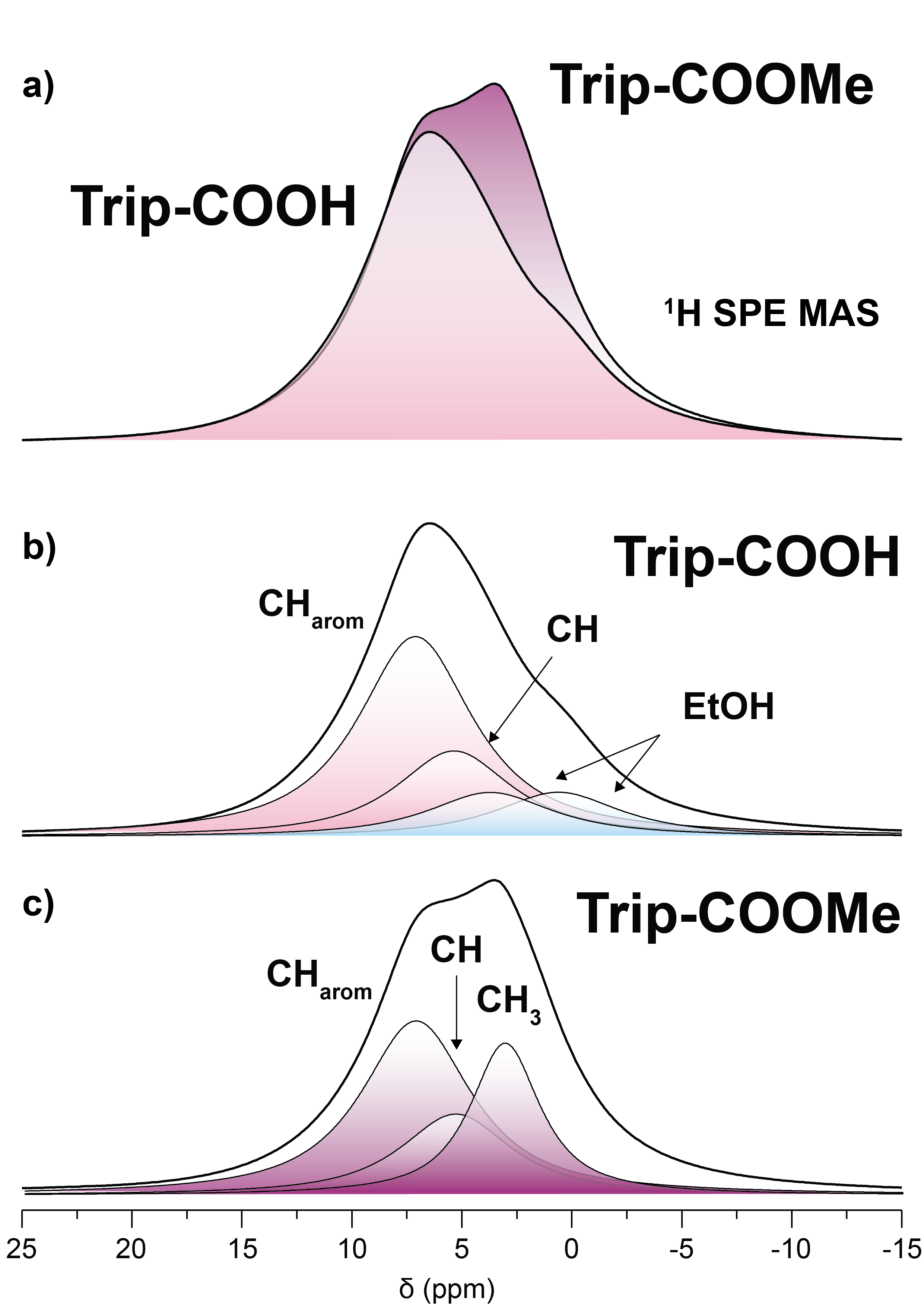
**

**Figure S36**. ^1^H SPE MAS spectra of **Trip-COOH** (b) and **Trip-COOMe** (c) collected at room temperature, 7.04 T, with a spinning speed of 12.5 kHz and a recycle delay of 20 s. a) Direct comparison between the two spectra: the intensities of the spectra were scaled to match the intensity of the deconvoluted peak associated with the C**H_arom_** signal.

**Table S11**. ^1^H chemical shifts of **Trip-COOH** and **Trip-COOMe** from the simulation of quantitative MAS spectra collected at room temperature, 30.0 kHz and 14.09 T.

| **Trip-COOH** | **Assignment** | **δ (ppm)**  **^1^H SPE MAS** | **Amount (%)** |
| --- | --- | --- | --- |
| 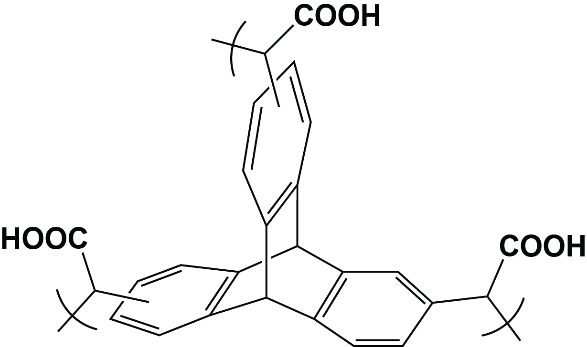 | C**Harom** | 7.1 | 52.5 |
|  | C**H** | 5.3 | 22.5 |
|  | **EtOH** | 3.7, 0.6 | 25.0 |
| **Trip-COOMe** | **Assignment** | **δ (ppm)**  **^1^H SPE MAS** | **Amount (%)** |
| 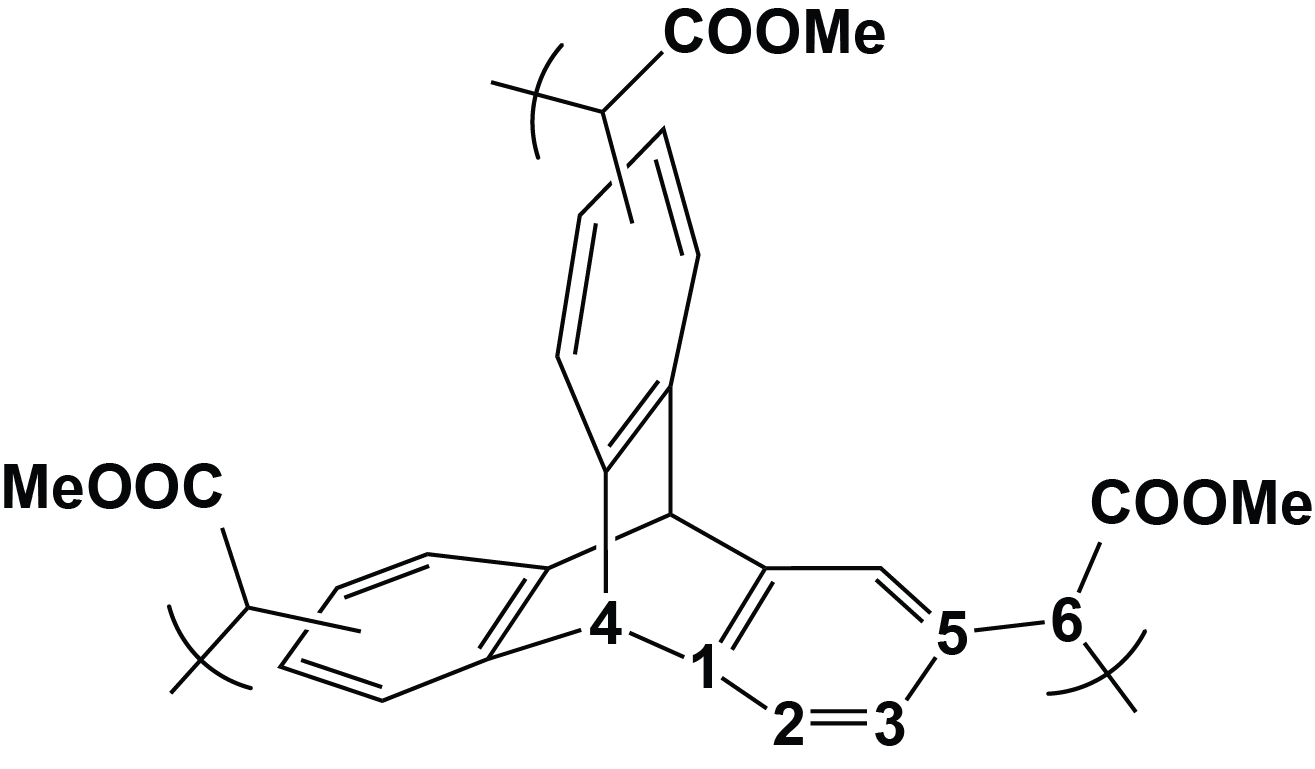 | C**Harom** | 7.1 | 49.0 |
|  | C**H** | 5.3 | 22.9 |
|  | C**H_3_** | 3.2 | 28.1 |

**Thermal analysis - Trip-COOMe**


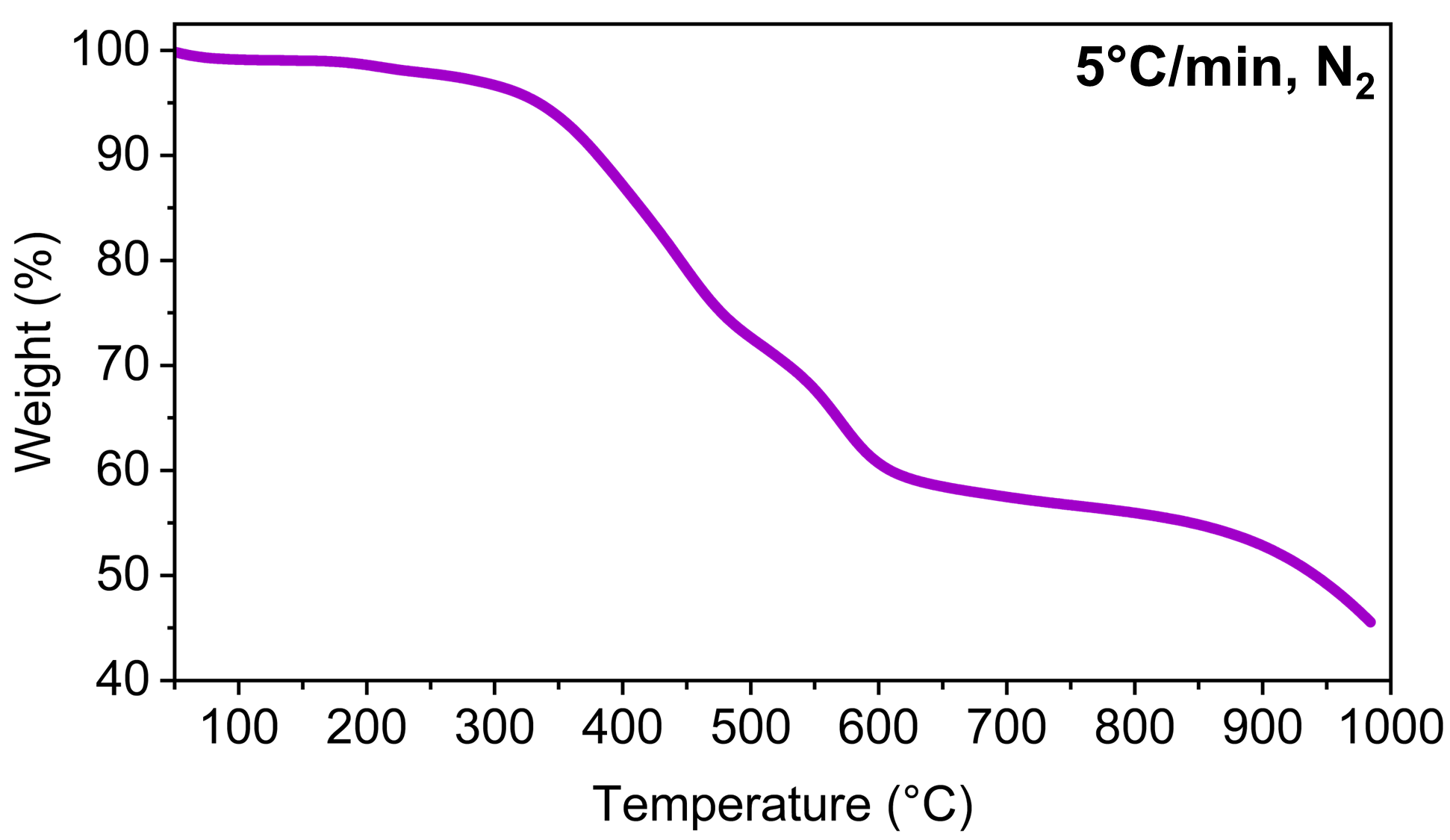


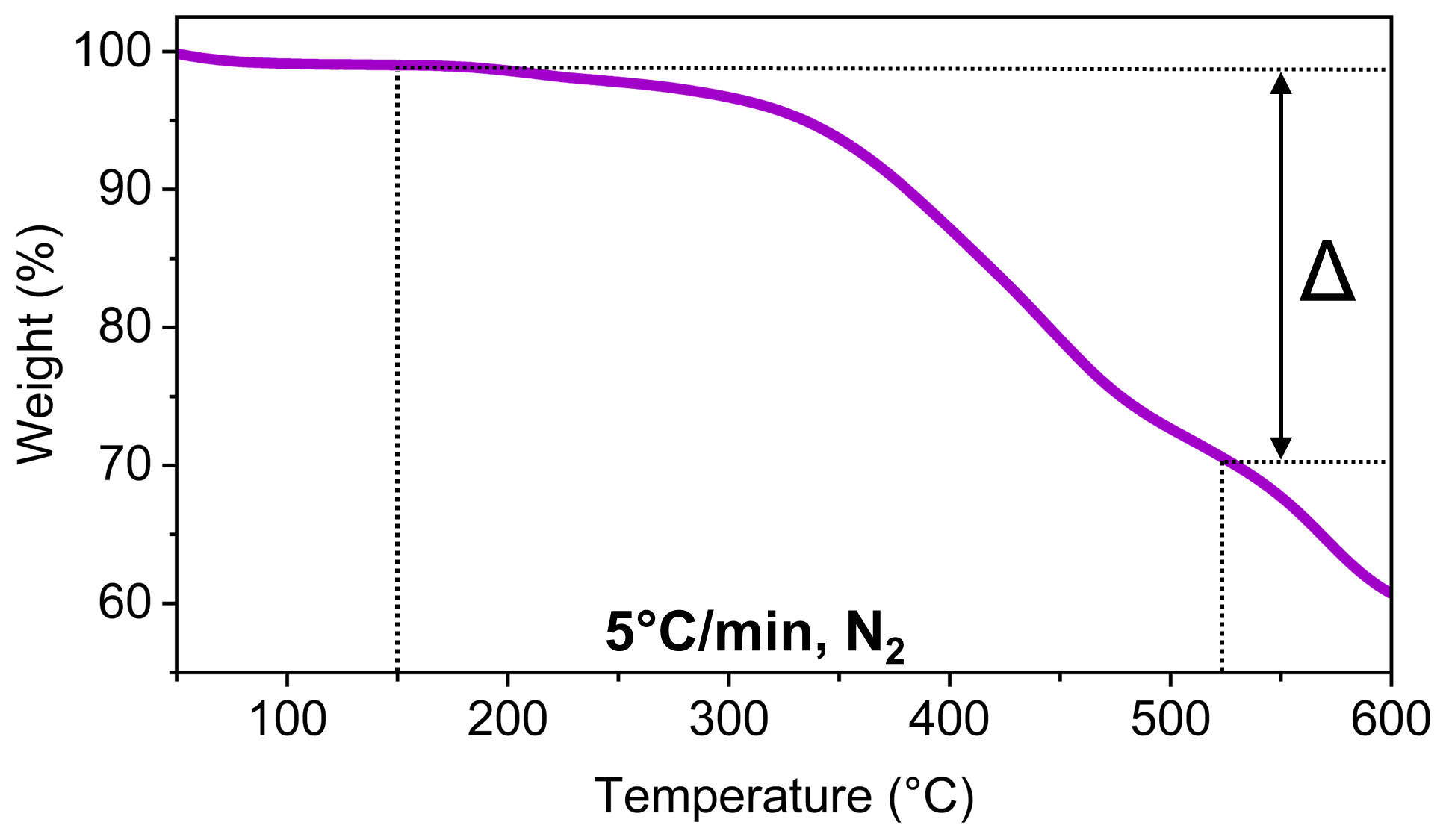


**Figure S37**.Top: thermal analysis of **Trip-COOMe** collected from 50°C to 1000°C. The thermogram was collected under N_2_ flow (50 mL/min) with a heating rate of 5°C/min. Bottom**:** enlargement of the thermogram of **Trip-COOMe** between 50°C and 600°C. The thermogram was collected under N_2_ flow (50 mL/min) with a heating rate of 5°C/min. The normalized weight loss of 28.4 wt% between 150°C and 520°C was used to calculate the amount of methyl ester groups in the framework.


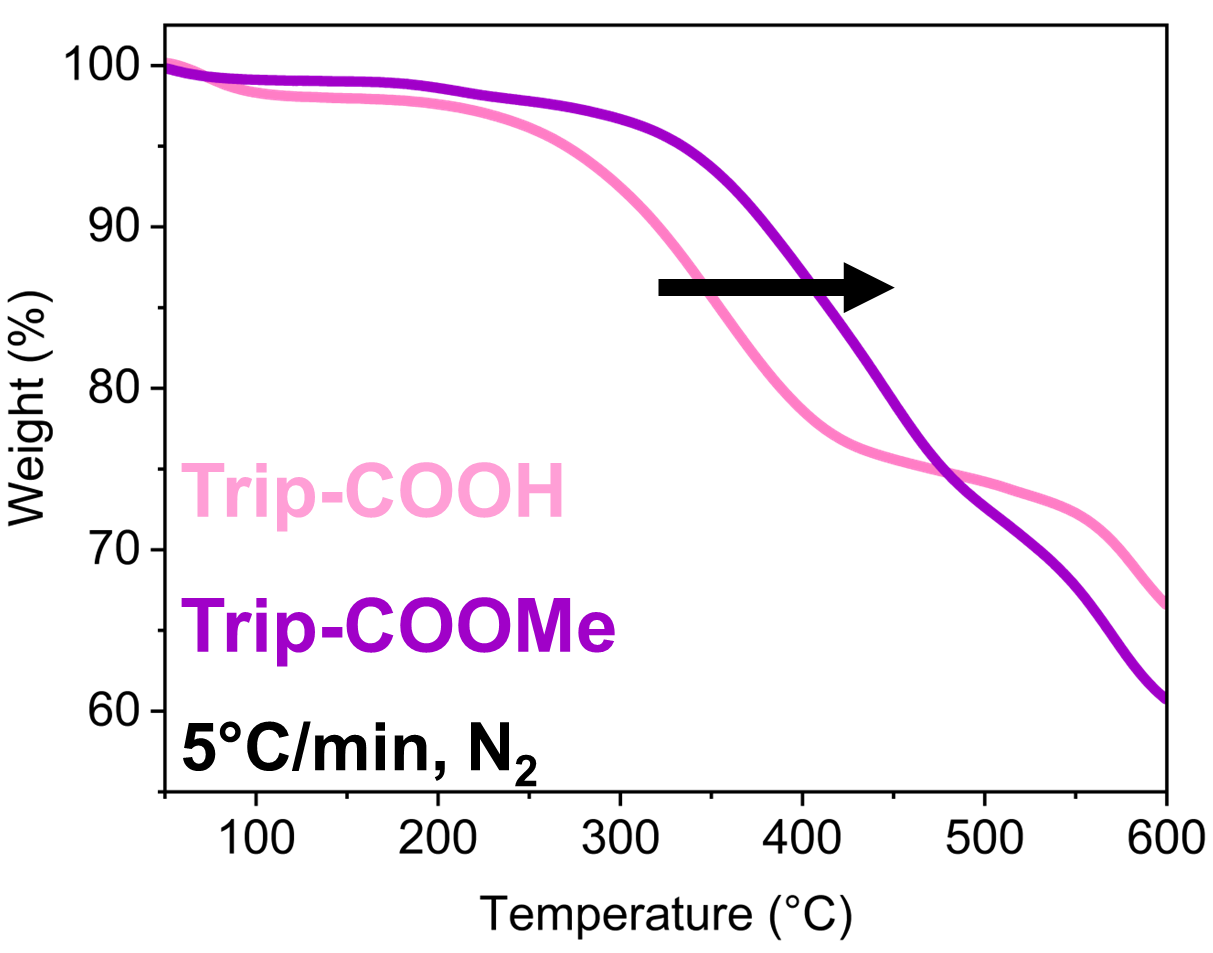


**Figure S38**. Thermal analysis of **Trip-COOH** and **Trip-COOMe**. The thermograms were collected under N_2_ flow (50 mL/min) with a heating rate of 5°C/min. The arrow highlighted the increased thermal stability of sample **Trip-COOMe** compared to **Trip-COOH**.


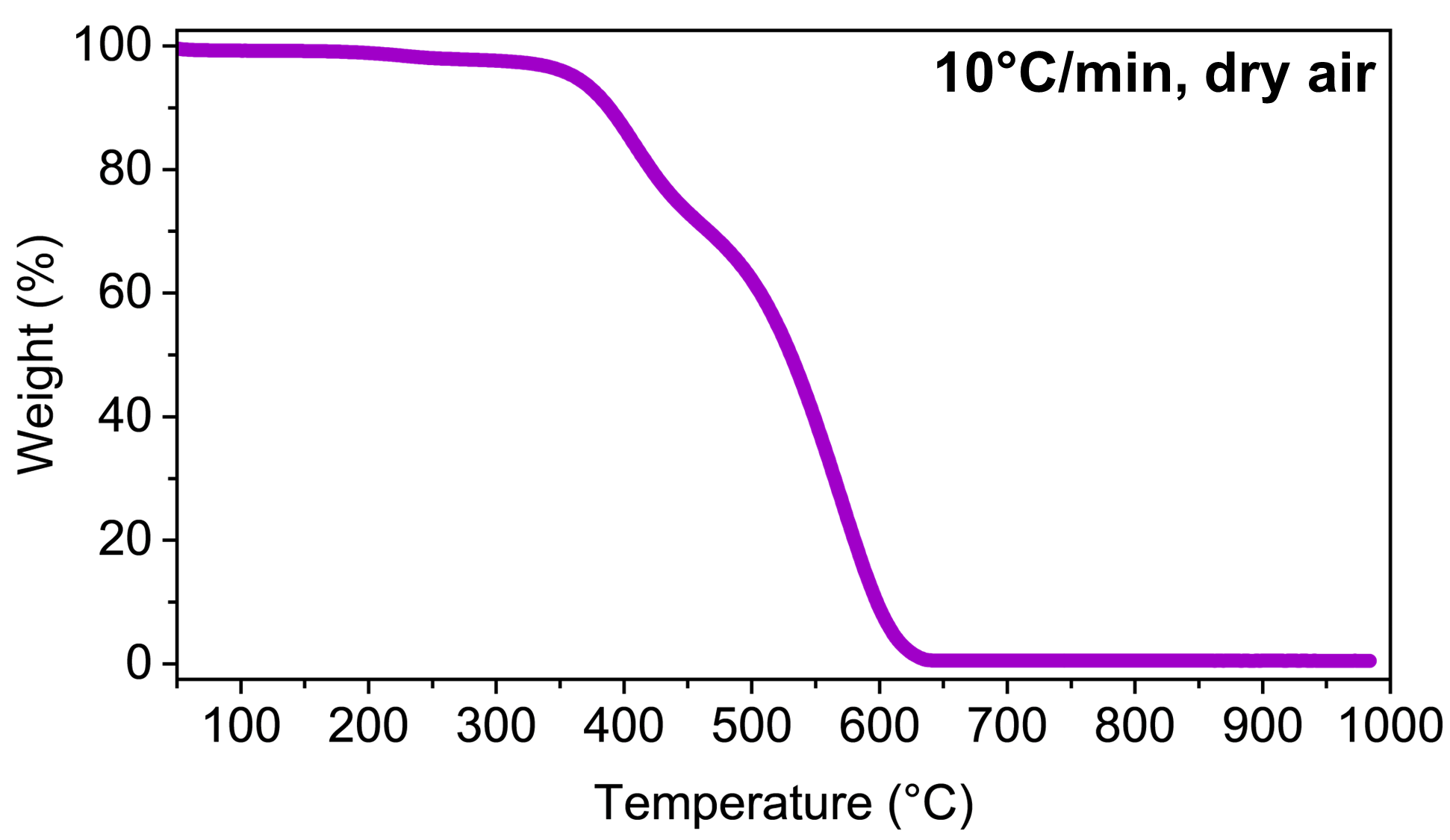


**Figure S39**. Thermal analysis of **Trip-COOMe** collected from 50°C to 1000°C. The thermogram was collected under dry air flow (50 mL/min) with a heating rate of 10°C/min. The residue at 700°C is about 0.3 wt%.

**FT-IR coupled TGA analysis - Trip-COOMe**


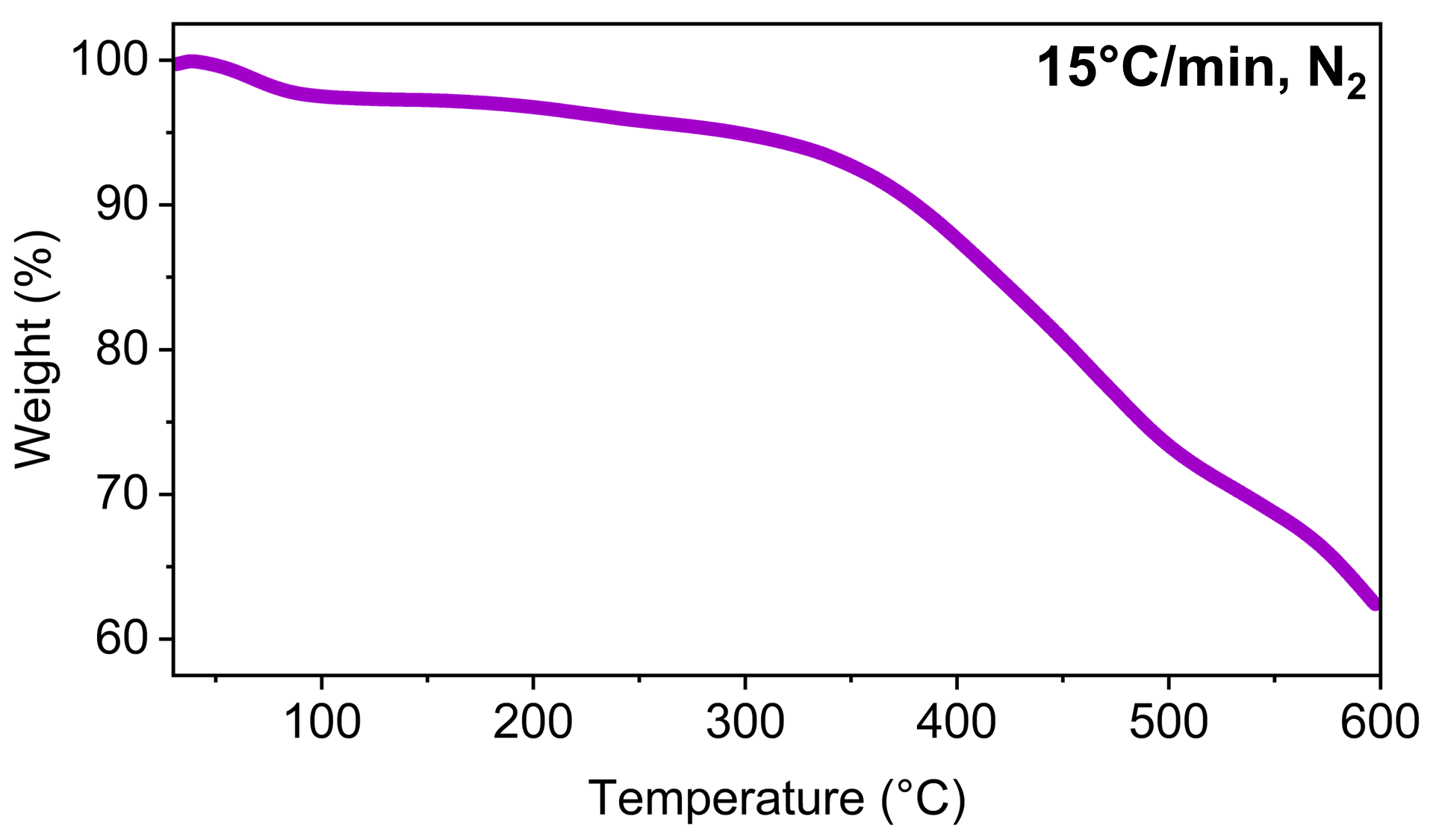


**
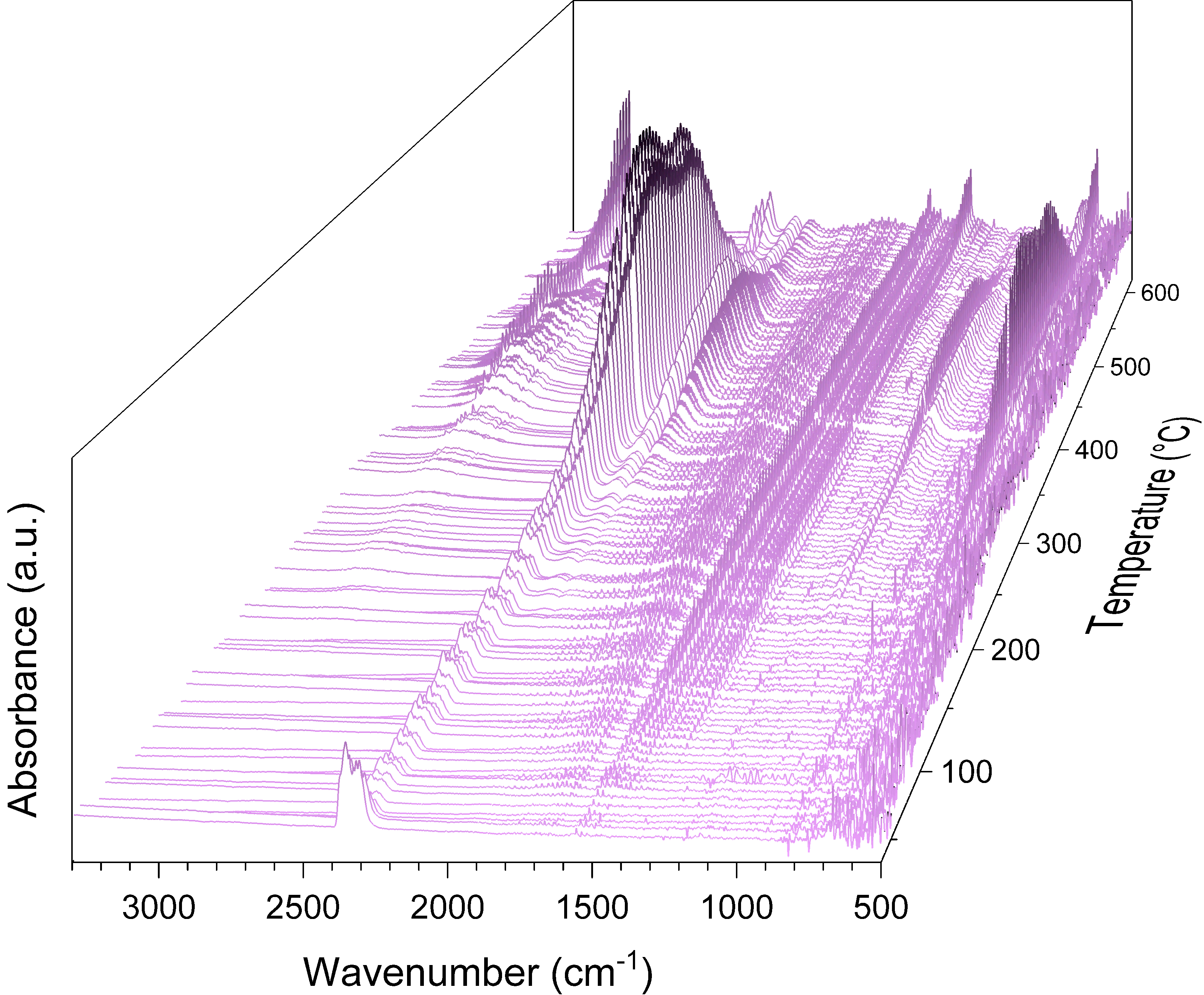
**

**Figure S40**. Top: thermal analysis of **Trip-COOMe** coupled with FT-IR. The thermogram was collected under N_2_ flow (50 mL/min) with a heating rate of 15°C/min. Bottom: 3D plot of TGA-IR data of **Trip-COOMe**. The plot displays the FT-IR spectra as a function of the sample temperature. The strongest bands at higher temperatures are related to the degradation products (see **Figure S40**).

**
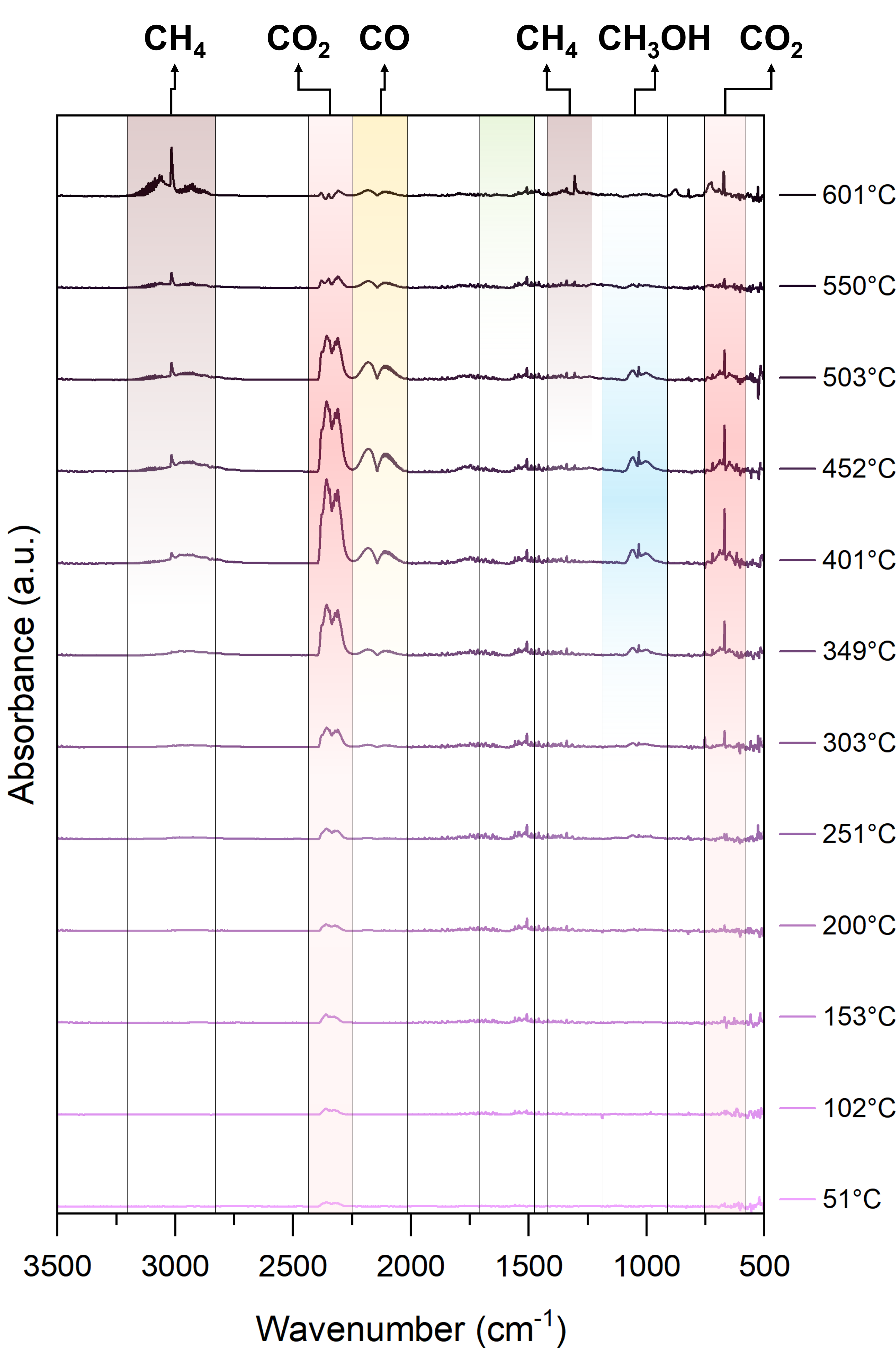
**

**Figure S41.** Selected FT-IR spectra of the evolved gases/vapors collected at different temperatures. The bands of CO_2_ molecules in the temperature range between 50 °C and 150 °C are related to the desorption of guest molecular species from the porous framework. In the temperature range between 300 °C and 500 °C, strong IR-active bands at 668 cm^-1^ and between 2300/2350 cm^-1^ are due to the bending and asymmetric stretching of CO_2_, respectively. Additional bands at 2105/2180 cm^-1^ are related to the vibration of CO, while bands over 3000 cm^-1^ correspond to the vibration of methane and the stretching and bending of the C-H bond. At 1060/1030/1010 cm^-1^ and 2930 cm^-1^ vibration bands of methanol (CH_3_OH) are present.

**^13^C and ^1^H solid-state NMR spectroscopy - Trip-COONa and Trip-COOLi**


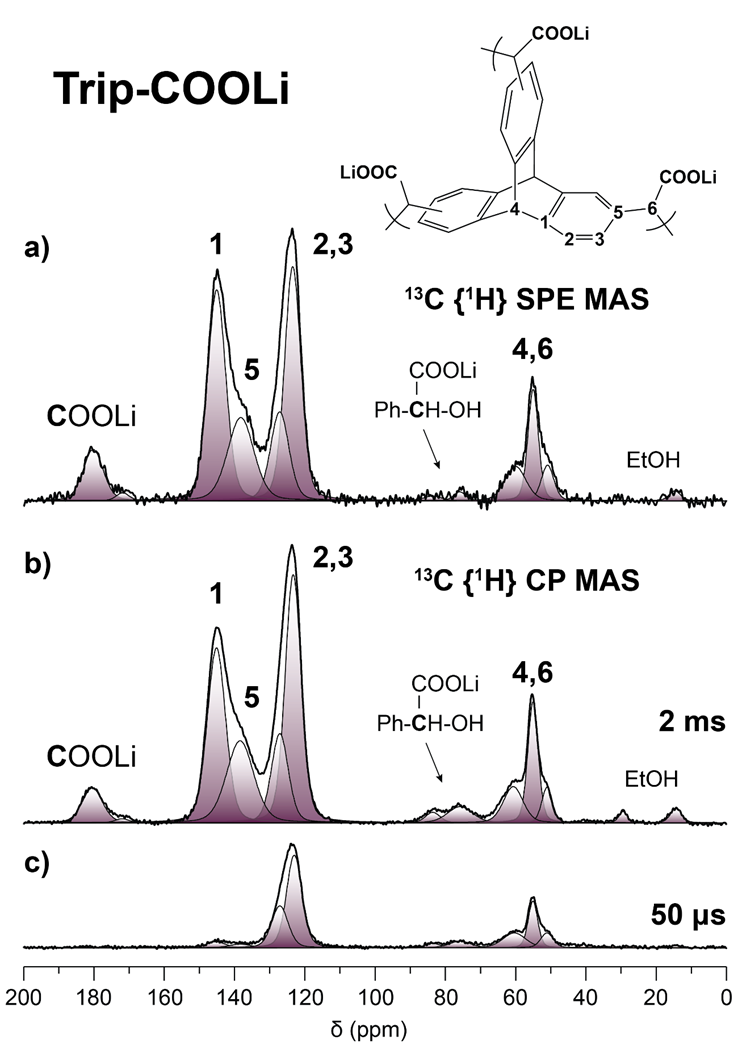


**Figure S42**. ^13^C MAS NMR analysis of **Trip-COOLi** performed at room temperature, 7.04 T, with a spinning speed of 12.5 kHz: a) ^13^C {^1^H} SPE spectrum collected with a recycle delay of 60 s; ^13^C {^1^H} CP spectra collected with a contact time of 2 ms (b) and 0.05 ms (c).

**Table S12**. ^13^C chemical shifts of Trip-COOLi from the simulation of ^13^C {^1^H} SPE MAS spectrum collected at room temperature, 7.04 T, with a spinning speed of 12.5 kHz and a recycle delay of 60 s.

| **Trip-COOLi** | **Assignment** | **δ (ppm)**  **^13^C {^1^H}**  **CP MAS** | **Amount (%)** |
| --- | --- | --- | --- |
| 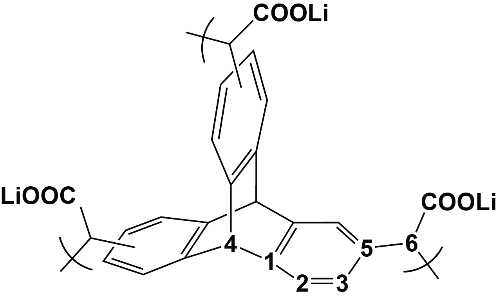 | **C**OOLi | 180.2 | 6.7 |
|  | **C**OOH | 171.8 | 0.7 |
|  | **1** | 145.0 | 75.3 |
|  | **5** | 138.2 |  |
|  | **2, 3** | 127.1, 123.4 |  |
|  | **C**H-OH-COOLi | 83.6, 75.9 | 1.2 |
|  | **6, 4** | 60.5, 55.1, 50.9 | 15.3 |
|  | **C**H_3_-CH_2_OH | 14.7 | 0.7 |

**
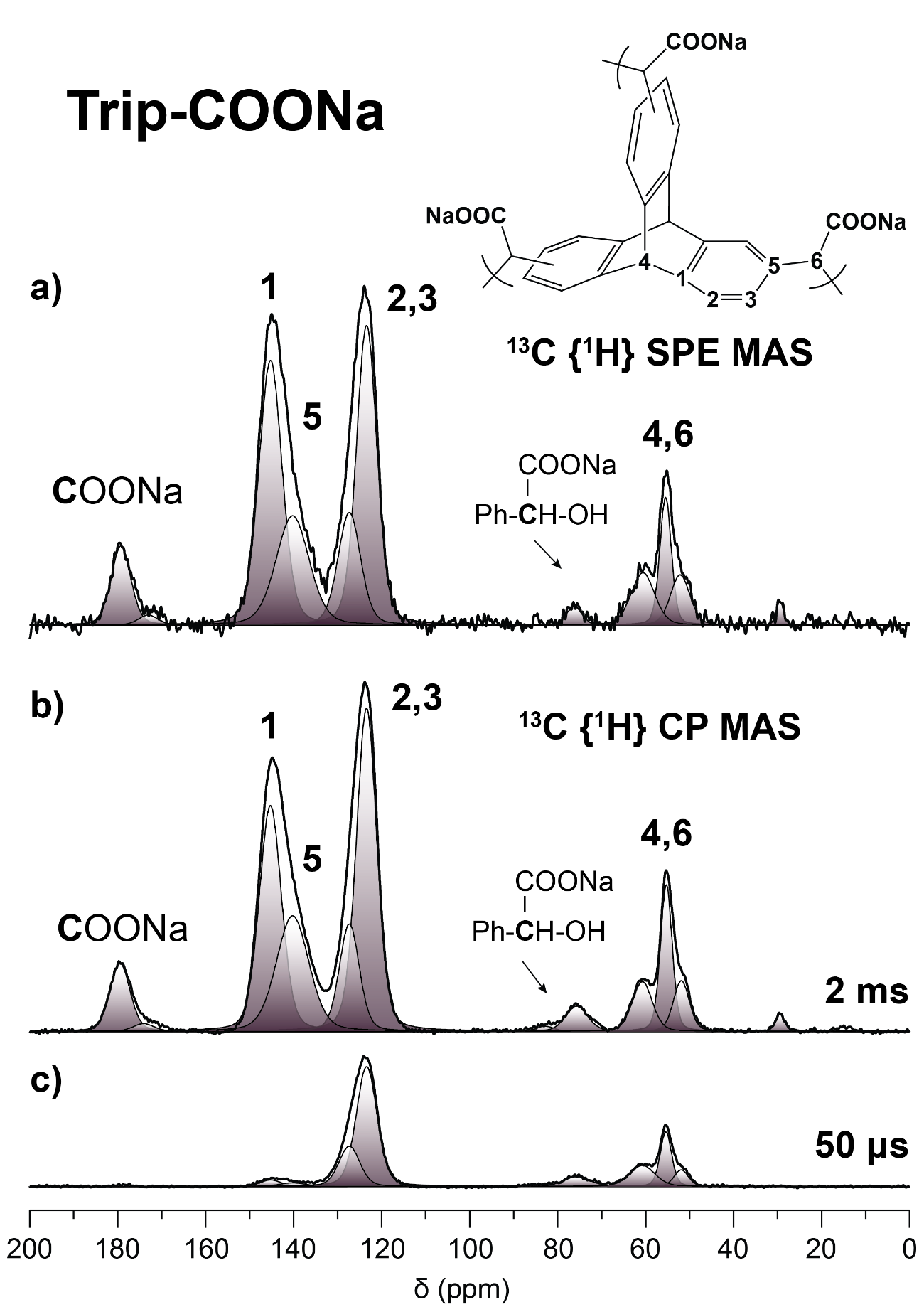
**

**Figure S43**. ^13^C MAS NMR analysis of **Trip-COONa** performed at room temperature, 7.04 T, with a spinning speed of 12.5 kHz: a) quantitative ^13^C {^1^H} SPE spectrum collected with a recycle delay of 60 s; ^13^C {^1^H} CP spectra collected with a contact time of 2 ms (b) and 0.05 ms (c).

**Table S13**. ^13^C chemical shifts of **Trip-COONa** from the simulation of ^13^C {^1^H} SPE MAS spectrum collected at room temperature, 7.04 T, with a spinning speed of 12.5 kHz and a recycle delay of 60 s.

| **Trip-COONa** | **Assignment** | **δ (ppm)**  **^13^C {^1^H}**  **SPE MAS** | **Amount (%)** |
| --- | --- | --- | --- |
| 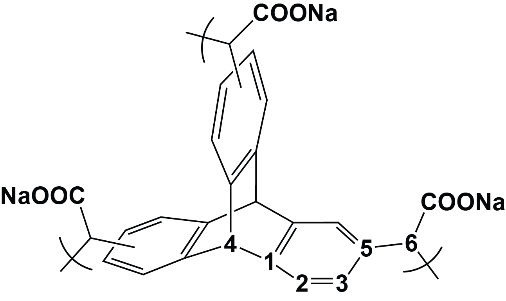 | **C**OONa | 179.2 | 6.4 |
|  | **C**OOH | 173.0 | 0.8 |
|  | **1** | 145.2 | 75.7 |
|  | **5** | 140.2 |  |
|  | **2, 3** | 127.3, 123.4 |  |
|  | **C**H-OH-COONa | 76.2 | 1.2 |
|  | **6, 4** | 60.6, 55.4, 52.1 | 15.2 |
|  | **C**H_3_-CH_2_OH | 29.4 | 0.7 |

**
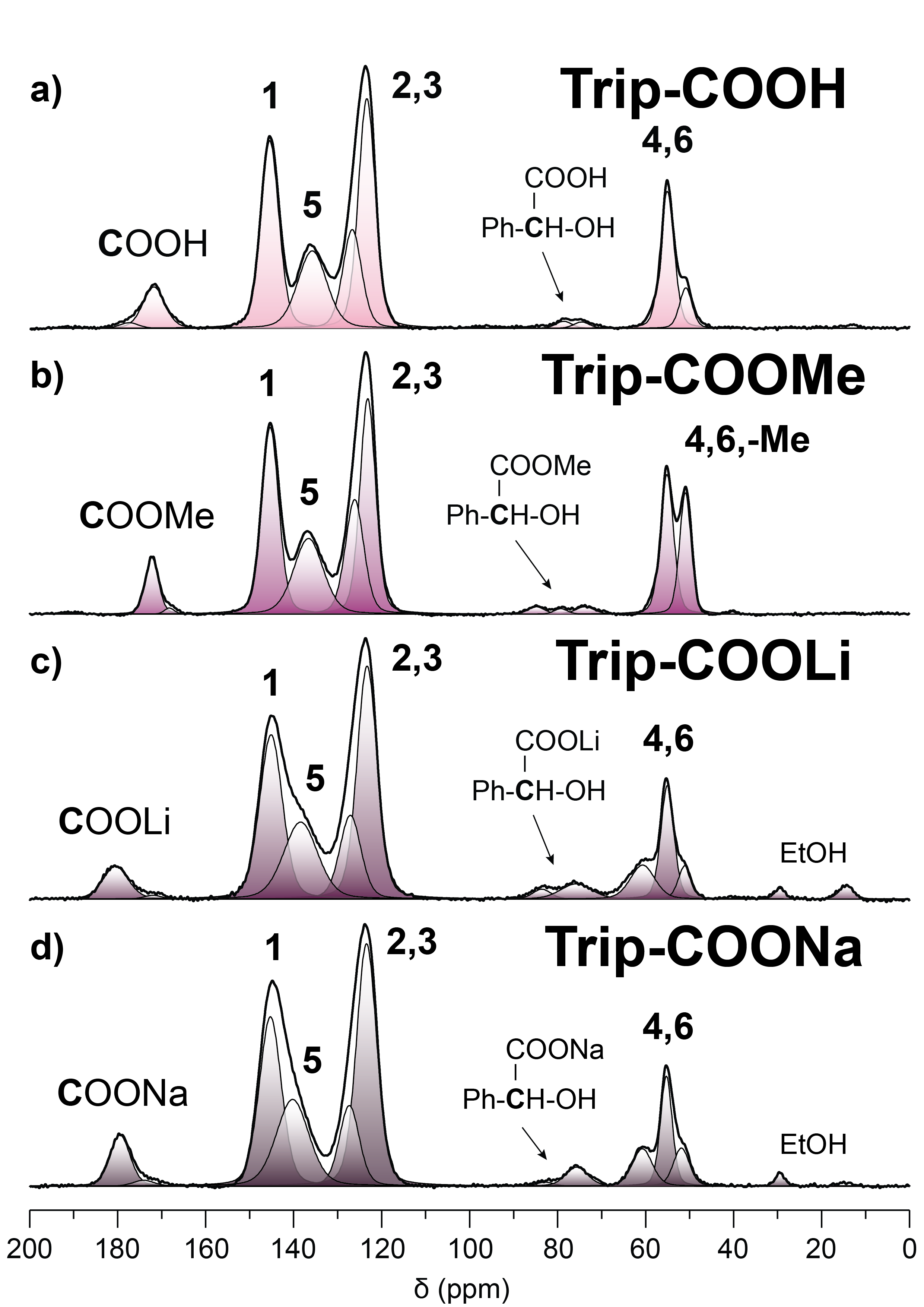
**

**Figure S44**. ^13^C {^1^H} CP MAS spectra collected at room temperature, 7.04 T, with a spinning speed of 12.5 kHz and a contact time of 2 ms of a) **Trip -COOH**, b) **Trip-COOMe**, c) **Trip-COOLi**, d) **Trip-COONa**.

**Thermal analysis - Trip-COONa and Trip-COOLi**


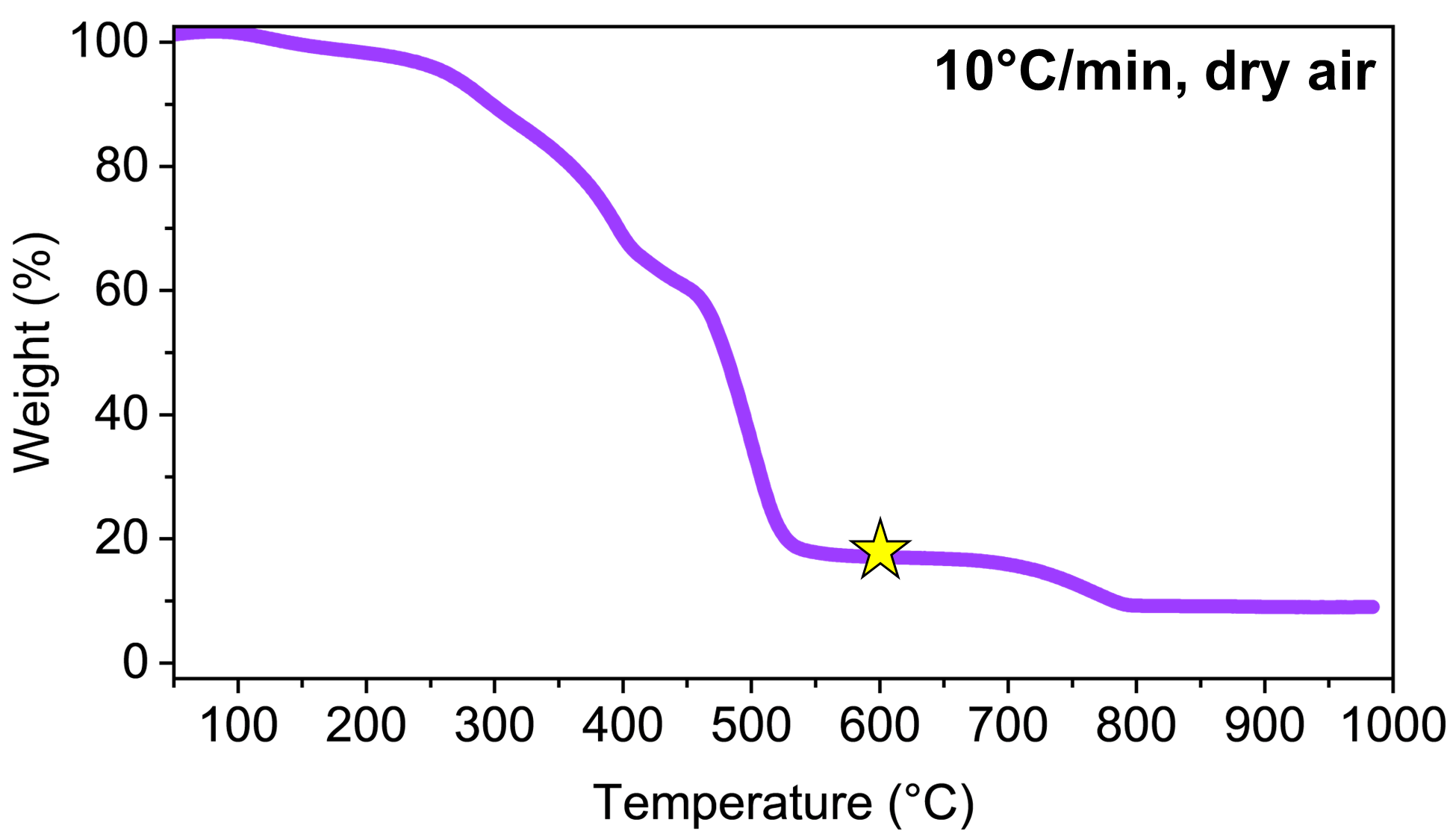


**Figure S45**. Thermal analysis of **Trip-COOLi** collected from 50°C to 1000°C. The thermogram was collected under dry air flow (50 mL/min) with a heating rate of 10°C/min. The residue at 600°C (yellow star) is Li_2_CO_3_, as demonstrated by collecting the PXRD of the white residue of the sample treated at 600°C, which is about 16.9 wt%. This value was used to calculate the percentage of lithium carboxylate groups in the framework.


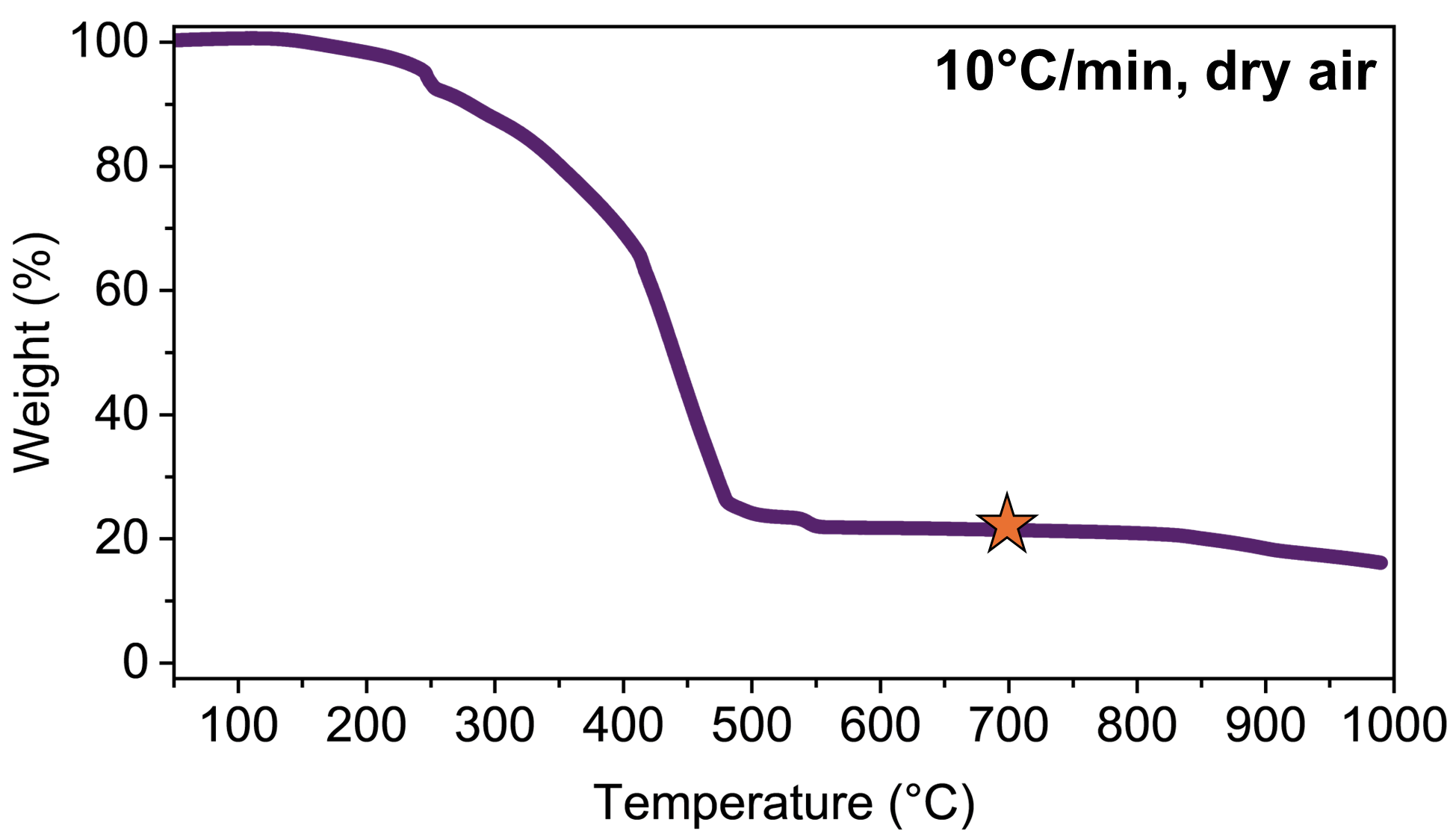


**Figure S46**. Thermal analysis of **Trip-COONa** collected from 50°C to 1000°C. The thermogram was collected under dry air flow (50 mL/min) with a heating rate of 10°C/min. The residue at 700°C (orange star) is Na_2_CO_3_, as demonstrated by collecting the PXRD of the residue of a sample treated at 700°C, and it is about 23.1 wt%. This value was used to calculate the percentage of sodium carboxylate groups in the framework.

**Infrared spectroscopy**

**
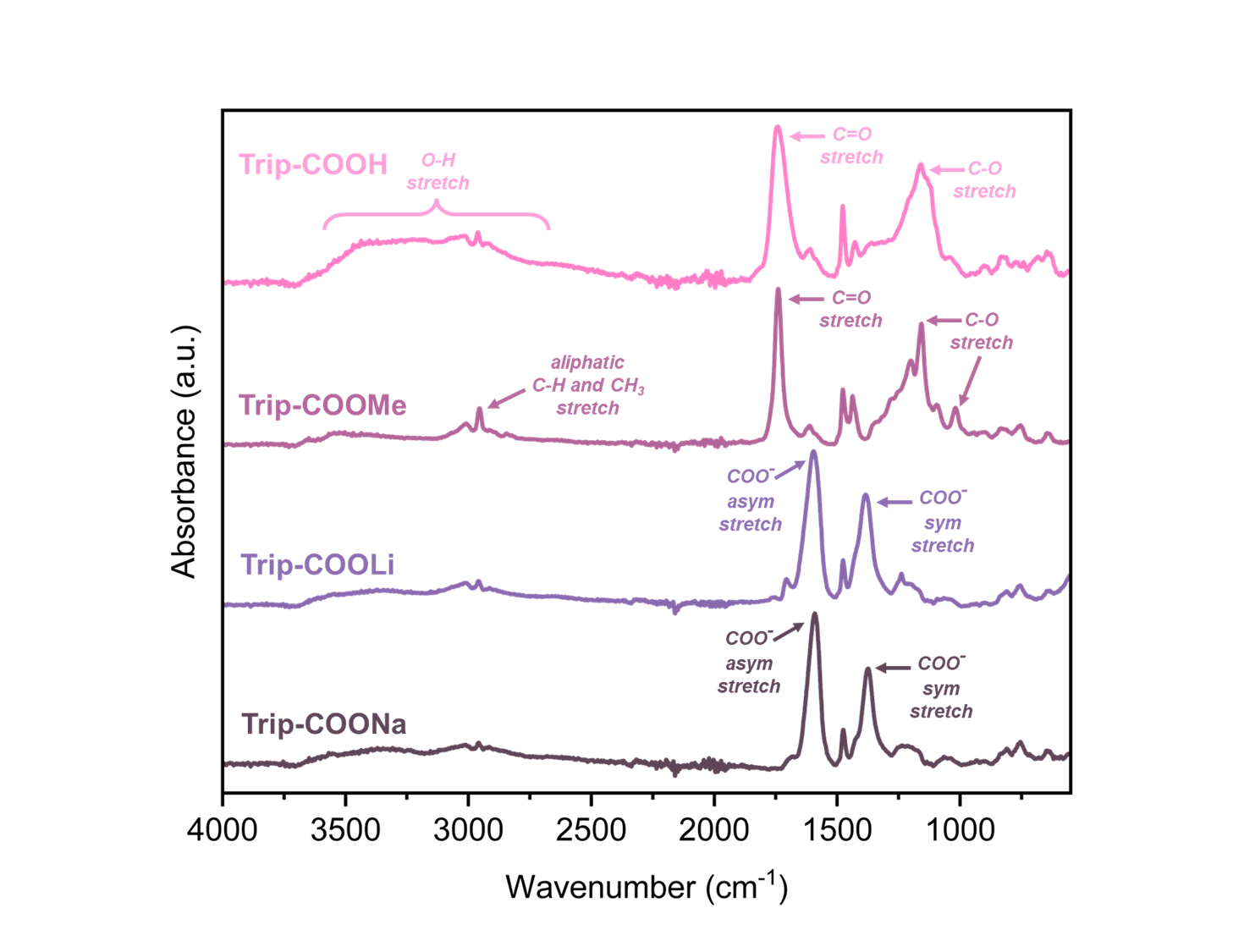
**

**Figure S47**. FT-IR spectra of **Trip-COOH** (top, pink), **Trip-COOMe** (purple), **Trip-COOLi** (violet) and **Trip-COONa** (bottom, dark violet) collected from 525 cm^-1^ to 4000 cm^-1^. The characteristic vibrational bands are highlighted in the figures.

**Gas adsorption measurements**

**
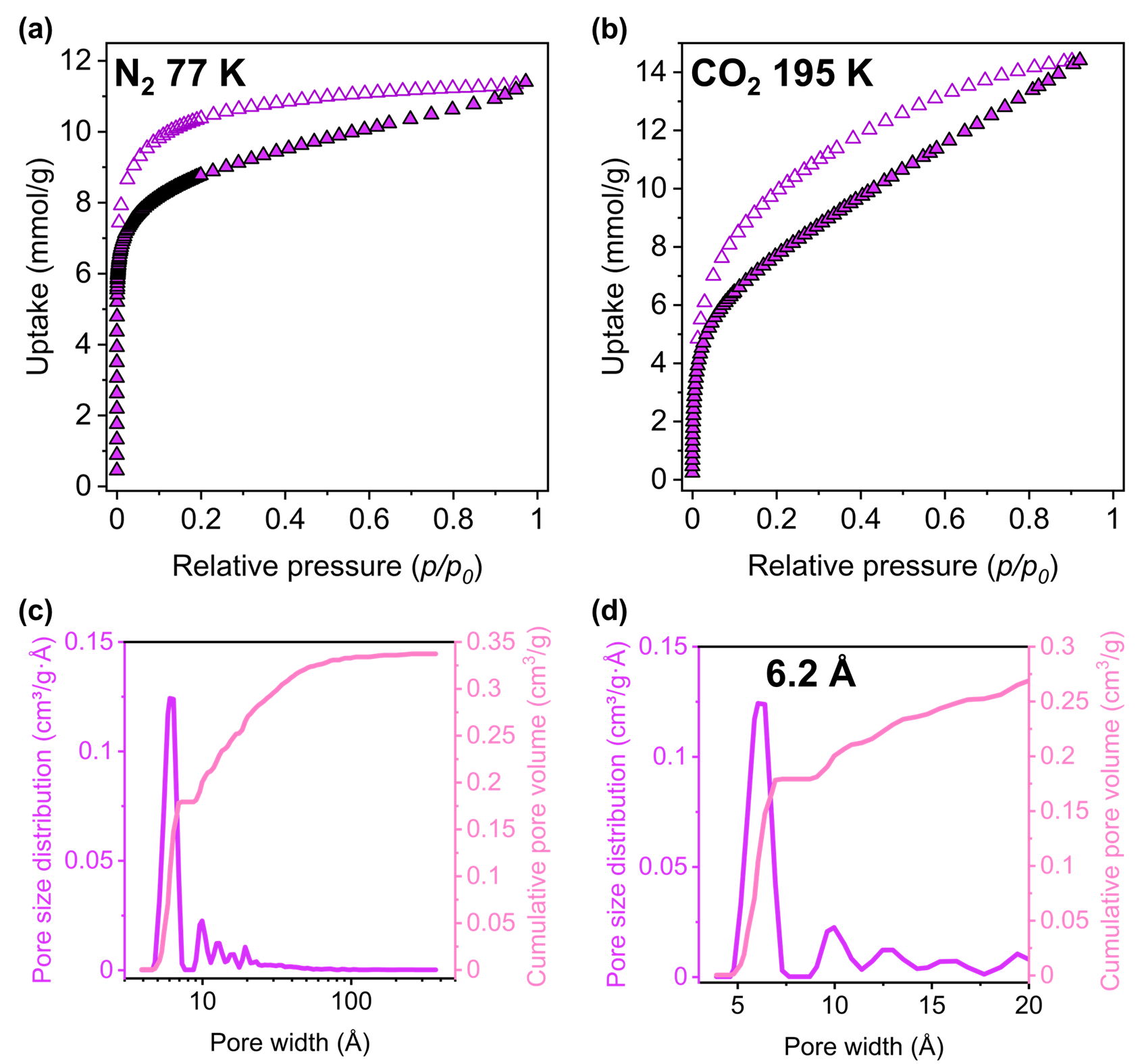
**

**Figure S48**. (a) N_2_ adsorption isotherm collected at 77 K and (b) CO_2_ adsorption isotherm collected at 195 K of **Trip-COOMe**. Filled and empty symbols represent sorption and desorption branches, respectively. (c) Differential pore size distribution and cumulative pore size distribution calculated from N_2_ adsorption isotherm at 77 K according to NLDFT theory and HS-2D-NLDFT, Carbon, N2, 77 K pore model. (d) Enlargement between 3 Å and 20 Å of the differential and cumulative pore size distribution highlights the ultramicroporous nature of **Trip-COOMe**.


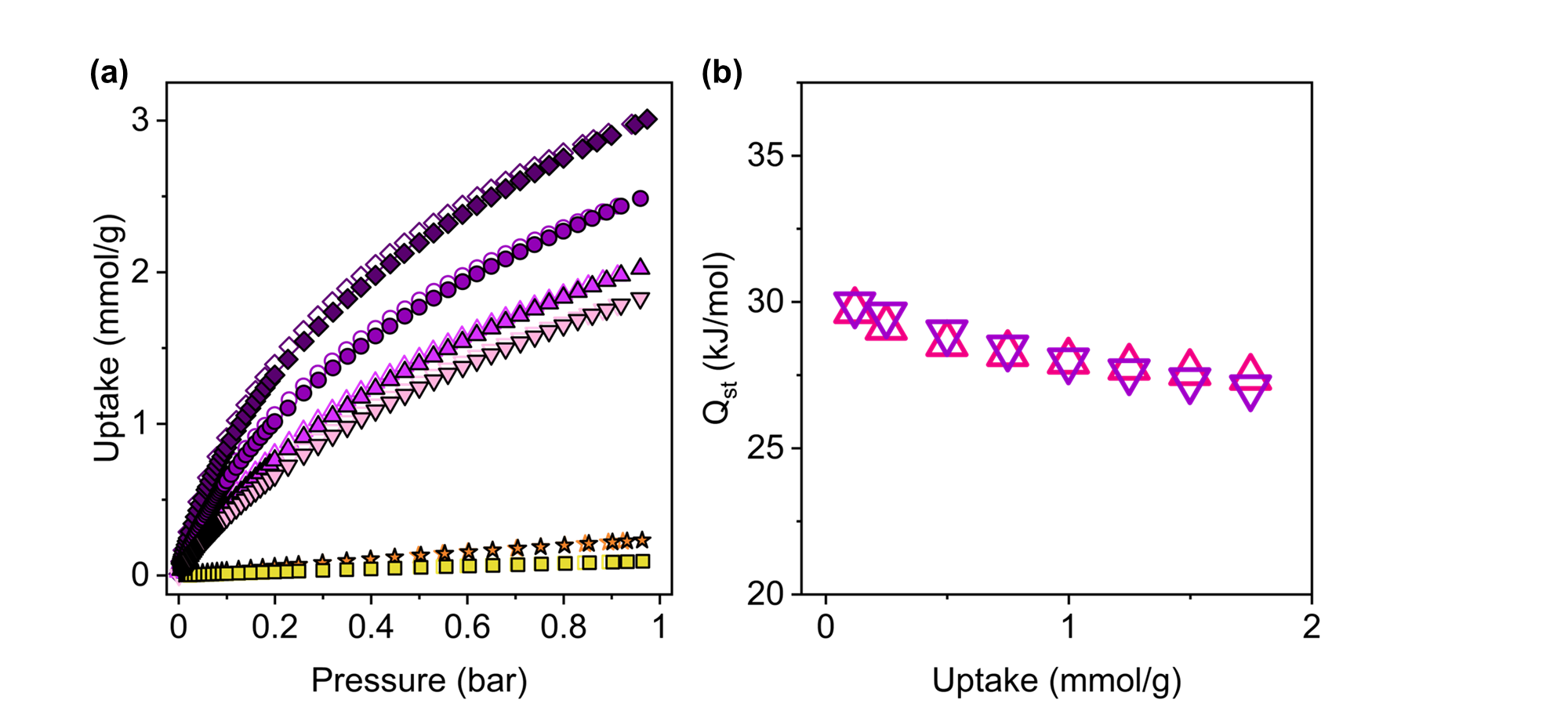


**Figure S49**. (a) CO_2_ adsorption isotherms of sample **Trip-COOMe** collected at 273 K (diamonds, dark purple), 283 K (circles, purple), 293 K (up-pointing triangles, light-purple) and 298 K (down-pointing triangles, pink). Filled and empty symbols represent sorption and desorption branches, respectively. N_2_ adsorption isotherms of sample **Trip-COOMe** collected at 273 K (stars, orange) and 298 K (squares, yellow). (b) Isosteric heat of adsorption for CO_2_ calculated from the isotherms collected at different temperatures using the virial method (down-pointing triangles, violet) and the Van’t Hoff method after fitting with Langmuir-Freundlich model (up-pointing triangles, purple).


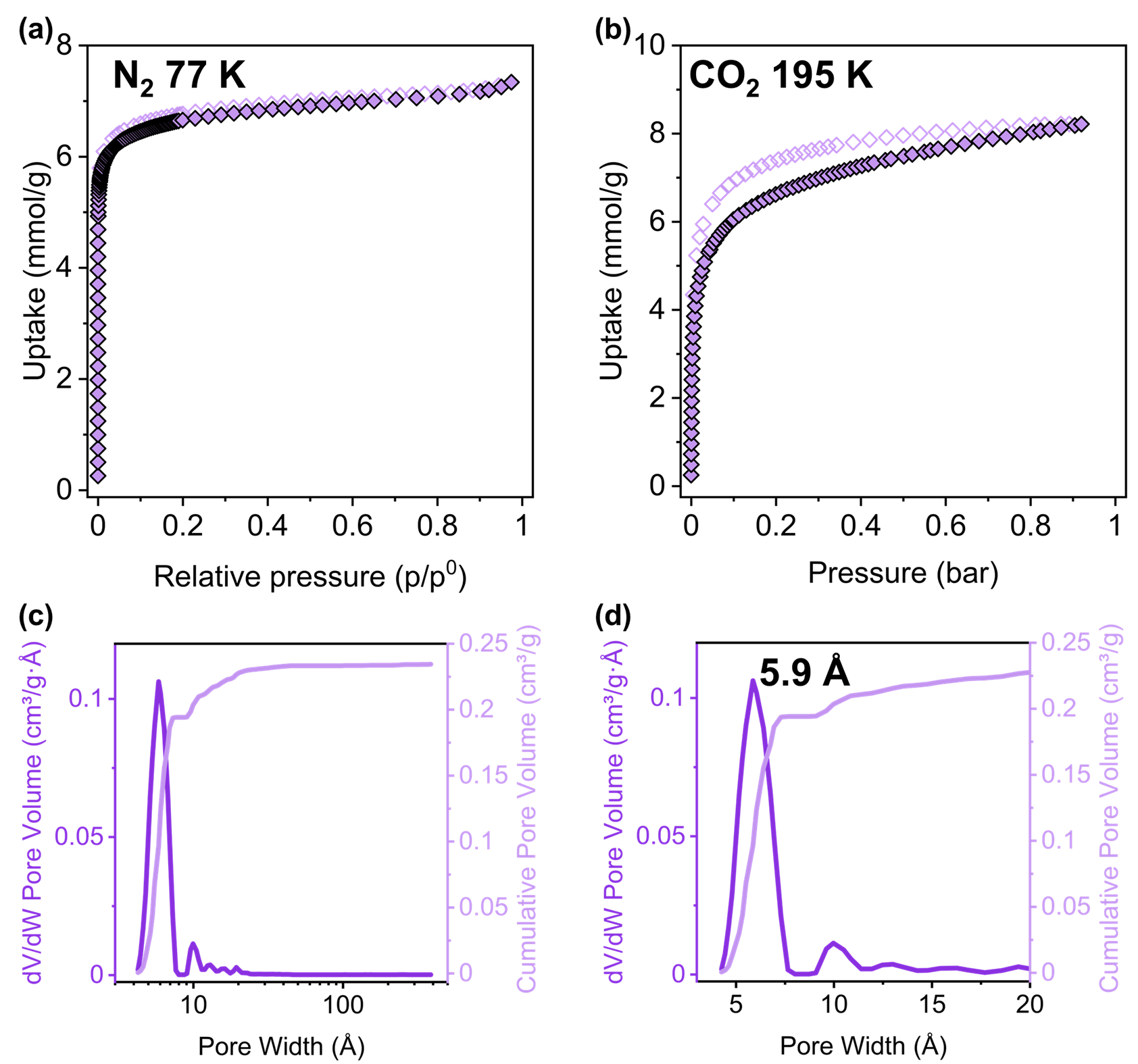


**Figure S50**. (a) N_2_ adsorption isotherm collected at 77 K and (b) CO_2_ adsorption isotherm collected at 195 K of **Trip-COOLi**. Filled and empty symbols represent sorption and desorption branches, respectively. (c) Differential pore size distribution and cumulative pore size distribution calculated from N_2_ adsorption isotherm at 77 K according to NLDFT theory and HS-2D-NLDFT, Carbon, N_2_, 77 K pore model. (d) Enlargement between 3 Å and 20 Å of the differential and cumulative pore size distribution highlights the ultramicroporous nature of **Trip-COOLi**.


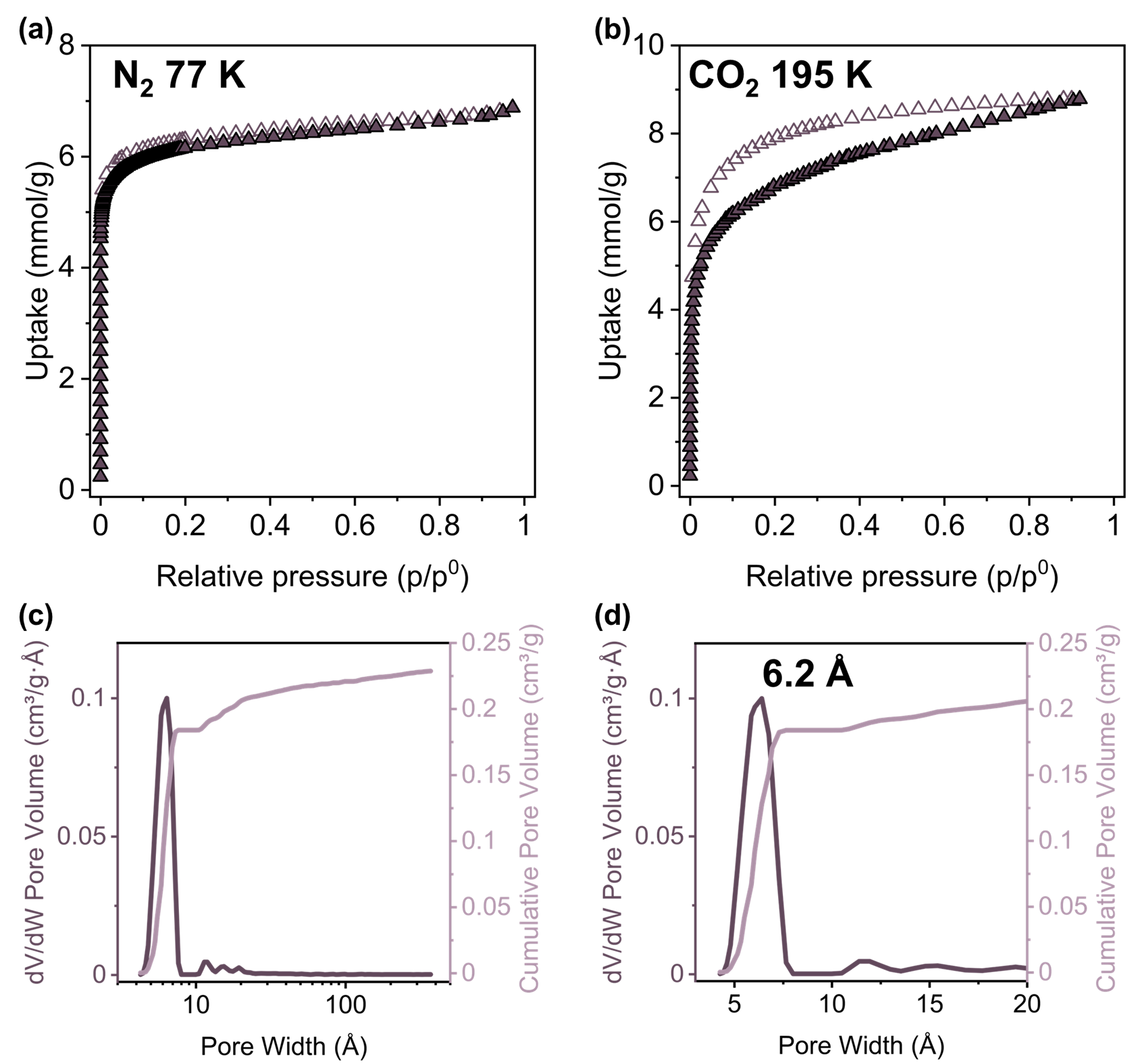


**Figure S51**. (a) N_2_ adsorption isotherm collected at 77 K and (b) CO_2_ adsorption isotherm collected at 195 K of **Trip-COONa**. Filled and empty symbols represent sorption and desorption branches, respectively. (c) Differential pore size distribution and cumulative pore size distribution calculated from N_2_ adsorption isotherm at 77 K according to NLDFT theory and HS-2D-NLDFT, Carbon, N2, 77 K pore model. (d) Enlargement between 3 Å and 20 Å of the differential and cumulative pore size distribution highlights the ultramicroporous nature of **Trip-COONa**.

**Table S14**. Textural properties measured and calculated from cryogenic N_2_ sorption (77 K) and CO_2_ sorption isotherms collected at 273 K, 283 K, 293 K and 298 K of **Trip-COOH**, **Trip-COONa**, **Trip-COOLi** and **Trip-COOMe** samples.

| Sample | Langmuir surface area (m^2^/g)^1^ | BET surface area (m^2^/g)^1^ | Pore volume (cm^3^/g)^2^ | Micropore volume (cm^3^/g)^2^ | CO_2_ uptake at 1 bar, 273 K (mmol/g) | CO_2_ uptake at 1 bar, 298 K (mmol/g) | Q_st_ at 0.1 mmol/g (kJ/mol) ^3^ |
| --- | --- | --- | --- | --- | --- | --- | --- |
| Trip-COOH | 890 | 822 | 0.35 | 0.30 | 4.12 | 2.67 | 32.0 |
| Trip-COONa | 575 | 540 | 0.23 | 0.21 | 3.76 | 2.77 | 49 |
| Trip-COOLi | 626 | 585 | 0.23 | 0.23 | 3.28 | 2.51 | 47 |
| Trip-COOMe | 795 | 733 | 0.34 | 0.27 | 3.01 | 1.83 | 29.7 |

^1^ BET surface areas were calculated in the range 0.015<*p/p_0_*<0.06 according to the Rouquerol analysis. Langmuir surface areas were calculated in the range 0.015<*p/p_0_*<0.06. ^2^ Total and micropore volumes were calculated according to NLDFT theory and HS-2D-NLDFT Carbon, N_2_, 77 K pore model. Micropore volume was evaluated in the pore size range between 0 Å and 20 Å.^3^ The isosteric heats of adsorption were calculated using the virial method, using the CO_2_ sorption isotherms collected at 273 K, 283 K, 293 K and 298 K.


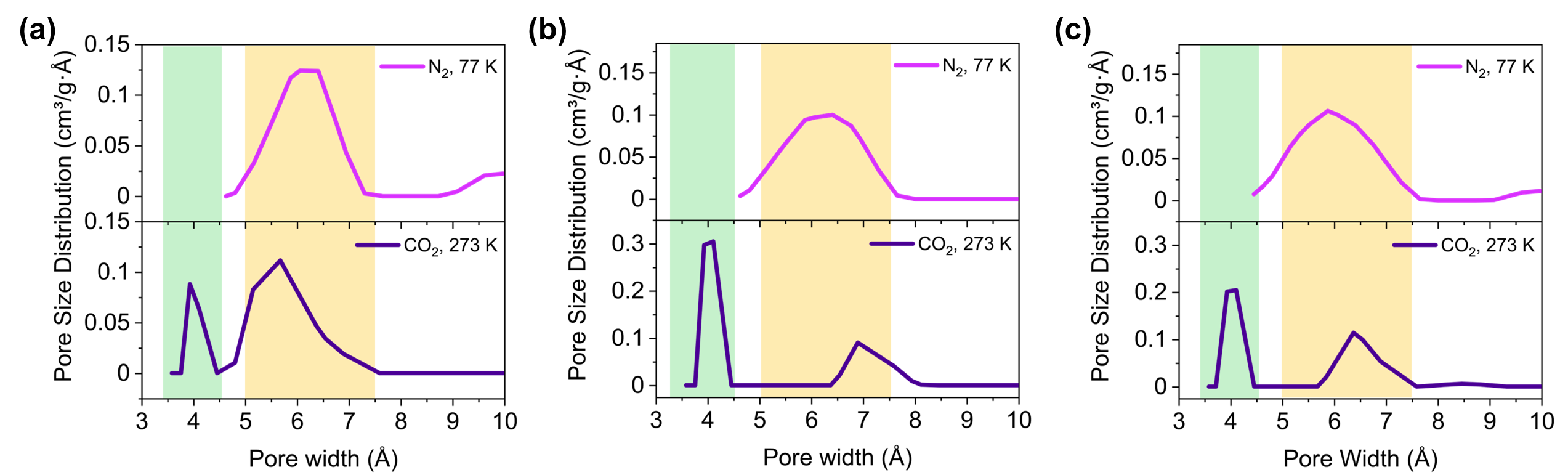


**Figure S52**. (a) Top: pore size distribution of **Trip-COOMe** calculated from N_2_ adsorption isotherm at 77 K according to the HS 2D-NLDFT theory and carbon slit pore model for nitrogen adsorption at 77 K (purple). Bottom: pore size distribution of **Trip-COOMe** calculated from CO_2_ adsorption isotherm at 273 K according to the HS 2D-NLDFT theory and carbon slit pore model for CO_2_ adsorption at 273 K (violet). (b) Top: pore size distribution of **Trip-COONa** calculated from N_2_ adsorption isotherm at 77 K according to the HS 2D-NLDFT theory and carbon slit pore model for nitrogen adsorption at 77 K (purple). Bottom: pore size distribution of **Trip-COONa** calculated from CO_2_ adsorption isotherm at 273 K according to the HS 2D-NLDFT theory and carbon slit pore model for CO_2_ adsorption at 273 K (violet). (c) Top: pore size distribution of **Trip-COOLi** calculated from N_2_ adsorption isotherm at 77 K according to the HS 2D-NLDFT theory and carbon slit pore model for nitrogen adsorption at 77 K (purple). Bottom: pore size distribution of **Trip-COOLi** calculated from CO_2_ adsorption isotherm at 273 K according to the HS 2D-NLDFT theory and carbon slit pore model for CO_2_ adsorption at 273 K (violet).

**Powder X-ray diffraction**

**
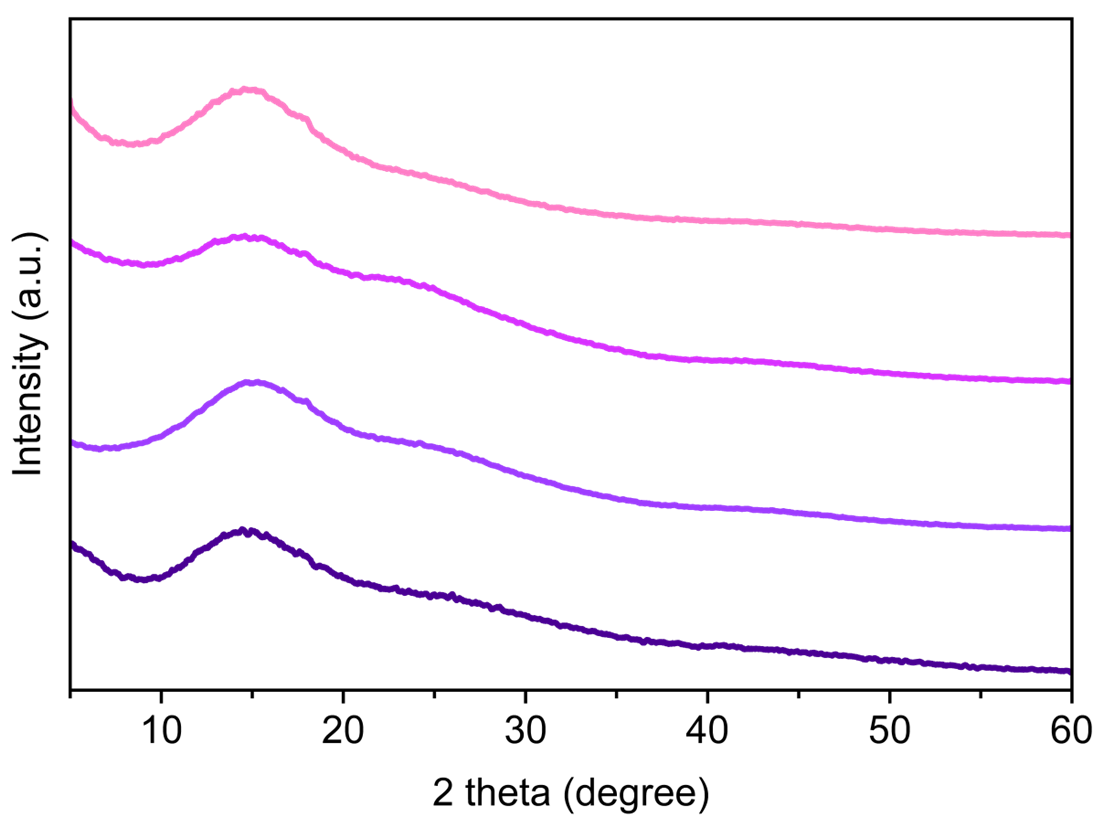
**

**Figure S53**. Powder X-ray diffraction patterns of sample **Trip-COOH** (top, pink), **Trip-COOMe** (purple), **Trip-COOLi** (violet) and **Trip-COONa** (bottom, dark violet).

**
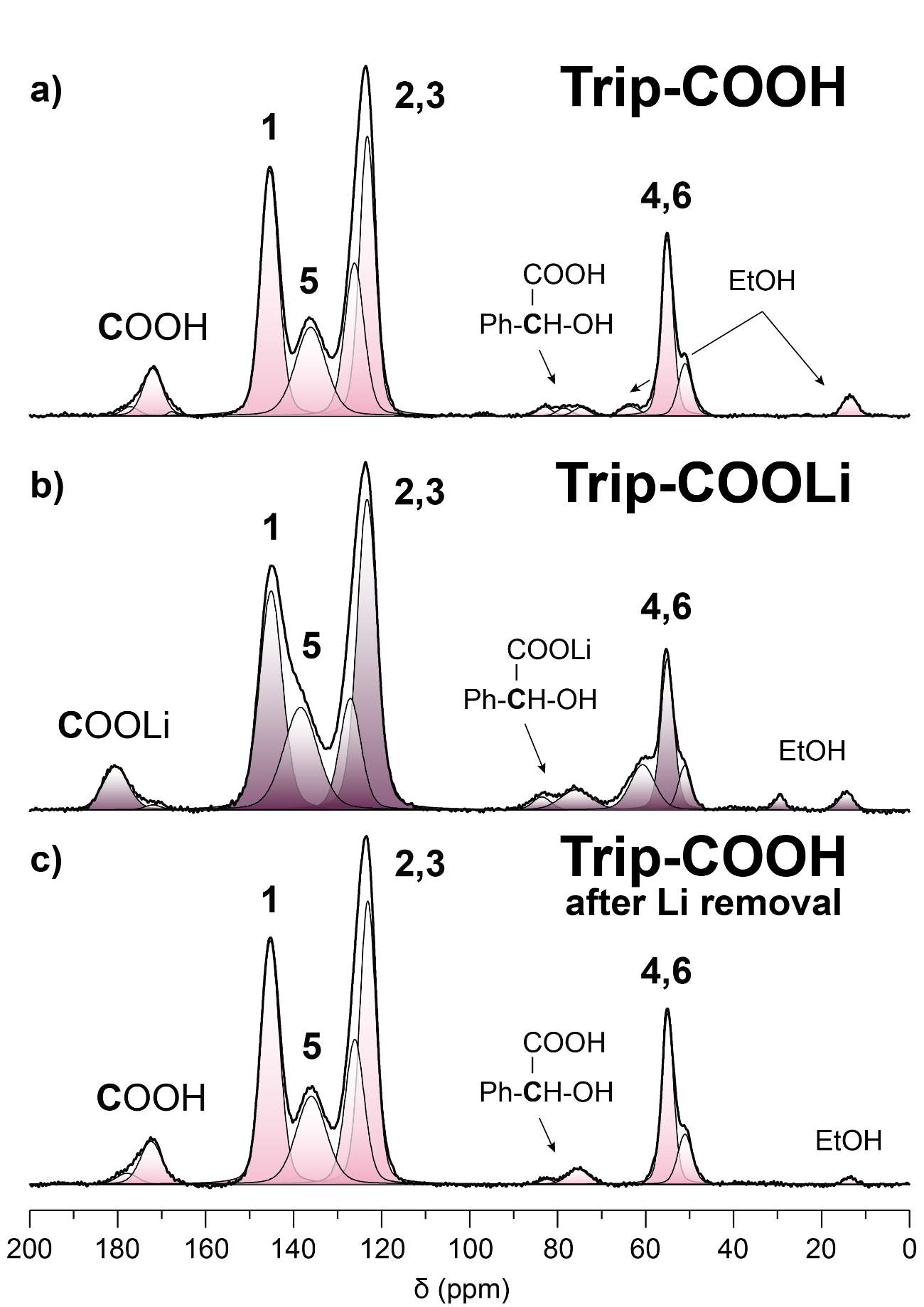
**

**Figure S54**. ^13^C {^1^H} CP MAS spectra collected at room temperature, 7.04 T, with a spinning speed of 12.5 kHz and a contact time of 2 ms of a) **Trip-COOH**, b) **Trip-COOLi**, c) **Trip-COOH** after Li removal.

**
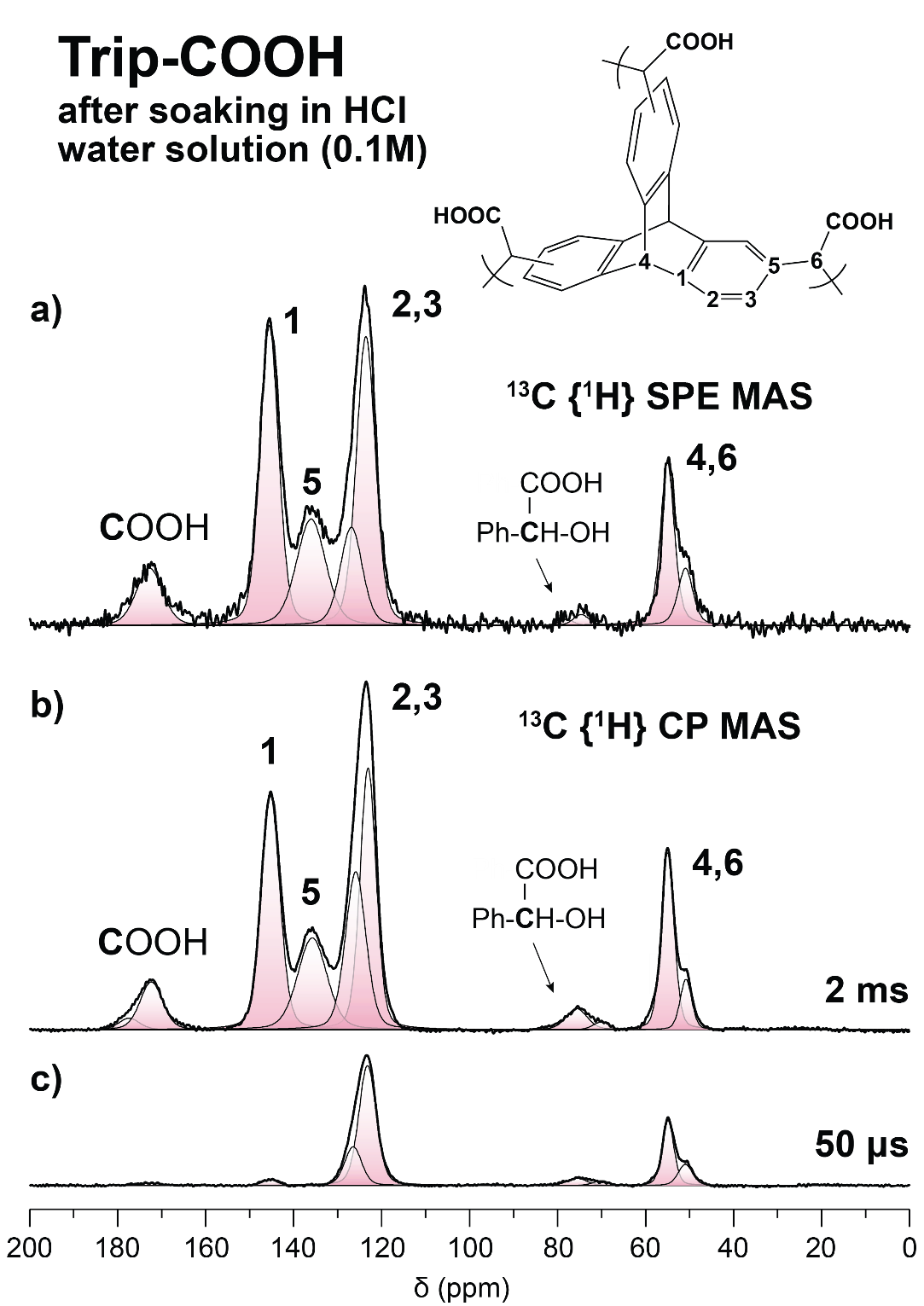
**

**Figure S55.** ^13^C MAS NMR analysis performed at 298 K, 7.04 T, with a spinning speed of 12.5 kHz of **Trip-COOH** after soaking in HCl solution (0.1 M) for 10 days at room temperature: a) quantitative ^13^C {^1^H} SPE spectrum collected with a recycle delay of 60 s; ^13^C {^1^H} CP spectra collected with a contact time of 2 ms (b) and 0.05 ms (c).

**
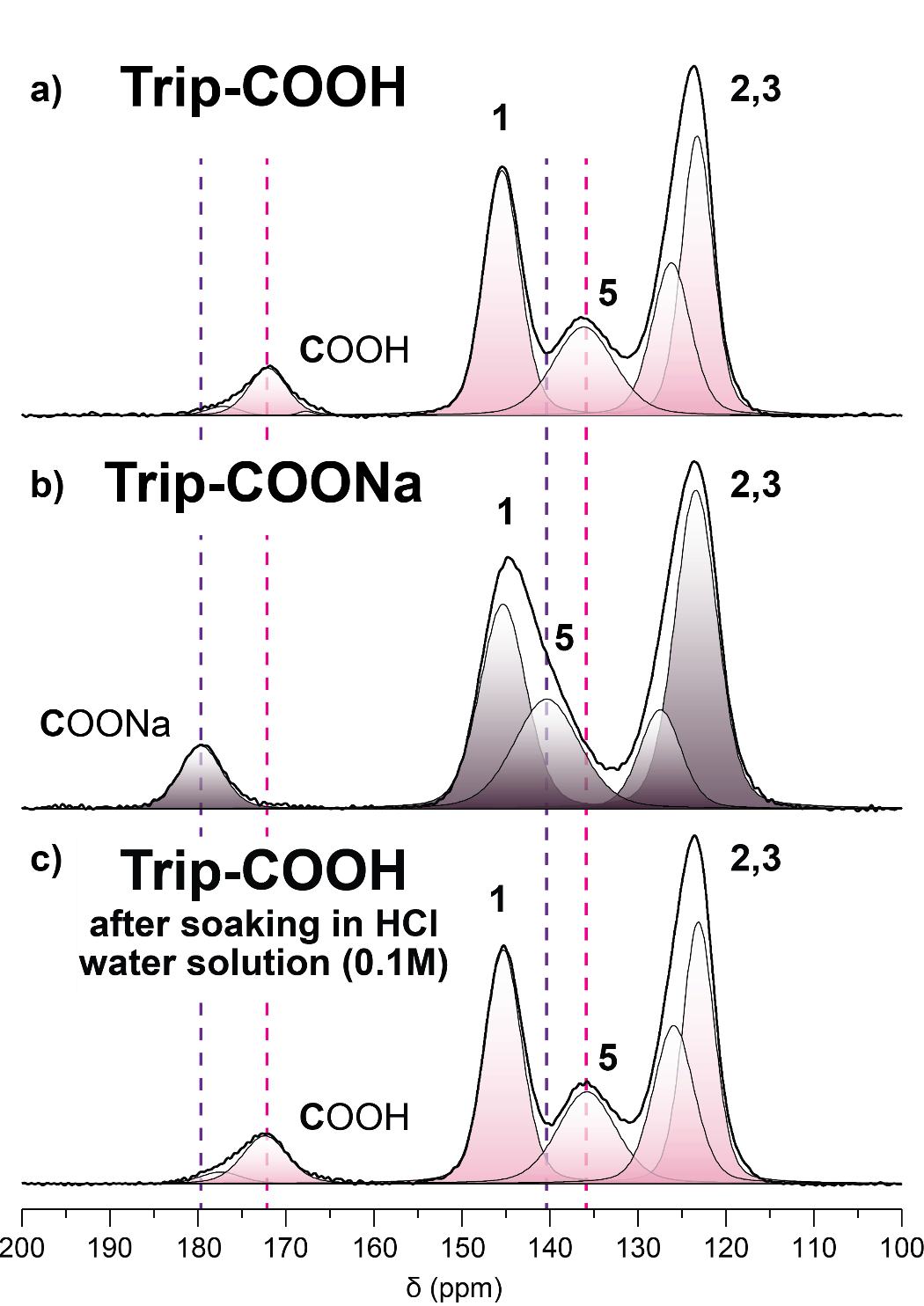
**

**Figure S56.** Zoom in the aromatic region of the ^13^C {^1^H} CP MAS spectra collected at room temperature, 7.04 T, with a spinning speed of 12.5 kHz and a contact time of 2 ms of a) **Trip-COOH**, b) **Trip-COONa**, c) **Trip-COOH** after soaking in HCl solution (0.1 M) for 10 days at room temperature.


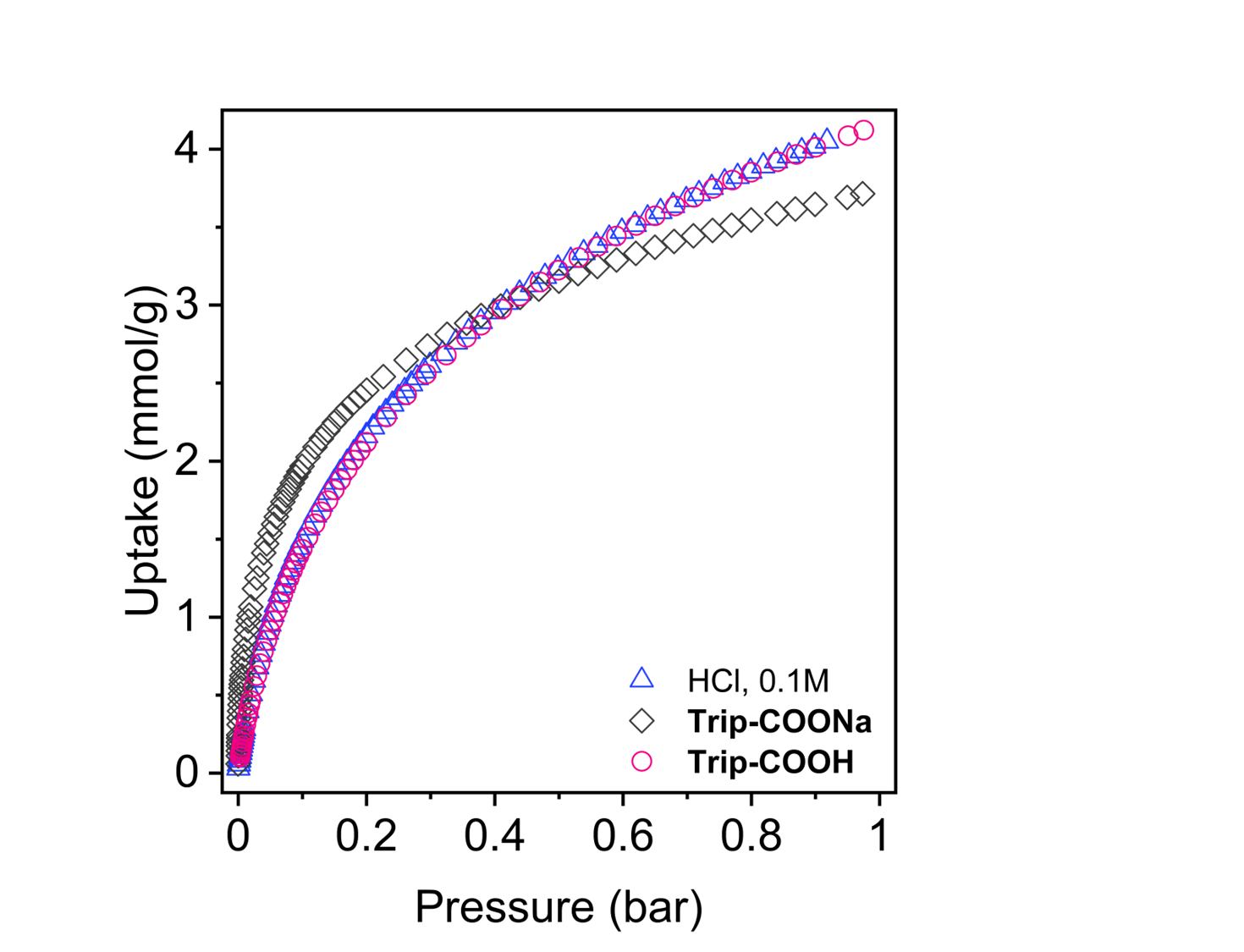


**Figure S57.** CO_2_ adsorption isotherms at 273 K of pristine **Trip-COOH**, **Trip-COONa** and after treatment with HCl (0.1 M). After soaking in a 0.1 M HCl solution for 10 days and activation under a high vacuum at 140°C, the sample displays an adsorption isotherm comparable to that collected for the pristine **Trip-COOH**. This result is in agreement with the solid-state NMR experiments (Figure S55, S56), which demonstrate the conversion of the sodium carboxylate groups to carboxylic acid after exposure to acidic conditions.

- **Adsorption-Coupled Calorimetry - Trip-COOH**

The calculated isosteric heat (Q_st_) of adsorption was validated against the direct measurement of the heat of adsorption using a homemade set-up for sorption-coupled microcalorimetry for **Trip-COOH**. The sorption isotherms were collected using a Micromeritics 2050 adsorber equipped with pressure cells. Simultaneously, the heat exchanged was measured using a microcalorimeter (Setaram µDSC7 evo). The measurement was repeated twice at 293 K to ensure reproducibility and improve the statistics.





**Figure S58**. CO_2_ adsorption isotherms collected at 293 K for **Trip-COOH**. The adsorption isotherms for measures 1 and 2 on the Micromeritics 2050 adsorber coupled to the microcalorimeter were consistent with the isotherm collected on the Micromeritics 2020 adsorber.

**
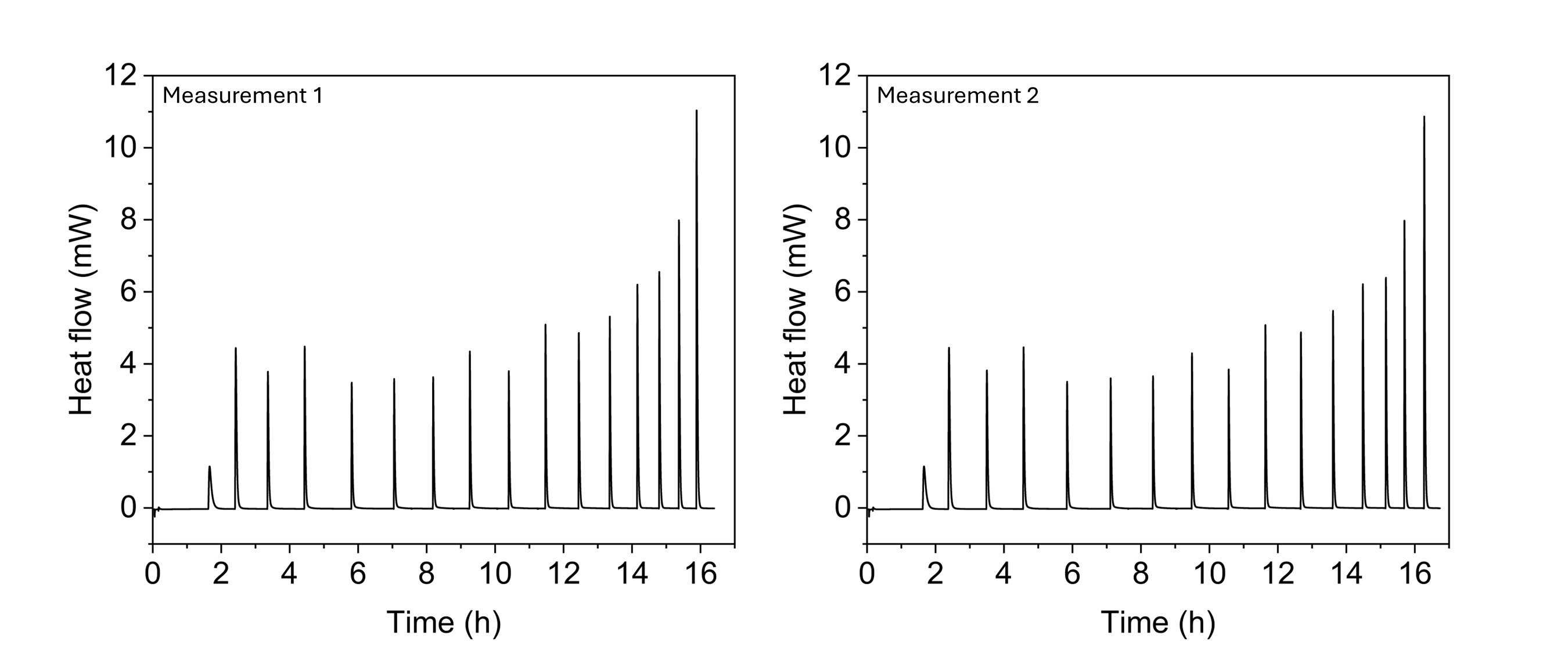
**

**Figure S59**. Heat flow measured using the ASAP 2050 and μDSC7 coupled system for measures 1 (left) and 2 (right). Each peak corresponded to the heat released during each CO_2_ loading.


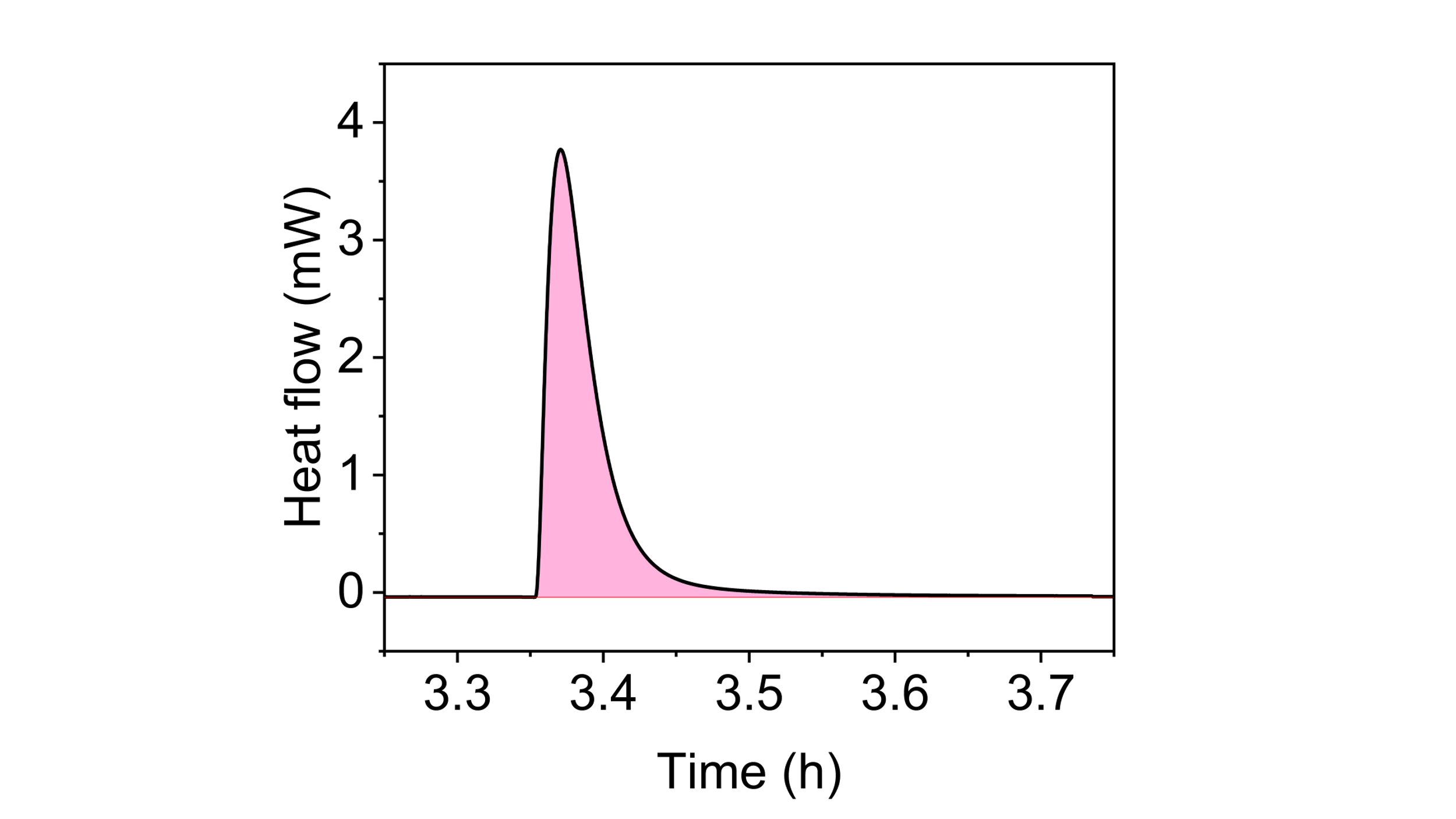


**Figure S60**. Expanded timeline between 3.25 and 3.75 hours, highlighting the peak shape of a single adsorption dose. The pink area highlighted the integral heat exchanged during the CO_2_ loading.


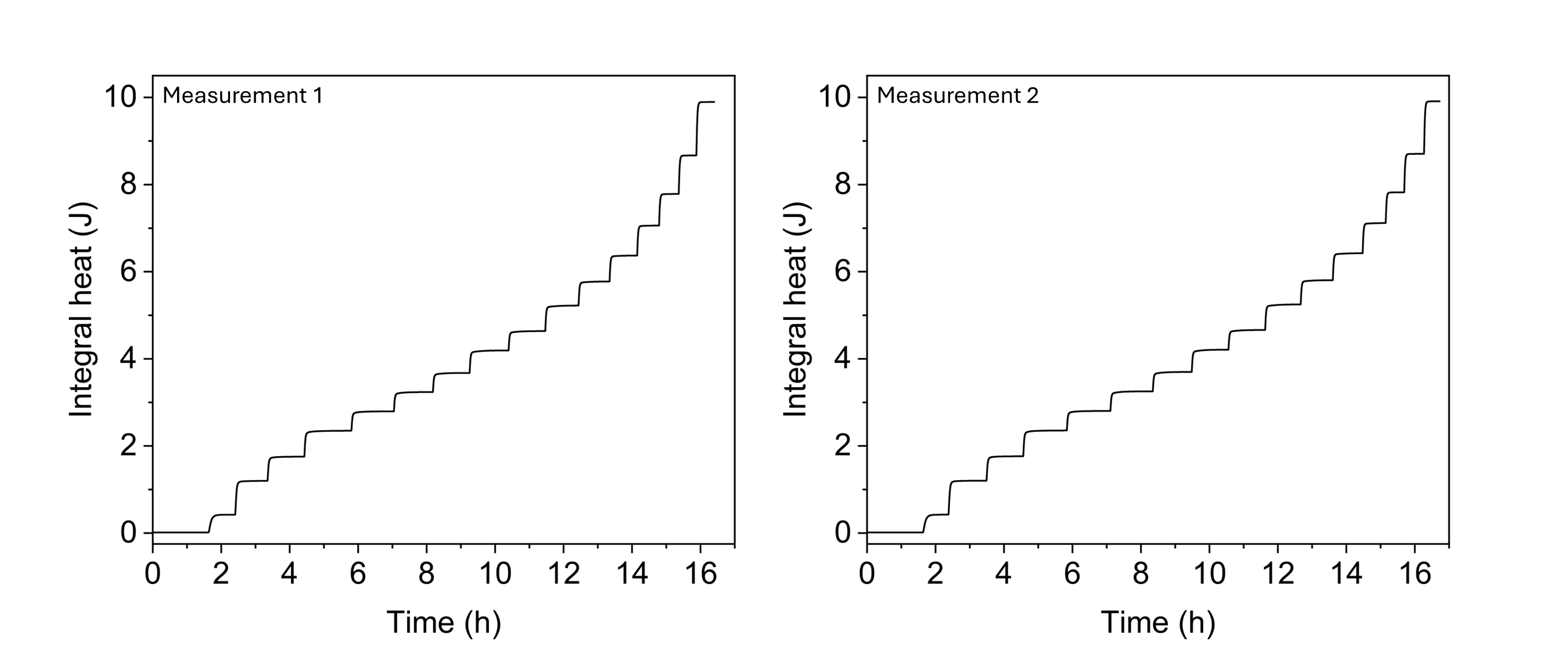


**Figure S61**. Cumulative integral heat exchanged during the CO_2_ loadings for measurements 1 (left) and 2 (right).


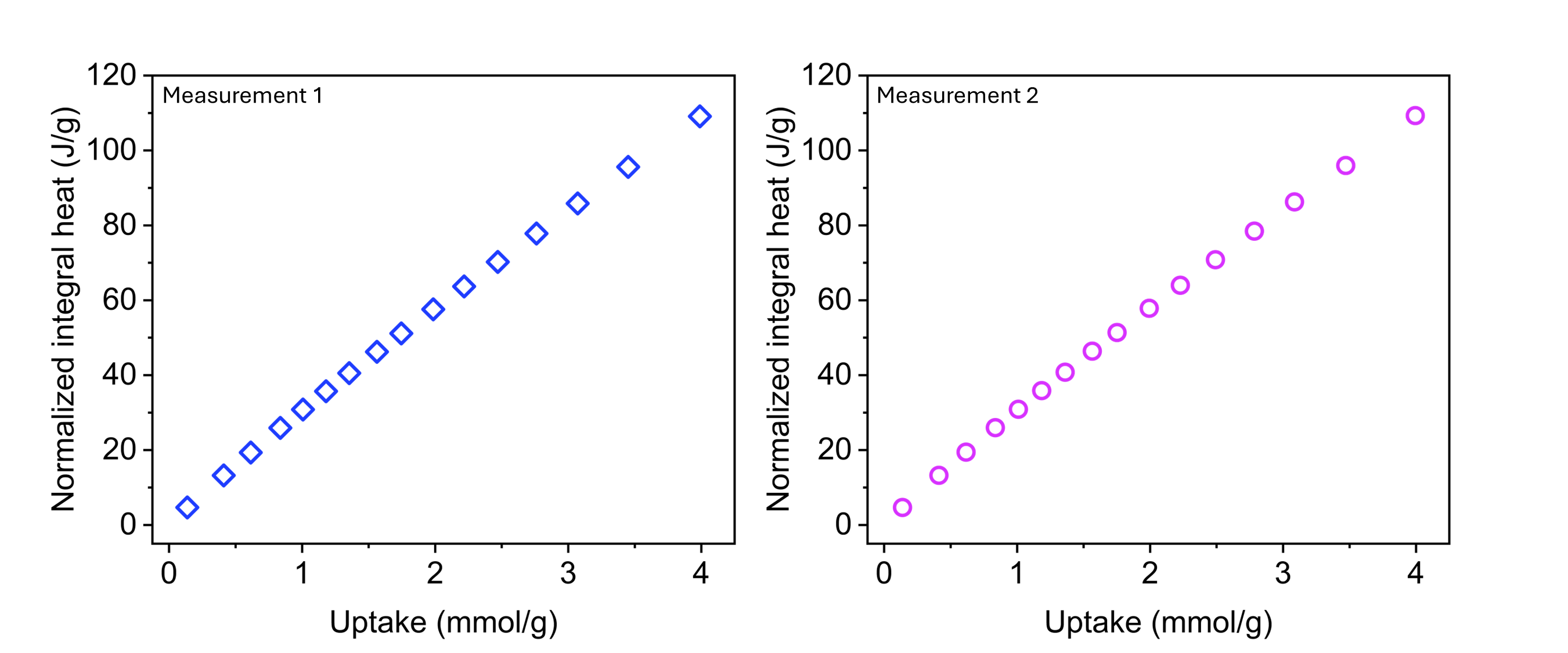


**Figure S62**. Normalized cumulative integral heat (J/g) measured during the CO_2_ loadings for measurements 1 (left) and 2 (right).

**

**

**Figure S63**. Isosteric heat of adsorption measured from adsorption-coupled calorimetry at 293 K. The measurements were performed twice to demonstrate the reproducibility.

- **Computational analysis**

**
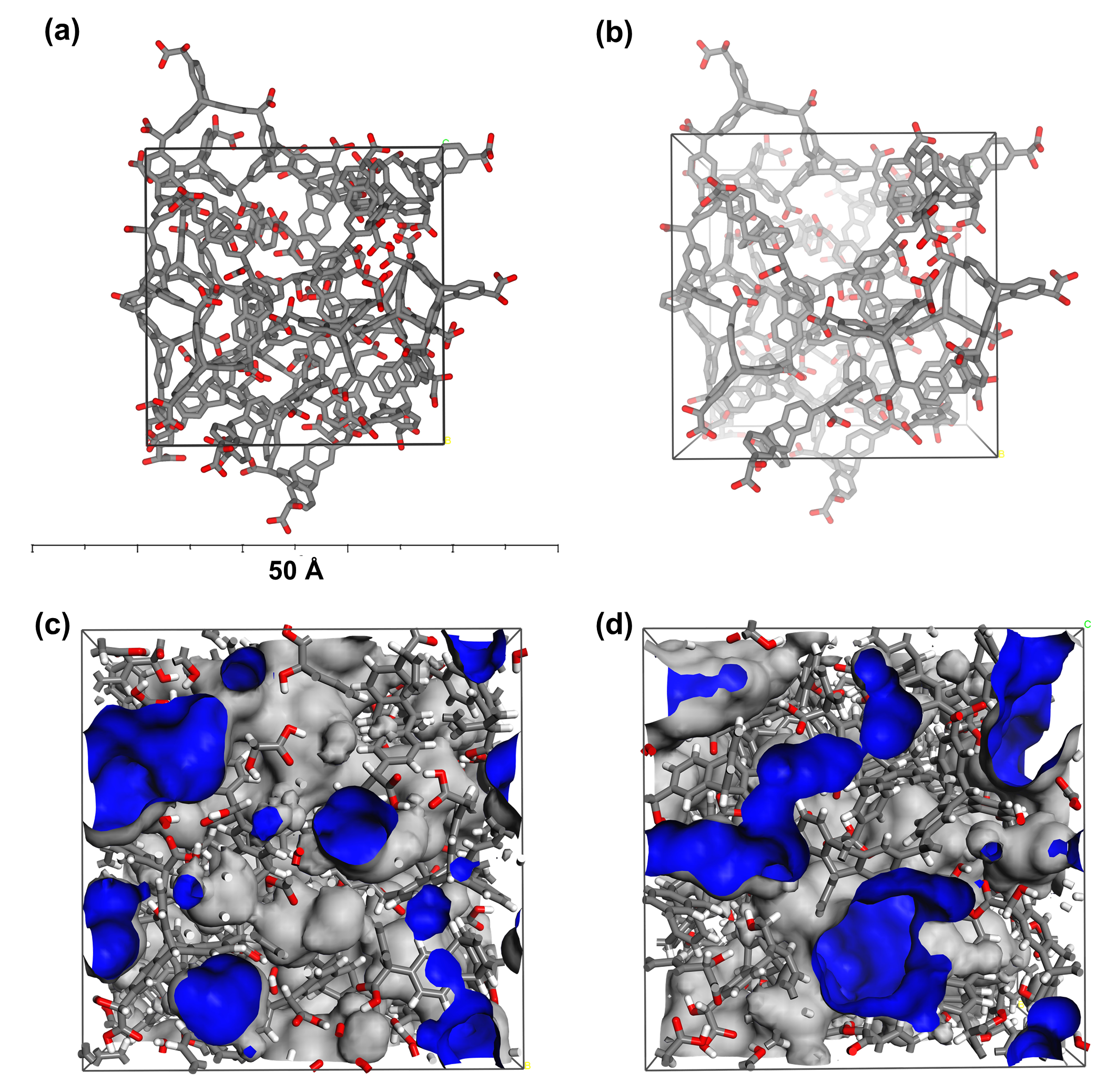
**

**Figure S64**. (a) and (b) Optimized model amorphous cell displayed in orthogonal and perspective view, respectively. (c) and (d) The isosurfaces show the available void volume in the amorphous structure calculated using a probe radius of 1.65 Å (kinetic diameter of CO_2_ = 3.3 Å), viewed along the **b** and **c** axes, respectively.

**
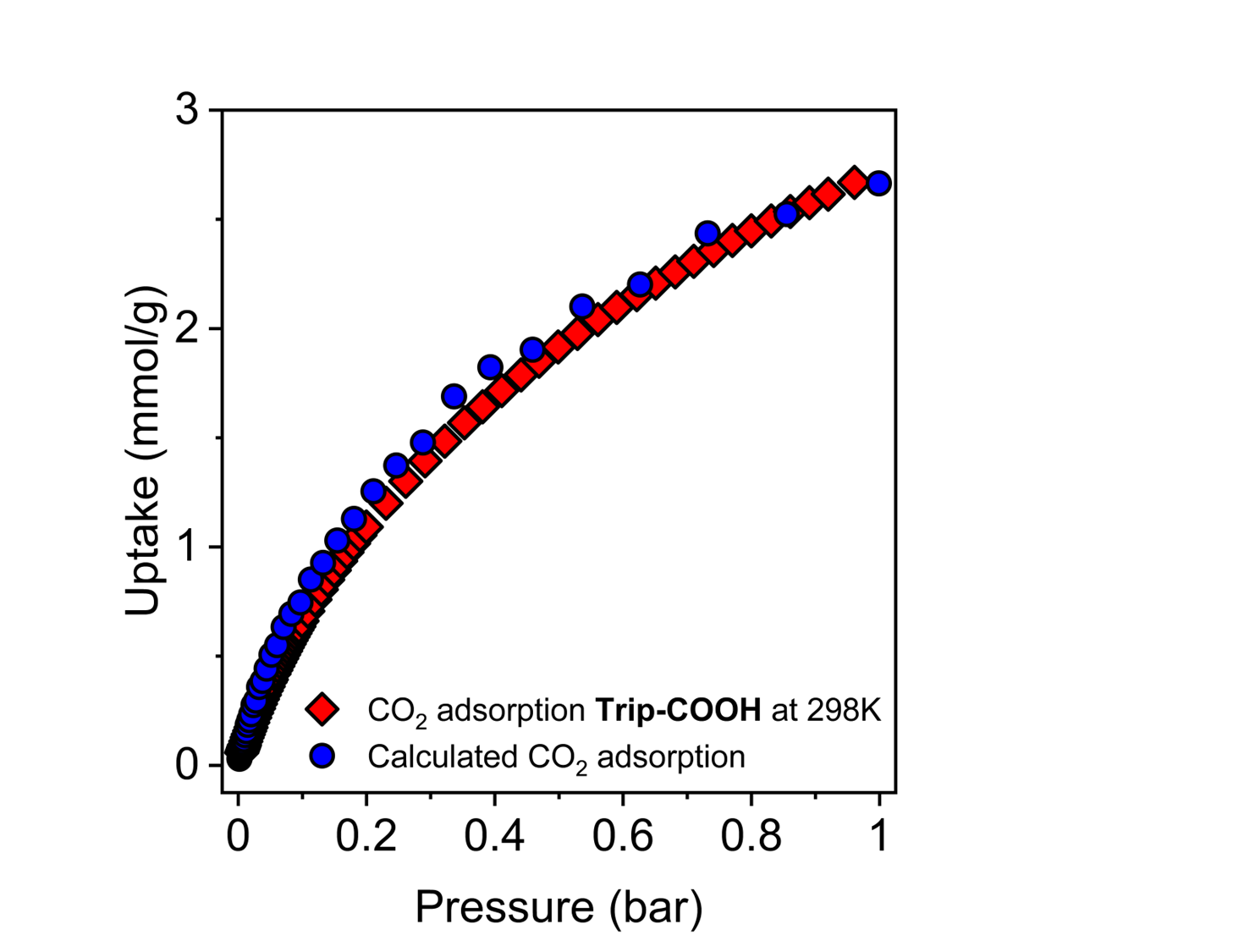
**

**Figure S65**. Comparison between the CO_2_ experimental adsorption isotherm of **Trip-COOH** measured at 298 K (red diamonds) and the calculated CO_2_ adsorption isotherm from the amorphous cell model using GCMC simulation (blue circles).

- **Reversibility, cyclability and stability of Trip-COOH and Trip-COONa**


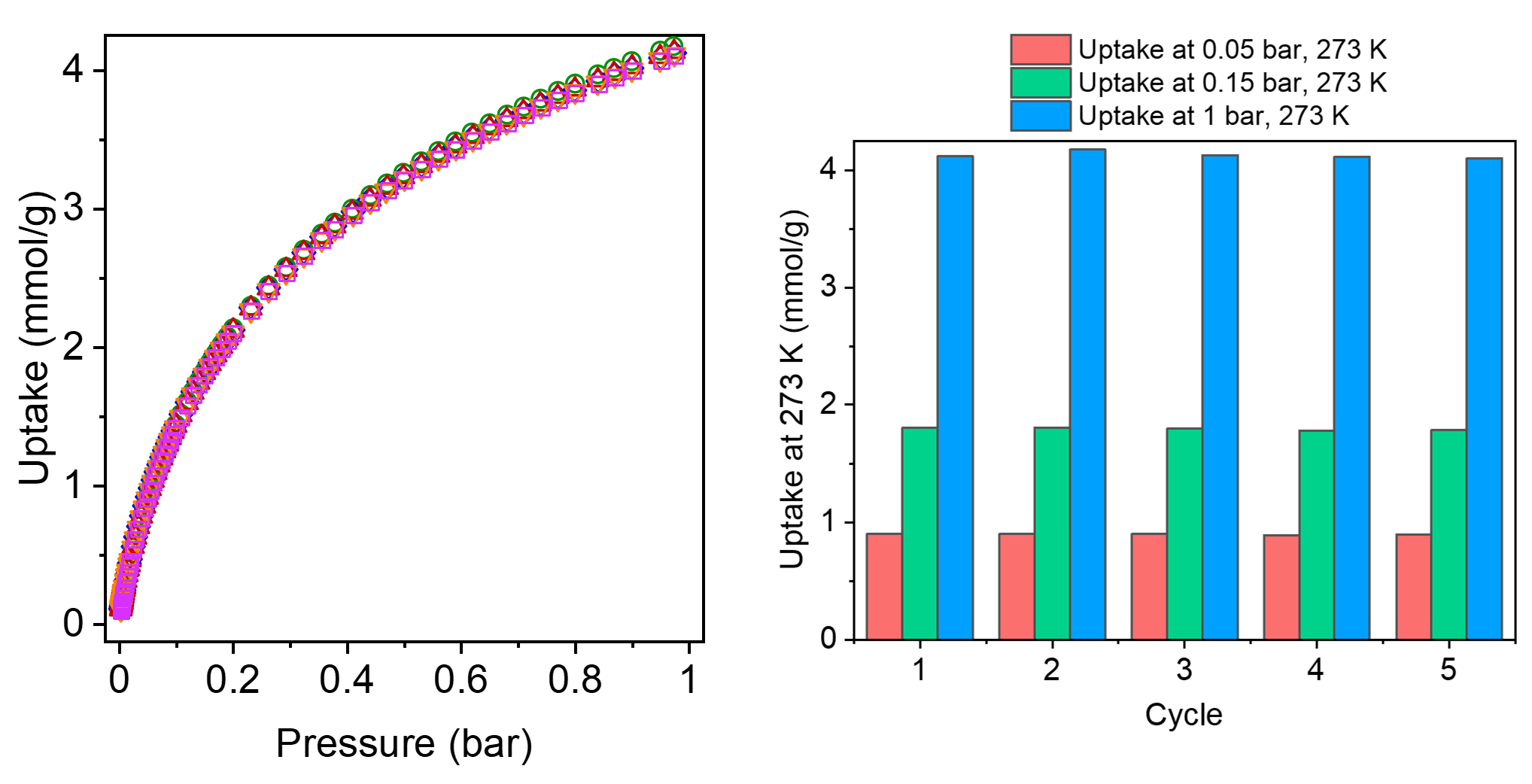


**Figure S66.** Cyclability of CO_2_ sorption in the sample **Trip-COOH**. The sample was activated at room temperature under high vacuum (p ≤ 1 µbar) for 3 hours after each CO_2_ sorption isotherm to ensure the complete release of adsorbed CO_2_.


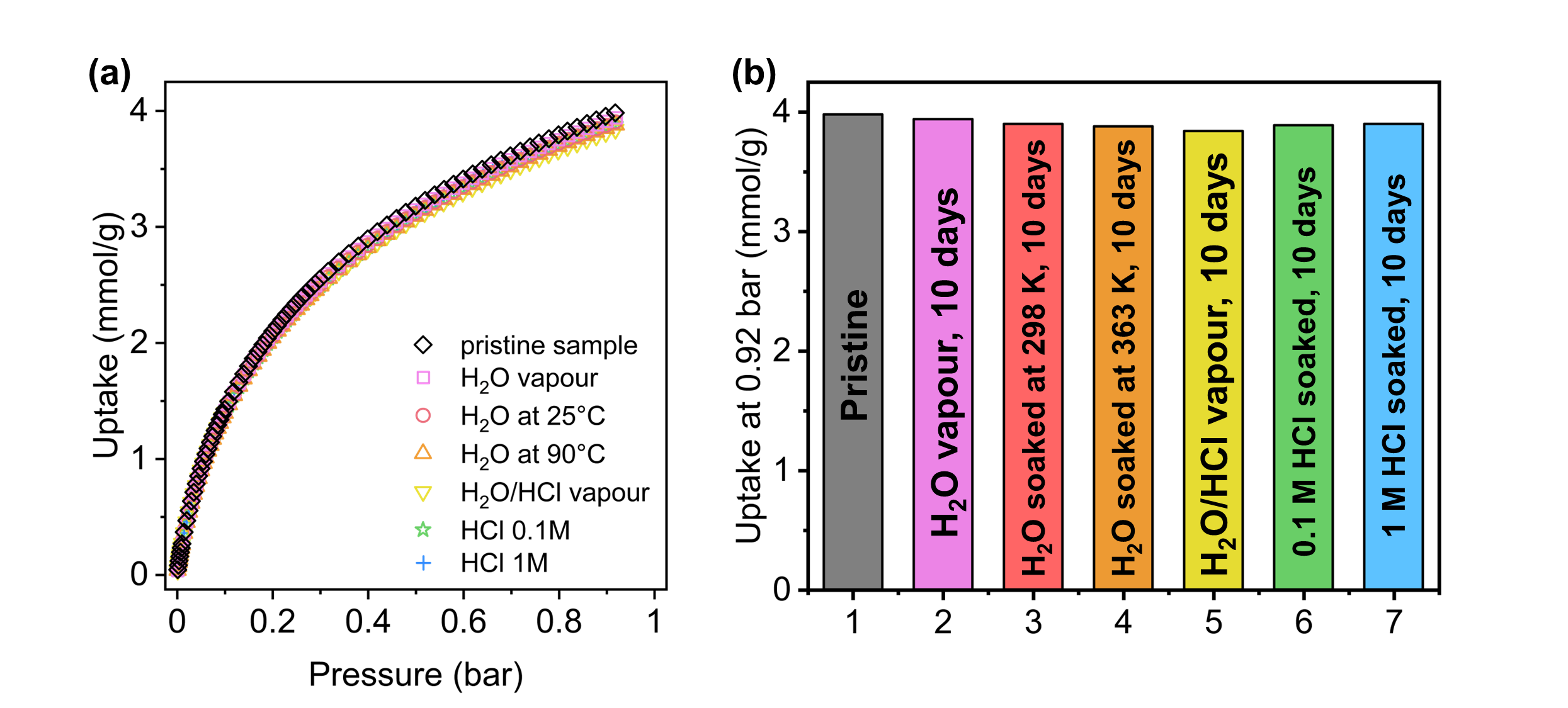


**Figure S67.** CO_2_ adsorption isotherms of **Trip-COOH** after distinct chemical treatments (sample **Trip-COOH_a** was used to perform stability tests) and re-activation under a high vacuum and 100°C: water vapour (RH ≈ 85%) (purple), soaking in water at 25°C and 90°C (red and orange, respectively), exposure to acidic vapour (humid HCl) (yellow), and in HCl solution (0.1 M and 1 M, green and light blue, respectively). **Trip-COOH** retains at least 97% CO_2_ sorption capacity at 1 bar and 273 K, demonstrating exceptional chemical and humidity stability.


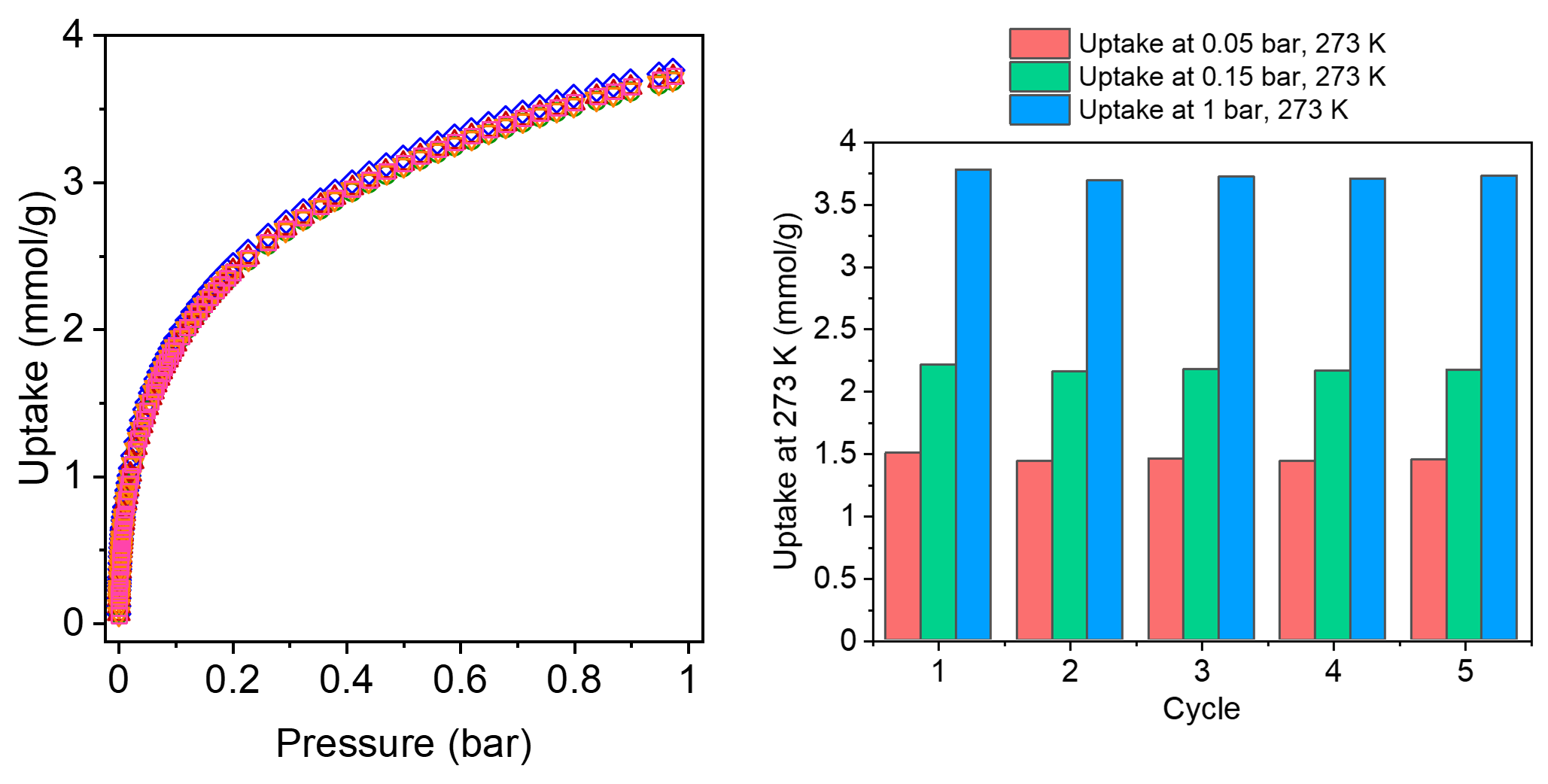


**Figure S68**. Cyclability test of CO_2_ sorption of sample **Trip-COONa**. The sample was activated at 100°C under high vacuum (p ≤ 1 µbar) for 3 hours after each CO_2_ sorption isotherm to ensure the complete release of adsorbed CO_2_.


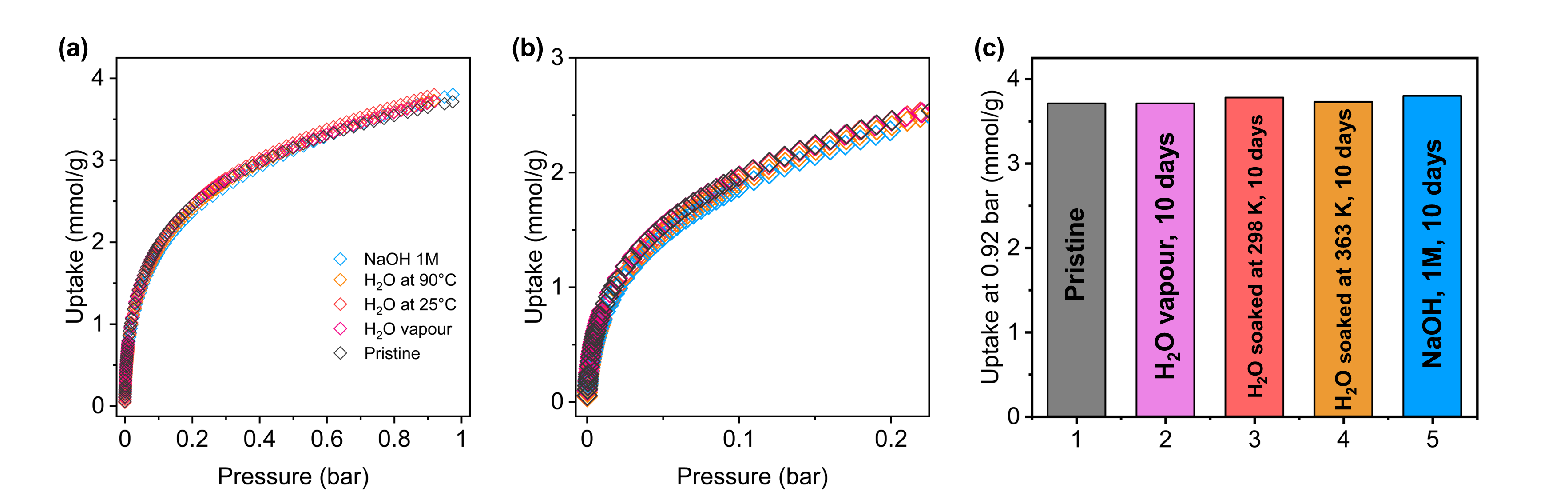


**Figure S69**. (a) CO_2_ adsorption isotherms of **Trip-COONa** at 273 K after distinct chemical treatments and re-activation under a high vacuum at 140°C: water vapour (RH ≈ 85%) (purple), soaking in water at 25°C and 90°C (red and orange, respectively), exposure to basic solution (NaOH, 1M, light blue). (b) Enlargement between 0 and 0.225 bar showing the shape of the sorption isotherms at low CO_2_ pressures. (c) histogram of the CO_2_ uptake at 0.92 bar and 273 K. The uptake at 1 bar and 273 K and the profile of the sorption isotherms of **Trip-COONa** after exposure to high humidity, soaking in water at 25°C and 90°C and basic solutions are in very good agreement with the pristine material, demonstrating the high stability of the material under these conditions.

- **Comparison with literature data**

**Table S15**. Comparison of the CO_2_ uptake at 298 K and pressures of 0.15 bar and 1 bar, CO_2_ isosteric heat of adsorption and CO_2_/N_2_ selectivity of **POP-COOHs** with notable POPs adsorbents under physisorption process.


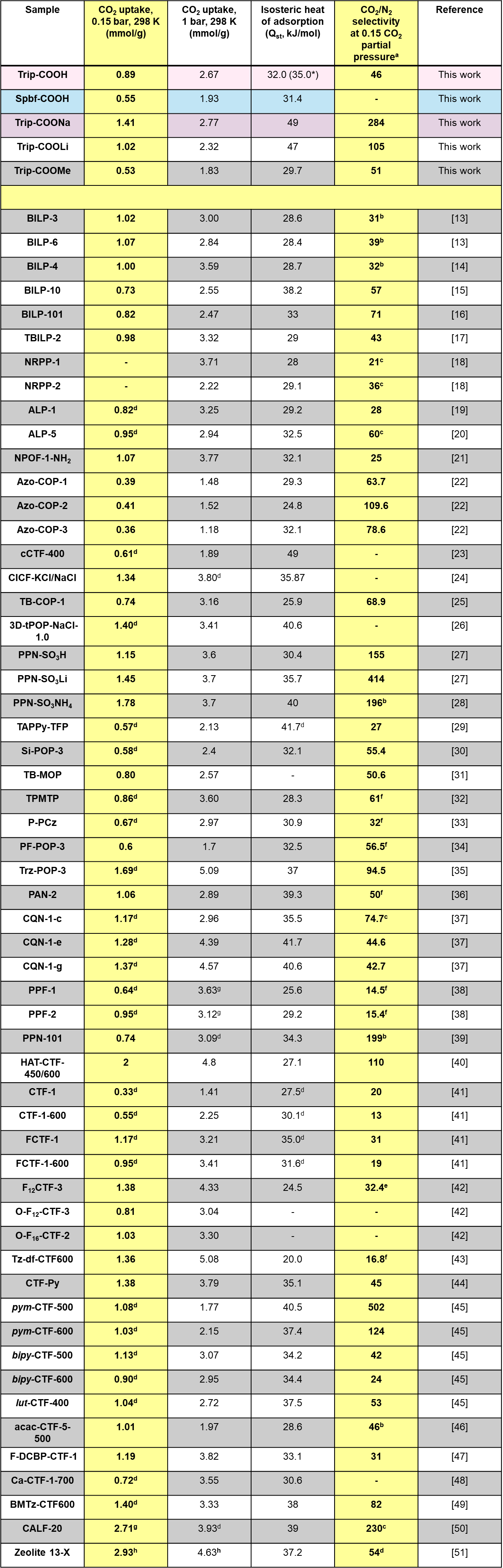


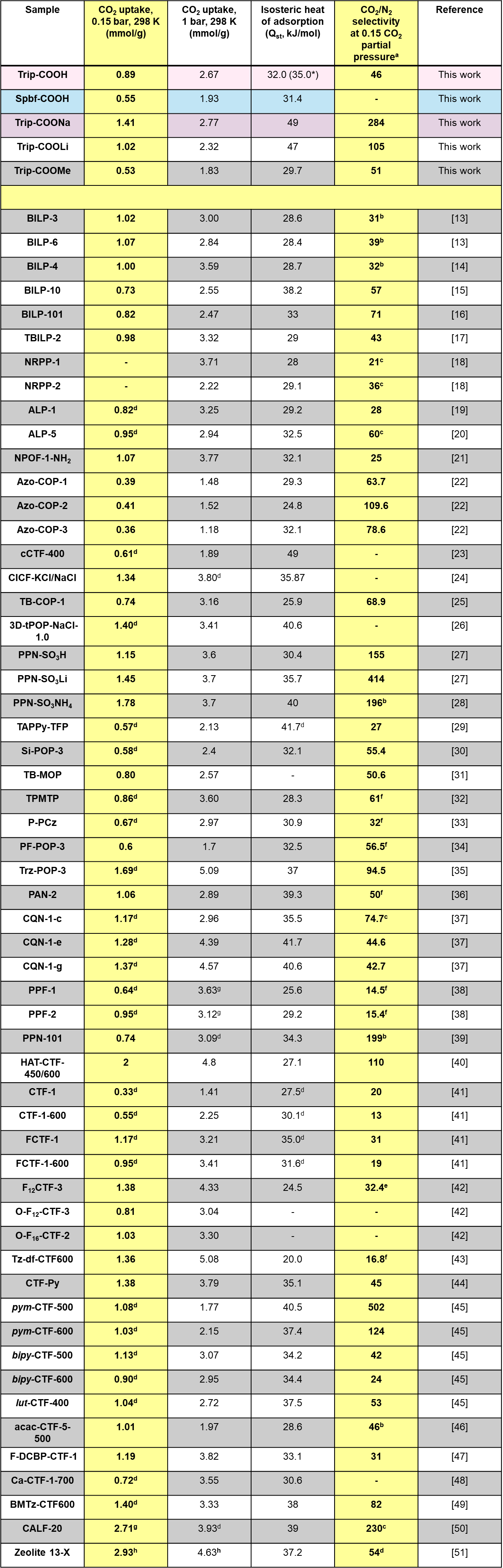


*Isosteric heat of adsorption measured directly with sorption-coupled calorimetry apparatus. ^a^IAST selectivity values are reported calculated at 15:85 CO_2_/N_2_ concentration at 298 K. ^b^Selectivity calculated according to the initial slope method. Selectivity calculated for a 10:90 CO_2_/N_2_ concentration. ^d^Data digitized from the literature. ^e^Selectivity calculated according to the initial slope method at 273 K. ^f^IAST selectivity calculated at 273 K. ^g^Data digitized from the literature considering the adsorption isotherm at 293 K. ^h^Data digitized from the literature. The experimental data were fitted with the Langmuir-Freundlich equation, and the adsorption values were calculated from the fitting curve.

- **Polymer-based composites: Trip-COOH@PVA and Trip-COONa@PVA**

**^13^C and ^1^H solid-state NMR spectroscopy**

**
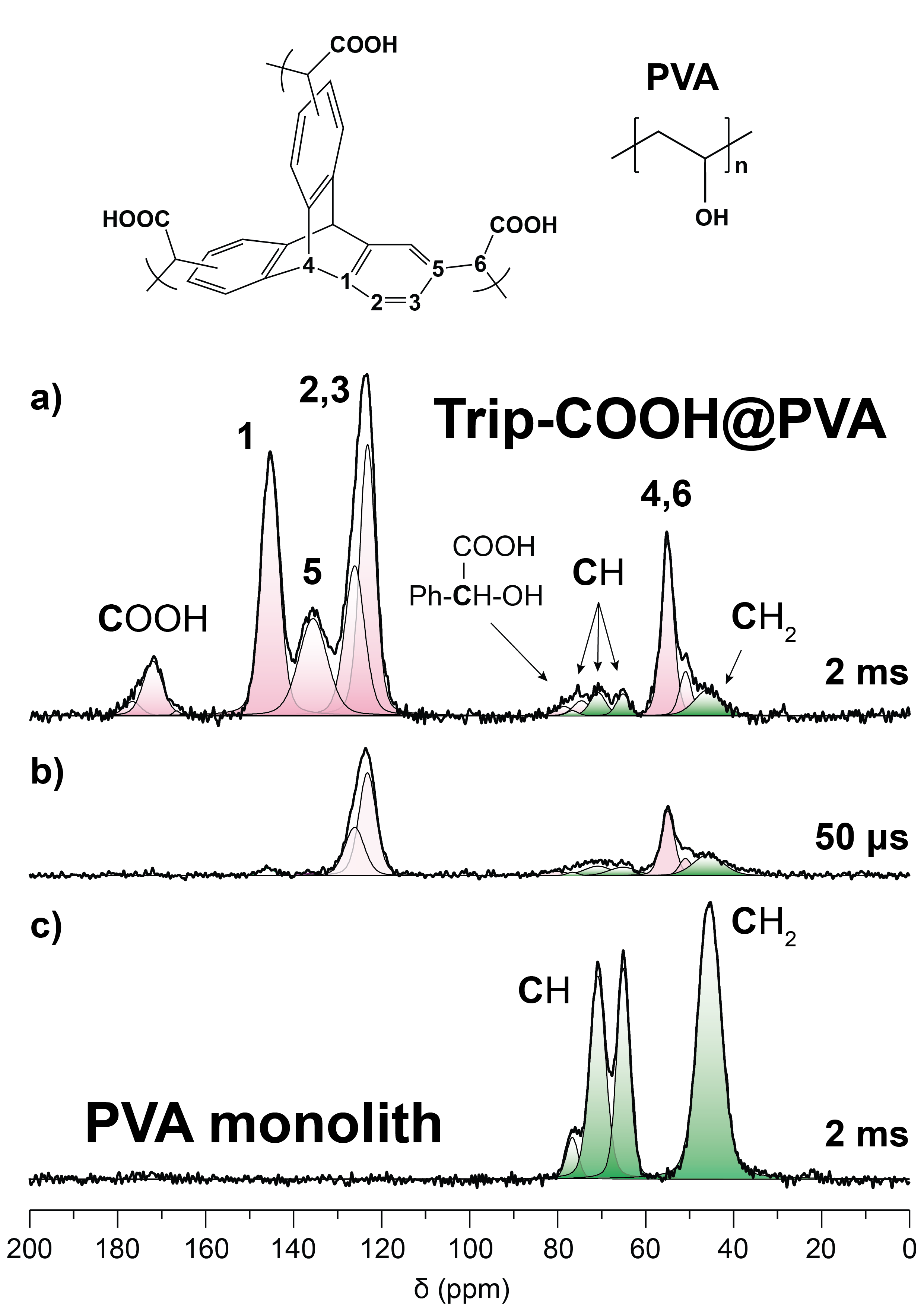
**

**Figure S70**. ^13^C CPMAS NMR spectra performed at room temperature, 7.04 T, with a spinning speed of 12.5 kHz of a) **Trip-COOH@PVA** with 2 ms (a) and 50 ms (c), and **PVA monolith**.

**Table S16**. ^13^C chemical shifts of PVA monolith and **Trip-COOH@PVA** from the simulation of ^13^C {^1^H} CP MAS spectra collected at room temperature, 7.04 T, with a spinning speed of 12.5 kHz and a contact time of 2 ms.^[52]^

| **Trip-COOH@PVA** | **Assignment** | **δ (ppm)**  **^13^C {^1^H} CP MAS** |
| --- | --- | --- |
| 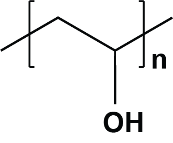 | **CH (mm)** | 76.6 |
|  | **CH (mr)** | 70.8 |
|  | **CH (rr)** | 65.1 |
|  | **CH_2_** | 45.5 |
| **Trip-COOH@PVA** | **Assignment** | **δ (ppm)**  **^13^C {^1^H} CP MAS** |
| 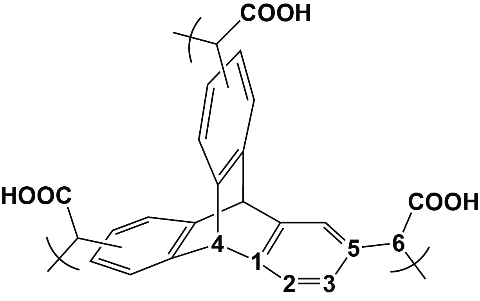  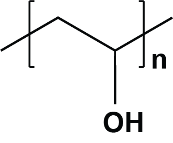 | **C**OOH | 176.6, 172.0, 166.6 |
|  | **1** | 145.3 |
|  | **5** | 135.6 |
|  | **2, 3** | 126.1, 123.1 |
|  | **C**H-OH-COOH | 78.5, 74.55 |
|  | **C**H (PVA) | 76.6, 70.8, 65.1 |
|  | **6, 4** | 55.0, 50.9 |
|  | **C**H_2_ (PVA) | 45.7 |

**Infrared spectroscopy**

**
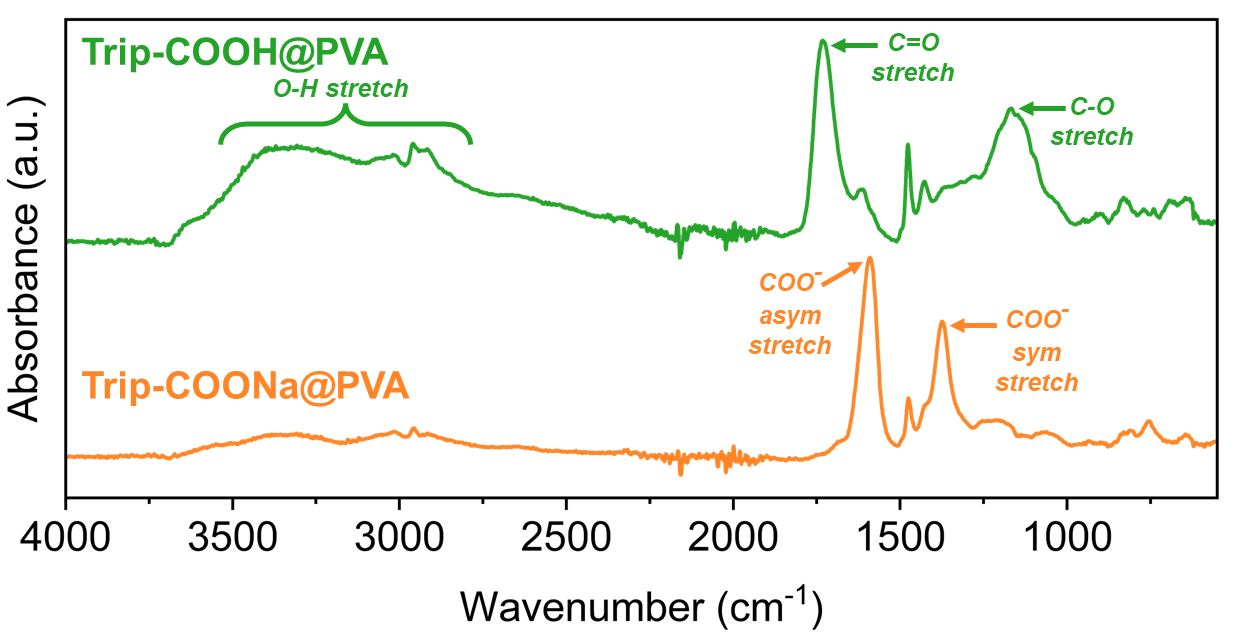
**

**Figure S71**. FT-IR spectra of **Trip-COOH@PVA** (top, green) and **Trip-COONa@PVA** (bottom, orange) collected from 525 cm^-1^ to 4000 cm^-1^. The characteristic vibrational bands associated with the carboxylic acid (**Trip-COOH@PVA)** and carboxylate (**Trip-COONa@PVA)** groups are highlighted in the figures.

**Thermal analysis**

**
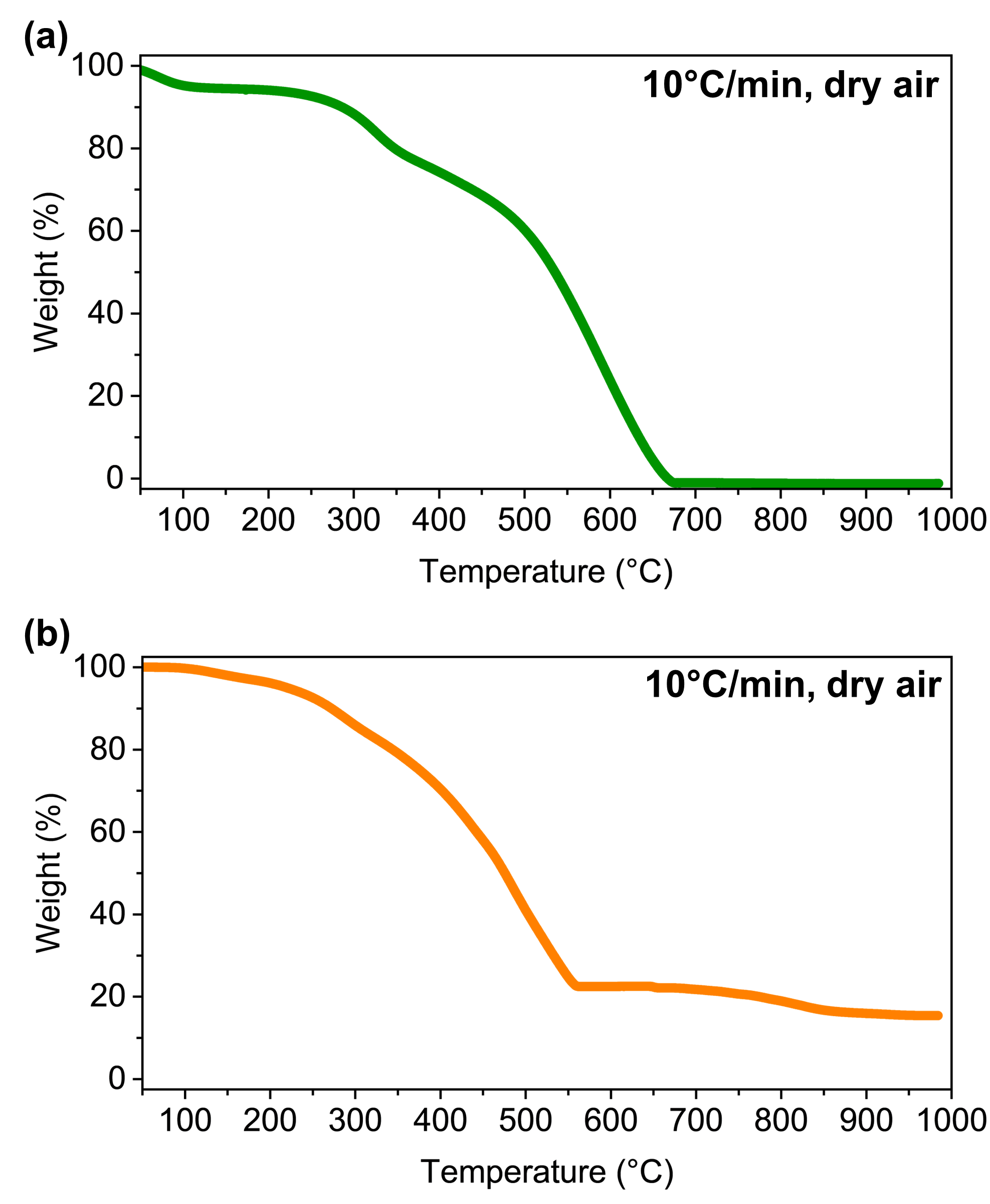
**

**Figure S72**. Thermal analysis of (a) **Trip-COOH@PVA** and (b) **Trip-COONa@PVA** collected from 50°C to 1000°C. The thermogram was collected under dry air flow (50 mL/min) with a heating rate of 10°C/min.

**FT-IR coupled TGA analysis**


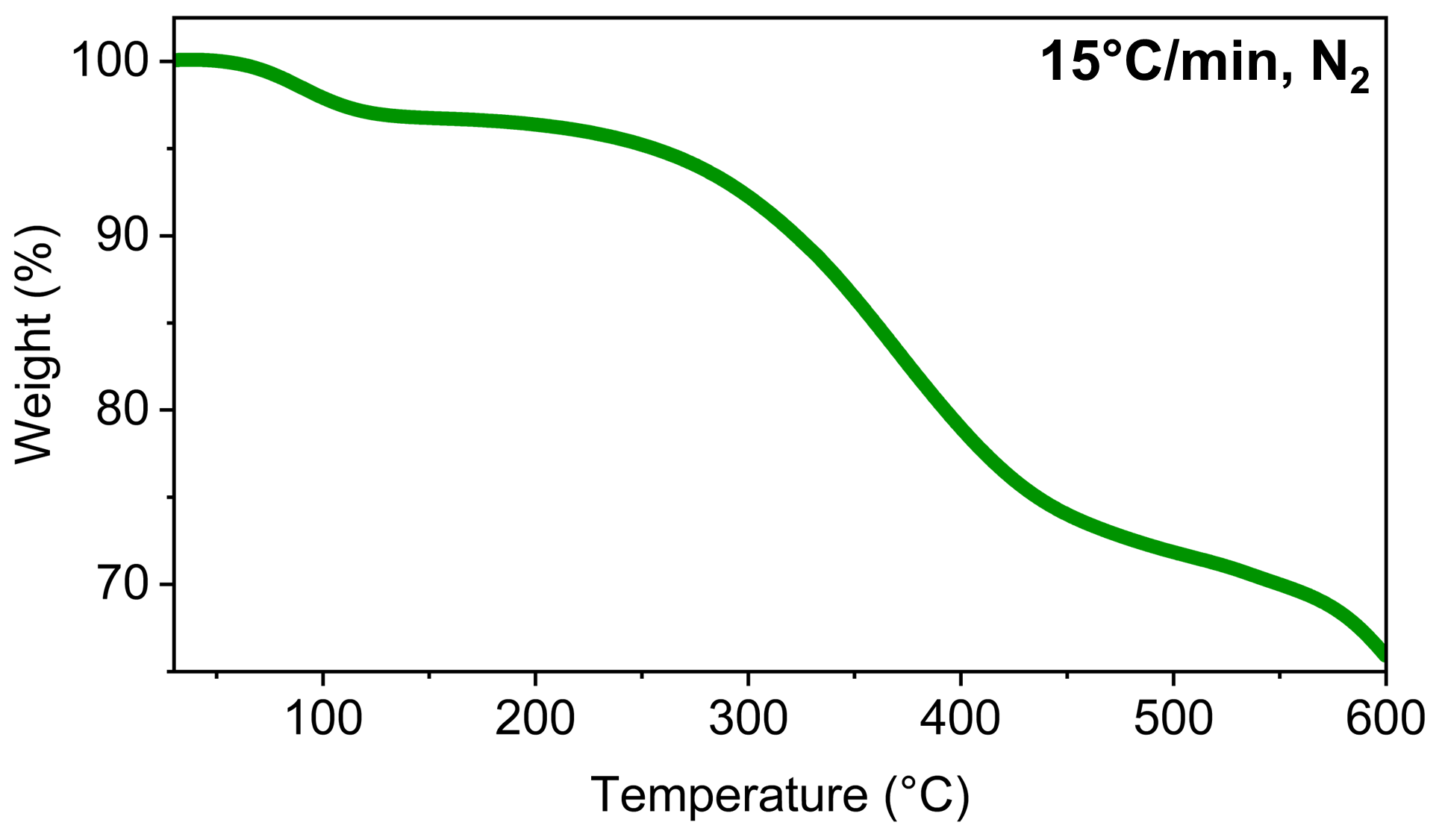


**
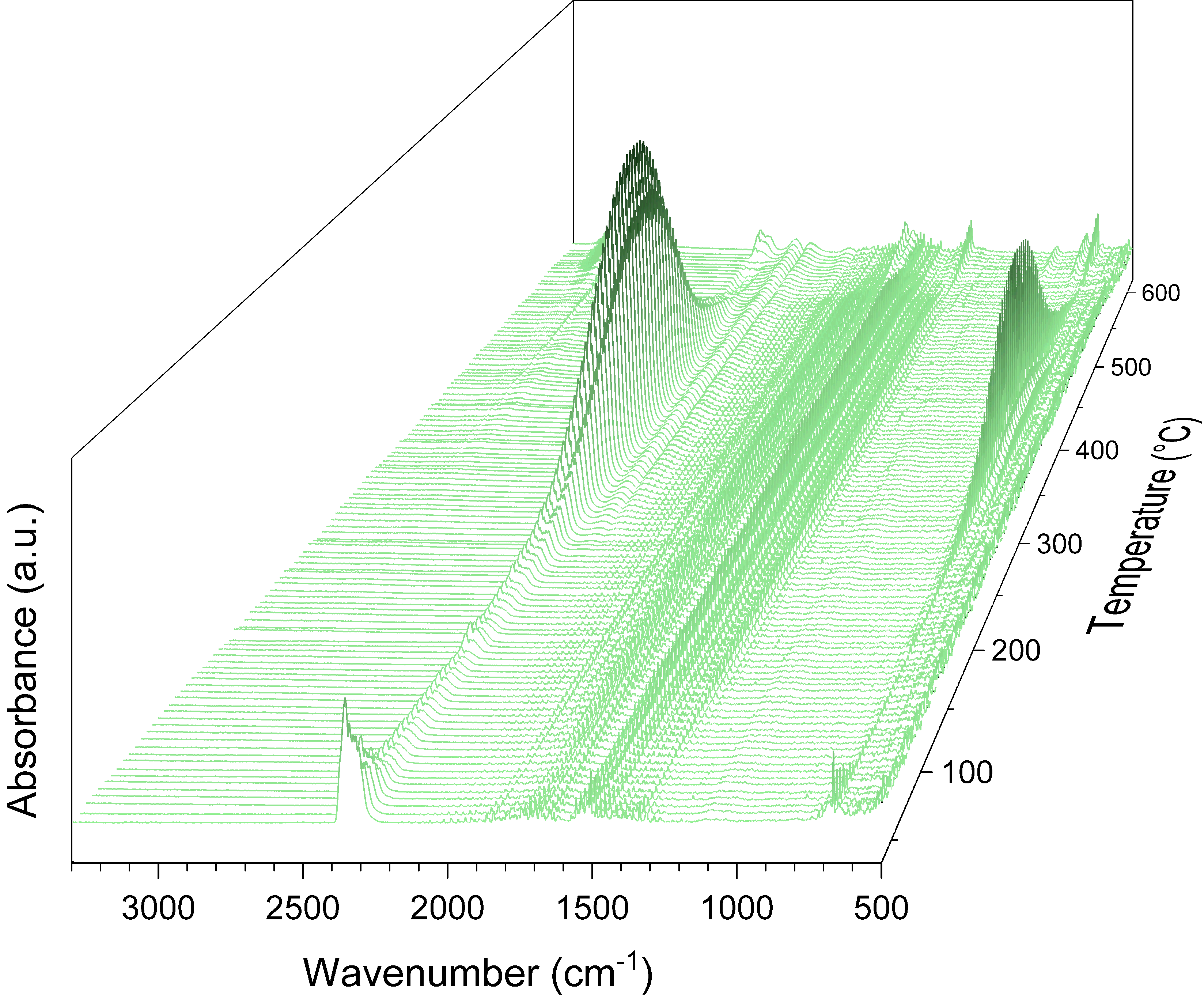
**

**Figure S73**. Top: thermal analysis of **Trip-COOH@PVA** coupled with FT-IR. The thermogram was collected under N_2_ flow (50 mL/min) with a heating rate of 15°C/min. Bottom: 3D plot of TGA-IR data of **Trip-COOH@PVA**. The plot displays the FT-IR spectra as a function of the sample temperature. The release of CO_2_ molecules in the temperature range between 50°C and 150°C is related to the desorption of guest molecular species from the porous framework. In the temperature range between 300°C and 500°C, strong IR-active bands centered at 668 cm^-1^ and between 2300/2350 cm^-1^ are due to the bending and asymmetric stretching of CO_2_, respectively.

**
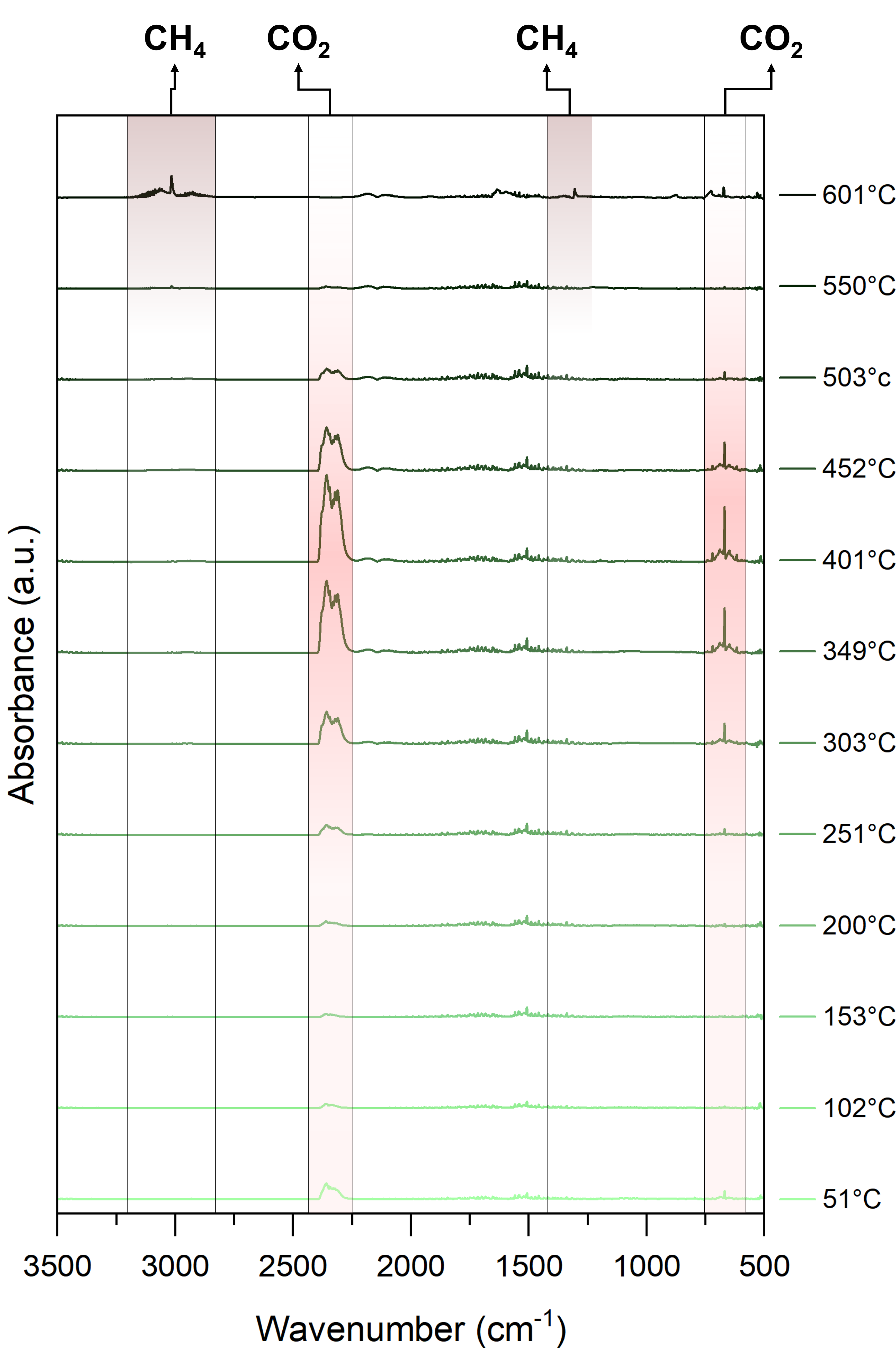
**

**Figure S74**. Selected FT-IR spectra of the evolved gases collected at different temperatures from **Trip-COOH@PVA**. The colored boxes highlight the CO_2_ vibrational bands.

**Powder X-ray diffraction**

**
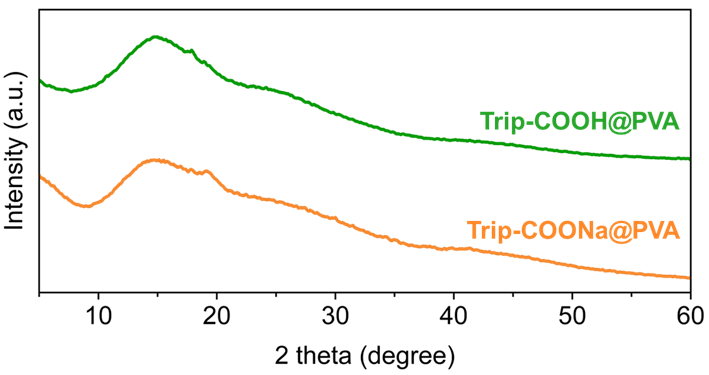
**

**Figure S75**. Powder X-ray diffraction patterns of the two polymer composites **Trip-COOH@PVA** (top, green) and **Trip-COONa@PVA** (bottom, orange) collected from 5 to 60 2 theta degrees.

**Gas adsorption measurements**

**
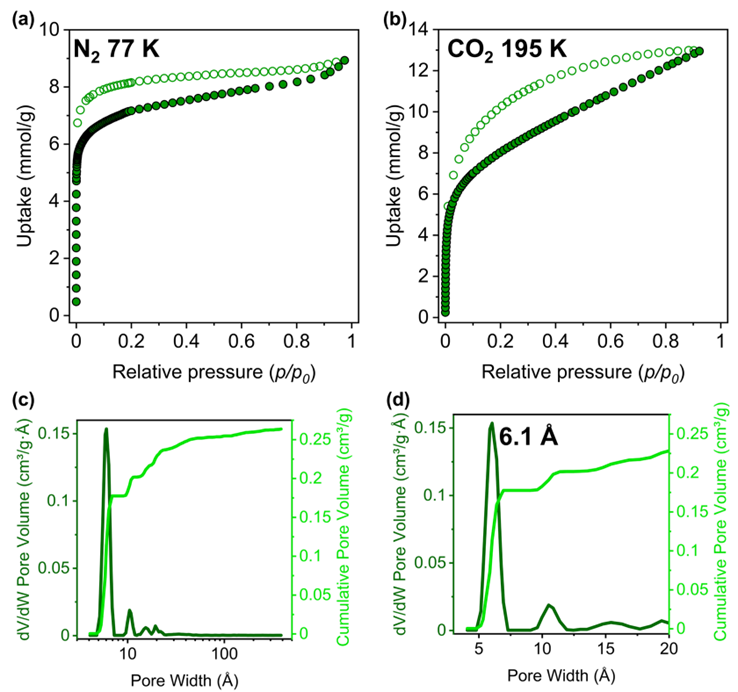
**

**Figure S76**. (a) N_2_ adsorption isotherm collected at 77 K and (b) CO_2_ adsorption isotherm collected at 195 K of **Trip-COOH@PVA**. Filled and empty symbols represent sorption and desorption branches, respectively. (c) Differential pore size distribution and cumulative pore size distribution calculated from N_2_ adsorption isotherm at 77 K according to NLDFT theory and HS-2D-NLDFT, Carbon, N2, 77 K pore model. (d) Enlargement between 3 Å and 20 Å of the differential and cumulative pore size distribution highlights the ultramicroporous nature of **Trip-COOH@PVA**.

**Table S17**. Textural properties measured and calculated from cryogenic N_2_ sorption (77 K) and CO_2_ sorption isotherms collected at 273 K, 283 K, 293 K and 298 K for samples **Trip-COOH@PVA** and **Trip-COONa@PVA**.

| Sample | Langmuir surface area (m^2^/g)^1^ | BET surface area (m^2^/g)^1^ | Pore volume (cm^3^/g)^2^ | Micropore volume (cm^3^/g)^2^ | CO_2_ uptake at 1 bar, 273 K (mmol/g) | CO_2_ uptake at 1 bar, 298 K (mmol/g) | Q_st_ at 0.1 mmol/g (kJ/mol) ^3^ |
| --- | --- | --- | --- | --- | --- | --- | --- |
| Trip-COOH@PVA | 662 | 607 | 0.26 | 0.23 | 3.75 | 2.57 | 31.1 |
| Trip-COONa@PVA | 179 | 166 | - | - | 2.90 | 2.03 | 45.2 |

^1^ BET surface areas were calculated in the range 0.015<*p/p_0_*<0.06 according to the Rouquerol analysis. Langmuir surface areas were calculated in the range 0.015<*p/p_0_*<0.06. ^2^ Total and micropore volumes were calculated according to NLDFT theory and HS-2D-NLDFT Carbon, N_2_, 77 K pore model. Micropore volume was evaluated in the pore size range between 0 Å and 20 Å.^3^ The isosteric heats of adsorption were calculated using the virial method, using the CO_2_ sorption isotherms collected at 273 K, 283 K, 293 K and 298 K.

**
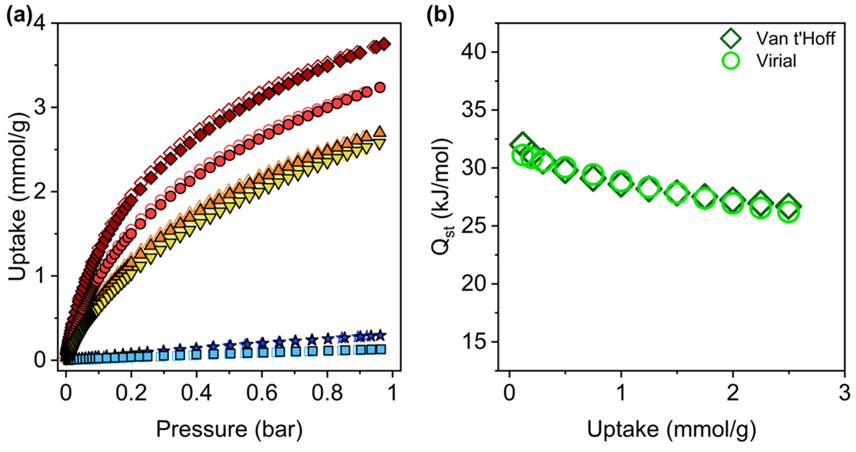
**

**Figure S77**. (a) CO_2_ adsorption isotherms of **Trip-COOH@PVA** collected at 273 K (diamonds, dark red), 283 K (circles, red), 293 K (up-pointing triangles, orange) and 298 K (down-pointing triangles, yellow), and N_2_ adsorption isotherms collected at 273 K (stars, blue) and 298 K (squares, light-blue). Filled and empty symbols represent sorption and desorption branches, respectively. (b) Isosteric heat of adsorption for CO_2_ calculated from the isotherms collected at different temperatures using the virial method (circles, light-green) and the Van’t Hoff method after fitting with the Langmuir-Freundlich model (diamonds, green).

**Comparison of CO_2_ volumetric adsorption capacity in Trip-COOH and Trip-COOH-PVA**

The experimental density of the pore walls of **Trip-COOH-PVA**, measured by He picnometry, is ρ_w_ = 1.40 g cm^-3^, and the apparent density, calculated considering the pore capacity of 0.26 cm^3^ g^-1^, corresponds to ρ_a_ = 0.97 g cm^-3^. For comparison, we measured the experimental density of the pore walls of **Trip-COOH (**ρ_w_ = 1.36 g cm^-3^) and the apparent density, calculated considering the pore capacity of 0.35 cm^3^ g^-1^, corresponding to ρ_a_ = 0.93 g cm^-3^. Taking into account the CO_2_ uptake at 298 K and 1 bar of 2.67 mmol g^-1^ for **Trip-COOH** and 2.57 mmol g^-1^ for **Trip-COOH@PVA**, the CO_2_ volumetric adsorption capacity of both samples is the same.

**
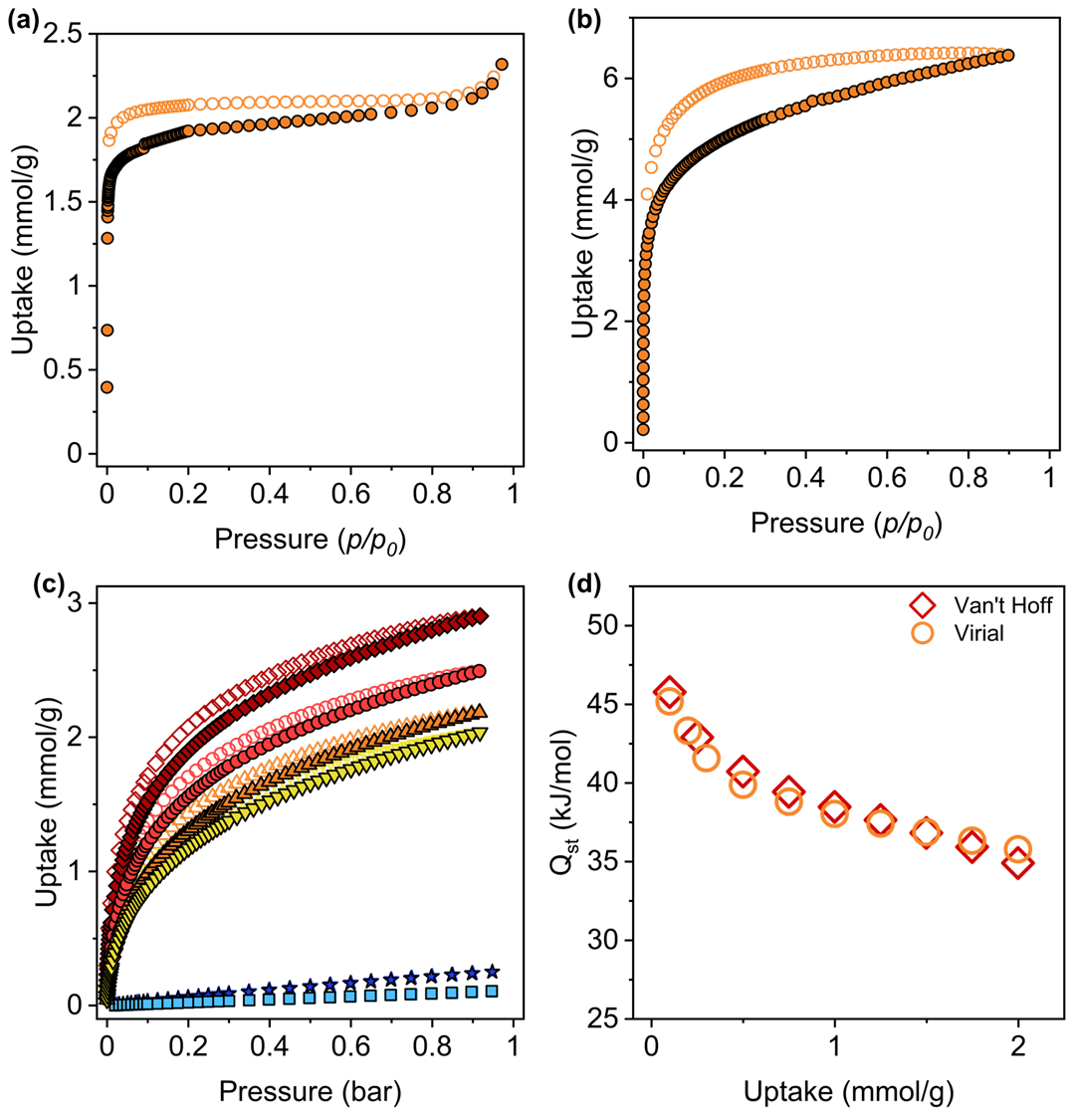
**

**Figure S78**. (a) N_2_ adsorption isotherm collected at 77 K and (b) CO_2_ adsorption isotherm collected at 195 K of **Trip-COONa@PVA**. (c) CO_2_ adsorption isotherms of sample **Trip-COONa@PVA** collected at 273 K (diamonds, dark red), 283 K (circles, red), 293 K (up-pointing triangles, orange) and 298 K (down-pointing triangles, yellow), and N_2_ adsorption isotherms collected at 273 K (stars, blue) and 298 K (squares, light-blue). Filled and empty symbols represent sorption and desorption branches, respectively. (d) Isosteric heat of adsorption for CO_2_ calculated from the isotherms collected at different temperatures using the virial method (circles, orange) and the Van’t Hoff method after fitting with the Langmuir-Freundlich model (diamonds, red).

**Figure S79**. (a) Top: pore size distribution of **Trip-COOH@PVA** calculated from N_2_ adsorption isotherm at 77 K according to the HS 2D-NLDFT theory and carbon slit pore model for nitrogen adsorption at 77 K (light green). Bottom: pore size distribution of **Trip-COOH@PVA** calculated from CO_2_ adsorption isotherm at 273 K according to the HS 2D-NLDFT theory and carbon slit pore model for CO_2_ adsorption at 273 K (dark green). (b) Pore size distribution of **Trip-COONa@PVA** calculated from CO_2_ adsorption isotherm at 273 K according to the HS 2D-NLDFT theory and carbon slit pore model for CO_2_ adsorption at 273 K (orange).

- **Continuous flow gas mixture separation (breakthrough experiments)**

**Figure S80**. Column breakthrough curves for **Trip-COOH@PVA** for CO_2_/N_2_ separation (20:80 mixture) at 298 K and ambient pressure with a total flow of 6 sccm. **Trip-COOH@PVA** effectively separated high-purity CO_2_ (N_2_ < 1.0%) for 14 min/g.

**Figure S81**. Experimental breakthrough curves for a 15:85 CO_2_:N_2_ mixture on **Trip-COOH@PVA** with total flow rate of 3 sccm, 6 sccm, 9 sccm and 12 sccm normalized on the sample mass. The time scales were corrected considering the dead volume of the experimental set-up using dedicated blank measurements as detailed in the experimental details.

**Figure S82**. (a) Experimental breakthrough curves of the first 100 cycles for a 15:85 CO_2_:N_2_ mixture on **Trip-COOH@PVA** with a total flow rate of 6 sccm (N_2_ and CO_2_ breakthrough curves were displayed with light blue-to-dark blue and orange-to-red colors, respectively). (b) Enlargement between 0 and 25 minutes of the breakthrough curves. The time scales were corrected to account for the dead volume of the experimental apparatus as described in the experimental details.

**Figure S83**. (a) Experimental CO_2_ breakthrough curves of the first 100 cycles for a 15:85 CO_2_:N_2_ mixture on **Trip-COOH@PVA** with a total flow rate of 6 sccm. The plot highlighted the region between 12.5 and 22.5 min/g to display the high reproducibility of the cycles. The time for y_CO2_ / y_CO2,0_ = 0.01 is 14.5±0.2 min/g, corresponding to a relative change of less than 1.5% over a hundred cycles.

**Figure S84**. Five experimental breakthrough curves for a 15:85 CO_2_:N_2_ mixture on **Trip-COONa@PVA** with total a flow rate of 6 sccm showing the recyclability of the materials in CO_2_/N_2_ separation process (N_2_ and CO_2_ breakthrough curves were displayed with violet-to-blue and orange-to-red colors, respectively). The sample was activated for 3 hours at 120°C after each breakthrough cycle to remove the adsorbed CO_2_.

- **POP-COOH@PVA membrane**

Mixed-matrix membranes were prepared using **Trip-COOH** or **Trip-COONa** porous polymers and polyvinyl alcohol (PVA) as a continuous polymer matrix by the casting method. The synthetic procedure is illustrated below.

**Trip-COOH** or **Trip-COONa** (see the table S18 and S19 for the details, respectively) were dispersed in water (2 mL). A stock solution of Polyvinyl alcohol (PVA) in water was prepared (30 mg/mL) and added to the dispersions of the porous polymers in order to obtain a final concentration of 10 wt% and 50 wt% of the porous polymer in the final membrane (see the tables below for the details). The mixtures were sonicated for 1 minute and then stirred at 60°C in open air to remove the water partially. Once the water content was reduced to ~ 1.5 mL, the solution was cooled to room temperature and transferred to a Teflon-lined circular mold. The membranes were treated at 35°C for 24 h and then peeled off.

Four samples were prepared with different weight percentages of **Trip-COOH** and **Trip-COONa**: 1) two samples with 10wt% and 50wt% of porous polymer in PVA, denominated **10wt%_Trip-COOH in PVA** and **50wt%_Trip-COOH in PVA**, respectively;

2) two samples with 10wt% and 50wt% of porous polymer in PVA, denominated **10wt%_Trip-COONa in PVA** and **50wt%_Trip-COONa in PVA**, respectively.

**Table S18**. Amounts of **Trip-COOH** and polyvinyl alcohol stock solution in the preparation of PVA-based MMM.

| **Sample** | **Trip-COOH (mg)** | **PVA stock solution, 30 mg/mL**  **(mL)** |
| --- | --- | --- |
| **10wt%_Trip-COOH in PVA** | 12 | 3.6 |
| **50wt%_Trip-COOH in PVA** | 60 | 2 |

**Table S19**. Amounts of **Trip-COONa** and polyvinyl alcohol stock solution in the preparation of PVA-based MMM.

| **Sample** | **Trip-COOH (mg)** | **PVA stock solution, 30 mg/mL**  **(mL)** |
| --- | --- | --- |
| **10wt%_Trip-COONa in PVA** | 12 | 3.6 |
| **50wt%_Trip-COONa in PVA** | 60 | 2 |

The following figures display the PVA-based membranes containing different amounts of **POP-COOHs**.

**Figure S85**. Digital images of the PVA-based membranes.

**Figure S86**. Digital image of the bending of **50wt%_Trip-COOH in PVA** mixed-matrix membrane.

Inspired by the literature work (reference 9 in the main text), we developed a modified procedure that produces membranes using an *in-situ* polymerization method. We applied the following procedure. Triptycene (800 mg) was dissolved in 2.6 mL of 1,2-dichloroethane (solution A). Separately, glyoxylic acid monohydrate (652 mg) was dissolved in trifluoromethane sulfonic acid (2 mL), and the solution was cooled to 0°C using a water-ice bath (solution B). 1.3 mL of solution A and 1 mL of solution B were quickly mixed and polymerized into a Teflon mold for 24 hours at room temperature. The reaction was quenched with water and washed with a water and acetone mixture. Finally, the membrane was extracted from the mold and dried in air.

**Figure S87**. Membrane obtained using an *in-situ* polymerization reaction.

- **Supplementary References**

# [1] G. Metz, X. Wu, S. O. Smith, Ramped-Amplitude Cross Polarization in Magic-Angle-Spinning NMR. *J. Magn. Reson. A* **1994**, 110, 219-227.

[2] D. A. Torchia, A. Szabo, Spin-Lattice Relaxation in Solids, J. Magn. Reson. 1969, 49, 107-121.

[3] D. Massiot, F. Fayon, M. Capron, I. King, S. Le Calvé, B. Alonso, J-O. Durand, B. Bujoli, Z. Gan, G. Hoatson, *Magn. Res. Chem.* **2002**, 40, 70-76.

[4] S. Sircar, R. Mohr, C. Ristic, M. B. Rao, Isosteric heat of Adsorption: Theory and Experiment. *J. Phys. Chem. B* **1999**, *103*, 6539-6546.

[5] J. A. Dunne, R. Mariwala, M. Rao, S. Sircar, R. J. Gorte, A. L. Myers, Calorimetric Heats of Adsorption and Adsorption Isotherms. 1. O_2_, N_2_, Ar, CO_2_, CH_4_, C_2_H_6_, and SF_6_ on Silicalite. *Langmuir***1996**, *12*, 5888-5895.

[6] W. K. Feldmann, K.-A. White, C. X. Bezuidenhout, V. J. Smith, C. Esterhuysen, L. J. Barbour, Direct Determination of Enthalpies of Sorption Using Pressure-Gradient Differential Scanning Calorimetry: CO2 Sorption by Cu-HKUST. *ChemSusChem* **2020**, *13*, 102-105.

[7] J. Perego, C. X. Bezuidenhout, A. Pedrini, S. Bracco, M. Negroni, A. Comotti, P. Sozzani, Reorientable fluorinated aryl rings in triangular channel Fe-MOFs: an investigation on CO2-matrix interactions. *J. Mater. Chem. A* **2020**, *8*, 11406-11413.

[8] N. S. Wilkins, A. Rajendran, S. Farooq, Dynamic column breakthrough experiments for measurement of adsorption equilibrium and kinetics. *Adsorption* **2021**, *27*, 397-422.

[9] Z. Hu , Y. Wang, S. Farooq, D. Zhao, A Highly Stable Metal-Organic Framework with Optimum Aperture Size for CO_2_ Capture. **2017**, *63*, 9, 4103-4114.

[10] T. T.T. Nguyen, J.-B. Lin, G. K.H. Shimizu b, A. Rajendran, Separation of CO2 and N2 on a hydrophobic metal organic framework CALF-20. *Chemical Engineering Journal* **2024**, *442*, 136263.

[11] R Bassilakis, R.M Carangelo, M.A Wójtowicz, TG-FTIR analysis of biomass pyrolysis. *Fuel*, **2001**, *80*, 12, 1765-1786.

[12] J. González-Rivera, R. Iglio, G. Barillaro, C. Duce, and M. R. Tinè, Structural and Thermoanalytical Characterization of 3D Porous PDMS Foam Materials: The Effect of Impurities Derived from a Sugar Templating Process. *Polymers*, **2018**, *10*, 616.

# [13] M. G. Rabbani , T. E. Reich , R. M. Kassab , K. T. Jackson, H. M. El-Kaderi, High CO_2_ uptake and selectivity by triptycene-derived benzimidazole-linked polymers, *Chem. Commun.* **2012**, *48*, 1141-1143.

[14] M. G. Rabbani, H. M. El-Kaderi, Synthesis and Characterization of Porous Benzimidazole-Linked Polymers and Their Performance in Small Gas Storage and Selective Uptake, *Chem. Mater.* **2012**, *24*, 8, 1511-1517.

[15] A. K. Sekizkardes, T. Islamoglu, Z. Kahveci, H. M. El-Kaderi, Application of pyrene-derived benzimidazole-linked polymers to CO_2_ separation under pressure and vacuum swing adsorption settings, *J. Mater. Chem. A*, **2014**, *2*, 12492-12500.

[16] A. K. Sekizkardes, J. T. Culp, T. Islamoglu, A. Marti, D. Hopkinson, C. Myers, H. M. El-Kaderi, H. B. Nulwala, An ultra-microporous organic polymer for high performance carbon dioxide capture and separation, *Chem. Commun.* **2015**, *51*, 13393-13396.

[17] A. K. Sekizkardes, S. Altarawneh, Z. Kahveci, T. İslamoğlu, H. M. El-Kaderi, Highly Selective CO_2_ Capture by Triazine-Based Benzimidazole-Linked Polymers, *Macromolecules* **2014**, *47*, 8328-8334.

[18] Y. H. Abdelmoaty, T.-D. Tessema, F. A. Choudhury, O. M. El-Kadri, H. M. El-Kaderi, Nitrogen-Rich Porous Polymers for Carbon Dioxide and Iodine Sequestration for Environmental Remediation, *ACS Appl. Mater. Interfaces* **2018**, *10*, 18, 16049-16058.

[19] P. Arab, M. G. Rabbani, A. K. Sekizkardes, T. İslamoglu, H. M. El-Kaderi, Copper(I)-Catalyzed Synthesis of Nanoporous Azo-Linked Polymers: Impact of Textural Properties on Gas Storage and Selective Carbon Dioxide Capture, *Chem. Mater.* **2014**, *26*, 1385-1392.

[20] P. Arab, E. Parrish, T. İslamoğlu, H. M. El-Kaderi, Synthesis and evaluation of porous azo-linked polymers for carbon dioxide capture and separation, *J. Mater. Chem. A*, **2015**, *3*, 20586-20594.

[21] T. Islamoglu, T. Kim, Z. Kahveci, O. M. El-Kadri, H. M. El-Kaderi, Systematic Postsynthetic Modiﬁcation of Nanoporous OrganicFrameworks for Enhanced CO_2_ Capture from Flue Gas and Landﬁll Gas, *J. Phys. Chem. C* **2016**, *120*, 2592-2599.

[22] H. A. Patel, S. H. Je, J. Park, D. P. Chen, Y. Jung, C. T. Yavuz, A. Coskun, Unprecedented high-temperature CO_2_ selectivity in N_2_-phobic nanoporous covalent organic polymers, *Nat. Commun.* **2013**, *4*, 1357.

[23] O. Buyukcakir, S. H. Je, S. N. Talapaneni, D. Kim, A. Coskun, Charged Covalent Triazine Frameworks for CO_2_ Capture and Conversion, *ACS Appl. Mater. Interfaces* **2017**, *9*, 7209-7216.

[24] K. S. Song, S. N. Talapaneni, T. Ashirov, A. Coskun, Molten Salt Templated Synthesis of Covalent Isocyanurate Frameworks with Tunable Morphology and High CO_2_ Uptake Capacity, *ACS Appl. Mater. Interfaces* **2021**, *13*, 26102-26108.

[25] J. Byun, S.-H. Je, H. A. Patel, A. Coskun, C. T. Yavuz, Nanoporous covalent organic polymers incorporating Troger's base functionalities for enhanced CO_2_ capture, J. Mater. Chem. A **2014**, *2*, 12507-12512.

[26] T. Ashirov, K. S. Song, A. Coskun, Salt-Templated Solvothermal Synthesis of Dioxane-Linked Three Dimensional Nanoporous Organic Polymers for Carbon Dioxide and Iodine Capture, *ACS Appl. Nano Mater.* **2022**, *5*, 10, 13711-13719.

[27] W. Lu, D. Yuan, J. Sculley, D. Zhao, R. Krishna, H.-C. Zhou, Sulfonate-Grafted Porous Polymer Networks for Preferential CO_2_ Adsorption at Low Pressure, *J. Am. Chem. Soc.* **2011**, *133*, 45, 18126-18129.

[28] W. Lu, W. M. Verdegaal, J. Yu, P. B. Balbuena, H.-K. Jeong, H.-C. Zhou, Building multiple adsorption sites in porous polymer networks for carbon capture applications, *Energy Environ. Sci.*, **2013**, *6*, 3559-3564.

[29] L. Zong, X. Li, P. Cai, H.-C. Zhou, N. Huang, β-Ketoenamine Porous Organic Polymers for High Efficiency Carbon Dioxide Adsorption and Separation, *ChemSusChem* **2025**, *18*, e202401500.

[30] L. Yang, P. Cai, X. Jin, Z. Wang, H.-C. Zhou, N. Huang, Phenolic Resin-type Microporous Organic Polymers for High-Performance Carbon Dioxide Adsorption, *Chem Asian J.* **2025**, *20*, e202401288.

[31] X. Zhu, C.-L. Do-Thanh, C. R. Murdock, K. M. Nelson, C. Tian, S. Brown, S. M. Mahurin, D. M. Jenkins, J. Hu, B. Zhao, H. Liu, S. Dai, Efficient CO_2_ Capture by a 3D Porous Polymer Derived from Tröger’s Base, *ACS Macro Lett.* **2013**, *2*, 660-663.

[32] T. Jin, Y. Xiong, X. Zhu, Z. Tian, D.-J. Tao, J. Hu, D. Jiang, H. Wang, H. Liu, S, Dai, Rational design and synthesis of a porous, task specific polycarbazole for efficient CO_2_ capture, *Chem. Commun.* **2016**, 52, 4454-4457.

[33] MD. W. Hussain, S. Bandyopadhyay, A. Patra, Microporous organic polymers involving thiadiazolopyridine for high and selective uptake of greenhouse gases at low pressure, *Chem. Commun.* **2017**, *53*, 10576-10579.

[34] S.-H. Jia, X. Ding, H.-T. Yu, B.-H. Han, Multi-hydroxyl-containing porous organic polymers based on phenol formaldehyde resin chemistry with high carbon dioxide capture capacity, *RSC Adv.* **2015**, *5*, 71095-71101.

[35] S. K. Das, P. Bhanja, S. K. Kundu, S. Mondal, A. Bhaumik, Role of Surface Phenolic-OH Groups in N‑Rich Porous Organic Polymers for Enhancing the CO_2_ Uptake and CO_2_/N_2_ Selectivity: Experimental and Computational Studies, *ACS Appl. Mater. Interfaces* **2018**, *10*, 23813-23824.

[36] G. Li, B. Zhang, J. Yan, Z. Wang, Tetraphenyladamantane-Based Polyaminals for Highly Efficient Captures of CO_2_ and Organic Vapors, *Macromolecules* **2014**, *47*, 6664-6670.

[37] O. Buyukcakir, R. Yuksel, Y. Jiang, S. H. Lee, W. K. Seong, X. Chen, and R. S. Ruoff, Synthesis of Porous Covalent Quinazoline Networks (CQNs) and Their Gas Sorption Properties, *Angew. Chem. Int. Ed.* **2019**, *58*, 872-876.

[38] Y. Zhu, H. Long, W. Zhang, Imine-Linked Porous Polymer Frameworks with High Small Gas (H_2_, CO_2_, CH_4_, C_2_H_2_) Uptake and CO_2_/N_2_ Selectivity, *Chem. Mater.* **2013**, *25*, 1630-1635.

[39] M. Zhang, Z. Perry, J. Park, H.-C. Zhou, Stable benzimidazole-incorporated porous polymer network for carbon capture with high efficiency and low cost, *Polymer* **2014**, 335-339.

[40] X. Zhu, C. Tian, G. M. Veith, C. W. Abney, J. Dehaudt, S. Dai, *In Situ* Doping Strategy for the Preparation of Conjugated Triazine Frameworks Displaying Eﬃcient CO_2_ Capture Performance, *J. Am. Chem. Soc.* **2016**, *138*, 11497-11500.

[41] Y. Zhao, K. X. Yao, B. Teng, T. Zhang, Y. Han, A perfluorinated covalent triazine-based framework for highly selective and water–tolerant CO_2_ capture, *Energy Environ. Sci.* **2013**, *6*, 3684-3692.

[42] Z. Yang, S. Wang, Z. Zhang, W. Guo, K. Jie, M. I. Hashim, O. S. Miljanić, D. Jiang, I. Popovs, S. Dai, Influence of fluorination on CO_2_ adsorption in materials derived from fluorinated covalent triazine framework precursors, *J. Mater. Chem. A* **2019**, *7*, 17277-17282.

[43] S. Mukherjee, M. Das, A. Manna, R. Krishna, S. Das, Dual Strategic Approach to Prepare Defluorinated Triazole Embedded Covalent Triazine Frameworks with High Gas Uptake Performance, *Chem. Mater.* **2019**, *31*, 3929-3940.

[44] G. Tuci, M. Pilaski, H. Ba, A. Rossin, L. Luconi, S. Caporali, C. Pham-Huu, R. Palkovits, G. Giambastiani, Unraveling Surface Basicity and Bulk Morphology Relationship on Covalent Triazine Frameworks with Unique Catalytic and Gas Adsorption Properties, *Adv. Funct. Mater.* **2017**, *27*, 1605672.

[45] S. Hug, L. Stegbauer, H. Oh, M. Hirscher, B. V. Lotsch, Nitrogen-Rich Covalent Triazine Frameworks as High-Performance Platforms for Selective Carbon Capture and Storage, *Chem. Mater.* **2015**, *27*, 8001-8010.

## [46] H. S. Jena, C. Krishnaraj, G. Wang, K. Leus, J. Schmidt, N. Chaoui, P. Van Der Voort, Acetylacetone Covalent Triazine Framework: An Efficient Carbon Capture and Storage Material and a Highly Stable Heterogeneous Catalyst, Chem. Mater. **2018**, *30*, 4102-4111.

[47] G. Wang, K. Leus, H. S. Jena, C. Krishnaraj, S. Zhao, H. Depauw, N. Tahir, Y.-Y. Liu, P. Van Der Voort A fluorine-containing hydrophobic covalent triazine framework with excellent selective CO_2_ capture performance, *J. Mater. Chem. A* **2018**, *6*, 6370-6375.

[48] Y. J. Lee, S. Naidu Talapaneni, A. Coskun, Chemically Activated Covalent Triazine Frameworks with Enhanced Textural Properties for High Capacity Gas Storage, *ACS Appl. Mater. Interfaces* **2017**, *9*, 30679-30685.

[49] A. K. Maharana, S. K. Sarkar, S. Mukherjee, R. Sarkar, G. Rambabu, K. Sugamata, S. Das, Triazole-boosted dual-structured covalent triazine frameworks for ultra-stable high-energy and -power density aqueous supercapacitors and notable selective CO_2_ capture, *J. Mater. Chem. A* **2025**, *13*, 11717-11731.

[50] J.-B. Lin, T. T. T. Nguyen, R. Vaidhyanathan, J. Burner, J. M. Taylor, H. Durekova, F. Akhtar, R. K. Mah, O. Ghaffari-Nik, S. Marx, N. Fylstra, S. S. Iremonger, K. W. Dawson, P. Sarkar, P. Hovington, A. Rajendran, T. K. Woo, G. K. H. Shimizu, A scalable metal-organic framework as a durable physisorbent for carbon dioxide capture, *Science* **2021**, *374*, 1464-1469.

[51] S. Cavenati, C. A. Grande, A. Â. E. Rodrigues, Adsorption Equilibrium of Methane, Carbon Dioxide, and Nitrogen on Zeolite 13X at High Pressures, *J. Chem. Eng. Data*, **2004**, *49*, 1095-1101.

# [52] T. Terao, S. Maeda, A. Saika, High-resolution solid-state carbon-13 NMR of poly(vinyl alcohol): enhancement of tacticity splitting by intramolecular hydrogen bonds. *Macromolecules* **1983**, *16*, 1535.
